# Supplementary material for: Downregulation of miR-192 Alleviates Oxidative Stress-Induced Porcine Granulosa Cell Injury by Directly Targeting Acvr2a
Source: Cells. 2022 Aug 1;11(15):2362. doi: 10.3390/cells11152362 (PMC9368079; doi:10.3390/cells11152362)
Supplement: Supplementary file 1 [file cells-11-02362-s001.zip › cells-1822791-supplementary.pdf]

Supplementary Information for

Downregulation of miR-192 alleviates oxidative stress-induced porcine granulosa cell injury by directly targeting acvr2a

Supplementary Table S1. Up- and down-regulated miRNAs in H2O2 treated porcine granulosa cells

Low concentration (100 μM) versus control group				
**miRNA ID**	**Mature sequence (5'-3')**	**p-value**	**log2FC**	**Regulation**
ssc-miR-339_L-2	CCTGTCCTCCAGGAGCTCA	1.92×10^{-2}	-2.68	down
ssc-mir-374a-p5	CTTATCAGGTTGTATTGTAA	3.84×10^{-2}	-2.46	down
mmu-miR-1983_L-2R+1	CACCTGGAGCATGTTTTCTT	2.95×10^{-2}	-2.40	down
hsa-miR-26a-1-3p	CCTATTCTTGGTTACTTGCACG	1.81×10^{-2}	-1.93	down
mmu-miR-2137_L-2R-1	CGGCGGGAGCCCCGGGGA	3.18×10^{-2}	-1.75	down
hsa-miR-149-3p_L+1	GAGGGAGGGACGGGGGCTGTGC	4.23×10^{-2}	-1.72	down
hsa-miR-4508_L+2R-1	AAGCGGGGCTGGGCGCGC	4.82×10^{-2}	-1.16	down
sha-mir-24-1-p3_1ss2GC	TCGATTGGACCCGCCCTCCG	3.84×10^{-3}	-1.11	down
ssc-miR-192	CTGACCTATGAATTGACAGCC	3.73×10^{-3}	1.63	up
ssc-miR-139-3p	TGGAGACGCGGCCCTGTTGGAGT	2.24×10^{-2}	1.76	up
hsa-miR-27a-5p	AGGGCTTAGCTGCTTGTGAGCA	2.07×10^{-3}	1.82	up
ssc-miR-194b-5p_1	TGTAACAGCAACTCCATGTGGA	3.78×10^{-3}	1.88	up
ssc-miR-338_R+1	TCCAGCATCAGTGATTTTGTTGA	4.72×10^{-2}	2.21	up
ssc-miR-1839-3p_R+2	AGACCTACTTTTCTACCAACAGC	1.82×10^{-2}	2.60	up
ssc-miR-190b	TGATATGTTTGATATTGGGTTG	2.78×10^{-2}	2.65	up
PC-5p-9551_196	CTCCCTGGGCTCTGCCTCCC	3.73×10^{-2}	2.78	up
hsa-miR-222-5p_L+2R-1	GGCTCAGTAGCCAGTGTAGATCC	1.70×10^{-3}	2.88	up
hsa-miR-141-3p_R+1	TAACACTGTCTGGTAAAGATGGC	2.87×10^{-3}	3.31	up
hsa-mir-1973-p5	GTATTCTGACCGTGCAAAGGTAGCA	1.29×10^{-3}	3.95	up
ssc-miR-215_R+1	ATGACCTATGAATTGACAGACA	5.18×10^{-3}	3.96	up
hsa-miR-200c-3p	TAATACTGCCGGGTAATGATGGA	1.15×10^{-2}	4.23	up
PC-5p-11132_163	TGTCTAGAAAGTCTGTTGT	3.39×10^{-2}	4.48	up
**High concentration (300 μM) versus control group**				

cfa-miR-8903_R-2_1ss21GA	TCTTGGGCCCCACCCCCGGAAA	1.43×10^{-2}	-2.96	down
hsa-miR-873-3p_L-1_1ss11GA	GAGACTGATAAGTTCCCGGGA	3.86×10^{-2}	-2.51	down
PC-3p-39317_30	TGCCTCCCTCTGTGCCCACAGT	4.90×10^{-2}	-2.22	down
sha-miR-21_L+2R-2	AATAGCTTATCAGACTGATGTTGAC	3.02×10^{-3}	-1.68	down
ssc-miR-27b-5p_R-1	AGAGCTTAGCTGATTGGTGAAC	2.16×10^{-3}	-1.52	down
mmu-miR-26a-2-3p_1ss4GA	CCTATTCTTGATTACTTGTTTC	2.40×10^{-3}	-1.52	down
mmu-miR-107-5p	AGCTTCTTTACAGTGTTGCCTTG	2.28×10^{-3}	-1.26	down
hsa-miR-129-1-3p	AAGCCCTTACCCCAAAAAGTAT	5.35×10^{-3}	-1.23	down
sha-mir-24-1-p3_1ss2GC	TCGATTGGACCCGCCCTCCG	1.72×10^{-3}	-1.20	down
hsa-miR-31-3p_R+1	TGCTATGCCAACATATTGCCATC	1.17×10^{-3}	-1.16	down
hsa-miR-590-5p_1ss19GA	GAGCTTATTCATAAAAGTACAG	1.29×10^{-2}	-1.13	down
hsa-miR-23b-5p	TGGGTTCCTGGCATGCTGATTT	7.44×10^{-3}	-1.11	down
ssc-miR-181d-5p	AACATTCATTGTTGTCGGTGGGTT	3.99×10^{-3}	-1.10	down
ssc-miR-19b	TGTGCAAATCCATGCAAAACTGA	1.02×10^{-2}	-1.06	down
bta-mir-2478-p3_1ss20GA	ATCCCACTTCTGACACCATA	2.32×10^{-2}	1.87	up
hsa-miR-222-5p_L+2R-1	GGCTCAGTAGCCAGTGTAGATCC	9.18×10^{-3}	1.92	up
ssc-miR-424-5p_R-3	CAGCAGCAATTCATGTTTT	9.36×10^{-3}	1.93	up
ssc-miR-339_R-2	TCCCTGTCCTCCAGGAGCT	1.94×10^{-3}	1.96	up
ssc-miR-192	CTGACCTATGAATTGACAGCC	3.66×10^{-2}	2.19	up
ssc-miR-1839-3p_R+2	AGACCTACTTTTCTACCAACAGC	3.88×10^{-2}	2.29	up
mmu-let-7j_R-2	TGAGGTATTAGTTTGTGCTGTT	3.27×10^{-2}	2.50	up
ssc-miR-190b	TGATATGTTTGATATTGGGTTG	2.71×10^{-3}	2.86	up
PC-5p-9551_196	CTCCCTGGGCTCTGCCTCCC	2.49×10^{-2}	2.97	up
hsa-miR-375	TTTGTTCGTTCGGCTCGCGTGA	4.76×10^{-3}	3.13	up
hsa-miR-141-3p_R+1	TAACACTGTCTGGTAAAGATGGC	4.43×10^{-2}	3.83	up
PC-3p-21836_71	CTCTTTTCTTTGTGAAGG	2.08×10^{-2}	4.08	up
ssc-miR-215_R+1	ATGACCTATGAATTGACAGACA	1.59×10^{-3}	4.12	up
hsa-miR-1246_L+1R+2	AAATGGATTTTTGGAGCAGGGA	1.02×10^{-2}	4.15	up
PC-5p-12443_142	CAGCCTCTGGCATGTTGGA	1.81×10^{-2}	4.52	up
hsa-miR-4792_1ss9GT	CGGTGAGCTCTCGCTGGC	4.27×10^{-2}	4.66	up
PC-5p-6765_300	TGTGCTTGGCTGAGGAGC	6.38×10^{-2}	5.85	up
mmu-mir-6240-p5_1ss13AG	TCGCGAAGGCCCGCGGCGG	3.83×10^{-5}	5.99	up

ssc-mir-4332-p5_1ss18CA	CGGCCGCCGCCGGGCGCATT	3.98×10^{-2}	7.58	up

|Log_2(Fold change)| ≥ 1 and p-value < 0.05 was the cut-off criteria for DEmiRNAs.

Supplementary Table S2. Predicted target genes of differentially expressed miRNAs in100μM H_2O_2 treated porcine GCs

Gene ID	Symbol	miRNA ID
ENSSSCG00000000002	GTSE1	mmu-miR-1983_L-2R+1
ENSSSCG00000000003	TTC38	ssc-miR-192
ENSSSCG00000000003	TTC38	ssc-miR-215_R+1
ENSSSCG00000000003	TTC38	ssc-miR-338_R+1
ENSSSCG00000000011	ENSSSCG00000000011	ssc-miR-339_L-2
ENSSSCG00000000013	ENSSSCG00000000013	ssc-miR-194b-5p_1ss10GA
ENSSSCG00000000018	ENSSSCG00000000018	mmu-miR-2137_L-2R-1_1ss16AG
ENSSSCG00000000029	SCUBE1	hsa-miR-149-3p_L+1
ENSSSCG00000000029	SCUBE1	hsa-miR-4508_L+2R-1
ENSSSCG00000000029	SCUBE1	mmu-miR-2137_L-2R-1_1ss16AG
ENSSSCG00000000029	SCUBE1	ssc-miR-339_L-2
ENSSSCG00000000031	MCAT	PC-5p-9551_196
ENSSSCG00000000036	PACSIN2	PC-5p-9551_196
ENSSSCG00000000036	PACSIN2	hsa-miR-141-3p_R+1
ENSSSCG00000000040	43346	hsa-miR-149-3p_L+1
ENSSSCG00000000040	43346	ssc-miR-192
ENSSSCG00000000040	43346	ssc-miR-215_R+1
ENSSSCG00000000040	43346	ssc-miR-339_L-2
ENSSSCG00000000045	ENSSSCG00000000045	ssc-miR-338_R+1
ENSSSCG00000023108	ENSSSCG00000023108	hsa-miR-149-3p_L+1
ENSSSCG00000023108	ENSSSCG00000023108	hsa-miR-222-5p_L+2R-1
ENSSSCG00000000058	SNU13	hsa-miR-149-3p_L+1
ENSSSCG00000000058	SNU13	mmu-miR-2137_L-2R-1_1ss16AG
ENSSSCG00000000059	XRCC6	ssc-miR-194b-5p_1ss10GA
ENSSSCG00000000060	DESI1	PC-5p-9551_196
ENSSSCG00000000064	ACO2	mmu-miR-2137_L-2R-1_1ss16AG
ENSSSCG00000000066	L3MBTL2	PC-5p-9551_196
ENSSSCG00000000066	L3MBTL2	hsa-miR-222-5p_L+2R-1
ENSSSCG00000000066	L3MBTL2	ssc-miR-139-3p
ENSSSCG00000000066	L3MBTL2	ssc-miR-1839-3p_R+2
ENSSSCG00000000066	L3MBTL2	ssc-miR-338_R+1
ENSSSCG00000000067	CHADL	hsa-miR-27a-5p

ENSSSCG00000000072	SLC25A17	hsa-miR-200c-3p
ENSSSCG00000000077	ADSL	hsa-mir-1973-p5_1ss5CT
ENSSSCG00000000080	GRAP2	PC-5p-9551_196
ENSSSCG00000000082	CACNA1I	hsa-miR-4508_L+2R-1
ENSSSCG00000000093	DNAL4	mmu-miR-2137_L-2R-1_1ss16AG
ENSSSCG00000000095	GTPBP1	hsa-miR-149-3p_L+1
ENSSSCG00000000095	GTPBP1	hsa-miR-27a-5p
ENSSSCG00000000095	GTPBP1	hsa-miR-4508_L+2R-1
ENSSSCG00000000107	CSNK1E	hsa-miR-200c-3p
ENSSSCG00000000107	CSNK1E	hsa-miR-4508_L+2R-1
ENSSSCG00000000111	BAIAP2L2	ssc-miR-139-3p
ENSSSCG00000000117	ENSSSCG00000000117	PC-5p-9551_196
ENSSSCG00000000120	ANKRD54	ssc-miR-338_R+1
ENSSSCG00000029945	ENSSSCG00000029945	PC-5p-9551_196
ENSSSCG00000000130	CYTH4	PC-5p-9551_196
ENSSSCG00000000130	CYTH4	hsa-miR-26a-1-3p
ENSSSCG00000000130	CYTH4	hsa-mir-1973-p5_1ss5CT
ENSSSCG00000000134	MPST	ssc-miR-339_L-2
ENSSSCG00000000136	CSF2RB	hsa-miR-149-3p_L+1
ENSSSCG00000000136	CSF2RB	hsa-miR-27a-5p
ENSSSCG00000000137	NCF4	PC-5p-9551_196
ENSSSCG00000000137	NCF4	ssc-miR-139-3p
ENSSSCG00000000138	PVALB	sha-mir-24-1-p3_1ss2GC
ENSSSCG00000000139	IFT27	ssc-miR-139-3p
ENSSSCG00000000142	FOXRED2	hsa-miR-149-3p_L+1
ENSSSCG00000000142	FOXRED2	hsa-miR-222-5p_L+2R-1
ENSSSCG00000000142	FOXRED2	hsa-miR-26a-1-3p
ENSSSCG00000000142	FOXRED2	mmu-miR-1983_L-2R+1
ENSSSCG00000000142	FOXRED2	mmu-miR-2137_L-2R-1_1ss16AG
ENSSSCG00000000142	FOXRED2	ssc-miR-1839-3p_R+2
ENSSSCG00000000142	FOXRED2	ssc-miR-339_L-2
ENSSSCG00000000144	TXN2	PC-5p-9551_196
ENSSSCG00000000144	TXN2	mmu-miR-2137_L-2R-1_1ss16AG
ENSSSCG00000000144	TXN2	ssc-miR-139-3p
ENSSSCG00000000157	BPIFC	PC-5p-9551_196
ENSSSCG00000000157	BPIFC	ssc-miR-194b-5p_1ss10GA
ENSSSCG00000000158	RTCB	hsa-miR-141-3p_R+1
ENSSSCG00000000160	PRDM4	mmu-miR-2137_L-2R-1_1ss16AG
ENSSSCG00000000162	BTBD11	ssc-miR-190b
ENSSSCG00000000175	ENSSSCG00000000175	PC-5p-9551_196
ENSSSCG00000000176	CACNB3	PC-5p-9551_196
ENSSSCG00000000176	CACNB3	mmu-miR-1983_L-2R+1
ENSSSCG00000000181	RND1	hsa-miR-149-3p_L+1
ENSSSCG00000000181	RND1	ssc-miR-339_L-2

ENSSSCG00000000191	NR5A1	PC-5p-9551_196
ENSSSCG00000000195	PRPH	hsa-miR-27a-5p
ENSSSCG00000000195	PRPH	mmu-miR-2137_L-2R-1_1ss16AG
ENSSSCG00000000203	ENSSSCG00000000203	hsa-mir-1973-p5_1ss5CT
ENSSSCG00000000206	FAIM2	PC-5p-11132_163
ENSSSCG00000000206	FAIM2	hsa-miR-149-3p_L+1
ENSSSCG00000000211	AQP5	mmu-miR-2137_L-2R-1_1ss16AG
ENSSSCG00000000214	ENSSSCG00000000214	PC-5p-9551_196
ENSSSCG00000000214	ENSSSCG00000000214	hsa-miR-141-3p_R+1
ENSSSCG00000000216	ENSSSCG00000000216	PC-5p-9551_196
ENSSSCG00000000216	ENSSSCG00000000216	hsa-miR-149-3p_L+1
ENSSSCG00000000216	ENSSSCG00000000216	ssc-miR-139-3p
ENSSSCG00000000223	ENSSSCG00000000223	hsa-miR-149-3p_L+1
ENSSSCG00000000223	ENSSSCG00000000223	hsa-miR-26a-1-3p
ENSSSCG00000000223	ENSSSCG00000000223	hsa-mir-1973-p5_1ss5CT
ENSSSCG00000000223	ENSSSCG00000000223	ssc-miR-192
ENSSSCG00000000223	ENSSSCG00000000223	ssc-miR-215_R+1
ENSSSCG00000000223	ENSSSCG00000000223	ssc-miR-338_R+1
ENSSSCG00000000224	SMAGP	hsa-miR-149-3p_L+1
ENSSSCG00000000232	ACVRL1	PC-5p-9551_196
ENSSSCG00000000232	ACVRL1	ssc-miR-1839-3p_R+2
ENSSSCG00000000232	ACVRL1	ssc-miR-339_L-2
ENSSSCG00000000233	ACVR1B	sha-mir-24-1-p3_1ss2GC
ENSSSCG00000000235	KRT82	PC-5p-9551_196
ENSSSCG00000000235	KRT82	mmu-miR-2137_L-2R-1_1ss16AG
ENSSSCG00000000235	KRT82	ssc-miR-339_L-2
ENSSSCG00000000243	ENSSSCG00000000243	ssc-mir-374a-p5
ENSSSCG00000000244	PPBP	ssc-miR-339_L-2
ENSSSCG00000000246	ENSSSCG00000000246	hsa-miR-27a-5p
ENSSSCG00000000248	KRT5	mmu-miR-2137_L-2R-1_1ss16AG
ENSSSCG00000000251	KRT1	ssc-miR-192
ENSSSCG00000000251	KRT1	ssc-miR-215_R+1
ENSSSCG00000000252	KRT8	hsa-mir-1973-p5_1ss5CT
ENSSSCG00000000258	RARG	PC-5p-9551_196
ENSSSCG00000000258	RARG	hsa-miR-149-3p_L+1
ENSSSCG00000000258	RARG	mmu-miR-2137_L-2R-1_1ss16AG
ENSSSCG00000000259	CSAD	hsa-miR-141-3p_R+1
ENSSSCG00000000259	CSAD	hsa-miR-149-3p_L+1
ENSSSCG00000000259	CSAD	ssc-miR-139-3p
ENSSSCG00000000262	SPRYD3	hsa-miR-141-3p_R+1
ENSSSCG00000000262	SPRYD3	hsa-miR-149-3p_L+1
ENSSSCG00000000268	AAAS	PC-5p-9551_196
ENSSSCG00000000268	AAAS	hsa-miR-141-3p_R+1
ENSSSCG00000000268	AAAS	mmu-miR-1983_L-2R+1

ENSSSCG00000000271	AMHR2	PC-5p-9551_196
ENSSSCG00000000271	AMHR2	ssc-miR-339_L-2
ENSSSCG00000000274	PCBP2	mmu-miR-1983_L-2R+1
ENSSSCG00000000290	COPZ1	hsa-miR-149-3p_L+1
ENSSSCG00000000293	ITGA5	PC-5p-9551_196
ENSSSCG00000000293	ITGA5	hsa-miR-149-3p_L+1
ENSSSCG00000000293	ITGA5	ssc-miR-339_L-2
ENSSSCG00000000297	ENSSSCG00000000297	hsa-miR-4508_L+2R-1
ENSSSCG00000000297	ENSSSCG00000000297	mmu-miR-1983_L-2R+1
ENSSSCG00000000297	ENSSSCG00000000297	ssc-miR-339_L-2
ENSSSCG00000000361	CD63	mmu-miR-1983_L-2R+1
ENSSSCG00000000363	GDF11	hsa-miR-149-3p_L+1
ENSSSCG00000000363	GDF11	hsa-miR-26a-1-3p
ENSSSCG00000000366	DNAJC14	ssc-miR-192
ENSSSCG00000000366	DNAJC14	ssc-miR-215_R+1
ENSSSCG00000000369	PYM1	PC-5p-9551_196
ENSSSCG00000000369	PYM1	hsa-miR-141-3p_R+1
ENSSSCG00000000369	PYM1	hsa-mir-1973-p5_1ss5CT
ENSSSCG00000000370	ENSSSCG00000000370	hsa-miR-200c-3p
ENSSSCG00000000373	CDK2	hsa-miR-200c-3p
ENSSSCG00000000377	ENSSSCG00000000377	PC-5p-9551_196
ENSSSCG00000000377	ENSSSCG00000000377	hsa-miR-149-3p_L+1
ENSSSCG00000000379	ESYT1	PC-5p-9551_196
ENSSSCG00000000379	ESYT1	ssc-miR-339_L-2
ENSSSCG00000000382	ENSSSCG00000000382	PC-5p-9551_196
ENSSSCG00000000382	ENSSSCG00000000382	hsa-miR-149-3p_L+1
ENSSSCG00000000382	ENSSSCG00000000382	ssc-miR-139-3p
ENSSSCG00000000383	ENSSSCG00000000383	PC-5p-9551_196
ENSSSCG00000000385	SLC39A5	mmu-miR-1983_L-2R+1
ENSSSCG00000000399	TIMELESS	hsa-miR-149-3p_L+1
ENSSSCG00000000399	TIMELESS	hsa-miR-222-5p_L+2R-1
ENSSSCG00000000401	ENSSSCG00000000401	mmu-miR-1983_L-2R+1
ENSSSCG00000000403	BAZ2A	hsa-miR-149-3p_L+1
ENSSSCG00000000403	BAZ2A	hsa-miR-27a-5p
ENSSSCG00000000406	PTGES3	hsa-miR-141-3p_R+1
ENSSSCG00000000406	PTGES3	ssc-mir-374a-p5
ENSSSCG00000000408	ENSSSCG00000000408	mmu-miR-1983_L-2R+1
ENSSSCG00000025960	ENSSSCG00000025960	ssc-miR-338_R+1
ENSSSCG00000000411	ENSSSCG00000000411	hsa-miR-149-3p_L+1
ENSSSCG00000025542	ENSSSCG00000025542	PC-5p-9551_196
ENSSSCG00000000418	TAC3	mmu-miR-2137_L-2R-1_1ss16AG
ENSSSCG00000000420	ENSSSCG00000000420	hsa-miR-4508_L+2R-1
ENSSSCG00000000420	ENSSSCG00000000420	mmu-miR-2137_L-2R-1_1ss16AG
ENSSSCG00000000423	ENSSSCG00000000423	hsa-miR-200c-3p

ENSSSCG00000000426	ENSSSCG00000000426	hsa-miR-149-3p_L+1
ENSSSCG00000026524	ENSSSCG00000026524	ssc-miR-338_R+1
ENSSSCG00000000433	B4GALNT1	ssc-miR-139-3p
ENSSSCG00000000437	ENSSSCG00000000437	PC-5p-9551_196
ENSSSCG00000000437	ENSSSCG00000000437	hsa-miR-149-3p_L+1
ENSSSCG00000000437	ENSSSCG00000000437	mmu-miR-1983_L-2R+1
ENSSSCG00000000437	ENSSSCG00000000437	ssc-miR-139-3p
ENSSSCG00000000437	ENSSSCG00000000437	ssc-miR-1839-3p_R+2
ENSSSCG00000000437	ENSSSCG00000000437	ssc-miR-192
ENSSSCG00000000439	KIF5A	PC-5p-9551_196
ENSSSCG00000000439	KIF5A	hsa-miR-149-3p_L+1
ENSSSCG00000000443	ENSSSCG00000000443	ssc-miR-139-3p
ENSSSCG00000000443	ENSSSCG00000000443	ssc-miR-1839-3p_R+2
ENSSSCG00000000443	ENSSSCG00000000443	ssc-miR-339_L-2
ENSSSCG00000000443	ENSSSCG00000000443	ssc-mir-374a-p5
ENSSSCG00000000456	SLC16A7	PC-5p-9551_196
ENSSSCG00000000456	SLC16A7	hsa-mir-1973-p5_1ss5CT
ENSSSCG00000000456	SLC16A7	ssc-miR-192
ENSSSCG00000000456	SLC16A7	ssc-miR-215_R+1
ENSSSCG00000000457	ENSSSCG00000000457	hsa-mir-1973-p5_1ss5CT
ENSSSCG00000000457	ENSSSCG00000000457	ssc-miR-1839-3p_R+2
ENSSSCG00000000457	ENSSSCG00000000457	ssc-miR-338_R+1
ENSSSCG00000000458	MON2	ssc-miR-192
ENSSSCG00000000458	MON2	ssc-miR-215_R+1
ENSSSCG00000028869	ENSSSCG00000028869	ssc-miR-190b
ENSSSCG00000000481	IFNG	hsa-mir-1973-p5_1ss5CT
ENSSSCG00000000481	IFNG	ssc-mir-374a-p5
ENSSSCG00000000500	ENSSSCG00000000500	hsa-miR-149-3p_L+1
ENSSSCG00000000510	TMEM19	hsa-miR-141-3p_R+1
ENSSSCG00000000512	TBC1D15	hsa-miR-141-3p_R+1
ENSSSCG00000000512	TBC1D15	ssc-miR-190b
ENSSSCG00000000519	GLIPR1	hsa-miR-26a-1-3p
ENSSSCG00000000519	GLIPR1	ssc-miR-1839-3p_R+2
ENSSSCG00000000519	GLIPR1	ssc-miR-192
ENSSSCG00000000519	GLIPR1	ssc-miR-215_R+1
ENSSSCG00000000520	KRR1	PC-5p-11132_163
ENSSSCG00000000520	KRR1	PC-5p-9551_196
ENSSSCG00000000520	KRR1	hsa-miR-141-3p_R+1
ENSSSCG00000000520	KRR1	hsa-miR-222-5p_L+2R-1
ENSSSCG00000000520	KRR1	hsa-miR-26a-1-3p
ENSSSCG00000000520	KRR1	mmu-miR-1983_L-2R+1
ENSSSCG00000000522	NAP1L1	ssc-miR-194b-5p_1ss10GA
ENSSSCG00000000529	DNM1L	PC-5p-11132_163
ENSSSCG00000000529	DNM1L	ssc-miR-1839-3p_R+2

ENSSSCG00000000529	DNM1L	ssc-miR-190b
ENSSSCG00000000529	DNM1L	ssc-miR-194b-5p_1ss10GA
ENSSSCG00000000529	DNM1L	ssc-miR-339_L-2
ENSSSCG00000000529	DNM1L	ssc-mir-374a-p5
ENSSSCG00000000534	IPO8	hsa-miR-149-3p_L+1
ENSSSCG00000000534	IPO8	hsa-miR-200c-3p
ENSSSCG00000000534	IPO8	mmu-miR-1983_L-2R+1
ENSSSCG00000000534	IPO8	ssc-miR-339_L-2
ENSSSCG00000000536	ERGIC2	hsa-miR-27a-5p
ENSSSCG00000000536	ERGIC2	hsa-mir-1973-p5_1ss5CT
ENSSSCG00000000536	ERGIC2	ssc-miR-194b-5p_1ss10GA
ENSSSCG00000000539	ENSSSCG00000000539	hsa-miR-141-3p_R+1
ENSSSCG00000000539	ENSSSCG00000000539	ssc-miR-190b
ENSSSCG00000000541	FAR2	hsa-mir-1973-p5_1ss5CT
ENSSSCG00000000541	FAR2	ssc-mir-374a-p5
ENSSSCG00000000544	PTHLH	hsa-miR-200c-3p
ENSSSCG00000000544	PTHLH	ssc-miR-190b
ENSSSCG00000000545	KLHL42	ssc-miR-192
ENSSSCG00000000545	KLHL42	ssc-miR-215_R+1
ENSSSCG00000000547	MRPS35	hsa-miR-141-3p_R+1
ENSSSCG00000000547	MRPS35	hsa-miR-200c-3p
ENSSSCG00000000549	PPFIBP1	hsa-miR-149-3p_L+1
ENSSSCG00000000549	PPFIBP1	hsa-miR-200c-3p
ENSSSCG00000000552	MED21	hsa-mir-1973-p5_1ss5CT
ENSSSCG00000000553	TM7SF3	PC-5p-9551_196
ENSSSCG00000000553	TM7SF3	ssc-miR-338_R+1
ENSSSCG00000000554	FGFR1OP2	hsa-miR-141-3p_R+1
ENSSSCG00000000554	FGFR1OP2	hsa-miR-149-3p_L+1
ENSSSCG00000000554	FGFR1OP2	hsa-miR-222-5p_L+2R-1
ENSSSCG00000000555	ENSSSCG00000000555	hsa-mir-1973-p5_1ss5CT
ENSSSCG00000000555	ENSSSCG00000000555	ssc-miR-194b-5p_1ss10GA
ENSSSCG00000000577	GYS2	hsa-miR-200c-3p
ENSSSCG00000000579	GOLT1B	ssc-miR-194b-5p_1ss10GA
ENSSSCG00000000580	RECQL	hsa-mir-1973-p5_1ss5CT
ENSSSCG00000000580	RECQL	ssc-miR-1839-3p_R+2
ENSSSCG00000000583	PYROXD1	PC-5p-9551_196
ENSSSCG00000000583	PYROXD1	hsa-miR-222-5p_L+2R-1
ENSSSCG00000000584	SLCO1A2	PC-5p-9551_196
ENSSSCG00000000584	SLCO1A2	ssc-miR-1839-3p_R+2
ENSSSCG00000000584	SLCO1A2	ssc-miR-339_L-2
ENSSSCG00000000587	AEBP2	hsa-miR-141-3p_R+1
ENSSSCG00000000587	AEBP2	hsa-miR-26a-1-3p
ENSSSCG00000000600	ENSSSCG00000000600	hsa-miR-200c-3p
ENSSSCG00000000601	ENSSSCG00000000601	hsa-miR-149-3p_L+1

ENSSSCG00000000604	ARHGDIB	hsa-miR-149-3p_L+1
ENSSSCG00000000604	ARHGDIB	hsa-mir-1973-p5_1ss5CT
ENSSSCG00000000607	ART4	hsa-miR-27a-5p
ENSSSCG00000000607	ART4	hsa-mir-1973-p5_1ss5CT
ENSSSCG00000000607	ART4	ssc-miR-1839-3p_R+2
ENSSSCG00000000611	WBP11	PC-5p-9551_196
ENSSSCG00000000611	WBP11	hsa-miR-141-3p_R+1
ENSSSCG00000000611	WBP11	ssc-miR-1839-3p_R+2
ENSSSCG00000000612	ATF7IP	ssc-miR-338_R+1
ENSSSCG00000000613	ENSSSCG00000000613	PC-5p-9551_196
ENSSSCG00000000619	FAM234B	hsa-miR-149-3p_L+1
ENSSSCG00000000619	FAM234B	mmu-miR-1983_L-2R+1
ENSSSCG00000000619	FAM234B	ssc-miR-192
ENSSSCG00000000619	FAM234B	ssc-miR-215_R+1
ENSSSCG00000000619	FAM234B	ssc-miR-338_R+1
ENSSSCG00000000620	ENSSSCG00000000620	hsa-miR-200c-3p
ENSSSCG00000000620	ENSSSCG00000000620	ssc-miR-339_L-2
ENSSSCG00000000623	BCL2L14	ssc-miR-339_L-2
ENSSSCG00000000625	LRP6	hsa-miR-149-3p_L+1
ENSSSCG00000000625	LRP6	ssc-miR-194b-5p_1ss10GA
ENSSSCG00000000633	YBX3	ssc-miR-338_R+1
ENSSSCG00000000634	STYK1	PC-5p-11132_163
ENSSSCG00000000634	STYK1	hsa-miR-222-5p_L+2R-1
ENSSSCG00000000634	STYK1	ssc-mir-374a-p5
ENSSSCG00000000640	ENSSSCG00000000640	hsa-miR-26a-1-3p
ENSSSCG00000000640	ENSSSCG00000000640	hsa-miR-27a-5p
ENSSSCG00000000640	ENSSSCG00000000640	ssc-mir-374a-p5
ENSSSCG00000000645	GABARAPL1	hsa-miR-222-5p_L+2R-1
ENSSSCG00000000645	GABARAPL1	ssc-miR-339_L-2
ENSSSCG00000000647	OLR1	ssc-miR-192
ENSSSCG00000000647	OLR1	ssc-miR-215_R+1
ENSSSCG00000000648	CLEC7A	hsa-miR-149-3p_L+1
ENSSSCG00000000648	CLEC7A	ssc-mir-374a-p5
ENSSSCG00000000648	CLEC7A	hsa-miR-149-3p_L+1
ENSSSCG00000000648	CLEC7A	ssc-mir-374a-p5
ENSSSCG00000000648	CLEC7A	hsa-miR-149-3p_L+1
ENSSSCG00000000648	CLEC7A	ssc-mir-374a-p5
ENSSSCG00000000653	CD69	ssc-mir-374a-p5
ENSSSCG00000000653	CD69	hsa-miR-141-3p_R+1
ENSSSCG00000000653	CD69	hsa-miR-26a-1-3p
ENSSSCG00000000653	CD69	ssc-mir-374a-p5
ENSSSCG00000000655	KLRF1	hsa-miR-27a-5p
ENSSSCG00000000655	KLRF1	mmu-miR-1983_L-2R+1
ENSSSCG00000000655	KLRF1	ssc-mir-374a-p5

ENSSSCG00000000662	M6PR	hsa-miR-200c-3p
ENSSSCG00000000662	M6PR	ssc-miR-194b-5p_1ss10GA
ENSSSCG00000000685	TPI1	PC-5p-9551_196
ENSSSCG00000000685	TPI1	hsa-miR-27a-5p
ENSSSCG00000000685	TPI1	ssc-miR-139-3p
ENSSSCG00000000687	CD4	hsa-miR-149-3p_L+1
ENSSSCG00000000687	CD4	hsa-miR-149-3p_L+1
ENSSSCG00000000690	ENSSSCG00000000690	hsa-miR-149-3p_L+1
ENSSSCG00000000690	ENSSSCG00000000690	hsa-miR-4508_L+2R-1
ENSSSCG00000000691	ENSSSCG00000000691	hsa-miR-149-3p_L+1
ENSSSCG00000000692	PIANP	hsa-miR-149-3p_L+1
ENSSSCG00000000702	ZNF384	hsa-miR-149-3p_L+1
ENSSSCG00000000702	ZNF384	hsa-miR-222-5p_L+2R-1
ENSSSCG00000000704	TAPBPL	PC-5p-9551_196
ENSSSCG00000000704	TAPBPL	hsa-miR-149-3p_L+1
ENSSSCG00000000704	TAPBPL	hsa-mir-1973-p5_1ss5CT
ENSSSCG00000000704	TAPBPL	ssc-miR-339_L-2
ENSSSCG00000000709	PLEKHG6	PC-5p-9551_196
ENSSSCG00000000709	PLEKHG6	hsa-miR-149-3p_L+1
ENSSSCG00000000713	ENSSSCG00000000713	ssc-miR-139-3p
ENSSSCG00000000723	C12orf4	ssc-miR-338_R+1
ENSSSCG00000000728	PARP11	PC-5p-9551_196
ENSSSCG00000000728	PARP11	hsa-mir-1973-p5_1ss5CT
ENSSSCG00000000730	PRMT8	hsa-miR-149-3p_L+1
ENSSSCG00000000730	PRMT8	ssc-miR-1839-3p_R+2
ENSSSCG00000000730	PRMT8	ssc-miR-339_L-2
ENSSSCG00000000735	TSPAN9	hsa-miR-141-3p_R+1
ENSSSCG00000000735	TSPAN9	mmu-miR-2137_L-2R-1_1ss16AG
ENSSSCG00000000736	TEAD4	hsa-miR-4508_L+2R-1
ENSSSCG00000000736	TEAD4	hsa-miR-4508_L+2R-1
ENSSSCG00000000739	FOXM1	PC-5p-11132_163
ENSSSCG00000000739	FOXM1	hsa-miR-149-3p_L+1
ENSSSCG00000000739	FOXM1	ssc-miR-339_L-2
ENSSSCG00000000742	ITFG2	ssc-mir-374a-p5
ENSSSCG00000000746	WASH1	hsa-miR-222-5p_L+2R-1
ENSSSCG00000000746	WASH1	ssc-miR-339_L-2
ENSSSCG00000000749	SLC6A12	PC-5p-11132_163
ENSSSCG00000000749	SLC6A12	ssc-miR-339_L-2
ENSSSCG00000000751	CCDC77	PC-5p-9551_196
ENSSSCG00000000753	WNK1	hsa-miR-149-3p_L+1
ENSSSCG00000000753	WNK1	ssc-miR-192
ENSSSCG00000000753	WNK1	ssc-miR-215_R+1
ENSSSCG00000000753	WNK1	ssc-miR-338_R+1
ENSSSCG00000000753	WNK1	ssc-miR-339_L-2

ENSSSCG00000000753	WNK1	ssc-mir-374a-p5
ENSSSCG00000000754	RAD52	PC-5p-9551_196
ENSSSCG00000000754	RAD52	mmu-miR-1983_L-2R+1
ENSSSCG00000000760	LRTM2	ssc-miR-139-3p
ENSSSCG00000000760	LRTM2	ssc-miR-338_R+1
ENSSSCG00000027190	ENSSSCG00000027190	hsa-miR-222-5p_L+2R-1
ENSSSCG00000027190	ENSSSCG00000027190	ssc-miR-194b-5p_1ss10GA
ENSSSCG00000000774	USP18	hsa-miR-149-3p_L+1
ENSSSCG00000000774	USP18	hsa-mir-1973-p5_1ss5CT
ENSSSCG00000000774	USP18	mmu-miR-1983_L-2R+1
ENSSSCG00000000774	USP18	ssc-miR-338_R+1
ENSSSCG00000000778	CPNE8	PC-5p-9551_196
ENSSSCG00000000778	CPNE8	hsa-mir-1973-p5_1ss5CT
ENSSSCG00000000778	CPNE8	ssc-miR-190b
ENSSSCG00000000784	LRRK2	hsa-miR-141-3p_R+1
ENSSSCG00000000784	LRRK2	hsa-miR-200c-3p
ENSSSCG00000000784	LRRK2	hsa-mir-1973-p5_1ss5CT
ENSSSCG00000000784	LRRK2	ssc-miR-338_R+1
ENSSSCG00000000784	LRRK2	ssc-mir-374a-p5
ENSSSCG00000000793	PPHLN1	hsa-miR-141-3p_R+1
ENSSSCG00000000793	PPHLN1	hsa-miR-149-3p_L+1
ENSSSCG00000000798	TWF1	PC-5p-9551_196
ENSSSCG00000000798	TWF1	hsa-miR-149-3p_L+1
ENSSSCG00000000798	TWF1	ssc-miR-190b
ENSSSCG00000000798	TWF1	ssc-miR-339_L-2
ENSSSCG00000000798	TWF1	ssc-mir-374a-p5
ENSSSCG00000000800	PUS7L	hsa-mir-1973-p5_1ss5CT
ENSSSCG00000000800	PUS7L	ssc-miR-192
ENSSSCG00000000800	PUS7L	ssc-miR-215_R+1
ENSSSCG00000000800	PUS7L	ssc-miR-339_L-2
ENSSSCG00000000806	SCAF11	hsa-miR-141-3p_R+1
ENSSSCG00000000806	SCAF11	hsa-miR-200c-3p
ENSSSCG00000000816	SENP1	PC-5p-9551_196
ENSSSCG00000000839	ALDH1L2	PC-5p-9551_196
ENSSSCG00000000839	ALDH1L2	hsa-miR-141-3p_R+1
ENSSSCG00000000839	ALDH1L2	hsa-miR-222-5p_L+2R-1
ENSSSCG00000000839	ALDH1L2	hsa-miR-27a-5p
ENSSSCG00000000839	ALDH1L2	ssc-miR-192
ENSSSCG00000000839	ALDH1L2	ssc-miR-215_R+1
ENSSSCG00000000839	ALDH1L2	PC-5p-9551_196
ENSSSCG00000000839	ALDH1L2	hsa-miR-141-3p_R+1
ENSSSCG00000000839	ALDH1L2	hsa-miR-222-5p_L+2R-1
ENSSSCG00000000839	ALDH1L2	hsa-miR-27a-5p
ENSSSCG00000000839	ALDH1L2	ssc-miR-192

ENSSSCG00000000839	ALDH1L2	ssc-miR-215_R+1
ENSSSCG00000000840	ENSSSCG00000000840	hsa-miR-141-3p_R+1
ENSSSCG00000000840	ENSSSCG00000000840	hsa-miR-222-5p_L+2R-1
ENSSSCG00000000841	APPL2	hsa-miR-141-3p_R+1
ENSSSCG00000000841	APPL2	hsa-mir-1973-p5_1ss5CT
ENSSSCG00000000841	APPL2	ssc-mir-374a-p5
ENSSSCG00000000842	KANSL2	PC-5p-11132_163
ENSSSCG00000000842	KANSL2	hsa-miR-141-3p_R+1
ENSSSCG00000000844	NFYB	hsa-miR-141-3p_R+1
ENSSSCG00000000846	HCFC2	hsa-miR-200c-3p
ENSSSCG00000000846	HCFC2	ssc-miR-1839-3p_R+2
ENSSSCG00000000846	HCFC2	ssc-miR-190b
ENSSSCG00000000846	HCFC2	ssc-miR-192
ENSSSCG00000000846	HCFC2	ssc-miR-215_R+1
ENSSSCG00000000846	HCFC2	ssc-miR-339_L-2
ENSSSCG00000000847	TDG	hsa-miR-141-3p_R+1
ENSSSCG00000000847	TDG	hsa-mir-1973-p5_1ss5CT
ENSSSCG00000000847	TDG	ssc-miR-192
ENSSSCG00000000847	TDG	ssc-miR-215_R+1
ENSSSCG00000000847	TDG	ssc-miR-338_R+1
ENSSSCG00000000854	ENSSSCG00000000854	hsa-miR-141-3p_R+1
ENSSSCG00000000862	ENSSSCG00000000862	hsa-miR-200c-3p
ENSSSCG00000000862	ENSSSCG00000000862	hsa-miR-222-5p_L+2R-1
ENSSSCG00000000863	SYCP3	ssc-miR-190b
ENSSSCG00000000863	SYCP3	ssc-miR-194b-5p_1ss10GA
ENSSSCG00000000870	ARL1	hsa-miR-200c-3p
ENSSSCG00000000870	ARL1	hsa-miR-222-5p_L+2R-1
ENSSSCG00000000870	ARL1	ssc-miR-339_L-2
ENSSSCG00000000877	ENSSSCG00000000877	hsa-miR-222-5p_L+2R-1
ENSSSCG00000000877	ENSSSCG00000000877	ssc-miR-194b-5p_1ss10GA
ENSSSCG00000025682	ENSSSCG00000025682	hsa-miR-141-3p_R+1
ENSSSCG00000000884	ENSSSCG00000000884	hsa-miR-200c-3p
ENSSSCG00000000884	ENSSSCG00000000884	ssc-miR-338_R+1
ENSSSCG00000000884	ENSSSCG00000000884	ssc-mir-374a-p5
ENSSSCG00000000886	SLC25A3	hsa-miR-141-3p_R+1
ENSSSCG00000000893	AMDHD1	hsa-miR-141-3p_R+1
ENSSSCG00000000893	AMDHD1	ssc-miR-1839-3p_R+2
ENSSSCG00000000895	SNRPF	hsa-miR-27a-5p
ENSSSCG00000000902	NR2C1	hsa-miR-141-3p_R+1
ENSSSCG00000000902	NR2C1	ssc-miR-192
ENSSSCG00000000902	NR2C1	ssc-miR-215_R+1
ENSSSCG00000000905	NDUFA12	ssc-miR-194b-5p_1ss10GA
ENSSSCG00000000907	PLXNC1	PC-5p-9551_196
ENSSSCG00000000907	PLXNC1	hsa-miR-141-3p_R+1

ENSSSCG00000000907	PLXNC1	ssc-miR-194b-5p_1ss10GA
ENSSSCG00000000908	UBE2N	hsa-miR-149-3p_L+1
ENSSSCG00000000908	UBE2N	hsa-mir-1973-p5_1ss5CT
ENSSSCG00000000910	SOCS2	ssc-miR-194b-5p_1ss10GA
ENSSSCG00000000912	EEA1	hsa-miR-26a-1-3p
ENSSSCG00000000916	LUM	hsa-miR-26a-1-3p
ENSSSCG00000000916	LUM	ssc-miR-192
ENSSSCG00000000916	LUM	ssc-miR-215_R+1
ENSSSCG00000000921	ENSSSCG00000000921	hsa-miR-222-5p_L+2R-1
ENSSSCG00000000924	C12orf50	ssc-miR-1839-3p_R+2
ENSSSCG00000000925	C12orf29	PC-5p-9551_196
ENSSSCG00000000925	C12orf29	hsa-miR-200c-3p
ENSSSCG00000000925	C12orf29	ssc-miR-192
ENSSSCG00000000925	C12orf29	ssc-miR-215_R+1
ENSSSCG00000000926	CEP290	ssc-miR-190b
ENSSSCG00000000926	CEP290	ssc-miR-339_L-2
ENSSSCG00000000939	ACSS3	PC-5p-11132_163
ENSSSCG00000000948	SYT1	hsa-miR-27a-5p
ENSSSCG00000000948	SYT1	ssc-mir-374a-p5
ENSSSCG00000000950	E2F7	ssc-miR-1839-3p_R+2
ENSSSCG00000000950	E2F7	ssc-mir-374a-p5
ENSSSCG00000000951	CSRP2	mmu-miR-2137_L-2R-1_1ss16AG
ENSSSCG00000000952	ENSSSCG00000000952	hsa-miR-222-5p_L+2R-1
ENSSSCG00000000952	ENSSSCG00000000952	mmu-miR-2137_L-2R-1_1ss16AG
ENSSSCG00000000952	ENSSSCG00000000952	ssc-miR-139-3p
ENSSSCG00000000952	ENSSSCG00000000952	ssc-miR-194b-5p_1ss10GA
ENSSSCG00000024510	ENSSSCG00000024510	ssc-miR-190b
ENSSSCG00000000959	ENSSSCG00000000959	hsa-miR-149-3p_L+1
ENSSSCG00000000973	SELO	PC-5p-9551_196
ENSSSCG00000000975	PANX2	hsa-miR-200c-3p
ENSSSCG00000000978	MLC1	hsa-miR-27a-5p
ENSSSCG00000000978	MLC1	mmu-miR-2137_L-2R-1_1ss16AG
ENSSSCG00000000985	NR5A1	PC-5p-9551_196
ENSSSCG00000000994	GMDS	ssc-miR-192
ENSSSCG00000000994	GMDS	ssc-miR-215_R+1
ENSSSCG00000000997	PPP1R3G	mmu-miR-2137_L-2R-1_1ss16AG
ENSSSCG00000000998	RPP40	hsa-mir-1973-p5_1ss5CT
ENSSSCG00000000998	RPP40	ssc-miR-192
ENSSSCG00000000998	RPP40	ssc-miR-215_R+1
ENSSSCG00000000999	ENSSSCG00000000999	PC-5p-9551_196
ENSSSCG00000000999	ENSSSCG00000000999	hsa-mir-1973-p5_1ss5CT
ENSSSCG00000001000	ECI2	PC-5p-9551_196
ENSSSCG00000001009	RIPK1	hsa-miR-141-3p_R+1
ENSSSCG00000026211	ENSSSCG00000026211	ssc-miR-194b-5p_1ss10GA

ENSSSCG00000026211	ENSSSCG00000026211	ssc-miR-339_L-2
ENSSSCG00000025199	ENSSSCG00000025199	hsa-mir-1973-p5_1ss5CT
ENSSSCG00000024310	ENSSSCG00000024310	hsa-mir-1973-p5_1ss5CT
ENSSSCG00000026199	ENSSSCG00000026199	ssc-miR-194b-5p_1ss10GA
ENSSSCG00000026199	ENSSSCG00000026199	ssc-miR-339_L-2
ENSSSCG00000001021	RREB1	PC-5p-9551_196
ENSSSCG00000001021	RREB1	hsa-miR-149-3p_L+1
ENSSSCG00000001027	BMP6	hsa-miR-27a-5p
ENSSSCG00000001027	BMP6	ssc-miR-338_R+1
ENSSSCG00000001030	ENSSSCG00000001030	hsa-miR-141-3p_R+1
ENSSSCG00000001033	SLC35B3	ssc-miR-190b
ENSSSCG00000001036	TFAP2A	hsa-miR-141-3p_R+1
ENSSSCG00000001036	TFAP2A	hsa-miR-149-3p_L+1
ENSSSCG00000001036	TFAP2A	hsa-miR-200c-3p
ENSSSCG00000001039	GCNT2	PC-5p-9551_196
ENSSSCG00000001049	HIVEP1	hsa-miR-222-5p_L+2R-1
ENSSSCG00000001050	EDN1	ssc-miR-194b-5p_1ss10GA
ENSSSCG00000001053	TBC1D7	hsa-miR-141-3p_R+1
ENSSSCG00000001056	RANBP9	hsa-miR-141-3p_R+1
ENSSSCG00000001056	RANBP9	hsa-miR-200c-3p
ENSSSCG00000001056	RANBP9	hsa-miR-4508_L+2R-1
ENSSSCG00000001057	MCUR1	PC-5p-9551_196
ENSSSCG00000001057	MCUR1	hsa-miR-149-3p_L+1
ENSSSCG00000001057	MCUR1	hsa-miR-222-5p_L+2R-1
ENSSSCG00000001063	MYLIP	ssc-miR-139-3p
ENSSSCG00000001063	MYLIP	ssc-miR-338_R+1
ENSSSCG00000001066	RBM24	PC-5p-9551_196
ENSSSCG00000001066	RBM24	hsa-miR-141-3p_R+1
ENSSSCG00000001066	RBM24	ssc-miR-1839-3p_R+2
ENSSSCG00000001068	ENSSSCG00000001068	mmu-miR-1983_L-2R+1
ENSSSCG00000001073	TPMT	hsa-miR-149-3p_L+1
ENSSSCG00000001073	TPMT	hsa-miR-27a-5p
ENSSSCG00000001073	TPMT	ssc-miR-1839-3p_R+2
ENSSSCG00000001073	TPMT	ssc-miR-338_R+1
ENSSSCG00000001074	KDM1B	PC-5p-11132_163
ENSSSCG00000001074	KDM1B	hsa-miR-149-3p_L+1
ENSSSCG00000001074	KDM1B	hsa-mir-1973-p5_1ss5CT
ENSSSCG00000001074	KDM1B	ssc-mir-374a-p5
ENSSSCG00000001076	RNF144B	PC-5p-9551_196
ENSSSCG00000001076	RNF144B	mmu-miR-1983_L-2R+1
ENSSSCG00000001076	RNF144B	mmu-miR-2137_L-2R-1_1ss16AG
ENSSSCG00000001076	RNF144B	ssc-miR-194b-5p_1ss10GA
ENSSSCG00000001076	RNF144B	ssc-mir-374a-p5
ENSSSCG00000001079	E2F3	hsa-miR-141-3p_R+1

ENSSSCG00000001079	E2F3	hsa-miR-149-3p_L+1
ENSSSCG00000001079	E2F3	hsa-miR-200c-3p
ENSSSCG00000001079	E2F3	hsa-miR-222-5p_L+2R-1
ENSSSCG00000001079	E2F3	hsa-mir-1973-p5_1ss5CT
ENSSSCG00000001085	DCDC2	hsa-miR-222-5p_L+2R-1
ENSSSCG00000001087	MRS2	ssc-miR-194b-5p_1ss10GA
ENSSSCG00000001087	MRS2	ssc-miR-339_L-2
ENSSSCG00000001087	MRS2	ssc-mir-374a-p5
ENSSSCG00000001091	KIAA0319	hsa-miR-141-3p_R+1
ENSSSCG00000001091	KIAA0319	hsa-miR-200c-3p
ENSSSCG00000001091	KIAA0319	hsa-miR-26a-1-3p
ENSSSCG00000001092	TDP2	hsa-miR-200c-3p
ENSSSCG00000001094	C6orf62	ssc-miR-1839-3p_R+2
ENSSSCG00000001094	C6orf62	ssc-miR-192
ENSSSCG00000001094	C6orf62	ssc-miR-215_R+1
ENSSSCG00000001095	GMNN	ssc-miR-194b-5p_1ss10GA
ENSSSCG00000027958	ENSSSCG00000027958	PC-5p-11132_163
ENSSSCG00000027958	ENSSSCG00000027958	hsa-miR-200c-3p
ENSSSCG00000027958	ENSSSCG00000027958	ssc-miR-192
ENSSSCG00000027958	ENSSSCG00000027958	ssc-miR-215_R+1
ENSSSCG00000001156	ZNF322	PC-5p-9551_196
ENSSSCG00000022394	ENSSSCG00000022394	PC-5p-11132_163
ENSSSCG00000022394	ENSSSCG00000022394	hsa-miR-200c-3p
ENSSSCG00000022394	ENSSSCG00000022394	ssc-miR-192
ENSSSCG00000022394	ENSSSCG00000022394	ssc-miR-215_R+1
ENSSSCG00000001202	ZNF389	hsa-miR-141-3p_R+1
ENSSSCG00000001203	ZSCAN9	ssc-miR-338_R+1
ENSSSCG00000001204	ENSSSCG00000001204	mmu-miR-1983_L-2R+1
ENSSSCG00000001209	ZSCAN12	hsa-miR-26a-1-3p
ENSSSCG00000001209	ZSCAN12	ssc-miR-1839-3p_R+2
ENSSSCG00000001209	ZSCAN12	ssc-miR-192
ENSSSCG00000001209	ZSCAN12	ssc-miR-215_R+1
ENSSSCG00000001209	ZSCAN12	ssc-miR-338_R+1
ENSSSCG00000001214	GPX5	hsa-miR-26a-1-3p
ENSSSCG00000001219	TRIM27	ssc-miR-139-3p
ENSSSCG00000001219	TRIM27	ssc-miR-190b
ENSSSCG00000001233	TRIM26	hsa-miR-149-3p_L+1
ENSSSCG00000001233	TRIM26	hsa-mir-1973-p5_1ss5CT
ENSSSCG00000001235	TRIM15	hsa-miR-4508_L+2R-1
ENSSSCG00000001235	TRIM15	mmu-miR-2137_L-2R-1_1ss16AG
ENSSSCG00000028173	TRIM31	hsa-miR-149-3p_L+1
ENSSSCG00000024259	ENSSSCG00000024259	PC-5p-9551_196
ENSSSCG00000024259	ENSSSCG00000024259	mmu-miR-1983_L-2R+1
ENSSSCG00000028347	PPP1R11	PC-5p-9551_196

ENSSSCG00000028347	PPP1R11	mmu-miR-1983_L-2R+1
ENSSSCG00000001239	ENSSSCG00000001239	hsa-miR-141-3p_R+1
ENSSSCG00000022261	ENSSSCG00000022261	hsa-miR-149-3p_L+1
ENSSSCG00000001245	ZNRD1	hsa-miR-141-3p_R+1
ENSSSCG00000001252	UBD	ssc-miR-192
ENSSSCG00000001252	UBD	ssc-miR-215_R+1
ENSSSCG00000001346	ABCF1	ssc-miR-338_R+1
ENSSSCG00000001347	PPP1R10	hsa-miR-149-3p_L+1
ENSSSCG00000001362	MDC1	hsa-miR-141-3p_R+1
ENSSSCG00000001362	MDC1	hsa-miR-200c-3p
ENSSSCG00000001379	TUBB	hsa-miR-149-3p_L+1
ENSSSCG00000001379	TUBB	hsa-miR-200c-3p
ENSSSCG00000001379	TUBB	hsa-miR-27a-5p
ENSSSCG00000001384	VARS2	hsa-miR-149-3p_L+1
ENSSSCG00000001384	VARS2	ssc-miR-339_L-2
ENSSSCG00000001390	PSORS1C2	hsa-miR-222-5p_L+2R-1
ENSSSCG00000001394	MIC-2	ssc-miR-1839-3p_R+2
ENSSSCG00000001403	NR5A1	hsa-miR-149-3p_L+1
ENSSSCG00000001404	NR5A1	PC-5p-9551_196
ENSSSCG00000001404	NR5A1	sha-mir-24-1-p3_1ss2GC
ENSSSCG00000001410	BAG6	hsa-miR-200c-3p
ENSSSCG00000001410	BAG6	hsa-miR-200c-3p
ENSSSCG00000001412	C6orf47	mmu-miR-1983_L-2R+1
ENSSSCG00000001419	SLC44A4	mmu-miR-2137_L-2R-1_1ss16AG
ENSSSCG00000001420	EHMT2	hsa-miR-149-3p_L+1
ENSSSCG00000001420	EHMT2	hsa-miR-149-3p_L+1
ENSSSCG00000001422	C2	mmu-miR-2137_L-2R-1_1ss16AG
ENSSSCG00000001428	CYP21A2	hsa-miR-149-3p_L+1
ENSSSCG00000001433	NR5A1	hsa-miR-141-3p_R+1
ENSSSCG00000001435	AGPAT1	PC-5p-9551_196
ENSSSCG00000001435	AGPAT1	hsa-miR-4508_L+2R-1
ENSSSCG00000001435	AGPAT1	mmu-miR-2137_L-2R-1_1ss16AG
ENSSSCG00000001436	RNF5	hsa-miR-149-3p_L+1
ENSSSCG00000001439	GPSM3	hsa-miR-149-3p_L+1
ENSSSCG00000001441	BTNL5	hsa-miR-26a-1-3p
ENSSSCG00000001441	BTNL5	mmu-miR-1983_L-2R+1
ENSSSCG00000001459	ENSSSCG00000001459	hsa-miR-149-3p_L+1
ENSSSCG00000001459	ENSSSCG00000001459	ssc-miR-139-3p
ENSSSCG00000001465	SLA-DOB	ssc-miR-139-3p
ENSSSCG00000020893	NR5A1	hsa-miR-149-3p_L+1
ENSSSCG00000001471	BRD2	hsa-miR-149-3p_L+1
ENSSSCG00000001472	SLA-DOA	hsa-miR-141-3p_R+1
ENSSSCG00000001475	LOC100155600	hsa-miR-27a-5p
ENSSSCG00000001477	RING1	mmu-miR-2137_L-2R-1_1ss16AG

ENSSSCG00000001478	ENSSSCG00000001478	ssc-miR-1839-3p_R+2
ENSSSCG00000001478	ENSSSCG00000001478	ssc-miR-192
ENSSSCG00000001478	ENSSSCG00000001478	ssc-miR-215_R+1
ENSSSCG00000001479	HMGCLL1	hsa-miR-141-3p_R+1
ENSSSCG00000001479	HMGCLL1	ssc-miR-190b
ENSSSCG00000001479	HMGCLL1	ssc-miR-194b-5p_1ss10GA
ENSSSCG00000001483	FAM83B	hsa-miR-26a-1-3p
ENSSSCG00000001483	FAM83B	hsa-mir-1973-p5_1ss5CT
ENSSSCG00000001486	LRRC1	hsa-miR-141-3p_R+1
ENSSSCG00000001486	LRRC1	hsa-mir-1973-p5_1ss5CT
ENSSSCG00000001486	LRRC1	ssc-miR-139-3p
ENSSSCG00000001488	GCLC	ssc-miR-139-3p
ENSSSCG00000001493	ENSSSCG00000001493	hsa-miR-149-3p_L+1
ENSSSCG00000001498	BEND6	hsa-miR-149-3p_L+1
ENSSSCG00000001498	BEND6	ssc-miR-194b-5p_1ss10GA
ENSSSCG00000001503	B3GALT4	hsa-miR-149-3p_L+1
ENSSSCG00000001510	ENSSSCG00000001510	ssc-miR-1839-3p_R+2
ENSSSCG00000001516	BAK1	PC-5p-11132_163
ENSSSCG00000001516	BAK1	PC-5p-9551_196
ENSSSCG00000001516	BAK1	hsa-miR-149-3p_L+1
ENSSSCG00000001516	BAK1	ssc-miR-139-3p
ENSSSCG00000001516	BAK1	ssc-miR-192
ENSSSCG00000001516	BAK1	ssc-miR-215_R+1
ENSSSCG00000001518	ITPR3	hsa-miR-222-5p_L+2R-1
ENSSSCG00000001526	HMGA1	PC-5p-9551_196
ENSSSCG00000001539	PPARD	PC-5p-9551_196
ENSSSCG00000001554	SRPK1	hsa-miR-149-3p_L+1
ENSSSCG00000001556	MAPK14	mmu-miR-1983_L-2R+1
ENSSSCG00000001556	MAPK14	ssc-miR-339_L-2
ENSSSCG00000001562	KCTD20	ssc-miR-1839-3p_R+2
ENSSSCG00000001562	KCTD20	ssc-miR-194b-5p_1ss10GA
ENSSSCG00000001564	NR5A1	ssc-miR-215_R+1
ENSSSCG00000001569	C6orf89	ssc-miR-1839-3p_R+2
ENSSSCG00000001571	MTCH1	ssc-mir-374a-p5
ENSSSCG00000001573	PIM1	hsa-miR-149-3p_L+1
ENSSSCG00000001573	PIM1	ssc-miR-338_R+1
ENSSSCG00000001577	ENSSSCG00000001577	ssc-miR-192
ENSSSCG00000001577	ENSSSCG00000001577	ssc-miR-215_R+1
ENSSSCG00000001577	ENSSSCG00000001577	ssc-miR-339_L-2
ENSSSCG00000001579	ENSSSCG00000001579	hsa-miR-149-3p_L+1
ENSSSCG00000001579	ENSSSCG00000001579	hsa-miR-27a-5p
ENSSSCG00000001596	NR5A1	PC-5p-9551_196
ENSSSCG00000001597	NR5A1	PC-5p-9551_196
ENSSSCG00000001597	NR5A1	hsa-miR-149-3p_L+1

ENSSSCG00000001597	NR5A1	hsa-miR-222-5p_L+2R-1
ENSSSCG00000001597	NR5A1	ssc-miR-139-3p
ENSSSCG00000001600	ENSSSCG00000001600	hsa-miR-200c-3p
ENSSSCG00000001600	ENSSSCG00000001600	ssc-miR-139-3p
ENSSSCG00000001600	ENSSSCG00000001600	ssc-miR-339_L-2
ENSSSCG00000024348	NR5A1	hsa-miR-222-5p_L+2R-1
ENSSSCG00000024348	NR5A1	ssc-miR-338_R+1
ENSSSCG00000001611	NR5A1	hsa-miR-200c-3p
ENSSSCG00000001611	NR5A1	hsa-miR-27a-5p
ENSSSCG00000001611	NR5A1	mmu-miR-1983_L-2R+1
ENSSSCG00000001611	NR5A1	ssc-miR-139-3p
ENSSSCG00000001611	NR5A1	ssc-miR-339_L-2
ENSSSCG00000001611	NR5A1	ssc-mir-374a-p5
ENSSSCG00000001613	TREML1	ssc-mir-374a-p5
ENSSSCG00000001616	TREML-2	PC-5p-9551_196
ENSSSCG00000001615	ENSSSCG00000001615	hsa-mir-1973-p5_1ss5CT
ENSSSCG00000001617	TREM1	hsa-mir-1973-p5_1ss5CT
ENSSSCG00000001619	FOXP4	hsa-miR-149-3p_L+1
ENSSSCG00000001619	FOXP4	mmu-miR-2137_L-2R-1_1ss16AG
ENSSSCG00000001620	MDFI	hsa-miR-149-3p_L+1
ENSSSCG00000001620	MDFI	ssc-miR-339_L-2
ENSSSCG00000001621	TFEB	hsa-miR-149-3p_L+1
ENSSSCG00000001624	ENSSSCG00000001624	ssc-miR-139-3p
ENSSSCG00000001639	NR5A1	hsa-miR-200c-3p
ENSSSCG00000001639	NR5A1	ssc-miR-1839-3p_R+2
ENSSSCG00000001639	NR5A1	ssc-miR-192
ENSSSCG00000001639	NR5A1	ssc-miR-215_R+1
ENSSSCG00000001639	NR5A1	ssc-miR-339_L-2
ENSSSCG00000001646	GLTSCR1L	hsa-miR-27a-5p
ENSSSCG00000001646	GLTSCR1L	ssc-miR-192
ENSSSCG00000001646	GLTSCR1L	ssc-miR-215_R+1
ENSSSCG00000001654	PPP2R5D	hsa-mir-1973-p5_1ss5CT
ENSSSCG00000001655	MEA1	hsa-miR-149-3p_L+1
ENSSSCG00000001656	RRP36	ssc-miR-1839-3p_R+2
ENSSSCG00000001656	RRP36	ssc-mir-374a-p5
ENSSSCG00000001657	CUL7	hsa-mir-1973-p5_1ss5CT
ENSSSCG00000001660	PTK7	hsa-miR-149-3p_L+1
ENSSSCG00000001661	SRF	PC-5p-9551_196
ENSSSCG00000001661	SRF	hsa-miR-149-3p_L+1
ENSSSCG00000001661	SRF	hsa-miR-200c-3p
ENSSSCG00000026568	DNPH1	mmu-miR-2137_L-2R-1_1ss16AG
ENSSSCG00000001667	ZNF318	ssc-miR-1839-3p_R+2
ENSSSCG00000001667	ZNF318	ssc-miR-190b
ENSSSCG00000001667	ZNF318	ssc-miR-192

ENSSSCG00000001667	ZNF318	ssc-miR-215_R+1
ENSSSCG00000026568	DNPH1	mmu-miR-2137_L-2R-1_1ss16AG
ENSSSCG00000001683	POLH	hsa-miR-200c-3p
ENSSSCG00000001683	POLH	hsa-mir-1973-p5_1ss5CT
ENSSSCG00000001683	POLH	ssc-miR-194b-5p_1ss10GA
ENSSSCG00000001683	POLH	ssc-miR-338_R+1
ENSSSCG00000001686	NR5A1	hsa-miR-149-3p_L+1
ENSSSCG00000001686	NR5A1	ssc-miR-339_L-2
ENSSSCG00000001688	NR5A1	hsa-miR-141-3p_R+1
ENSSSCG00000001688	NR5A1	hsa-miR-149-3p_L+1
ENSSSCG00000001688	NR5A1	ssc-miR-194b-5p_1ss10GA
ENSSSCG00000001701	HSP90AB1	mmu-miR-2137_L-2R-1_1ss16AG
ENSSSCG00000001702	SLC35B2	hsa-miR-149-3p_L+1
ENSSSCG00000001702	SLC35B2	hsa-miR-200c-3p
ENSSSCG00000001703	NFKBIE	PC-5p-9551_196
ENSSSCG00000001703	NFKBIE	ssc-miR-338_R+1
ENSSSCG00000001703	NFKBIE	ssc-miR-339_L-2
ENSSSCG00000001706	AARS2	PC-5p-9551_196
ENSSSCG00000001706	AARS2	hsa-miR-222-5p_L+2R-1
ENSSSCG00000001708	CDC5L	ssc-miR-338_R+1
ENSSSCG00000001711	ENSSSCG00000001711	PC-5p-9551_196
ENSSSCG00000001711	ENSSSCG00000001711	hsa-miR-141-3p_R+1
ENSSSCG00000001715	ENPP5	PC-5p-9551_196
ENSSSCG00000001715	ENPP5	hsa-miR-141-3p_R+1
ENSSSCG00000001715	ENPP5	ssc-miR-1839-3p_R+2
ENSSSCG00000001718	ENSSSCG00000001718	ssc-miR-339_L-2
ENSSSCG00000001725	ADGRF5	hsa-mir-1973-p5_1ss5CT
ENSSSCG00000001731	CENPQ	hsa-mir-1973-p5_1ss5CT
ENSSSCG00000001731	CENPQ	ssc-miR-190b
ENSSSCG00000001731	CENPQ	ssc-miR-338_R+1
ENSSSCG00000001732	ENSSSCG00000001732	PC-5p-11132_163
ENSSSCG00000001732	ENSSSCG00000001732	ssc-mir-374a-p5
ENSSSCG00000001743	DEFB110	ssc-miR-194b-5p_1ss10GA
ENSSSCG00000001752	CHRNA3	PC-5p-11132_163
ENSSSCG00000001752	CHRNA3	PC-5p-9551_196
ENSSSCG00000001752	CHRNA3	hsa-miR-149-3p_L+1
ENSSSCG00000001752	CHRNA3	hsa-miR-200c-3p
ENSSSCG00000001752	CHRNA3	hsa-miR-222-5p_L+2R-1
ENSSSCG00000001759	DNAJA4	hsa-miR-141-3p_R+1
ENSSSCG00000001759	DNAJA4	hsa-miR-149-3p_L+1
ENSSSCG00000001759	DNAJA4	ssc-mir-374a-p5
ENSSSCG00000001760	ACSBG1	PC-5p-9551_196
ENSSSCG00000001760	ACSBG1	hsa-miR-141-3p_R+1
ENSSSCG00000001760	ACSBG1	mmu-miR-1983_L-2R+1

ENSSSCG00000001760	ACSBG1	ssc-miR-192
ENSSSCG00000001760	ACSBG1	ssc-miR-215_R+1
ENSSSCG00000001761	IDH3A	PC-5p-11132_163
ENSSSCG00000001761	IDH3A	hsa-miR-149-3p_L+1
ENSSSCG00000001761	IDH3A	hsa-miR-222-5p_L+2R-1
ENSSSCG00000001762	CIB2	PC-5p-9551_196
ENSSSCG00000001771	RASGRF1	hsa-miR-149-3p_L+1
ENSSSCG00000001771	RASGRF1	hsa-mir-1973-p5_1ss5CT
ENSSSCG00000001779	ZFAND6	hsa-miR-200c-3p
ENSSSCG00000001779	ZFAND6	hsa-miR-26a-1-3p
ENSSSCG00000001780	FAH	hsa-miR-200c-3p
ENSSSCG00000001788	NR5A1	PC-5p-9551_196
ENSSSCG00000001792	NR5A1	PC-5p-11132_163
ENSSSCG00000001792	NR5A1	PC-5p-9551_196
ENSSSCG00000001805	WHAMM	ssc-miR-338_R+1
ENSSSCG00000001814	ENSSSCG00000001814	hsa-miR-200c-3p
ENSSSCG00000001819	MAN2A2	PC-5p-9551_196
ENSSSCG00000001819	MAN2A2	hsa-miR-222-5p_L+2R-1
ENSSSCG00000001819	MAN2A2	hsa-mir-1973-p5_1ss5CT
ENSSSCG00000001819	MAN2A2	mmu-miR-1983_L-2R+1
ENSSSCG00000001819	MAN2A2	ssc-miR-1839-3p_R+2
ENSSSCG00000001820	HDDC3	hsa-miR-222-5p_L+2R-1
ENSSSCG00000001821	UNC45A	PC-5p-9551_196
ENSSSCG00000001821	UNC45A	ssc-miR-339_L-2
ENSSSCG00000001824	ZXDC	hsa-miR-222-5p_L+2R-1
ENSSSCG00000001834	MFGE8	PC-5p-9551_196
ENSSSCG00000001836	RLBP1	hsa-miR-149-3p_L+1
ENSSSCG00000001849	ANPEP	hsa-miR-149-3p_L+1
ENSSSCG00000001849	ANPEP	mmu-miR-2137_L-2R-1_1ss16AG
ENSSSCG00000001852	IDH2	hsa-miR-149-3p_L+1
ENSSSCG00000001859	GDPGP1	hsa-miR-222-5p_L+2R-1
ENSSSCG00000001859	GDPGP1	ssc-miR-1839-3p_R+2
ENSSSCG00000001863	ENSSSCG00000001863	PC-5p-9551_196
ENSSSCG00000001863	ENSSSCG00000001863	hsa-miR-149-3p_L+1
ENSSSCG00000001866	RCN2	hsa-miR-222-5p_L+2R-1
ENSSSCG00000001866	RCN2	ssc-mir-374a-p5
ENSSSCG00000001868	TSPAN3	ssc-miR-194b-5p_1ss10GA
ENSSSCG00000001871	ENSSSCG00000001871	hsa-miR-141-3p_R+1
ENSSSCG00000001871	ENSSSCG00000001871	hsa-miR-27a-5p
ENSSSCG00000001876	IMP3	ssc-miR-339_L-2
ENSSSCG00000001877	SNUPN	hsa-miR-141-3p_R+1
ENSSSCG00000001877	SNUPN	ssc-miR-338_R+1
ENSSSCG00000001877	SNUPN	ssc-miR-339_L-2
ENSSSCG00000001878	PTPN9	PC-5p-9551_196

ENSSSCG00000001878	PTPN9	hsa-miR-149-3p_L+1
ENSSSCG00000001878	PTPN9	hsa-miR-222-5p_L+2R-1
ENSSSCG00000001882	NEIL1	hsa-miR-27a-5p
ENSSSCG00000001885	C15orf39	hsa-miR-222-5p_L+2R-1
ENSSSCG00000001887	SCAMP5	PC-5p-9551_196
ENSSSCG00000001887	SCAMP5	mmu-miR-1983_L-2R+1
ENSSSCG00000001887	SCAMP5	ssc-miR-338_R+1
ENSSSCG00000001892	SCAMP2	ssc-miR-1839-3p_R+2
ENSSSCG00000001895	CPLX3	PC-5p-11132_163
ENSSSCG00000001895	CPLX3	hsa-miR-149-3p_L+1
ENSSSCG00000001898	ULK3	hsa-miR-149-3p_L+1
ENSSSCG00000001901	CYP1A2	hsa-miR-222-5p_L+2R-1
ENSSSCG00000001903	EDC3	PC-5p-9551_196
ENSSSCG00000001903	EDC3	hsa-miR-4508_L+2R-1
ENSSSCG00000001903	EDC3	ssc-miR-338_R+1
ENSSSCG00000001904	CLK3	ssc-miR-139-3p
ENSSSCG00000001906	CYP1A1	hsa-miR-4508_L+2R-1
ENSSSCG00000001906	CYP1A1	ssc-miR-339_L-2
ENSSSCG00000001909	ENSSSCG00000001909	PC-5p-9551_196
ENSSSCG00000001909	ENSSSCG00000001909	hsa-miR-149-3p_L+1
ENSSSCG00000001910	ISLR	PC-5p-11132_163
ENSSSCG00000001910	ISLR	mmu-miR-1983_L-2R+1
ENSSSCG00000001910	ISLR	ssc-miR-339_L-2
ENSSSCG00000001917	ENSSSCG00000001917	ssc-miR-338_R+1
ENSSSCG00000001918	NPTN	hsa-miR-141-3p_R+1
ENSSSCG00000001918	NPTN	ssc-miR-338_R+1
ENSSSCG00000001918	NPTN	ssc-miR-339_L-2
ENSSSCG00000001921	ENSSSCG00000001921	PC-5p-9551_196
ENSSSCG00000001921	ENSSSCG00000001921	hsa-miR-141-3p_R+1
ENSSSCG00000001921	ENSSSCG00000001921	hsa-miR-222-5p_L+2R-1
ENSSSCG00000001921	ENSSSCG00000001921	hsa-miR-26a-1-3p
ENSSSCG00000001921	ENSSSCG00000001921	ssc-miR-339_L-2
ENSSSCG00000001924	BBS4	hsa-mir-1973-p5_1ss5CT
ENSSSCG00000001927	HEXA	hsa-miR-149-3p_L+1
ENSSSCG00000001930	PKM	PC-5p-9551_196
ENSSSCG00000001930	PKM	hsa-miR-149-3p_L+1
ENSSSCG00000001931	ENSSSCG00000001931	mmu-miR-1983_L-2R+1
ENSSSCG00000001931	ENSSSCG00000001931	mmu-miR-1983_L-2R+1
ENSSSCG00000001934	SEC23A	PC-5p-11132_163
ENSSSCG00000001934	SEC23A	hsa-miR-200c-3p
ENSSSCG00000001934	SEC23A	hsa-mir-1973-p5_1ss5CT
ENSSSCG00000001934	SEC23A	ssc-mir-374a-p5
ENSSSCG00000001940	FOXA1	ssc-miR-194b-5p_1ss10GA
ENSSSCG00000001940	FOXA1	ssc-miR-338_R+1

ENSSSCG00000001942	SLC25A21	PC-5p-9551_196
ENSSSCG00000001942	SLC25A21	hsa-miR-27a-5p
ENSSSCG00000001942	SLC25A21	ssc-miR-1839-3p_R+2
ENSSSCG00000001942	SLC25A21	ssc-miR-192
ENSSSCG00000001942	SLC25A21	ssc-miR-215_R+1
ENSSSCG00000001942	SLC25A21	ssc-miR-338_R+1
ENSSSCG00000001942	SLC25A21	ssc-miR-339_L-2
ENSSSCG00000001942	SLC25A21	ssc-mir-374a-p5
ENSSSCG00000001943	PAX9	ssc-miR-192
ENSSSCG00000001943	PAX9	ssc-miR-215_R+1
ENSSSCG00000001943	PAX9	ssc-miR-339_L-2
ENSSSCG00000001945	NKX2-1	ssc-miR-339_L-2
ENSSSCG00000001946	MBIP	hsa-miR-200c-3p
ENSSSCG00000001947	BRMS1L	hsa-miR-141-3p_R+1
ENSSSCG00000001947	BRMS1L	ssc-mir-374a-p5
ENSSSCG00000001959	CFL2	hsa-miR-141-3p_R+1
ENSSSCG00000001959	CFL2	hsa-miR-149-3p_L+1
ENSSSCG00000001959	CFL2	hsa-miR-200c-3p
ENSSSCG00000001959	CFL2	hsa-miR-222-5p_L+2R-1
ENSSSCG00000001959	CFL2	ssc-miR-194b-5p_1ss10GA
ENSSSCG00000001960	EAPP	hsa-miR-141-3p_R+1
ENSSSCG00000001963	EGLN3	PC-5p-9551_196
ENSSSCG00000001963	EGLN3	ssc-mir-374a-p5
ENSSSCG00000001970	HEATR5A	hsa-miR-200c-3p
ENSSSCG00000001970	HEATR5A	hsa-miR-26a-1-3p
ENSSSCG00000001974	G2E3	hsa-miR-200c-3p
ENSSSCG00000001974	G2E3	ssc-miR-194b-5p_1ss10GA
ENSSSCG00000001984	KHNYN	ssc-miR-339_L-2
ENSSSCG00000001985	CBLN3	PC-5p-9551_196
ENSSSCG00000001985	CBLN3	hsa-miR-222-5p_L+2R-1
ENSSSCG00000001985	CBLN3	ssc-miR-139-3p
ENSSSCG00000001989	CIDEB	PC-5p-11132_163
ENSSSCG00000001989	CIDEB	hsa-miR-222-5p_L+2R-1
ENSSSCG00000001990	NOP9	hsa-miR-141-3p_R+1
ENSSSCG00000001990	NOP9	hsa-miR-222-5p_L+2R-1
ENSSSCG00000002001	REC8	ssc-miR-139-3p
ENSSSCG00000002005	EMC9	hsa-miR-4508_L+2R-1
ENSSSCG00000002008	DCAF11	PC-5p-9551_196
ENSSSCG00000002008	DCAF11	hsa-miR-149-3p_L+1
ENSSSCG00000002008	DCAF11	hsa-miR-222-5p_L+2R-1
ENSSSCG00000002008	DCAF11	hsa-miR-26a-1-3p
ENSSSCG00000002008	DCAF11	ssc-miR-1839-3p_R+2
ENSSSCG00000002014	JPH4	hsa-miR-149-3p_L+1
ENSSSCG00000002016	THTPA	hsa-miR-149-3p_L+1

ENSSSCG00000002025	SLC22A17	hsa-miR-149-3p_L+1
ENSSSCG00000002026	EFS	hsa-miR-149-3p_L+1
ENSSSCG00000002029	MYH7	hsa-mir-1973-p5_1ss5CT
ENSSSCG00000002039	NR5A1	hsa-miR-141-3p_R+1
ENSSSCG00000002039	NR5A1	hsa-miR-149-3p_L+1
ENSSSCG00000002040	NR5A1	PC-5p-9551_196
ENSSSCG00000002041	NR5A1	hsa-miR-27a-5p
ENSSSCG00000002041	NR5A1	hsa-mir-1973-p5_1ss5CT
ENSSSCG00000002041	NR5A1	mmu-miR-1983_L-2R+1
ENSSSCG00000002041	NR5A1	ssc-miR-338_R+1
ENSSSCG00000002041	NR5A1	ssc-miR-339_L-2
ENSSSCG00000002041	NR5A1	ssc-mir-374a-p5
ENSSSCG00000002050	DAD1	hsa-mir-1973-p5_1ss5CT
ENSSSCG00000002051	ABHD4	hsa-miR-26a-1-3p
ENSSSCG00000002051	ABHD4	hsa-mir-1973-p5_1ss5CT
ENSSSCG00000002051	ABHD4	mmu-miR-2137_L-2R-1_1ss16AG
ENSSSCG00000002051	ABHD4	ssc-miR-338_R+1
ENSSSCG00000002081	ENSSSCG00000002081	hsa-miR-222-5p_L+2R-1
ENSSSCG00000002081	ENSSSCG00000002081	ssc-miR-338_R+1
ENSSSCG00000002131	ENSSSCG00000002131	ssc-miR-338_R+1
ENSSSCG00000002136	TMEM55B	PC-5p-9551_196
ENSSSCG00000002136	TMEM55B	mmu-miR-2137_L-2R-1_1ss16AG
ENSSSCG00000002136	TMEM55B	ssc-miR-194b-5p_1ss10GA
ENSSSCG00000002142	ENSSSCG00000002142	ssc-miR-338_R+1
ENSSSCG00000002145	TTC5	hsa-miR-141-3p_R+1
ENSSSCG00000002145	TTC5	sha-mir-24-1-p3_1ss2GC
ENSSSCG00000002252	ARRDC4	PC-5p-11132_163
ENSSSCG00000002252	ARRDC4	hsa-miR-200c-3p
ENSSSCG00000002259	ENSSSCG00000002259	hsa-miR-222-5p_L+2R-1
ENSSSCG00000002262	SV2B	hsa-miR-149-3p_L+1
ENSSSCG00000002262	SV2B	ssc-miR-194b-5p_1ss10GA
ENSSSCG00000002262	SV2B	ssc-miR-338_R+1
ENSSSCG00000002265	FAM174B	PC-5p-9551_196
ENSSSCG00000002265	FAM174B	hsa-miR-149-3p_L+1
ENSSSCG00000002265	FAM174B	ssc-miR-139-3p
ENSSSCG00000002265	FAM174B	ssc-miR-338_R+1
ENSSSCG00000002267	RGMA	PC-5p-11132_163
ENSSSCG00000002277	SPTB	PC-5p-9551_196
ENSSSCG00000002277	SPTB	hsa-miR-149-3p_L+1
ENSSSCG00000002279	GPX2	hsa-miR-222-5p_L+2R-1
ENSSSCG00000002283	FUT8	PC-5p-11132_163
ENSSSCG00000002285	GPHN	hsa-miR-141-3p_R+1
ENSSSCG00000002285	GPHN	ssc-miR-190b
ENSSSCG00000002288	ATP6V1D	PC-5p-9551_196

ENSSSCG00000002288	ATP6V1D	hsa-miR-141-3p_R+1
ENSSSCG00000002288	ATP6V1D	hsa-miR-27a-5p
ENSSSCG00000002288	ATP6V1D	ssc-miR-190b
ENSSSCG00000002288	ATP6V1D	ssc-miR-194b-5p_1ss10GA
ENSSSCG00000002290	PLEK2	hsa-miR-149-3p_L+1
ENSSSCG00000002290	PLEK2	hsa-miR-222-5p_L+2R-1
ENSSSCG00000002290	PLEK2	hsa-mir-1973-p5_1ss5CT
ENSSSCG00000002290	PLEK2	ssc-miR-215_R+1
ENSSSCG00000002290	PLEK2	ssc-miR-338_R+1
ENSSSCG00000002290	PLEK2	ssc-miR-339_L-2
ENSSSCG00000002292	PLEKHH1	PC-5p-11132_163
ENSSSCG00000002292	PLEKHH1	hsa-miR-222-5p_L+2R-1
ENSSSCG00000002292	PLEKHH1	ssc-miR-192
ENSSSCG00000002292	PLEKHH1	ssc-miR-215_R+1
ENSSSCG00000002292	PLEKHH1	ssc-miR-338_R+1
ENSSSCG00000002296	RDH11	hsa-miR-200c-3p
ENSSSCG00000002296	RDH11	hsa-miR-27a-5p
ENSSSCG00000002297	RDH12	hsa-miR-141-3p_R+1
ENSSSCG00000002297	RDH12	hsa-miR-26a-1-3p
ENSSSCG00000002297	RDH12	hsa-miR-27a-5p
ENSSSCG00000002297	RDH12	ssc-miR-1839-3p_R+2
ENSSSCG00000002298	ZFYVE26	PC-5p-11132_163
ENSSSCG00000002298	ZFYVE26	ssc-miR-139-3p
ENSSSCG00000002298	ZFYVE26	ssc-miR-192
ENSSSCG00000002298	ZFYVE26	ssc-miR-215_R+1
ENSSSCG00000002298	ZFYVE26	ssc-miR-339_L-2
ENSSSCG00000002305	EXD2	hsa-miR-149-3p_L+1
ENSSSCG00000002306	GALNT16	hsa-miR-149-3p_L+1
ENSSSCG00000002306	GALNT16	hsa-miR-27a-5p
ENSSSCG00000002306	GALNT16	ssc-miR-339_L-2
ENSSSCG00000002309	PLEKHD1	ssc-miR-339_L-2
ENSSSCG00000002311	SUSD6	PC-5p-9551_196
ENSSSCG00000002311	SUSD6	hsa-miR-149-3p_L+1
ENSSSCG00000002311	SUSD6	hsa-miR-222-5p_L+2R-1
ENSSSCG00000002311	SUSD6	hsa-miR-4508_L+2R-1
ENSSSCG00000002311	SUSD6	ssc-miR-339_L-2
ENSSSCG00000002316	COX16	ssc-mir-374a-p5
ENSSSCG00000002330	PCNX1	hsa-miR-200c-3p
ENSSSCG00000002332	SIPA1L1	PC-5p-9551_196
ENSSSCG00000002332	SIPA1L1	hsa-miR-149-3p_L+1
ENSSSCG00000002340	PSEN1	mmu-miR-1983_L-2R+1
ENSSSCG00000002345	ENSSSCG00000002345	hsa-miR-149-3p_L+1
ENSSSCG00000002345	ENSSSCG00000002345	hsa-miR-27a-5p
ENSSSCG00000002345	ENSSSCG00000002345	ssc-miR-190b

ENSSSCG00000002345	ENSSSCG00000002345	ssc-miR-194b-5p_1ss10GA
ENSSSCG00000002348	ENSSSCG00000002348	hsa-miR-149-3p_L+1
ENSSSCG00000002349	ACOT4	ssc-miR-190b
ENSSSCG00000002352	ZNF410	PC-5p-9551_196
ENSSSCG00000002352	ZNF410	hsa-miR-222-5p_L+2R-1
ENSSSCG00000002352	ZNF410	hsa-mir-1973-p5_1ss5CT
ENSSSCG00000002352	ZNF410	ssc-miR-338_R+1
ENSSSCG00000002352	ZNF410	ssc-mir-374a-p5
ENSSSCG00000002353	FAM161B	hsa-miR-141-3p_R+1
ENSSSCG00000002353	FAM161B	hsa-miR-149-3p_L+1
ENSSSCG00000002354	COQ6	ssc-miR-1839-3p_R+2
ENSSSCG00000002354	COQ6	ssc-miR-338_R+1
ENSSSCG00000002370	AREL1	hsa-miR-149-3p_L+1
ENSSSCG00000002370	AREL1	mmu-miR-1983_L-2R+1
ENSSSCG00000002370	AREL1	mmu-miR-2137_L-2R-1_1ss16AG
ENSSSCG00000002370	AREL1	ssc-miR-338_R+1
ENSSSCG00000002370	AREL1	ssc-miR-339_L-2
ENSSSCG00000002374	DLST	PC-5p-9551_196
ENSSSCG00000002375	RPS6KL1	hsa-miR-149-3p_L+1
ENSSSCG00000002379	ACYP1	hsa-miR-200c-3p
ENSSSCG00000002381	NEK9	hsa-miR-149-3p_L+1
ENSSSCG00000002381	NEK9	ssc-miR-139-3p
ENSSSCG00000002383	FOS	ssc-miR-338_R+1
ENSSSCG00000002385	TGFB3	mmu-miR-1983_L-2R+1
ENSSSCG00000002389	VASH1	PC-5p-9551_196
ENSSSCG00000002389	VASH1	hsa-miR-149-3p_L+1
ENSSSCG00000002396	GSTZ1	mmu-miR-1983_L-2R+1
ENSSSCG00000002403	VIPAS39	hsa-miR-222-5p_L+2R-1
ENSSSCG00000002404	SPTLC2	PC-5p-9551_196
ENSSSCG00000002404	SPTLC2	hsa-miR-26a-1-3p
ENSSSCG00000002404	SPTLC2	hsa-miR-27a-5p
ENSSSCG00000002411	GTF2A1	hsa-miR-26a-1-3p
ENSSSCG00000002427	EML5	hsa-miR-141-3p_R+1
ENSSSCG00000002427	EML5	hsa-miR-27a-5p
ENSSSCG00000002431	ENSSSCG00000002431	hsa-miR-26a-1-3p
ENSSSCG00000002436	ENSSSCG00000002436	ssc-miR-139-3p
ENSSSCG00000002436	ENSSSCG00000002436	ssc-miR-194b-5p_1ss10GA
ENSSSCG00000002441	PPP4R3A	hsa-mir-1973-p5_1ss5CT
ENSSSCG00000002444	FBLN5	PC-5p-9551_196
ENSSSCG00000002446	ENSSSCG00000002446	hsa-miR-149-3p_L+1
ENSSSCG00000002446	ENSSSCG00000002446	hsa-miR-27a-5p
ENSSSCG00000002448	ENSSSCG00000002448	ssc-miR-194b-5p_1ss10GA
ENSSSCG00000002448	ENSSSCG00000002448	ssc-mir-374a-p5
ENSSSCG00000002451	ENSSSCG00000002451	hsa-miR-222-5p_L+2R-1

ENSSSCG00000002451	ENSSSCG00000002451	mmu-miR-2137_L-2R-1_1ss16AG
ENSSSCG00000002452	ENSSSCG00000002452	PC-5p-9551_196
ENSSSCG00000002452	ENSSSCG00000002452	ssc-miR-139-3p
ENSSSCG00000002452	ENSSSCG00000002452	ssc-miR-192
ENSSSCG00000002452	ENSSSCG00000002452	ssc-miR-215_R+1
ENSSSCG00000002455	GOLGA5	ssc-miR-338_R+1
ENSSSCG00000002457	ITPK1	mmu-miR-2137_L-2R-1_1ss16AG
ENSSSCG00000002457	ITPK1	ssc-miR-139-3p
ENSSSCG00000002460	ENSSSCG00000002460	hsa-miR-141-3p_R+1
ENSSSCG00000002460	ENSSSCG00000002460	mmu-miR-1983_L-2R+1
ENSSSCG00000002460	ENSSSCG00000002460	ssc-miR-339_L-2
ENSSSCG00000002461	BTBD7	hsa-miR-222-5p_L+2R-1
ENSSSCG00000002461	BTBD7	ssc-miR-194b-5p_1ss10GA
ENSSSCG00000002461	BTBD7	ssc-mir-374a-p5
ENSSSCG00000002469	OTUB2	hsa-miR-149-3p_L+1
ENSSSCG00000002470	ENSSSCG00000002470	hsa-miR-141-3p_R+1
ENSSSCG00000002474	PPP4R4	hsa-miR-141-3p_R+1
ENSSSCG00000002474	PPP4R4	ssc-mir-374a-p5
ENSSSCG00000002475	SERPINA6	ssc-mir-374a-p5
ENSSSCG00000002476	SERPINA1	hsa-miR-149-3p_L+1
ENSSSCG00000002481	SERPINA5	hsa-miR-222-5p_L+2R-1
ENSSSCG00000002494	CLMN	ssc-miR-139-3p
ENSSSCG00000002494	CLMN	ssc-miR-1839-3p_R+2
ENSSSCG00000002496	GLRX5	PC-5p-9551_196
ENSSSCG00000002496	GLRX5	ssc-miR-1839-3p_R+2
ENSSSCG00000002497	TCL1B	mmu-miR-1983_L-2R+1
ENSSSCG00000002497	TCL1B	ssc-miR-338_R+1
ENSSSCG00000002509	CCNK	PC-5p-9551_196
ENSSSCG00000002509	CCNK	hsa-miR-149-3p_L+1
ENSSSCG00000002509	CCNK	hsa-miR-222-5p_L+2R-1
ENSSSCG00000002509	CCNK	ssc-miR-194b-5p_1ss10GA
ENSSSCG00000002520	SLC25A29	mmu-miR-2137_L-2R-1_1ss16AG
ENSSSCG00000002523	CDC42BPB	hsa-miR-4508_L+2R-1
ENSSSCG00000002525	TRAF3	ssc-miR-194b-5p_1ss10GA
ENSSSCG00000002533	ENSSSCG00000002533	mmu-miR-1983_L-2R+1
ENSSSCG00000002544	PPP1R13B	PC-5p-9551_196
ENSSSCG00000002544	PPP1R13B	hsa-miR-27a-5p
ENSSSCG00000002544	PPP1R13B	hsa-miR-4508_L+2R-1
ENSSSCG00000002544	PPP1R13B	ssc-miR-139-3p
ENSSSCG00000002550	C14orf2	hsa-mir-1973-p5_1ss5CT
ENSSSCG00000002554	PLD4	PC-5p-9551_196
ENSSSCG00000002628	ICK	ssc-miR-339_L-2
ENSSSCG00000002633	NR5A1	hsa-miR-149-3p_L+1
ENSSSCG00000002637	NR5A1	PC-5p-9551_196

ENSSSCG00000002648	CBFA2T3	ssc-miR-339_L-2
ENSSSCG00000002655	MAP1LC3B	PC-5p-11132_163
ENSSSCG00000002655	MAP1LC3B	mmu-miR-1983_L-2R+1
ENSSSCG00000002655	MAP1LC3B	ssc-miR-192
ENSSSCG00000002655	MAP1LC3B	ssc-miR-215_R+1
ENSSSCG00000002655	MAP1LC3B	ssc-miR-339_L-2
ENSSSCG00000002667	ZDHHC7	hsa-miR-222-5p_L+2R-1
ENSSSCG00000002667	ZDHHC7	mmu-miR-1983_L-2R+1
ENSSSCG00000002671	ATP2C2	hsa-miR-149-3p_L+1
ENSSSCG00000002681	HSDL1	ssc-miR-194b-5p_1ss10GA
ENSSSCG00000002681	HSDL1	ssc-miR-339_L-2
ENSSSCG00000002682	MBTPS1	PC-5p-9551_196
ENSSSCG00000002684	ENSSSCG00000002684	hsa-miR-200c-3p
ENSSSCG00000002690	GAN	PC-5p-11132_163
ENSSSCG00000002690	GAN	hsa-miR-141-3p_R+1
ENSSSCG00000002690	GAN	hsa-miR-200c-3p
ENSSSCG00000002690	GAN	ssc-miR-190b
ENSSSCG00000002696	VAT1L	hsa-miR-149-3p_L+1
ENSSSCG00000002696	VAT1L	hsa-miR-200c-3p
ENSSSCG00000002696	VAT1L	hsa-miR-26a-1-3p
ENSSSCG00000002697	ENSSSCG00000002697	hsa-miR-141-3p_R+1
ENSSSCG00000002706	ADAT1	ssc-miR-338_R+1
ENSSSCG00000002706	ADAT1	ssc-mir-374a-p5
ENSSSCG00000002707	GABARAPL2	PC-5p-9551_196
ENSSSCG00000002707	GABARAPL2	hsa-miR-222-5p_L+2R-1
ENSSSCG00000002708	NR5A1	hsa-miR-149-3p_L+1
ENSSSCG00000002708	NR5A1	ssc-miR-192
ENSSSCG00000002708	NR5A1	ssc-miR-215_R+1
ENSSSCG00000002712	NR5A1	mmu-miR-1983_L-2R+1
ENSSSCG00000002714	ENSSSCG00000002714	ssc-miR-339_L-2
ENSSSCG00000002715	RFWD3	mmu-miR-1983_L-2R+1
ENSSSCG00000002715	RFWD3	ssc-miR-338_R+1
ENSSSCG00000002718	FA2H	hsa-miR-149-3p_L+1
ENSSSCG00000002721	ENSSSCG00000002721	hsa-miR-149-3p_L+1
ENSSSCG00000002721	ENSSSCG00000002721	ssc-miR-139-3p
ENSSSCG00000002721	ENSSSCG00000002721	ssc-miR-339_L-2
ENSSSCG00000002725	ENSSSCG00000002725	hsa-miR-141-3p_R+1
ENSSSCG00000002734	MARVELD3	hsa-miR-141-3p_R+1
ENSSSCG00000002734	MARVELD3	hsa-miR-149-3p_L+1
ENSSSCG00000002734	MARVELD3	ssc-miR-339_L-2
ENSSSCG00000002736	TAT	hsa-miR-27a-5p
ENSSSCG00000002740	AP1G1	hsa-miR-27a-5p
ENSSSCG00000002743	ENSSSCG00000002743	hsa-miR-149-3p_L+1
ENSSSCG00000002754	NQO1	hsa-miR-149-3p_L+1

ENSSSCG00000002754	NQO1	hsa-miR-200c-3p
ENSSSCG00000002760	NIP7	hsa-miR-149-3p_L+1
ENSSSCG00000002760	NIP7	hsa-miR-26a-1-3p
ENSSSCG00000002760	NIP7	hsa-mir-1973-p5_1ss5CT
ENSSSCG00000002762	TERF2	mmu-miR-1983_L-2R+1
ENSSSCG00000002768	CENPT	mmu-miR-1983_L-2R+1
ENSSSCG00000002776	ATP6V0D1	hsa-miR-149-3p_L+1
ENSSSCG00000002776	ATP6V0D1	mmu-miR-1983_L-2R+1
ENSSSCG00000002780	TPPP3	hsa-miR-4508_L+2R-1
ENSSSCG00000002795	NR5A1	hsa-miR-27a-5p
ENSSSCG00000002795	NR5A1	ssc-miR-192
ENSSSCG00000002795	NR5A1	ssc-miR-215_R+1
ENSSSCG00000002797	ENSSSCG00000002797	hsa-miR-200c-3p
ENSSSCG00000002799	CNOT1	hsa-miR-26a-1-3p
ENSSSCG00000002799	CNOT1	mmu-miR-1983_L-2R+1
ENSSSCG00000002799	CNOT1	ssc-miR-339_L-2
ENSSSCG00000002800	ENSSSCG00000002800	PC-5p-9551_196
ENSSSCG00000002800	ENSSSCG00000002800	hsa-miR-222-5p_L+2R-1
ENSSSCG00000002802	GINS3	PC-5p-11132_163
ENSSSCG00000002802	GINS3	PC-5p-9551_196
ENSSSCG00000002802	GINS3	ssc-miR-139-3p
ENSSSCG00000002802	GINS3	ssc-miR-338_R+1
ENSSSCG00000002804	CSNK2A2	PC-5p-9551_196
ENSSSCG00000002804	CSNK2A2	hsa-miR-149-3p_L+1
ENSSSCG00000002804	CSNK2A2	hsa-miR-222-5p_L+2R-1
ENSSSCG00000002805	CFAP20	hsa-miR-26a-1-3p
ENSSSCG00000002805	CFAP20	ssc-miR-338_R+1
ENSSSCG00000002806	MMP15	hsa-miR-149-3p_L+1
ENSSSCG00000002806	MMP15	ssc-miR-139-3p
ENSSSCG00000002813	KATNB1	hsa-miR-222-5p_L+2R-1
ENSSSCG00000002814	ADGRG3	mmu-miR-1983_L-2R+1
ENSSSCG00000002818	PLLP	hsa-miR-149-3p_L+1
ENSSSCG00000002818	PLLP	hsa-mir-1973-p5_1ss5CT
ENSSSCG00000002819	ARL2BP	ssc-miR-139-3p
ENSSSCG00000002820	RSPRY1	hsa-miR-200c-3p
ENSSSCG00000002820	RSPRY1	mmu-miR-1983_L-2R+1
ENSSSCG00000002820	RSPRY1	ssc-miR-339_L-2
ENSSSCG00000002821	CCL22	hsa-miR-149-3p_L+1
ENSSSCG00000002821	CCL22	ssc-miR-194b-5p_1ss10GA
ENSSSCG00000002821	CCL22	ssc-mir-374a-p5
ENSSSCG00000002822	ENSSSCG00000002822	PC-5p-9551_196
ENSSSCG00000002822	ENSSSCG00000002822	hsa-miR-200c-3p
ENSSSCG00000002822	ENSSSCG00000002822	ssc-miR-194b-5p_1ss10GA
ENSSSCG00000002822	ENSSSCG00000002822	ssc-miR-338_R+1

ENSSSCG00000002829	MMP2	hsa-miR-149-3p_L+1
ENSSSCG00000002830	IRX6	hsa-miR-222-5p_L+2R-1
ENSSSCG00000002830	IRX6	hsa-miR-27a-5p
ENSSSCG00000002841	N4BP1	PC-5p-11132_163
ENSSSCG00000002841	N4BP1	hsa-miR-149-3p_L+1
ENSSSCG00000002841	N4BP1	ssc-miR-338_R+1
ENSSSCG00000002844	PHKB	hsa-miR-141-3p_R+1
ENSSSCG00000002844	PHKB	ssc-miR-338_R+1
ENSSSCG00000002846	DNAJA2	hsa-mir-1973-p5_1ss5CT
ENSSSCG00000002854	POP4	PC-5p-11132_163
ENSSSCG00000002854	POP4	ssc-miR-1839-3p_R+2
ENSSSCG00000002854	POP4	ssc-mir-374a-p5
ENSSSCG00000002859	ANKRD27	hsa-miR-149-3p_L+1
ENSSSCG00000002859	ANKRD27	hsa-miR-222-5p_L+2R-1
ENSSSCG00000002860	PDCD5	ssc-miR-339_L-2
ENSSSCG00000002861	ENSSSCG00000002861	PC-5p-9551_196
ENSSSCG00000002861	ENSSSCG00000002861	hsa-miR-222-5p_L+2R-1
ENSSSCG00000002863	LRP3	PC-5p-9551_196
ENSSSCG00000002863	LRP3	hsa-miR-4508_L+2R-1
ENSSSCG00000002864	SLC7A10	hsa-miR-4508_L+2R-1
ENSSSCG00000002872	KIAA0355	hsa-miR-222-5p_L+2R-1
ENSSSCG00000002872	KIAA0355	hsa-miR-26a-1-3p
ENSSSCG00000002872	KIAA0355	ssc-miR-190b
ENSSSCG00000002872	KIAA0355	ssc-miR-338_R+1
ENSSSCG00000002879	GRAMD1A	PC-5p-9551_196
ENSSSCG00000002879	GRAMD1A	hsa-miR-149-3p_L+1
ENSSSCG00000002880	ZNF792	PC-5p-9551_196
ENSSSCG00000002880	ZNF792	hsa-miR-222-5p_L+2R-1
ENSSSCG00000002880	ZNF792	ssc-miR-338_R+1
ENSSSCG00000002885	USF2	hsa-miR-27a-5p
ENSSSCG00000002885	USF2	ssc-miR-339_L-2
ENSSSCG00000002893	ENSSSCG00000002893	PC-5p-9551_196
ENSSSCG00000002893	ENSSSCG00000002893	hsa-miR-4508_L+2R-1
ENSSSCG00000002893	ENSSSCG00000002893	ssc-miR-338_R+1
ENSSSCG00000002895	KMT2B	hsa-miR-4508_L+2R-1
ENSSSCG00000002898	ENSSSCG00000002898	mmu-miR-2137_L-2R-1_1ss16AG
ENSSSCG00000002904	HAUS5	hsa-miR-222-5p_L+2R-1
ENSSSCG00000002904	HAUS5	ssc-miR-339_L-2
ENSSSCG00000002905	RBM42	hsa-miR-222-5p_L+2R-1
ENSSSCG00000002917	NFKBID	mmu-miR-1983_L-2R+1
ENSSSCG00000002921	CLIP3	PC-5p-9551_196
ENSSSCG00000002921	CLIP3	hsa-miR-26a-1-3p
ENSSSCG00000002921	CLIP3	ssc-miR-139-3p
ENSSSCG00000002921	CLIP3	ssc-miR-339_L-2

ENSSSCG00000002932	ZNF567	hsa-mir-1973-p5_1ss5CT
ENSSSCG00000002950	ENSSSCG00000002950	hsa-miR-149-3p_L+1
ENSSSCG00000002952	YIF1B	ssc-miR-194b-5p_1ss10GA
ENSSSCG00000002999	C19orf54	ssc-miR-339_L-2
ENSSSCG00000003000	ITPKC	PC-5p-9551_196
ENSSSCG00000003000	ITPKC	hsa-miR-222-5p_L+2R-1
ENSSSCG00000003000	ITPKC	ssc-miR-339_L-2
ENSSSCG00000003006	CYP2B6	hsa-miR-149-3p_L+1
ENSSSCG00000003006	CYP2B6	hsa-miR-4508_L+2R-1
ENSSSCG00000003006	CYP2B6	mmu-miR-1983_L-2R+1
ENSSSCG00000003006	CYP2B6	mmu-miR-2137_L-2R-1_1ss16AG
ENSSSCG00000003006	CYP2B6	ssc-miR-139-3p
ENSSSCG00000003006	CYP2B6	ssc-miR-339_L-2
ENSSSCG00000003007	ENSSSCG00000003007	ssc-miR-194b-5p_1ss10GA
ENSSSCG00000003015	ENSSSCG00000003015	mmu-miR-2137_L-2R-1_1ss16AG
ENSSSCG00000003015	ENSSSCG00000003015	ssc-miR-194b-5p_1ss10GA
ENSSSCG00000003017	TGFB1	hsa-miR-222-5p_L+2R-1
ENSSSCG00000003021	MEGF8	PC-5p-9551_196
ENSSSCG00000003042	ENSSSCG00000003042	ssc-miR-1839-3p_R+2
ENSSSCG00000003046	RABAC1	PC-5p-9551_196
ENSSSCG00000003046	RABAC1	ssc-miR-194b-5p_1ss10GA
ENSSSCG00000003054	ZNF575	mmu-miR-1983_L-2R+1
ENSSSCG00000003061	CADM4	PC-5p-9551_196
ENSSSCG00000003062	LYPD3	PC-5p-9551_196
ENSSSCG00000003062	LYPD3	ssc-miR-339_L-2
ENSSSCG00000003063	PHLDB3	hsa-miR-149-3p_L+1
ENSSSCG00000003091	IRF2BP1	hsa-miR-149-3p_L+1
ENSSSCG00000003093	SYMPK	hsa-miR-149-3p_L+1
ENSSSCG00000003106	AP2S1	hsa-miR-149-3p_L+1
ENSSSCG00000003107	ARHGAP35	hsa-mir-1973-p5_1ss5CT
ENSSSCG00000003110	ENSSSCG00000003110	ssc-miR-338_R+1
ENSSSCG00000003114	DHX34	PC-5p-9551_196
ENSSSCG00000003119	ZNF541	ssc-miR-192
ENSSSCG00000003119	ZNF541	ssc-miR-215_R+1
ENSSSCG00000003119	ZNF541	ssc-miR-192
ENSSSCG00000003119	ZNF541	ssc-miR-215_R+1
ENSSSCG00000003120	NAPA	hsa-miR-149-3p_L+1
ENSSSCG00000003120	NAPA	hsa-miR-27a-5p
ENSSSCG00000003121	KPTN	hsa-miR-4508_L+2R-1
ENSSSCG00000003134	GRWD1	PC-5p-9551_196
ENSSSCG00000003134	GRWD1	hsa-miR-149-3p_L+1
ENSSSCG00000003135	KCNJ14	PC-5p-9551_196
ENSSSCG00000003135	KCNJ14	ssc-miR-338_R+1
ENSSSCG00000003136	ENSSSCG00000003136	hsa-miR-149-3p_L+1

ENSSSCG00000003139	BCAT2	PC-5p-9551_196
ENSSSCG00000003139	BCAT2	hsa-miR-4508_L+2R-1
ENSSSCG00000003139	BCAT2	mmu-miR-1983_L-2R+1
ENSSSCG00000003139	BCAT2	ssc-miR-1839-3p_R+2
ENSSSCG00000003141	FUT1	PC-5p-9551_196
ENSSSCG00000003141	FUT1	hsa-miR-27a-5p
ENSSSCG00000003141	FUT1	ssc-miR-338_R+1
ENSSSCG00000003143	MAMSTR	mmu-miR-2137_L-2R-1_1ss16AG
ENSSSCG00000003147	ENSSSCG00000003147	hsa-mir-1973-p5_1ss5CT
ENSSSCG00000023153	ENSSSCG00000023153	hsa-mir-1973-p5_1ss5CT
ENSSSCG00000003148	ENSSSCG00000003148	PC-5p-9551_196
ENSSSCG00000003148	ENSSSCG00000003148	hsa-miR-149-3p_L+1
ENSSSCG00000003150	NUCB1	hsa-miR-149-3p_L+1
ENSSSCG00000003154	GYS1	PC-5p-9551_196
ENSSSCG00000003154	GYS1	hsa-miR-149-3p_L+1
ENSSSCG00000003154	GYS1	hsa-miR-27a-5p
ENSSSCG00000003154	GYS1	mmu-miR-2137_L-2R-1_1ss16AG
ENSSSCG00000003157	LIN7B	hsa-miR-200c-3p
ENSSSCG00000003161	PRRG2	hsa-miR-222-5p_L+2R-1
ENSSSCG00000003170	NR5A1	PC-5p-9551_196
ENSSSCG00000003177	BCL2L12	PC-5p-11132_163
ENSSSCG00000003177	BCL2L12	PC-5p-9551_196
ENSSSCG00000003182	SCAF1	ssc-miR-139-3p
ENSSSCG00000003183	RRAS	mmu-miR-2137_L-2R-1_1ss16AG
ENSSSCG00000003187	FCGRT	hsa-miR-149-3p_L+1
ENSSSCG00000003187	FCGRT	hsa-miR-149-3p_L+1
ENSSSCG00000003187	FCGRT	hsa-miR-149-3p_L+1
ENSSSCG00000003194	NR5A1	ssc-miR-192
ENSSSCG00000003194	NR5A1	ssc-miR-215_R+1
ENSSSCG00000003199	AP2A1	PC-5p-9551_196
ENSSSCG00000003199	AP2A1	hsa-miR-149-3p_L+1
ENSSSCG00000003201	ATF5	hsa-miR-141-3p_R+1
ENSSSCG00000003201	ATF5	hsa-miR-149-3p_L+1
ENSSSCG00000003204	VRK3	PC-5p-9551_196
ENSSSCG00000003204	VRK3	hsa-miR-149-3p_L+1
ENSSSCG00000003204	VRK3	ssc-miR-338_R+1
ENSSSCG00000003211	NR1H2	ssc-miR-192
ENSSSCG00000003211	NR1H2	ssc-miR-215_R+1
ENSSSCG00000003212	NAPSA	hsa-miR-4508_L+2R-1
ENSSSCG00000003214	KCNC3	hsa-miR-149-3p_L+1
ENSSSCG00000003215	SHANK1	hsa-miR-149-3p_L+1
ENSSSCG00000003215	SHANK1	ssc-miR-139-3p
ENSSSCG00000003215	SHANK1	hsa-miR-149-3p_L+1
ENSSSCG00000003215	SHANK1	ssc-miR-139-3p

ENSSSCG00000003222	SYT3	hsa-mir-1973-p5_1ss5CT
ENSSSCG00000003231	NKG7	hsa-miR-149-3p_L+1
ENSSSCG00000003235	CTU1	hsa-miR-27a-5p
ENSSSCG00000003248	ENSSSCG00000003248	ssc-miR-194b-5p_1ss10GA
ENSSSCG00000003253	ENSSSCG00000003253	hsa-miR-141-3p_R+1
ENSSSCG00000003253	ENSSSCG00000003253	hsa-miR-149-3p_L+1
ENSSSCG00000003253	ENSSSCG00000003253	hsa-miR-27a-5p
ENSSSCG00000003253	ENSSSCG00000003253	ssc-miR-338_R+1
ENSSSCG00000003257	CACNG6	PC-5p-9551_196
ENSSSCG00000003264	CNOT3	PC-5p-9551_196
ENSSSCG00000003268	TSEN34	hsa-miR-149-3p_L+1
ENSSSCG00000003276	GP6	ssc-miR-339_L-2
ENSSSCG00000003278	ENSSSCG00000003278	hsa-miR-222-5p_L+2R-1
ENSSSCG00000027895	NR5A1	mmu-miR-2137_L-2R-1_1ss16AG
ENSSSCG00000003284	NR5A1	sha-mir-24-1-p3_1ss2GC
ENSSSCG00000003284	NR5A1	ssc-miR-190b
ENSSSCG00000022675	ENSSSCG00000022675	mmu-miR-2137_L-2R-1_1ss16AG
ENSSSCG00000003288	HSPBP1	ssc-miR-339_L-2
ENSSSCG00000003322	ZNF582	hsa-miR-222-5p_L+2R-1
ENSSSCG00000003322	ZNF582	ssc-mir-374a-p5
ENSSSCG00000003329	ACAP3	PC-5p-9551_196
ENSSSCG00000003331	TNFRSF4	mmu-miR-2137_L-2R-1_1ss16AG
ENSSSCG00000003339	INTS11	PC-5p-9551_196
ENSSSCG00000003344	VWA1	hsa-miR-4508_L+2R-1
ENSSSCG00000003344	VWA1	ssc-miR-139-3p
ENSSSCG00000003344	VWA1	ssc-miR-339_L-2
ENSSSCG00000003360	WRAP73	hsa-miR-141-3p_R+1
ENSSSCG00000003360	WRAP73	ssc-miR-192
ENSSSCG00000003360	WRAP73	ssc-miR-215_R+1
ENSSSCG00000003361	ENSSSCG00000003361	ssc-miR-339_L-2
ENSSSCG00000003363	C1orf174	mmu-miR-1983_L-2R+1
ENSSSCG00000003369	ICMT	PC-5p-11132_163
ENSSSCG00000003369	ICMT	PC-5p-9551_196
ENSSSCG00000003369	ICMT	sha-mir-24-1-p3_1ss2GC
ENSSSCG00000003369	ICMT	ssc-miR-190b
ENSSSCG00000029135	NR5A1	hsa-miR-27a-5p
ENSSSCG00000003377	NR5A1	hsa-miR-141-3p_R+1

Supplementary Table S3. Predicted target genes of differentially expressed miRNAs in 300μM H2O2 treated porcine GCs

Gene ID	Symbol	miRNA ID

ENSSSCG00000000003	TTC38	PC-5p-12443_142
ENSSSCG00000000003	TTC38	ssc-miR-192
ENSSSCG00000000003	TTC38	ssc-miR-215_R+1
ENSSSCG00000000005	ENSSSCG00000000005	PC-3p-39317_30
ENSSSCG00000000007	TRMU	PC-3p-39317_30
ENSSSCG00000000018	ENSSSCG00000000018	mmu-let-7j_R-2
ENSSSCG00000000018	ENSSSCG00000000018	ssc-miR-339_R-2
ENSSSCG00000000029	SCUBE1	PC-3p-39317_30
ENSSSCG00000000029	SCUBE1	mmu-mir-6240-p5_1ss13AG
ENSSSCG00000000029	SCUBE1	ssc-mir-4332-p5_1ss18CA
ENSSSCG00000000031	MCAT	PC-5p-9551_196
ENSSSCG00000000036	PACSIN2	PC-5p-9551_196
ENSSSCG00000000036	PACSIN2	hsa-miR-141-3p_R+1
ENSSSCG00000000036	PACSIN2	hsa-miR-375
ENSSSCG00000000037	ARFGAP3	cfa-miR-8903_R-2_1ss21GA
ENSSSCG00000000037	ARFGAP3	ssc-miR-339_R-2
ENSSSCG00000000038	CYB5R3	PC-3p-39317_30
ENSSSCG00000000039	POLDIP3	PC-5p-12443_142
ENSSSCG00000000040	43346	hsa-miR-129-1-3p
ENSSSCG00000000040	43346	ssc-miR-192
ENSSSCG00000000040	43346	ssc-miR-215_R+1
ENSSSCG00000000042	ENSSSCG00000000042	hsa-miR-4792_1ss9GT
ENSSSCG00000000042	ENSSSCG00000000042	ssc-mir-4332-p5_1ss18CA
ENSSSCG00000020945	ENSSSCG00000020945	mmu-miR-107-5p
ENSSSCG00000023108	ENSSSCG00000023108	hsa-miR-222-5p_L+2R-1
ENSSSCG00000023108	ENSSSCG00000023108	hsa-miR-23b-5p
ENSSSCG00000023108	ENSSSCG00000023108	mmu-miR-107-5p
ENSSSCG00000023108	ENSSSCG00000023108	ssc-miR-424-5p_R-3
ENSSSCG00000000060	DESI1	PC-5p-9551_196
ENSSSCG00000000060	DESI1	ssc-miR-424-5p_R-3
ENSSSCG00000000062	CSDC2	ssc-mir-4332-p5_1ss18CA
ENSSSCG00000000063	POLR3H	ssc-mir-4332-p5_1ss18CA
ENSSSCG00000000064	ACO2	cfa-miR-8903_R-2_1ss21GA
ENSSSCG00000000066	L3MBTL2	PC-5p-9551_196

ENSSSCG00000000066	L3MBTL2	hsa-miR-1246_L+1R+2
ENSSSCG00000000066	L3MBTL2	hsa-miR-222-5p_L+2R-1
ENSSSCG00000000066	L3MBTL2	hsa-miR-4792_1ss9GT
ENSSSCG00000000066	L3MBTL2	mmu-let-7j_R-2
ENSSSCG00000000066	L3MBTL2	mmu-miR-107-5p
ENSSSCG00000000066	L3MBTL2	sha-miR-21_L+2R-2
ENSSSCG00000000066	L3MBTL2	ssc-miR-1839-3p_R+2
ENSSSCG00000000068	EP300	bta-mir-2478-p3_1ss20GA
ENSSSCG00000000077	ADSL	ssc-miR-181d-5p
ENSSSCG00000000077	ADSL	ssc-miR-19b
ENSSSCG00000000080	GRAP2	PC-5p-9551_196
ENSSSCG00000000083	RPS19BP1	cfa-miR-8903_R-2_1ss21GA
ENSSSCG00000000091	APOBEC3H	cfa-miR-8903_R-2_1ss21GA
ENSSSCG00000000095	GTPBP1	hsa-miR-4792_1ss9GT
ENSSSCG00000000095	GTPBP1	hsa-miR-590-5p_1ss19GA
ENSSSCG00000000114	PICK1	ssc-mir-4332-p5_1ss18CA
ENSSSCG00000000115	SOX10	PC-3p-39317_30
ENSSSCG00000000117	ENSSSCG00000000117	PC-5p-9551_196
ENSSSCG00000000117	ENSSSCG00000000117	hsa-miR-31-3p_R+1
ENSSSCG00000029945	ENSSSCG00000029945	PC-5p-9551_196
ENSSSCG00000000130	CYTH4	PC-3p-39317_30
ENSSSCG00000000130	CYTH4	PC-5p-9551_196
ENSSSCG00000000130	CYTH4	mmu-miR-26a-2-3p_1ss4GA
ENSSSCG00000000133	TST	hsa-miR-4792_1ss9GT
ENSSSCG00000000136	CSF2RB	PC-3p-39317_30
ENSSSCG00000000136	CSF2RB	ssc-miR-19b
ENSSSCG00000000137	NCF4	PC-3p-21836_71
ENSSSCG00000000137	NCF4	PC-3p-39317_30
ENSSSCG00000000137	NCF4	PC-5p-12443_142
ENSSSCG00000000137	NCF4	PC-5p-9551_196
ENSSSCG00000000137	NCF4	hsa-miR-129-1-3p
ENSSSCG00000000138	PVALB	sha-mir-24-1-p3_1ss2GC
ENSSSCG00000000142	FOXRED2	PC-5p-6765_300
ENSSSCG00000000142	FOXRED2	cfa-miR-8903_R-2_1ss21GA
ENSSSCG00000000142	FOXRED2	hsa-miR-222-5p_L+2R-1
ENSSSCG00000000142	FOXRED2	mmu-miR-26a-2-3p_1ss4GA
ENSSSCG00000000142	FOXRED2	ssc-miR-1839-3p_R+2
ENSSSCG00000000144	TXN2	PC-3p-21836_71
ENSSSCG00000000144	TXN2	PC-5p-9551_196
ENSSSCG00000000144	TXN2	hsa-miR-375
ENSSSCG00000000157	BPIFC	PC-3p-39317_30

ENSSSCG00000000157	BPIFC	PC-5p-9551_196
ENSSSCG00000000158	RTCB	PC-3p-21836_71
ENSSSCG00000000158	RTCB	PC-3p-39317_30
ENSSSCG00000000158	RTCB	hsa-miR-141-3p_R+1
ENSSSCG00000000158	RTCB	ssc-miR-181d-5p
ENSSSCG00000000160	PRDM4	mmu-miR-107-5p
ENSSSCG00000000160	PRDM4	ssc-miR-181d-5p
ENSSSCG00000000160	PRDM4	ssc-miR-424-5p_R-3
ENSSSCG00000000162	BTBD11	bta-mir-2478-p3_1ss20GA
ENSSSCG00000000162	BTBD11	ssc-miR-190b
ENSSSCG00000000162	BTBD11	ssc-miR-339_R-2
ENSSSCG00000000166	TMEM263	PC-3p-21836_71
ENSSSCG00000000175	ENSSSCG00000000175	PC-3p-39317_30
ENSSSCG00000000175	ENSSSCG00000000175	PC-5p-9551_196
ENSSSCG00000000176	CACNB3	PC-5p-9551_196
ENSSSCG00000000176	CACNB3	ssc-miR-424-5p_R-3
ENSSSCG00000000185	PRKAG1	PC-3p-39317_30
ENSSSCG00000000189	NR5A1	PC-3p-39317_30
ENSSSCG00000000193	NR5A1	ssc-miR-424-5p_R-3
ENSSSCG00000000191	NR5A1	PC-3p-39317_30
ENSSSCG00000000191	NR5A1	PC-5p-9551_196
ENSSSCG00000000194	NR5A1	ssc-miR-19b
ENSSSCG00000000202	ENSSSCG00000000202	ssc-miR-27b-5p_R-1
ENSSSCG00000000203	ENSSSCG00000000203	PC-3p-39317_30
ENSSSCG00000000206	FAIM2	mmu-miR-107-5p
ENSSSCG00000000214	ENSSSCG00000000214	PC-3p-39317_30
ENSSSCG00000000214	ENSSSCG00000000214	PC-5p-9551_196
ENSSSCG00000000214	ENSSSCG00000000214	hsa-miR-141-3p_R+1
ENSSSCG00000000216	ENSSSCG00000000216	PC-5p-9551_196
ENSSSCG00000000216	ENSSSCG00000000216	hsa-miR-23b-5p
ENSSSCG00000000216	ENSSSCG00000000216	hsa-miR-4792_1ss9GT
ENSSSCG00000000216	ENSSSCG00000000216	mmu-miR-107-5p

ENSSSCG00000000217	ENSSSCG00000000217	ssc-miR-19b
ENSSSCG00000000223	ENSSSCG00000000223	PC-3p-39317_30
ENSSSCG00000000223	ENSSSCG00000000223	mmu-miR-26a-2-3p_1ss4GA
ENSSSCG00000000223	ENSSSCG00000000223	ssc-miR-192
ENSSSCG00000000223	ENSSSCG00000000223	ssc-miR-215_R+1
ENSSSCG00000000224	SMAGP	mmu-miR-107-5p
ENSSSCG00000000232	ACVRL1	PC-5p-12443_142
ENSSSCG00000000232	ACVRL1	PC-5p-9551_196
ENSSSCG00000000232	ACVRL1	bta-mir-2478-p3_1ss20GA
ENSSSCG00000000232	ACVRL1	hsa-miR-23b-5p
ENSSSCG00000000232	ACVRL1	hsa-miR-4792_1ss9GT
ENSSSCG00000000232	ACVRL1	ssc-miR-1839-3p_R+2
ENSSSCG00000000232	ACVRL1	ssc-miR-339_R-2
ENSSSCG00000000232	ACVRL1	ssc-miR-424-5p_R-3
ENSSSCG00000000233	ACVR1B	PC-3p-39317_30
ENSSSCG00000000233	ACVR1B	bta-mir-2478-p3_1ss20GA
ENSSSCG00000000233	ACVR1B	sha-mir-24-1-p3_1ss2GC
ENSSSCG00000000233	ACVR1B	ssc-mir-4332-p5_1ss18CA
ENSSSCG00000000235	KRT82	PC-5p-9551_196
ENSSSCG00000000235	KRT82	mmu-let-7j_R-2
ENSSSCG00000000235	KRT82	ssc-miR-339_R-2
ENSSSCG00000000240	KRT85	PC-5p-12443_142
ENSSSCG00000000240	KRT85	ssc-miR-424-5p_R-3
ENSSSCG00000000243	ENSSSCG00000000243	ssc-miR-339_R-2
ENSSSCG00000000243	ENSSSCG00000000243	ssc-miR-424-5p_R-3
ENSSSCG00000000244	PPBP	PC-3p-21836_71
ENSSSCG00000000244	PPBP	cfa-miR-8903_R-2_1ss21GA
ENSSSCG00000000244	PPBP	hsa-miR-1246_L+1R+2
ENSSSCG00000000244	PPBP	ssc-miR-181d-5p
ENSSSCG00000000248	KRT5	cfa-miR-8903_R-2_1ss21GA
ENSSSCG00000000251	KRT1	PC-3p-21836_71
ENSSSCG00000000251	KRT1	ssc-miR-192
ENSSSCG00000000251	KRT1	ssc-miR-215_R+1
ENSSSCG00000000251	KRT1	ssc-miR-424-5p_R-3
ENSSSCG00000000252	KRT8	PC-3p-39317_30
ENSSSCG00000000258	RARG	PC-5p-9551_196
ENSSSCG00000000259	CSAD	hsa-miR-141-3p_R+1

ENSSSCG00000000259	CSAD	hsa-miR-23b-5p
ENSSSCG00000000262	SPRYD3	hsa-miR-141-3p_R+1
ENSSSCG00000000262	SPRYD3	ssc-miR-19b
ENSSSCG00000000262	SPRYD3	ssc-miR-339_R-2
ENSSSCG00000000262	SPRYD3	ssc-miR-424-5p_R-3
ENSSSCG00000000263	ENSSSCG00000000263	bta-mir-2478-p3_1ss20GA
ENSSSCG00000000263	ENSSSCG00000000263	cfa-miR-8903_R-2_1ss21GA
ENSSSCG00000000268	AAAS	PC-3p-39317_30
ENSSSCG00000000268	AAAS	PC-5p-6765_300
ENSSSCG00000000268	AAAS	PC-5p-9551_196
ENSSSCG00000000268	AAAS	hsa-miR-141-3p_R+1
ENSSSCG00000000271	AMHR2	PC-5p-9551_196
ENSSSCG00000000276	TARBP2	ssc-miR-424-5p_R-3
ENSSSCG00000000288	HNRNPA1	PC-5p-6765_300
ENSSSCG00000000288	HNRNPA1	ssc-miR-339_R-2
ENSSSCG00000000293	ITGA5	PC-5p-9551_196
ENSSSCG00000000293	ITGA5	hsa-miR-873-3p_L-1_1ss11GA
ENSSSCG00000000293	ITGA5	ssc-miR-339_R-2
ENSSSCG00000000295	ENSSSCG00000000295	hsa-miR-129-1-3p
ENSSSCG00000000297	ENSSSCG00000000297	PC-3p-39317_30
ENSSSCG00000000363	GDF11	PC-3p-39317_30
ENSSSCG00000000363	GDF11	bta-mir-2478-p3_1ss20GA
ENSSSCG00000000363	GDF11	cfa-miR-8903_R-2_1ss21GA
ENSSSCG00000000363	GDF11	hsa-miR-129-1-3p
ENSSSCG00000000363	GDF11	mmu-let-7j_R-2
ENSSSCG00000000363	GDF11	mmu-miR-26a-2-3p_1ss4GA
ENSSSCG00000000363	GDF11	ssc-miR-339_R-2
ENSSSCG00000000366	DNAJC14	bta-mir-2478-p3_1ss20GA
ENSSSCG00000000366	DNAJC14	hsa-miR-129-1-3p
ENSSSCG00000000366	DNAJC14	ssc-miR-192
ENSSSCG00000000366	DNAJC14	ssc-miR-215_R+1
ENSSSCG00000000366	DNAJC14	ssc-miR-27b-5p_R-1
ENSSSCG00000000368	MMP19	hsa-miR-129-1-3p
ENSSSCG00000000369	PYM1	PC-5p-9551_196
ENSSSCG00000000369	PYM1	cfa-miR-8903_R-2_1ss21GA
ENSSSCG00000000369	PYM1	hsa-miR-141-3p_R+1
ENSSSCG00000000369	PYM1	ssc-miR-424-5p_R-3
ENSSSCG00000000377	ENSSSCG00000000377	PC-3p-21836_71

ENSSSCG00000000377	ENSSSCG0000000037 7	PC-3p-39317_30
ENSSSCG00000000377	ENSSSCG0000000037 7	PC-5p-9551_196
ENSSSCG00000000379	ESYT1	PC-5p-9551_196
ENSSSCG00000000379	ESYT1	bta-mir-2478-p3_1ss20GA
ENSSSCG00000000379	ESYT1	hsa-miR-873-3p_L-1_1ss11GA
ENSSSCG00000000379	ESYT1	ssc-miR-339_R-2
ENSSSCG00000000382	ENSSSCG0000000038 2	PC-5p-9551_196
ENSSSCG00000000382	ENSSSCG0000000038 2	hsa-miR-129-1-3p
ENSSSCG00000000383	ENSSSCG0000000038 3	PC-5p-9551_196
ENSSSCG00000000383	ENSSSCG0000000038 3	hsa-miR-4792_1ss9GT
ENSSSCG00000000383	ENSSSCG0000000038 3	mmu-miR-107-5p
ENSSSCG00000000383	ENSSSCG0000000038 3	ssc-miR-339_R-2
ENSSSCG00000000393	PAN2	mmu-miR-107-5p
ENSSSCG00000000399	TIMELESS	hsa-miR-222-5p_L+2R-1
ENSSSCG00000000399	TIMELESS	mmu-let-7j_R-2
ENSSSCG00000000401	ENSSSCG0000000040 1	PC-5p-6765_300
ENSSSCG00000000401	ENSSSCG0000000040 1	ssc-miR-424-5p_R-3
ENSSSCG00000000403	BAZ2A	PC-3p-39317_30
ENSSSCG00000000403	BAZ2A	ssc-miR-424-5p_R-3
ENSSSCG00000000406	PTGES3	PC-5p-12443_142
ENSSSCG00000000406	PTGES3	bta-mir-2478-p3_1ss20GA
ENSSSCG00000000406	PTGES3	hsa-miR-141-3p_R+1
ENSSSCG00000000406	PTGES3	ssc-miR-19b
ENSSSCG00000025960	ENSSSCG0000002596 0	PC-3p-21836_71
ENSSSCG00000025960	ENSSSCG0000002596 0	PC-3p-39317_30
ENSSSCG00000025960	ENSSSCG0000002596 0	hsa-miR-23b-5p
ENSSSCG00000000411	ENSSSCG0000000041 1	PC-3p-21836_71
ENSSSCG00000000411	ENSSSCG0000000041 1	PC-3p-39317_30

ENSSSCG00000000411	ENSSSCG00000000411	hsa-miR-31-3p_R+1
ENSSSCG00000000411	ENSSSCG00000000411	mmu-miR-107-5p
ENSSSCG00000000411	ENSSSCG00000000411	ssc-miR-19b
ENSSSCG00000025542	ENSSSCG00000025542	PC-5p-9551_196
ENSSSCG00000026524	ENSSSCG00000026524	PC-3p-21836_71
ENSSSCG00000026524	ENSSSCG00000026524	PC-3p-39317_30
ENSSSCG00000026524	ENSSSCG00000026524	hsa-miR-23b-5p
ENSSSCG00000000436	ENSSSCG00000000436	PC-5p-12443_142
ENSSSCG00000000436	ENSSSCG00000000436	hsa-miR-23b-5p
ENSSSCG00000000436	ENSSSCG00000000436	ssc-mir-4332-p5_1ss18CA
ENSSSCG00000000437	ENSSSCG00000000437	PC-5p-9551_196
ENSSSCG00000000437	ENSSSCG00000000437	ssc-miR-1839-3p_R+2
ENSSSCG00000000437	ENSSSCG00000000437	ssc-miR-192
ENSSSCG00000000439	KIF5A	PC-5p-9551_196
ENSSSCG00000000443	ENSSSCG00000000443	hsa-miR-129-1-3p
ENSSSCG00000000443	ENSSSCG00000000443	ssc-miR-1839-3p_R+2
ENSSSCG00000000443	ENSSSCG00000000443	ssc-miR-339_R-2
ENSSSCG00000000451	ENSSSCG00000000451	ssc-miR-424-5p_R-3
ENSSSCG00000000453	ATP23	hsa-miR-590-5p_1ss19GA
ENSSSCG00000000456	SLC16A7	PC-3p-21836_71
ENSSSCG00000000456	SLC16A7	PC-5p-9551_196
ENSSSCG00000000456	SLC16A7	hsa-miR-129-1-3p
ENSSSCG00000000456	SLC16A7	mmu-miR-107-5p
ENSSSCG00000000456	SLC16A7	ssc-miR-192
ENSSSCG00000000456	SLC16A7	ssc-miR-215_R+1
ENSSSCG00000000456	SLC16A7	ssc-miR-339_R-2

ENSSSCG00000000457	ENSSSCG0000000045 7	ssc-miR-1839-3p_R+2
ENSSSCG00000000457	ENSSSCG0000000045 7	ssc-miR-424-5p_R-3
ENSSSCG00000000458	MON2	hsa-miR-4792_1ss9GT
ENSSSCG00000000458	MON2	mmu-let-7j_R-2
ENSSSCG00000000458	MON2	ssc-miR-192
ENSSSCG00000000458	MON2	ssc-miR-215_R+1
ENSSSCG00000028869	ENSSSCG0000002886 9	sha-miR-21_L+2R-2
ENSSSCG00000028869	ENSSSCG0000002886 9	ssc-miR-181d-5p
ENSSSCG00000028869	ENSSSCG0000002886 9	ssc-miR-190b
ENSSSCG00000028869	ENSSSCG0000002886 9	ssc-miR-19b
ENSSSCG00000000481	IFNG	ssc-miR-181d-5p
ENSSSCG00000000490	ENSSSCG0000000049 0	PC-3p-21836_71
ENSSSCG00000000490	ENSSSCG0000000049 0	hsa-miR-129-1-3p
ENSSSCG00000000490	ENSSSCG0000000049 0	ssc-miR-181d-5p
ENSSSCG00000000492	LYZ	PC-5p-12443_142
ENSSSCG00000000492	LYZ	hsa-miR-375
ENSSSCG00000000492	LYZ	hsa-miR-4792_1ss9GT
ENSSSCG00000000492	LYZ	mmu-let-7j_R-2
ENSSSCG00000000510	TMEM19	PC-3p-21836_71
ENSSSCG00000000510	TMEM19	hsa-miR-141-3p_R+1
ENSSSCG00000000510	TMEM19	hsa-miR-873-3p_L-1_1ss11GA
ENSSSCG00000000512	TBC1D15	hsa-miR-141-3p_R+1
ENSSSCG00000000512	TBC1D15	ssc-miR-190b
ENSSSCG00000000517	CAPS2	hsa-miR-873-3p_L-1_1ss11GA
ENSSSCG00000000519	GLIPR1	mmu-miR-107-5p
ENSSSCG00000000519	GLIPR1	mmu-miR-26a-2-3p_1ss4GA
ENSSSCG00000000519	GLIPR1	ssc-miR-1839-3p_R+2
ENSSSCG00000000519	GLIPR1	ssc-miR-192
ENSSSCG00000000519	GLIPR1	ssc-miR-215_R+1
ENSSSCG00000000519	GLIPR1	ssc-miR-339_R-2
ENSSSCG00000000519	GLIPR1	ssc-miR-424-5p_R-3
ENSSSCG00000000520	KRR1	PC-3p-21836_71
ENSSSCG00000000520	KRR1	PC-5p-9551_196
ENSSSCG00000000520	KRR1	bta-mir-2478-p3_1ss20GA
ENSSSCG00000000520	KRR1	cfa-miR-8903_R-2_1ss21GA

ENSSSCG00000000520	KRR1	hsa-miR-141-3p_R+1
ENSSSCG00000000520	KRR1	hsa-miR-222-5p_L+2R-1
ENSSSCG00000000520	KRR1	mmu-miR-26a-2-3p_1ss4GA
ENSSSCG00000000522	NAP1L1	hsa-miR-1246_L+1R+2
ENSSSCG00000000522	NAP1L1	mmu-let-7j_R-2
ENSSSCG00000000529	DNM1L	hsa-miR-375
ENSSSCG00000000529	DNM1L	ssc-miR-1839-3p_R+2
ENSSSCG00000000529	DNM1L	ssc-miR-190b
ENSSSCG00000000534	IPO8	PC-3p-39317_30
ENSSSCG00000000534	IPO8	cfa-miR-8903_R-2_1ss21GA
ENSSSCG00000000534	IPO8	hsa-miR-1246_L+1R+2
ENSSSCG00000000534	IPO8	ssc-miR-181d-5p
ENSSSCG00000000536	ERGIC2	PC-3p-21836_71
ENSSSCG00000000536	ERGIC2	cfa-miR-8903_R-2_1ss21GA
ENSSSCG00000000536	ERGIC2	mmu-miR-107-5p
ENSSSCG00000000539	ENSSSCG0000000053 9	PC-5p-12443_142
ENSSSCG00000000539	ENSSSCG0000000053 9	bta-mir-2478-p3_1ss20GA
ENSSSCG00000000539	ENSSSCG0000000053 9	hsa-miR-129-1-3p
ENSSSCG00000000539	ENSSSCG0000000053 9	hsa-miR-141-3p_R+1
ENSSSCG00000000539	ENSSSCG0000000053 9	ssc-miR-190b
ENSSSCG00000000541	FAR2	cfa-miR-8903_R-2_1ss21GA
ENSSSCG00000000541	FAR2	mmu-let-7j_R-2
ENSSSCG00000000544	PTHLH	ssc-miR-190b
ENSSSCG00000000544	PTHLH	ssc-miR-424-5p_R-3
ENSSSCG00000000545	KLHL42	PC-3p-21836_71
ENSSSCG00000000545	KLHL42	hsa-miR-23b-5p
ENSSSCG00000000545	KLHL42	ssc-miR-192
ENSSSCG00000000545	KLHL42	ssc-miR-19b
ENSSSCG00000000545	KLHL42	ssc-miR-215_R+1
ENSSSCG00000000547	MRPS35	hsa-miR-141-3p_R+1
ENSSSCG00000000549	PPFIBP1	PC-3p-21836_71
ENSSSCG00000000549	PPFIBP1	PC-5p-12443_142
ENSSSCG00000000549	PPFIBP1	ssc-miR-181d-5p
ENSSSCG00000000552	MED21	hsa-miR-590-5p_1ss19GA
ENSSSCG00000000552	MED21	mmu-miR-107-5p
ENSSSCG00000000553	TM7SF3	PC-5p-12443_142
ENSSSCG00000000553	TM7SF3	PC-5p-9551_196
ENSSSCG00000000553	TM7SF3	cfa-miR-8903_R-2_1ss21GA
ENSSSCG00000000553	TM7SF3	ssc-miR-424-5p_R-3

ENSSSCG00000000554	FGFR1OP2	hsa-miR-129-1-3p
ENSSSCG00000000554	FGFR1OP2	hsa-miR-141-3p_R+1
ENSSSCG00000000554	FGFR1OP2	hsa-miR-222-5p_L+2R-1
ENSSSCG00000000554	FGFR1OP2	ssc-miR-27b-5p_R-1
ENSSSCG00000000555	ENSSSCG00000000555	PC-3p-21836_71
ENSSSCG00000000555	ENSSSCG00000000555	PC-5p-6765_300
ENSSSCG00000000555	ENSSSCG00000000555	mmu-let-7j_R-2
ENSSSCG00000000555	ENSSSCG00000000555	mmu-miR-107-5p
ENSSSCG00000000570	ENSSSCG00000000570	PC-5p-12443_142
ENSSSCG00000000570	ENSSSCG00000000570	sha-miR-21_L+2R-2
ENSSSCG00000000570	ENSSSCG00000000570	ssc-miR-181d-5p
ENSSSCG00000000572	CMAS	mmu-miR-107-5p
ENSSSCG00000000574	ENSSSCG00000000574	ssc-miR-19b
ENSSSCG00000000577	GYS2	hsa-miR-1246_L+1R+2
ENSSSCG00000000579	GOLT1B	PC-5p-12443_142
ENSSSCG00000000579	GOLT1B	cfa-miR-8903_R-2_1ss21GA
ENSSSCG00000000579	GOLT1B	hsa-miR-873-3p_L-1_1ss11GA
ENSSSCG00000000579	GOLT1B	mmu-miR-107-5p
ENSSSCG00000000579	GOLT1B	sha-miR-21_L+2R-2
ENSSSCG00000000579	GOLT1B	ssc-miR-424-5p_R-3
ENSSSCG00000000580	RECQL	ssc-miR-1839-3p_R+2
ENSSSCG00000000580	RECQL	ssc-miR-27b-5p_R-1
ENSSSCG00000000583	PYROXD1	PC-5p-9551_196
ENSSSCG00000000583	PYROXD1	hsa-miR-222-5p_L+2R-1
ENSSSCG00000000583	PYROXD1	hsa-miR-873-3p_L-1_1ss11GA
ENSSSCG00000000584	SLCO1A2	PC-5p-6765_300
ENSSSCG00000000584	SLCO1A2	PC-5p-9551_196
ENSSSCG00000000584	SLCO1A2	hsa-miR-23b-5p
ENSSSCG00000000584	SLCO1A2	mmu-miR-107-5p
ENSSSCG00000000584	SLCO1A2	ssc-miR-1839-3p_R+2
ENSSSCG00000000584	SLCO1A2	ssc-miR-339_R-2
ENSSSCG00000000587	AEBP2	PC-3p-21836_71
ENSSSCG00000000587	AEBP2	hsa-miR-141-3p_R+1
ENSSSCG00000000587	AEBP2	mmu-miR-107-5p
ENSSSCG00000000587	AEBP2	ssc-miR-424-5p_R-3
ENSSSCG00000000588	PLEKHA5	ssc-miR-424-5p_R-3

ENSSSCG00000000597	LMO3	ssc-miR-181d-5p
ENSSSCG00000000600	ENSSSCG00000000600	mmu-miR-107-5p
ENSSSCG00000000601	ENSSSCG00000000601	cfa-miR-8903_R-2_1ss21GA
ENSSSCG00000000601	ENSSSCG00000000601	mmu-let-7j_R-2
ENSSSCG00000000602	RERG	ssc-miR-27b-5p_R-1
ENSSSCG00000000607	ART4	cfa-miR-8903_R-2_1ss21GA
ENSSSCG00000000607	ART4	ssc-miR-1839-3p_R+2
ENSSSCG00000000611	WBP11	PC-5p-12443_142
ENSSSCG00000000611	WBP11	PC-5p-9551_196
ENSSSCG00000000611	WBP11	cfa-miR-8903_R-2_1ss21GA
ENSSSCG00000000611	WBP11	hsa-miR-141-3p_R+1
ENSSSCG00000000611	WBP11	mmu-miR-107-5p
ENSSSCG00000000611	WBP11	ssc-miR-1839-3p_R+2
ENSSSCG00000000611	WBP11	ssc-miR-424-5p_R-3
ENSSSCG00000000612	ATF7IP	bta-mir-2478-p3_1ss20GA
ENSSSCG00000000612	ATF7IP	hsa-miR-1246_L+1R+2
ENSSSCG00000000612	ATF7IP	hsa-miR-873-3p_L-1_1ss11GA
ENSSSCG00000000612	ATF7IP	mmu-miR-107-5p
ENSSSCG00000000613	ENSSSCG00000000613	PC-5p-9551_196
ENSSSCG00000000619	FAM234B	PC-5p-6765_300
ENSSSCG00000000619	FAM234B	mmu-miR-107-5p
ENSSSCG00000000619	FAM234B	ssc-miR-192
ENSSSCG00000000619	FAM234B	ssc-miR-215_R+1
ENSSSCG00000000620	ENSSSCG00000000620	ssc-miR-19b
ENSSSCG00000000625	LRP6	PC-3p-21836_71
ENSSSCG00000000625	LRP6	hsa-miR-1246_L+1R+2
ENSSSCG00000000625	LRP6	hsa-miR-129-1-3p
ENSSSCG00000000625	LRP6	hsa-miR-31-3p_R+1
ENSSSCG00000000625	LRP6	mmu-let-7j_R-2
ENSSSCG00000000625	LRP6	ssc-miR-27b-5p_R-1
ENSSSCG00000000625	LRP6	ssc-miR-424-5p_R-3
ENSSSCG00000000634	STYK1	PC-3p-21836_71
ENSSSCG00000000634	STYK1	hsa-miR-129-1-3p
ENSSSCG00000000634	STYK1	hsa-miR-222-5p_L+2R-1
ENSSSCG00000000634	STYK1	mmu-miR-107-5p
ENSSSCG00000000638	ENSSSCG00000000638	ssc-miR-19b
ENSSSCG00000000640	ENSSSCG00000000640	mmu-miR-26a-2-3p_1ss4GA

ENSSSCG00000000645	GABARAPL1	hsa-miR-222-5p_L+2R-1
ENSSSCG00000000645	GABARAPL1	ssc-miR-19b
ENSSSCG00000000647	OLR1	hsa-miR-1246_L+1R+2
ENSSSCG00000000647	OLR1	sha-miR-21_L+2R-2
ENSSSCG00000000647	OLR1	ssc-miR-192
ENSSSCG00000000647	OLR1	ssc-miR-19b
ENSSSCG00000000647	OLR1	ssc-miR-215_R+1
ENSSSCG00000000648	CLEC7A	sha-miR-21_L+2R-2
ENSSSCG00000000648	CLEC7A	ssc-miR-19b
ENSSSCG00000000648	CLEC7A	sha-miR-21_L+2R-2
ENSSSCG00000000648	CLEC7A	ssc-miR-19b
ENSSSCG00000000648	CLEC7A	sha-miR-21_L+2R-2
ENSSSCG00000000648	CLEC7A	ssc-miR-19b
ENSSSCG00000000653	CD69	ssc-miR-424-5p_R-3
ENSSSCG00000000653	CD69	hsa-miR-141-3p_R+1
ENSSSCG00000000653	CD69	hsa-miR-375
ENSSSCG00000000653	CD69	hsa-miR-590-5p_1ss19GA
ENSSSCG00000000653	CD69	mmu-miR-26a-2-3p_1ss4GA
ENSSSCG00000000653	CD69	ssc-miR-181d-5p
ENSSSCG00000000653	CD69	ssc-miR-424-5p_R-3
ENSSSCG00000000656	CLEC2B	hsa-miR-1246_L+1R+2
ENSSSCG00000000656	CLEC2B	mmu-miR-26a-2-3p_1ss4GA
ENSSSCG00000000656	CLEC2B	ssc-miR-424-5p_R-3
ENSSSCG00000000662	M6PR	bta-mir-2478-p3_1ss20GA
ENSSSCG00000000672	ENSSSCG00000000672	ssc-miR-339_R-2
ENSSSCG00000000680	GPR162	hsa-miR-4792_1ss9GT
ENSSSCG00000000682	GNB3	PC-3p-39317_30
ENSSSCG00000000682	GNB3	PC-5p-6765_300
ENSSSCG00000000683	CDCA3	hsa-miR-4792_1ss9GT
ENSSSCG00000000683	CDCA3	ssc-mir-4332-p5_1ss18CA
ENSSSCG00000000684	USP5	PC-5p-12443_142
ENSSSCG00000000685	TPI1	PC-5p-9551_196
ENSSSCG00000000687	CD4	PC-5p-12443_142
ENSSSCG00000000687	CD4	cfa-miR-8903_R-2_1ss21GA
ENSSSCG00000000687	CD4	ssc-miR-339_R-2
ENSSSCG00000000687	CD4	PC-5p-12443_142
ENSSSCG00000000687	CD4	cfa-miR-8903_R-2_1ss21GA
ENSSSCG00000000687	CD4	ssc-miR-339_R-2
ENSSSCG00000000690	ENSSSCG00000000690	PC-5p-12443_142
ENSSSCG00000000690	ENSSSCG00000000690	ssc-miR-424-5p_R-3
ENSSSCG00000000702	ZNF384	PC-3p-21836_71

ENSSSCG00000000702	ZNF384	hsa-miR-222-5p_L+2R-1
ENSSSCG00000000704	TAPBPL	PC-5p-9551_196
ENSSSCG00000000704	TAPBPL	cfa-miR-8903_R-2_1ss21GA
ENSSSCG00000000704	TAPBPL	mmu-let-7j_R-2
ENSSSCG00000000704	TAPBPL	mmu-miR-107-5p
ENSSSCG00000000708	TNFRSF1A	PC-5p-12443_142
ENSSSCG00000000708	TNFRSF1A	ssc-miR-339_R-2
ENSSSCG00000000709	PLEKHG6	PC-5p-9551_196
ENSSSCG00000000709	PLEKHG6	hsa-miR-23b-5p
ENSSSCG00000000713	ENSSSCG00000000713	hsa-miR-129-1-3p
ENSSSCG00000000713	ENSSSCG00000000713	ssc-miR-424-5p_R-3
ENSSSCG00000000728	PARP11	PC-5p-9551_196
ENSSSCG00000000728	PARP11	hsa-miR-1246_L+1R+2
ENSSSCG00000000728	PARP11	mmu-miR-107-5p
ENSSSCG00000000730	PRMT8	PC-3p-39317_30
ENSSSCG00000000730	PRMT8	hsa-miR-4792_1ss9GT
ENSSSCG00000000730	PRMT8	ssc-miR-1839-3p_R+2
ENSSSCG00000000730	PRMT8	ssc-mir-4332-p5_1ss18CA
ENSSSCG00000000735	TSPAN9	hsa-miR-141-3p_R+1
ENSSSCG00000000735	TSPAN9	mmu-let-7j_R-2
ENSSSCG00000000735	TSPAN9	ssc-miR-339_R-2
ENSSSCG00000000736	TEAD4	cfa-miR-8903_R-2_1ss21GA
ENSSSCG00000000736	TEAD4	hsa-miR-23b-5p
ENSSSCG00000000736	TEAD4	cfa-miR-8903_R-2_1ss21GA
ENSSSCG00000000736	TEAD4	hsa-miR-23b-5p
ENSSSCG00000000739	FOXM1	bta-mir-2478-p3_1ss20GA
ENSSSCG00000000742	ITFG2	ssc-miR-19b
ENSSSCG00000000742	ITFG2	ssc-miR-339_R-2
ENSSSCG00000000746	WASH1	hsa-miR-222-5p_L+2R-1
ENSSSCG00000000749	SLC6A12	ssc-miR-339_R-2
ENSSSCG00000000751	CCDC77	PC-5p-9551_196
ENSSSCG00000000751	CCDC77	hsa-miR-1246_L+1R+2
ENSSSCG00000000753	WNK1	PC-3p-21836_71
ENSSSCG00000000753	WNK1	bta-mir-2478-p3_1ss20GA
ENSSSCG00000000753	WNK1	cfa-miR-8903_R-2_1ss21GA
ENSSSCG00000000753	WNK1	mmu-miR-107-5p
ENSSSCG00000000753	WNK1	sha-miR-21_L+2R-2
ENSSSCG00000000753	WNK1	ssc-miR-181d-5p
ENSSSCG00000000753	WNK1	ssc-miR-192
ENSSSCG00000000753	WNK1	ssc-miR-215_R+1
ENSSSCG00000000754	RAD52	PC-5p-9551_196
ENSSSCG00000000754	RAD52	sha-miR-21_L+2R-2

ENSSSCG00000000754	RAD52	ssc-miR-181d-5p
ENSSSCG00000000760	LRTM2	cfa-miR-8903_R-2_1ss21GA
ENSSSCG00000000760	LRTM2	ssc-miR-424-5p_R-3
ENSSSCG00000027190	ENSSSCG00000027190	hsa-miR-222-5p_L+2R-1
ENSSSCG00000000774	USP18	bta-mir-2478-p3_1ss20GA
ENSSSCG00000000778	CPNE8	PC-3p-21836_71
ENSSSCG00000000778	CPNE8	PC-5p-6765_300
ENSSSCG00000000778	CPNE8	PC-5p-9551_196
ENSSSCG00000000778	CPNE8	ssc-miR-190b
ENSSSCG00000000784	LRRK2	bta-mir-2478-p3_1ss20GA
ENSSSCG00000000784	LRRK2	hsa-miR-141-3p_R+1
ENSSSCG00000000784	LRRK2	ssc-miR-19b
ENSSSCG00000000784	LRRK2	ssc-miR-424-5p_R-3
ENSSSCG00000000793	PPHLN1	hsa-miR-1246_L+1R+2
ENSSSCG00000000793	PPHLN1	hsa-miR-141-3p_R+1
ENSSSCG00000000793	PPHLN1	hsa-miR-23b-5p
ENSSSCG00000000798	TWF1	PC-3p-21836_71
ENSSSCG00000000798	TWF1	PC-5p-12443_142
ENSSSCG00000000798	TWF1	PC-5p-9551_196
ENSSSCG00000000798	TWF1	ssc-miR-190b
ENSSSCG00000000798	TWF1	ssc-miR-19b
ENSSSCG00000000798	TWF1	ssc-miR-339_R-2
ENSSSCG00000000799	IRAK4	PC-3p-21836_71
ENSSSCG00000000799	IRAK4	PC-5p-6765_300
ENSSSCG00000000799	IRAK4	ssc-miR-27b-5p_R-1
ENSSSCG00000000799	IRAK4	ssc-miR-424-5p_R-3
ENSSSCG00000000800	PUS7L	PC-3p-21836_71
ENSSSCG00000000800	PUS7L	ssc-miR-181d-5p
ENSSSCG00000000800	PUS7L	ssc-miR-192
ENSSSCG00000000800	PUS7L	ssc-miR-215_R+1
ENSSSCG00000000800	PUS7L	ssc-miR-27b-5p_R-1
ENSSSCG00000000804	ANO6	PC-5p-6765_300
ENSSSCG00000000806	SCAF11	cfa-miR-8903_R-2_1ss21GA
ENSSSCG00000000806	SCAF11	hsa-miR-1246_L+1R+2
ENSSSCG00000000806	SCAF11	hsa-miR-141-3p_R+1
ENSSSCG00000000806	SCAF11	ssc-miR-181d-5p
ENSSSCG00000000806	SCAF11	ssc-miR-424-5p_R-3
ENSSSCG00000000816	SENP1	PC-5p-6765_300
ENSSSCG00000000816	SENP1	PC-5p-9551_196
ENSSSCG00000000816	SENP1	hsa-miR-129-1-3p
ENSSSCG00000000816	SENP1	hsa-miR-873-3p_L-1_1ss11GA
ENSSSCG00000000839	ALDH1L2	PC-5p-12443_142
ENSSSCG00000000839	ALDH1L2	PC-5p-6765_300

ENSSSCG00000000839	ALDH1L2	PC-5p-9551_196
ENSSSCG00000000839	ALDH1L2	hsa-miR-141-3p_R+1
ENSSSCG00000000839	ALDH1L2	hsa-miR-222-5p_L+2R-1
ENSSSCG00000000839	ALDH1L2	hsa-miR-23b-5p
ENSSSCG00000000839	ALDH1L2	mmu-let-7j_R-2
ENSSSCG00000000839	ALDH1L2	mmu-miR-107-5p
ENSSSCG00000000839	ALDH1L2	sha-miR-21_L+2R-2
ENSSSCG00000000839	ALDH1L2	ssc-miR-192
ENSSSCG00000000839	ALDH1L2	ssc-miR-215_R+1
ENSSSCG00000000839	ALDH1L2	ssc-miR-339_R-2
ENSSSCG00000000839	ALDH1L2	PC-5p-12443_142
ENSSSCG00000000839	ALDH1L2	PC-5p-6765_300
ENSSSCG00000000839	ALDH1L2	PC-5p-9551_196
ENSSSCG00000000839	ALDH1L2	hsa-miR-141-3p_R+1
ENSSSCG00000000839	ALDH1L2	hsa-miR-222-5p_L+2R-1
ENSSSCG00000000839	ALDH1L2	hsa-miR-23b-5p
ENSSSCG00000000839	ALDH1L2	mmu-let-7j_R-2
ENSSSCG00000000839	ALDH1L2	mmu-miR-107-5p
ENSSSCG00000000839	ALDH1L2	sha-miR-21_L+2R-2
ENSSSCG00000000839	ALDH1L2	ssc-miR-192
ENSSSCG00000000839	ALDH1L2	ssc-miR-215_R+1
ENSSSCG00000000839	ALDH1L2	ssc-miR-339_R-2
ENSSSCG00000000840	ENSSSCG00000000840	hsa-miR-141-3p_R+1
ENSSSCG00000000840	ENSSSCG00000000840	hsa-miR-222-5p_L+2R-1
ENSSSCG00000000840	ENSSSCG00000000840	hsa-miR-590-5p_1ss19GA
ENSSSCG00000000840	ENSSSCG00000000840	ssc-miR-181d-5p
ENSSSCG00000000841	APPL2	PC-5p-6765_300
ENSSSCG00000000841	APPL2	hsa-miR-141-3p_R+1
ENSSSCG00000000841	APPL2	sha-miR-21_L+2R-2
ENSSSCG00000000841	APPL2	ssc-miR-27b-5p_R-1
ENSSSCG00000000841	APPL2	ssc-miR-339_R-2
ENSSSCG00000000842	KANSL2	hsa-miR-141-3p_R+1
ENSSSCG00000000843	TXNRD1	bta-mir-2478-p3_1ss20GA
ENSSSCG00000000843	TXNRD1	ssc-miR-424-5p_R-3
ENSSSCG00000000844	NFYB	hsa-miR-141-3p_R+1
ENSSSCG00000000844	NFYB	ssc-miR-181d-5p
ENSSSCG00000000846	HCFC2	PC-3p-21836_71
ENSSSCG00000000846	HCFC2	PC-3p-39317_30
ENSSSCG00000000846	HCFC2	hsa-miR-1246_L+1R+2
ENSSSCG00000000846	HCFC2	mmu-miR-107-5p

ENSSSCG00000000846	HCFC2	mmu-miR-26a-2-3p_1ss4GA
ENSSSCG00000000846	HCFC2	ssc-miR-1839-3p_R+2
ENSSSCG00000000846	HCFC2	ssc-miR-190b
ENSSSCG00000000846	HCFC2	ssc-miR-192
ENSSSCG00000000846	HCFC2	ssc-miR-19b
ENSSSCG00000000846	HCFC2	ssc-miR-215_R+1
ENSSSCG00000000847	TDG	hsa-miR-129-1-3p
ENSSSCG00000000847	TDG	hsa-miR-141-3p_R+1
ENSSSCG00000000847	TDG	ssc-miR-192
ENSSSCG00000000847	TDG	ssc-miR-215_R+1
ENSSSCG00000000852	ENSSSCG00000000852	bta-mir-2478-p3_1ss20GA
ENSSSCG00000000854	ENSSSCG00000000854	hsa-miR-141-3p_R+1
ENSSSCG00000022156	ENSSSCG00000022156	mmu-miR-107-5p
ENSSSCG00000022156	ENSSSCG00000022156	sha-miR-21_L+2R-2
ENSSSCG00000000857	IGF1	bta-mir-2478-p3_1ss20GA
ENSSSCG00000000857	IGF1	cfa-miR-8903_R-2_1ss21GA
ENSSSCG00000000858	PMCH	PC-3p-21836_71
ENSSSCG00000000862	ENSSSCG00000000862	hsa-miR-129-1-3p
ENSSSCG00000000862	ENSSSCG00000000862	hsa-miR-222-5p_L+2R-1
ENSSSCG00000000863	SYCP3	PC-3p-21836_71
ENSSSCG00000000863	SYCP3	hsa-miR-873-3p_L-1_1ss11GA
ENSSSCG00000000863	SYCP3	ssc-miR-181d-5p
ENSSSCG00000000863	SYCP3	ssc-miR-190b
ENSSSCG00000000863	SYCP3	ssc-miR-19b
ENSSSCG00000000870	ARL1	hsa-miR-222-5p_L+2R-1
ENSSSCG00000000870	ARL1	mmu-miR-107-5p
ENSSSCG00000000870	ARL1	ssc-miR-424-5p_R-3
ENSSSCG00000000871	SLC5A8	ssc-miR-181d-5p
ENSSSCG00000000877	ENSSSCG00000000877	PC-3p-21836_71
ENSSSCG00000000877	ENSSSCG00000000877	PC-5p-6765_300
ENSSSCG00000000877	ENSSSCG00000000877	hsa-miR-222-5p_L+2R-1
ENSSSCG00000000877	ENSSSCG00000000877	hsa-miR-873-3p_L-1_1ss11GA
ENSSSCG00000000877	ENSSSCG00000000877	mmu-let-7j_R-2

ENSSSCG00000025682	ENSSSCG00000025682	hsa-miR-141-3p_R+1
ENSSSCG00000000884	ENSSSCG00000000884	ssc-miR-424-5p_R-3
ENSSSCG00000000886	SLC25A3	hsa-miR-141-3p_R+1
ENSSSCG00000000887	ENSSSCG00000000887	PC-3p-21836_71
ENSSSCG00000000887	ENSSSCG00000000887	bta-mir-2478-p3_1ss20GA
ENSSSCG00000000887	ENSSSCG00000000887	hsa-miR-1246_L+1R+2
ENSSSCG00000000887	ENSSSCG00000000887	sha-miR-21_L+2R-2
ENSSSCG00000000892	HAL	hsa-miR-4792_1ss9GT
ENSSSCG00000000892	HAL	mmu-miR-107-5p
ENSSSCG00000000893	AMDHD1	hsa-miR-141-3p_R+1
ENSSSCG00000000893	AMDHD1	mmu-mir-6240-p5_1ss13AG
ENSSSCG00000000893	AMDHD1	ssc-miR-1839-3p_R+2
ENSSSCG00000000896	NTN4	PC-3p-21836_71
ENSSSCG00000000896	NTN4	hsa-miR-1246_L+1R+2
ENSSSCG00000000900	ENSSSCG00000000900	hsa-miR-129-1-3p
ENSSSCG00000000902	NR2C1	PC-5p-6765_300
ENSSSCG00000000902	NR2C1	hsa-miR-129-1-3p
ENSSSCG00000000902	NR2C1	hsa-miR-141-3p_R+1
ENSSSCG00000000902	NR2C1	mmu-miR-107-5p
ENSSSCG00000000902	NR2C1	ssc-miR-192
ENSSSCG00000000902	NR2C1	ssc-miR-215_R+1
ENSSSCG00000000905	NDUFA12	PC-5p-12443_142
ENSSSCG00000000907	PLXNC1	PC-5p-6765_300
ENSSSCG00000000907	PLXNC1	PC-5p-9551_196
ENSSSCG00000000907	PLXNC1	hsa-miR-129-1-3p
ENSSSCG00000000907	PLXNC1	hsa-miR-141-3p_R+1
ENSSSCG00000000907	PLXNC1	ssc-miR-181d-5p
ENSSSCG00000000907	PLXNC1	ssc-miR-19b
ENSSSCG00000000907	PLXNC1	ssc-miR-424-5p_R-3
ENSSSCG00000000908	UBE2N	PC-5p-12443_142
ENSSSCG00000000910	SOCS2	PC-3p-21836_71
ENSSSCG00000000910	SOCS2	hsa-miR-1246_L+1R+2
ENSSSCG00000000910	SOCS2	hsa-miR-873-3p_L-1_1ss11GA
ENSSSCG00000000912	EEA1	bta-mir-2478-p3_1ss20GA
ENSSSCG00000000912	EEA1	hsa-miR-590-5p_1ss19GA
ENSSSCG00000000912	EEA1	mmu-miR-26a-2-3p_1ss4GA
ENSSSCG00000000912	EEA1	sha-miR-21_L+2R-2

ENSSSCG00000000912	EEA1	ssc-miR-27b-5p_R-1
ENSSSCG00000000912	EEA1	ssc-miR-424-5p_R-3
ENSSSCG00000000914	BTG1	hsa-miR-1246_L+1R+2
ENSSSCG00000000914	BTG1	ssc-miR-19b
ENSSSCG00000000916	LUM	PC-3p-21836_71
ENSSSCG00000000916	LUM	PC-5p-12443_142
ENSSSCG00000000916	LUM	PC-5p-6765_300
ENSSSCG00000000916	LUM	mmu-miR-107-5p
ENSSSCG00000000916	LUM	mmu-miR-26a-2-3p_1ss4GA
ENSSSCG00000000916	LUM	ssc-miR-192
ENSSSCG00000000916	LUM	ssc-miR-215_R+1
ENSSSCG00000000921	ENSSSCG0000000092 1	hsa-miR-222-5p_L+2R-1
ENSSSCG00000000921	ENSSSCG0000000092 1	mmu-miR-107-5p
ENSSSCG00000000924	C12orf50	PC-3p-21836_71
ENSSSCG00000000924	C12orf50	ssc-miR-1839-3p_R+2
ENSSSCG00000000925	C12orf29	PC-5p-9551_196
ENSSSCG00000000925	C12orf29	hsa-miR-1246_L+1R+2
ENSSSCG00000000925	C12orf29	hsa-miR-23b-5p
ENSSSCG00000000925	C12orf29	hsa-miR-31-3p_R+1
ENSSSCG00000000925	C12orf29	hsa-miR-590-5p_1ss19GA
ENSSSCG00000000925	C12orf29	ssc-miR-181d-5p
ENSSSCG00000000925	C12orf29	ssc-miR-192
ENSSSCG00000000925	C12orf29	ssc-miR-215_R+1
ENSSSCG00000000925	C12orf29	ssc-miR-27b-5p_R-1
ENSSSCG00000000925	C12orf29	ssc-miR-424-5p_R-3
ENSSSCG00000000926	CEP290	sha-miR-21_L+2R-2
ENSSSCG00000000926	CEP290	ssc-miR-190b
ENSSSCG00000000931	MGAT4C	mmu-let-7j_R-2
ENSSSCG00000000931	MGAT4C	ssc-miR-181d-5p
ENSSSCG00000026533	MYF6	bta-mir-2478-p3_1ss20GA
ENSSSCG00000026533	MYF6	hsa-miR-23b-5p
ENSSSCG00000026533	MYF6	ssc-miR-181d-5p
ENSSSCG00000000939	ACSS3	ssc-miR-424-5p_R-3
ENSSSCG00000000942	CCDC59	sha-miR-21_L+2R-2
ENSSSCG00000000948	SYT1	hsa-miR-31-3p_R+1
ENSSSCG00000000948	SYT1	hsa-miR-873-3p_L-1_1ss11GA
ENSSSCG00000000948	SYT1	mmu-let-7j_R-2
ENSSSCG00000000948	SYT1	ssc-miR-19b
ENSSSCG00000000950	E2F7	ssc-miR-181d-5p
ENSSSCG00000000950	E2F7	ssc-miR-1839-3p_R+2
ENSSSCG00000000950	E2F7	ssc-miR-424-5p_R-3
ENSSSCG00000000951	CSRP2	PC-5p-6765_300

ENSSSCG00000000952	ENSSSCG00000000952	hsa-miR-222-5p_L+2R-1
ENSSSCG00000000952	ENSSSCG00000000952	sha-miR-21_L+2R-2
ENSSSCG00000000952	ENSSSCG00000000952	ssc-miR-181d-5p
ENSSSCG00000000952	ENSSSCG00000000952	ssc-miR-27b-5p_R-1
ENSSSCG00000000955	TBC1D22A	hsa-miR-4792_1ss9GT
ENSSSCG00000024510	ENSSSCG00000024510	sha-miR-21_L+2R-2
ENSSSCG00000024510	ENSSSCG00000024510	ssc-miR-181d-5p
ENSSSCG00000024510	ENSSSCG00000024510	ssc-miR-190b
ENSSSCG00000024510	ENSSSCG00000024510	ssc-miR-19b
ENSSSCG00000000959	ENSSSCG00000000959	hsa-miR-23b-5p
ENSSSCG00000000959	ENSSSCG00000000959	ssc-miR-339_R-2
ENSSSCG00000000964	LMF2	ssc-mir-4332-p5_1ss18CA
ENSSSCG00000000965	NCAPH2	ssc-miR-424-5p_R-3
ENSSSCG00000000970	PPP6R2	ssc-miR-339_R-2
ENSSSCG00000000973	SELO	PC-5p-9551_196
ENSSSCG00000000973	SELO	ssc-miR-19b
ENSSSCG00000000974	TRABD	hsa-miR-129-1-3p
ENSSSCG00000000974	TRABD	ssc-miR-339_R-2
ENSSSCG00000000974	TRABD	ssc-mir-4332-p5_1ss18CA
ENSSSCG00000000978	MLC1	PC-5p-6765_300
ENSSSCG00000000985	NR5A1	PC-5p-9551_196
ENSSSCG00000027292	ENSSSCG00000027292	cfa-miR-8903_R-2_1ss21GA
ENSSSCG00000000994	GMDS	ssc-miR-192
ENSSSCG00000000994	GMDS	ssc-miR-215_R+1
ENSSSCG00000000997	PPP1R3G	PC-5p-6765_300
ENSSSCG00000000998	RPP40	ssc-miR-192
ENSSSCG00000000998	RPP40	ssc-miR-215_R+1
ENSSSCG00000000998	RPP40	ssc-miR-424-5p_R-3
ENSSSCG00000000999	ENSSSCG00000000999	PC-5p-9551_196
ENSSSCG00000000999	ENSSSCG00000000999	ssc-miR-181d-5p
ENSSSCG00000001000	ECI2	PC-3p-39317_30

ENSSSCG00000001000	ECI2	PC-5p-9551_196
ENSSSCG00000001009	RIPK1	hsa-miR-129-1-3p
ENSSSCG00000001009	RIPK1	hsa-miR-141-3p_R+1
ENSSSCG00000001009	RIPK1	ssc-miR-424-5p_R-3
ENSSSCG00000001009	RIPK1	ssc-mir-4332-p5_1ss18CA
ENSSSCG00000026211	ENSSSCG00000026211	PC-5p-6765_300
ENSSSCG00000025199	ENSSSCG00000025199	PC-3p-21836_71
ENSSSCG00000025199	ENSSSCG00000025199	ssc-miR-19b
ENSSSCG00000025199	ENSSSCG00000025199	ssc-miR-27b-5p_R-1
ENSSSCG00000024310	ENSSSCG00000024310	PC-3p-21836_71
ENSSSCG00000024310	ENSSSCG00000024310	ssc-miR-19b
ENSSSCG00000024310	ENSSSCG00000024310	ssc-miR-27b-5p_R-1
ENSSSCG00000026199	ENSSSCG00000026199	PC-5p-6765_300
ENSSSCG00000001021	RREB1	PC-5p-9551_196
ENSSSCG00000001021	RREB1	ssc-miR-424-5p_R-3
ENSSSCG00000001027	BMP6	hsa-miR-23b-5p
ENSSSCG00000001027	BMP6	ssc-miR-339_R-2
ENSSSCG00000001027	BMP6	ssc-mir-4332-p5_1ss18CA
ENSSSCG00000001030	ENSSSCG00000001030	PC-3p-39317_30
ENSSSCG00000001030	ENSSSCG00000001030	cfa-miR-8903_R-2_1ss21GA
ENSSSCG00000001030	ENSSSCG00000001030	hsa-miR-1246_L+1R+2
ENSSSCG00000001030	ENSSSCG00000001030	hsa-miR-141-3p_R+1
ENSSSCG00000001030	ENSSSCG00000001030	hsa-miR-23b-5p
ENSSSCG00000001033	SLC35B3	cfa-miR-8903_R-2_1ss21GA
ENSSSCG00000001033	SLC35B3	ssc-miR-181d-5p
ENSSSCG00000001033	SLC35B3	ssc-miR-190b
ENSSSCG00000001033	SLC35B3	ssc-miR-19b
ENSSSCG00000001036	TFAP2A	PC-3p-21836_71
ENSSSCG00000001036	TFAP2A	hsa-miR-141-3p_R+1
ENSSSCG00000001036	TFAP2A	hsa-miR-23b-5p
ENSSSCG00000001036	TFAP2A	ssc-miR-424-5p_R-3

ENSSSCG00000001039	GCNT2	PC-5p-9551_196
ENSSSCG00000001046	ENSSSCG0000000104 6	PC-3p-39317_30
ENSSSCG00000001046	ENSSSCG0000000104 6	hsa-miR-1246_L+1R+2
ENSSSCG00000001046	ENSSSCG0000000104 6	hsa-miR-23b-5p
ENSSSCG00000001049	HIVEP1	PC-5p-12443_142
ENSSSCG00000001049	HIVEP1	hsa-miR-222-5p_L+2R-1
ENSSSCG00000001049	HIVEP1	mmu-let-7j_R-2
ENSSSCG00000001049	HIVEP1	mmu-miR-107-5p
ENSSSCG00000001053	TBC1D7	hsa-miR-141-3p_R+1
ENSSSCG00000001056	RANBP9	hsa-miR-141-3p_R+1
ENSSSCG00000001056	RANBP9	ssc-miR-424-5p_R-3
ENSSSCG00000001057	MCUR1	PC-5p-12443_142
ENSSSCG00000001057	MCUR1	PC-5p-9551_196
ENSSSCG00000001057	MCUR1	hsa-miR-222-5p_L+2R-1
ENSSSCG00000001057	MCUR1	hsa-miR-873-3p_L-1_1ss11GA
ENSSSCG00000001057	MCUR1	mmu-miR-107-5p
ENSSSCG00000001063	MYLIP	PC-3p-39317_30
ENSSSCG00000001063	MYLIP	mmu-miR-26a-2-3p_1ss4GA
ENSSSCG00000001063	MYLIP	ssc-miR-19b
ENSSSCG00000001066	RBM24	PC-5p-9551_196
ENSSSCG00000001066	RBM24	bta-mir-2478-p3_1ss20GA
ENSSSCG00000001066	RBM24	hsa-miR-141-3p_R+1
ENSSSCG00000001066	RBM24	hsa-miR-375
ENSSSCG00000001066	RBM24	sha-miR-21_L+2R-2
ENSSSCG00000001066	RBM24	ssc-miR-1839-3p_R+2
ENSSSCG00000001068	ENSSSCG0000000106 8	mmu-let-7j_R-2
ENSSSCG00000001068	ENSSSCG0000000106 8	ssc-miR-27b-5p_R-1
ENSSSCG00000001068	ENSSSCG0000000106 8	ssc-miR-424-5p_R-3
ENSSSCG00000001073	TPMT	hsa-miR-23b-5p
ENSSSCG00000001073	TPMT	ssc-miR-1839-3p_R+2
ENSSSCG00000001074	KDM1B	PC-5p-6765_300
ENSSSCG00000001074	KDM1B	mmu-miR-26a-2-3p_1ss4GA
ENSSSCG00000001074	KDM1B	ssc-miR-27b-5p_R-1
ENSSSCG00000001076	RNF144B	PC-5p-9551_196
ENSSSCG00000001076	RNF144B	hsa-miR-23b-5p
ENSSSCG00000001076	RNF144B	ssc-miR-181d-5p
ENSSSCG00000001076	RNF144B	ssc-miR-424-5p_R-3
ENSSSCG00000001079	E2F3	bta-mir-2478-p3_1ss20GA

ENSSSCG00000001079	E2F3	hsa-miR-141-3p_R+1
ENSSSCG00000001079	E2F3	hsa-miR-222-5p_L+2R-1
ENSSSCG00000001079	E2F3	hsa-miR-4792_1ss9GT
ENSSSCG00000001079	E2F3	hsa-miR-873-3p_L-1_1ss11GA
ENSSSCG00000001079	E2F3	sha-miR-21_L+2R-2
ENSSSCG00000001079	E2F3	ssc-miR-424-5p_R-3
ENSSSCG00000001080	CDKAL1	ssc-miR-27b-5p_R-1
ENSSSCG00000001085	DCDC2	hsa-miR-222-5p_L+2R-1
ENSSSCG00000001085	DCDC2	ssc-miR-27b-5p_R-1
ENSSSCG00000001087	MRS2	PC-3p-39317_30
ENSSSCG00000001087	MRS2	hsa-miR-873-3p_L-1_1ss11GA
ENSSSCG00000001087	MRS2	ssc-miR-339_R-2
ENSSSCG00000001087	MRS2	ssc-miR-424-5p_R-3
ENSSSCG00000001091	KIAA0319	hsa-miR-141-3p_R+1
ENSSSCG00000001091	KIAA0319	mmu-miR-107-5p
ENSSSCG00000001091	KIAA0319	ssc-miR-181d-5p
ENSSSCG00000001094	C6orf62	PC-3p-21836_71
ENSSSCG00000001094	C6orf62	cfa-miR-8903_R-2_1ss21GA
ENSSSCG00000001094	C6orf62	ssc-miR-181d-5p
ENSSSCG00000001094	C6orf62	ssc-miR-1839-3p_R+2
ENSSSCG00000001094	C6orf62	ssc-miR-192
ENSSSCG00000001094	C6orf62	ssc-miR-215_R+1
ENSSSCG00000001100	CARMIL1	hsa-miR-1246_L+1R+2
ENSSSCG00000027958	ENSSSCG00000027958	PC-3p-21836_71
ENSSSCG00000027958	ENSSSCG00000027958	ssc-miR-192
ENSSSCG00000027958	ENSSSCG00000027958	ssc-miR-215_R+1
ENSSSCG00000027958	ENSSSCG00000027958	ssc-miR-339_R-2
ENSSSCG00000001156	ZNF322	PC-5p-9551_196
ENSSSCG00000022394	ENSSSCG00000022394	PC-3p-21836_71
ENSSSCG00000022394	ENSSSCG00000022394	ssc-miR-192
ENSSSCG00000022394	ENSSSCG00000022394	ssc-miR-215_R+1
ENSSSCG00000022394	ENSSSCG00000022394	ssc-miR-339_R-2
ENSSSCG00000001201	ENSSSCG00000001201	ssc-miR-181d-5p
ENSSSCG00000001202	ZNF389	hsa-miR-141-3p_R+1
ENSSSCG00000001203	ZSCAN9	ssc-miR-424-5p_R-3

ENSSSCG00000001209	ZSCAN12	PC-3p-21836_71
ENSSSCG00000001209	ZSCAN12	PC-5p-12443_142
ENSSSCG00000001209	ZSCAN12	bta-mir-2478-p3_1ss20GA
ENSSSCG00000001209	ZSCAN12	hsa-miR-873-3p_L-1_1ss11GA
ENSSSCG00000001209	ZSCAN12	mmu-miR-26a-2-3p_1ss4GA
ENSSSCG00000001209	ZSCAN12	ssc-miR-181d-5p
ENSSSCG00000001209	ZSCAN12	ssc-miR-1839-3p_R+2
ENSSSCG00000001209	ZSCAN12	ssc-miR-192
ENSSSCG00000001209	ZSCAN12	ssc-miR-215_R+1
ENSSSCG00000001214	GPX5	hsa-miR-873-3p_L-1_1ss11GA
ENSSSCG00000001214	GPX5	mmu-miR-26a-2-3p_1ss4GA
ENSSSCG00000001219	TRIM27	hsa-miR-129-1-3p
ENSSSCG00000001219	TRIM27	ssc-miR-190b
ENSSSCG00000001231	SLA-1	bta-mir-2478-p3_1ss20GA
ENSSSCG00000024161	SLA-7	hsa-miR-23b-5p
ENSSSCG00000001233	TRIM26	PC-3p-21836_71
ENSSSCG00000001234	TRIM10	hsa-miR-129-1-3p
ENSSSCG00000001235	TRIM15	cfa-miR-8903_R-2_1ss21GA
ENSSSCG00000001236	TRIM40	bta-mir-2478-p3_1ss20GA
ENSSSCG00000024259	ENSSSCG00000024259	PC-5p-9551_196
ENSSSCG00000024259	ENSSSCG00000024259	hsa-miR-4792_1ss9GT
ENSSSCG00000024259	ENSSSCG00000024259	ssc-miR-181d-5p
ENSSSCG00000024259	ENSSSCG00000024259	ssc-miR-424-5p_R-3
ENSSSCG00000028347	PPP1R11	PC-5p-9551_196
ENSSSCG00000028347	PPP1R11	hsa-miR-4792_1ss9GT
ENSSSCG00000028347	PPP1R11	ssc-miR-181d-5p
ENSSSCG00000028347	PPP1R11	ssc-miR-424-5p_R-3
ENSSSCG00000001239	ENSSSCG00000001239	hsa-miR-141-3p_R+1
ENSSSCG00000001242	GABBR1	cfa-miR-8903_R-2_1ss21GA
ENSSSCG00000001242	GABBR1	ssc-miR-339_R-2
ENSSSCG00000001242	GABBR1	ssc-mir-4332-p5_1ss18CA
ENSSSCG00000022261	ENSSSCG00000022261	ssc-miR-19b
ENSSSCG00000001245	ZNRD1	hsa-miR-141-3p_R+1
ENSSSCG00000001252	UBD	ssc-miR-192
ENSSSCG00000001252	UBD	ssc-miR-215_R+1
ENSSSCG00000001346	ABCF1	ssc-miR-424-5p_R-3
ENSSSCG00000001347	PPP1R10	mmu-miR-107-5p
ENSSSCG00000001362	MDC1	hsa-miR-141-3p_R+1

ENSSSCG00000001379	TUBB	PC-3p-21836_71
ENSSSCG00000001379	TUBB	mmu-miR-107-5p
ENSSSCG00000001382	DDR1	PC-3p-39317_30
ENSSSCG00000001390	PSORS1C2	hsa-miR-222-5p_L+2R-1
ENSSSCG00000001393	POU5F1	mmu-miR-107-5p
ENSSSCG00000001394	MIC-2	ssc-miR-1839-3p_R+2
ENSSSCG00000001394	MIC-2	ssc-miR-424-5p_R-3
ENSSSCG00000024161	SLA-7	hsa-miR-23b-5p
ENSSSCG00000001403	NR5A1	cfa-miR-8903_R-2_1ss21GA
ENSSSCG00000001404	NR5A1	PC-5p-9551_196
ENSSSCG00000001404	NR5A1	sha-mir-24-1-p3_1ss2GC
ENSSSCG00000001404	NR5A1	ssc-miR-181d-5p
ENSSSCG00000001405	NR5A1	ssc-miR-181d-5p
ENSSSCG00000001412	C6orf47	bta-mir-2478-p3_1ss20GA
ENSSSCG00000001414	CSNK2B	hsa-miR-23b-5p
ENSSSCG00000001414	CSNK2B	hsa-miR-873-3p_L-1_1ss11GA
ENSSSCG00000023611	ENSSSCG00000023611	hsa-miR-4792_1ss9GT
ENSSSCG00000001433	NR5A1	hsa-miR-141-3p_R+1
ENSSSCG00000001435	AGPAT1	PC-5p-9551_196
ENSSSCG00000001439	GPSM3	ssc-mir-4332-p5_1ss18CA
ENSSSCG00000001456	SLA-DQA	mmu-miR-107-5p
ENSSSCG00000026951	ENSSSCG00000026951	PC-5p-6765_300
ENSSSCG00000028872	PSMB8	PC-3p-39317_30
ENSSSCG00000028872	PSMB8	PC-5p-6765_300
ENSSSCG00000001469	NR5A1	cfa-miR-8903_R-2_1ss21GA
ENSSSCG00000001469	NR5A1	ssc-miR-424-5p_R-3
ENSSSCG00000001472	SLA-DOA	hsa-miR-141-3p_R+1
ENSSSCG00000001474	RXRB	hsa-miR-23b-5p
ENSSSCG00000001476	HSD17B8	PC-3p-39317_30
ENSSSCG00000001478	ENSSSCG00000001478	ssc-miR-1839-3p_R+2
ENSSSCG00000001478	ENSSSCG00000001478	ssc-miR-192
ENSSSCG00000001478	ENSSSCG00000001478	ssc-miR-215_R+1
ENSSSCG00000001479	HMGCLL1	PC-5p-12443_142
ENSSSCG00000001479	HMGCLL1	hsa-miR-1246_L+1R+2
ENSSSCG00000001479	HMGCLL1	hsa-miR-141-3p_R+1
ENSSSCG00000001479	HMGCLL1	mmu-let-7j_R-2
ENSSSCG00000001479	HMGCLL1	ssc-miR-181d-5p
ENSSSCG00000001479	HMGCLL1	ssc-miR-190b
ENSSSCG00000001483	FAM83B	PC-5p-6765_300

ENSSSCG00000001483	FAM83B	hsa-miR-1246_L+1R+2
ENSSSCG00000001483	FAM83B	ssc-miR-181d-5p
ENSSSCG00000001486	LRRC1	hsa-miR-141-3p_R+1
ENSSSCG00000001488	GCLC	PC-5p-12443_142
ENSSSCG00000001488	GCLC	ssc-miR-424-5p_R-3
ENSSSCG00000001498	BEND6	cfa-miR-8903_R-2_1ss21GA
ENSSSCG00000001498	BEND6	mmu-miR-107-5p
ENSSSCG00000001498	BEND6	ssc-miR-424-5p_R-3
ENSSSCG00000001499	DST	mmu-let-7j_R-2
ENSSSCG00000001499	DST	sha-miR-21_L+2R-2
ENSSSCG00000001503	B3GALT4	ssc-miR-339_R-2
ENSSSCG00000001506	NR5A1	PC-5p-12443_142
ENSSSCG00000001506	NR5A1	bta-mir-2478-p3_1ss20GA
ENSSSCG00000001506	NR5A1	cfa-miR-8903_R-2_1ss21GA
ENSSSCG00000001506	NR5A1	ssc-mir-4332-p5_1ss18CA
ENSSSCG00000001510	ENSSSCG0000000151 0	PC-3p-39317_30
ENSSSCG00000001510	ENSSSCG0000000151 0	ssc-miR-1839-3p_R+2
ENSSSCG00000001516	BAK1	PC-5p-9551_196
ENSSSCG00000001516	BAK1	ssc-miR-192
ENSSSCG00000001516	BAK1	ssc-miR-215_R+1
ENSSSCG00000001516	BAK1	ssc-miR-339_R-2
ENSSSCG00000001518	ITPR3	PC-3p-39317_30
ENSSSCG00000001518	ITPR3	PC-5p-6765_300
ENSSSCG00000001518	ITPR3	cfa-miR-8903_R-2_1ss21GA
ENSSSCG00000001518	ITPR3	hsa-miR-222-5p_L+2R-1
ENSSSCG00000001518	ITPR3	hsa-miR-23b-5p
ENSSSCG00000001518	ITPR3	hsa-miR-4792_1ss9GT
ENSSSCG00000001526	HMGA1	PC-5p-9551_196
ENSSSCG00000001526	HMGA1	cfa-miR-8903_R-2_1ss21GA
ENSSSCG00000001526	HMGA1	hsa-miR-4792_1ss9GT
ENSSSCG00000001526	HMGA1	ssc-miR-424-5p_R-3
ENSSSCG00000001528	NR5A1	hsa-miR-129-1-3p
ENSSSCG00000001531	NR5A1	bta-mir-2478-p3_1ss20GA
ENSSSCG00000001539	PPARD	PC-5p-9551_196
ENSSSCG00000001544	TEAD3	PC-3p-21836_71
ENSSSCG00000001556	MAPK14	PC-3p-39317_30
ENSSSCG00000001556	MAPK14	PC-5p-12443_142
ENSSSCG00000001556	MAPK14	ssc-miR-19b
ENSSSCG00000001556	MAPK14	ssc-miR-339_R-2
ENSSSCG00000001562	KCTD20	ssc-miR-1839-3p_R+2
ENSSSCG00000001563	NR5A1	bta-mir-2478-p3_1ss20GA
ENSSSCG00000001563	NR5A1	mmu-miR-107-5p

ENSSSCG00000001563	NR5A1	ssc-miR-19b
ENSSSCG00000001564	NR5A1	hsa-miR-590-5p_1ss19GA
ENSSSCG00000001564	NR5A1	ssc-miR-215_R+1
ENSSSCG00000001565	ENSSSCG0000000156 5	PC-5p-6765_300
ENSSSCG00000001565	ENSSSCG0000000156 5	ssc-miR-27b-5p_R-1
ENSSSCG00000001569	C6orf89	ssc-miR-1839-3p_R+2
ENSSSCG00000001573	PIM1	PC-5p-12443_142
ENSSSCG00000001577	ENSSSCG0000000157 7	hsa-miR-4792_1ss9GT
ENSSSCG00000001577	ENSSSCG0000000157 7	ssc-miR-192
ENSSSCG00000001577	ENSSSCG0000000157 7	ssc-miR-215_R+1
ENSSSCG00000001579	ENSSSCG0000000157 9	PC-3p-39317_30
ENSSSCG00000001579	ENSSSCG0000000157 9	ssc-miR-27b-5p_R-1
ENSSSCG00000001579	ENSSSCG0000000157 9	ssc-miR-424-5p_R-3
ENSSSCG00000001579	ENSSSCG0000000157 9	ssc-mir-4332-p5_1ss18CA
ENSSSCG00000021178	ENSSSCG0000002117 8	cfa-miR-8903_R-2_1ss21GA
ENSSSCG00000001596	NR5A1	PC-5p-9551_196
ENSSSCG00000001597	NR5A1	PC-5p-9551_196
ENSSSCG00000001597	NR5A1	hsa-miR-222-5p_L+2R-1
ENSSSCG00000001597	NR5A1	ssc-miR-19b
ENSSSCG00000001597	NR5A1	ssc-miR-339_R-2
ENSSSCG00000024348	NR5A1	PC-3p-21836_71
ENSSSCG00000024348	NR5A1	PC-5p-12443_142
ENSSSCG00000024348	NR5A1	hsa-miR-222-5p_L+2R-1
ENSSSCG00000024348	NR5A1	ssc-miR-339_R-2
ENSSSCG00000024348	NR5A1	ssc-miR-424-5p_R-3
ENSSSCG00000001611	NR5A1	PC-5p-6765_300
ENSSSCG00000001611	NR5A1	bta-mir-2478-p3_1ss20GA
ENSSSCG00000001611	NR5A1	hsa-miR-1246_L+1R+2
ENSSSCG00000001611	NR5A1	hsa-miR-873-3p_L-1_1ss11GA
ENSSSCG00000001616	TREML-2	PC-5p-9551_196
ENSSSCG00000001619	FOXP4	hsa-miR-23b-5p
ENSSSCG00000001620	MDFI	PC-5p-6765_300
ENSSSCG00000001620	MDFI	hsa-miR-23b-5p
ENSSSCG00000001620	MDFI	ssc-miR-339_R-2

ENSSSCG00000001639	NR5A1	ssc-miR-181d-5p
ENSSSCG00000001639	NR5A1	ssc-miR-1839-3p_R+2
ENSSSCG00000001639	NR5A1	ssc-miR-192
ENSSSCG00000001639	NR5A1	ssc-miR-215_R+1
ENSSSCG00000001646	GLTSCR1L	PC-3p-39317_30
ENSSSCG00000001646	GLTSCR1L	ssc-miR-192
ENSSSCG00000001646	GLTSCR1L	ssc-miR-215_R+1
ENSSSCG00000001651	CNPY3	hsa-miR-4792_1ss9GT
ENSSSCG00000001651	CNPY3	ssc-mir-4332-p5_1ss18CA
ENSSSCG00000001653	PEX6	cfa-miR-8903_R-2_1ss21GA
ENSSSCG00000001653	PEX6	mmu-let-7j_R-2
ENSSSCG00000001654	PPP2R5D	cfa-miR-8903_R-2_1ss21GA
ENSSSCG00000001654	PPP2R5D	hsa-miR-23b-5p
ENSSSCG00000001654	PPP2R5D	hsa-miR-873-3p_L-1_1ss11GA
ENSSSCG00000001654	PPP2R5D	ssc-miR-424-5p_R-3
ENSSSCG00000001656	RRP36	PC-3p-21836_71
ENSSSCG00000001656	RRP36	ssc-miR-1839-3p_R+2
ENSSSCG00000001660	PTK7	mmu-miR-107-5p
ENSSSCG00000001661	SRF	PC-3p-39317_30
ENSSSCG00000001661	SRF	PC-5p-9551_196
ENSSSCG00000001661	SRF	cfa-miR-8903_R-2_1ss21GA
ENSSSCG00000001667	ZNF318	hsa-miR-129-1-3p
ENSSSCG00000001667	ZNF318	hsa-miR-4792_1ss9GT
ENSSSCG00000001667	ZNF318	ssc-miR-1839-3p_R+2
ENSSSCG00000001667	ZNF318	ssc-miR-190b
ENSSSCG00000001667	ZNF318	ssc-miR-192
ENSSSCG00000001667	ZNF318	ssc-miR-215_R+1
ENSSSCG00000001667	ZNF318	ssc-miR-27b-5p_R-1
ENSSSCG00000001676	NR5A1	PC-3p-39317_30
ENSSSCG00000001683	POLH	ssc-miR-339_R-2
ENSSSCG00000001688	NR5A1	cfa-miR-8903_R-2_1ss21GA
ENSSSCG00000001688	NR5A1	hsa-miR-141-3p_R+1
ENSSSCG00000001695	ENSSSCG0000000169 5	ssc-miR-339_R-2
ENSSSCG00000001697	TMEM63B	ssc-miR-19b
ENSSSCG00000001697	TMEM63B	ssc-mir-4332-p5_1ss18CA
ENSSSCG00000001700	SLC29A1	PC-5p-12443_142
ENSSSCG00000001700	SLC29A1	ssc-miR-339_R-2
ENSSSCG00000001701	HSP90AB1	PC-3p-39317_30
ENSSSCG00000001701	HSP90AB1	ssc-miR-339_R-2
ENSSSCG00000001703	NFKBIE	PC-3p-39317_30
ENSSSCG00000001703	NFKBIE	PC-5p-9551_196
ENSSSCG00000001706	AARS2	PC-5p-9551_196
ENSSSCG00000001706	AARS2	hsa-miR-222-5p_L+2R-1

ENSSSCG00000001711	ENSSSCG00000001711	PC-5p-9551_196
ENSSSCG00000001711	ENSSSCG00000001711	cfa-miR-8903_R-2_1ss21GA
ENSSSCG00000001711	ENSSSCG00000001711	hsa-miR-141-3p_R+1
ENSSSCG00000001711	ENSSSCG00000001711	hsa-miR-23b-5p
ENSSSCG00000001715	ENPP5	PC-3p-21836_71
ENSSSCG00000001715	ENPP5	PC-5p-12443_142
ENSSSCG00000001715	ENPP5	PC-5p-9551_196
ENSSSCG00000001715	ENPP5	hsa-miR-141-3p_R+1
ENSSSCG00000001715	ENPP5	mmu-miR-107-5p
ENSSSCG00000001715	ENPP5	ssc-miR-181d-5p
ENSSSCG00000001715	ENPP5	ssc-miR-1839-3p_R+2
ENSSSCG00000001715	ENPP5	ssc-miR-19b
ENSSSCG00000001715	ENPP5	ssc-miR-424-5p_R-3
ENSSSCG00000001726	ADGRF1	PC-5p-12443_142
ENSSSCG00000001731	CENPQ	PC-5p-6765_300
ENSSSCG00000001731	CENPQ	ssc-miR-190b
ENSSSCG00000001732	ENSSSCG00000001732	bta-mir-2478-p3_1ss20GA
ENSSSCG00000001732	ENSSSCG00000001732	sha-miR-21_L+2R-2
ENSSSCG00000001732	ENSSSCG00000001732	ssc-miR-181d-5p
ENSSSCG00000001732	ENSSSCG00000001732	ssc-miR-424-5p_R-3
ENSSSCG00000001736	CRISP2	sha-miR-21_L+2R-2
ENSSSCG00000001741	DEFB114	ssc-miR-181d-5p
ENSSSCG00000001750	PAQR8	hsa-miR-23b-5p
ENSSSCG00000001752	CHRNA3	PC-3p-21836_71
ENSSSCG00000001752	CHRNA3	PC-5p-9551_196
ENSSSCG00000001752	CHRNA3	hsa-miR-1246_L+1R+2
ENSSSCG00000001752	CHRNA3	hsa-miR-222-5p_L+2R-1
ENSSSCG00000001752	CHRNA3	hsa-miR-4792_1ss9GT
ENSSSCG00000001754	PSMA4	PC-5p-6765_300
ENSSSCG00000001754	PSMA4	bta-mir-2478-p3_1ss20GA
ENSSSCG00000001754	PSMA4	hsa-miR-873-3p_L-1_1ss11GA
ENSSSCG00000001754	PSMA4	mmu-let-7j_R-2
ENSSSCG00000001759	DNAJA4	hsa-miR-129-1-3p
ENSSSCG00000001759	DNAJA4	hsa-miR-141-3p_R+1
ENSSSCG00000001759	DNAJA4	ssc-miR-181d-5p
ENSSSCG00000001760	ACSBG1	PC-5p-6765_300

ENSSSCG00000001760	ACSBG1	PC-5p-9551_196
ENSSSCG00000001760	ACSBG1	cfa-miR-8903_R-2_1ss21GA
ENSSSCG00000001760	ACSBG1	hsa-miR-141-3p_R+1
ENSSSCG00000001760	ACSBG1	hsa-miR-873-3p_L-1_1ss11GA
ENSSSCG00000001760	ACSBG1	mmu-miR-107-5p
ENSSSCG00000001760	ACSBG1	ssc-miR-192
ENSSSCG00000001760	ACSBG1	ssc-miR-19b
ENSSSCG00000001760	ACSBG1	ssc-miR-215_R+1
ENSSSCG00000001760	ACSBG1	ssc-miR-27b-5p_R-1
ENSSSCG00000001760	ACSBG1	ssc-miR-339_R-2
ENSSSCG00000001760	ACSBG1	ssc-miR-424-5p_R-3
ENSSSCG00000001761	IDH3A	hsa-miR-222-5p_L+2R-1
ENSSSCG00000001761	IDH3A	mmu-let-7j_R-2
ENSSSCG00000001761	IDH3A	ssc-miR-424-5p_R-3
ENSSSCG00000001762	CIB2	PC-5p-9551_196
ENSSSCG00000001765	ADAMTS7	PC-5p-12443_142
ENSSSCG00000001765	ADAMTS7	cfa-miR-8903_R-2_1ss21GA
ENSSSCG00000001765	ADAMTS7	ssc-miR-339_R-2
ENSSSCG00000001770	CTSH	mmu-miR-107-5p
ENSSSCG00000001770	CTSH	mmu-miR-107-5p
ENSSSCG00000001779	ZFAND6	mmu-miR-26a-2-3p_1ss4GA
ENSSSCG00000001779	ZFAND6	ssc-miR-181d-5p
ENSSSCG00000001782	ABHD17C	ssc-miR-19b
ENSSSCG00000001786	CFAP161	sha-miR-21_L+2R-2
ENSSSCG00000001788	NR5A1	PC-3p-21836_71
ENSSSCG00000001788	NR5A1	PC-5p-9551_196
ENSSSCG00000001788	NR5A1	ssc-miR-339_R-2
ENSSSCG00000001792	NR5A1	PC-5p-9551_196
ENSSSCG00000001804	HOMER2	hsa-miR-129-1-3p
ENSSSCG00000001805	WHAMM	PC-5p-6765_300
ENSSSCG00000001805	WHAMM	hsa-miR-4792_1ss9GT
ENSSSCG00000001805	WHAMM	ssc-miR-181d-5p
ENSSSCG00000001818	FES	PC-3p-39317_30
ENSSSCG00000001819	MAN2A2	PC-5p-9551_196
ENSSSCG00000001819	MAN2A2	bta-mir-2478-p3_1ss20GA
ENSSSCG00000001819	MAN2A2	hsa-miR-222-5p_L+2R-1
ENSSSCG00000001819	MAN2A2	mmu-miR-107-5p
ENSSSCG00000001819	MAN2A2	ssc-miR-1839-3p_R+2
ENSSSCG00000001819	MAN2A2	ssc-miR-424-5p_R-3
ENSSSCG00000001819	MAN2A2	ssc-mir-4332-p5_1ss18CA
ENSSSCG00000001820	HDDC3	hsa-miR-222-5p_L+2R-1
ENSSSCG00000001821	UNC45A	PC-5p-9551_196
ENSSSCG00000001823	UROC1	hsa-miR-129-1-3p
ENSSSCG00000001824	ZXDC	hsa-miR-222-5p_L+2R-1

ENSSSCG00000001824	ZXDC	hsa-miR-23b-5p
ENSSSCG00000001824	ZXDC	hsa-miR-4792_1ss9GT
ENSSSCG00000001834	MFGE8	PC-5p-9551_196
ENSSSCG00000001834	MFGE8	ssc-miR-339_R-2
ENSSSCG00000001836	RLBP1	hsa-miR-129-1-3p
ENSSSCG00000001841	RHCG	hsa-miR-23b-5p
ENSSSCG00000001859	GDPGP1	cfa-miR-8903_R-2_1ss21GA
ENSSSCG00000001859	GDPGP1	hsa-miR-222-5p_L+2R-1
ENSSSCG00000001859	GDPGP1	ssc-miR-1839-3p_R+2
ENSSSCG00000001863	ENSSSCG00000001863	PC-5p-9551_196
ENSSSCG00000001866	RCN2	hsa-miR-222-5p_L+2R-1
ENSSSCG00000001866	RCN2	mmu-let-7j_R-2
ENSSSCG00000001868	TSPAN3	PC-3p-39317_30
ENSSSCG00000001868	TSPAN3	hsa-miR-873-3p_L-1_1ss11GA
ENSSSCG00000001868	TSPAN3	ssc-miR-424-5p_R-3
ENSSSCG00000001871	ENSSSCG00000001871	hsa-miR-141-3p_R+1
ENSSSCG00000001877	SNUPN	hsa-miR-141-3p_R+1
ENSSSCG00000001878	PTPN9	PC-5p-6765_300
ENSSSCG00000001878	PTPN9	PC-5p-9551_196
ENSSSCG00000001878	PTPN9	hsa-miR-222-5p_L+2R-1
ENSSSCG00000001878	PTPN9	hsa-miR-23b-5p
ENSSSCG00000001878	PTPN9	hsa-miR-590-5p_1ss19GA
ENSSSCG00000001878	PTPN9	ssc-miR-181d-5p
ENSSSCG00000001878	PTPN9	ssc-miR-27b-5p_R-1
ENSSSCG00000001880	SIN3A	PC-5p-6765_300
ENSSSCG00000001881	MAN2C1	hsa-miR-23b-5p
ENSSSCG00000001884	COMMD4	PC-3p-39317_30
ENSSSCG00000001885	C15orf39	PC-3p-39317_30
ENSSSCG00000001885	C15orf39	hsa-miR-222-5p_L+2R-1
ENSSSCG00000001887	SCAMP5	PC-3p-39317_30
ENSSSCG00000001887	SCAMP5	PC-5p-9551_196
ENSSSCG00000001887	SCAMP5	ssc-miR-339_R-2
ENSSSCG00000001888	RPP25	ssc-miR-339_R-2
ENSSSCG00000001892	SCAMP2	PC-3p-21836_71
ENSSSCG00000001892	SCAMP2	bta-mir-2478-p3_1ss20GA
ENSSSCG00000001892	SCAMP2	ssc-miR-1839-3p_R+2
ENSSSCG00000001892	SCAMP2	ssc-miR-339_R-2
ENSSSCG00000001898	ULK3	hsa-miR-4792_1ss9GT
ENSSSCG00000001899	LMAN1L	PC-5p-12443_142
ENSSSCG00000001901	CYP1A2	hsa-miR-222-5p_L+2R-1
ENSSSCG00000001901	CYP1A2	hsa-miR-23b-5p
ENSSSCG00000001903	EDC3	PC-5p-9551_196

ENSSSCG00000001903	EDC3	hsa-miR-129-1-3p
ENSSSCG00000001903	EDC3	hsa-miR-31-3p_R+1
ENSSSCG00000001903	EDC3	ssc-miR-424-5p_R-3
ENSSSCG00000001904	CLK3	sha-miR-21_L+2R-2
ENSSSCG00000001908	CCDC33	cfa-miR-8903_R-2_1ss21GA
ENSSSCG00000001909	ENSSSCG00000001909	PC-5p-9551_196
ENSSSCG00000001910	ISLR	ssc-miR-424-5p_R-3
ENSSSCG00000001913	STOML1	cfa-miR-8903_R-2_1ss21GA
ENSSSCG00000001917	ENSSSCG00000001917	ssc-mir-4332-p5_1ss18CA
ENSSSCG00000001918	NPTN	PC-3p-21836_71
ENSSSCG00000001918	NPTN	hsa-miR-129-1-3p
ENSSSCG00000001918	NPTN	hsa-miR-141-3p_R+1
ENSSSCG00000001921	ENSSSCG00000001921	PC-5p-9551_196
ENSSSCG00000001921	ENSSSCG00000001921	hsa-miR-141-3p_R+1
ENSSSCG00000001921	ENSSSCG00000001921	hsa-miR-222-5p_L+2R-1
ENSSSCG00000001921	ENSSSCG00000001921	mmu-miR-26a-2-3p_1ss4GA
ENSSSCG00000001924	BBS4	PC-3p-39317_30
ENSSSCG00000001927	HEXA	bta-mir-2478-p3_1ss20GA
ENSSSCG00000001927	HEXA	ssc-miR-181d-5p
ENSSSCG00000001929	PARP6	hsa-miR-129-1-3p
ENSSSCG00000001929	PARP6	ssc-miR-339_R-2
ENSSSCG00000001929	PARP6	ssc-mir-4332-p5_1ss18CA
ENSSSCG00000001930	PKM	PC-5p-9551_196
ENSSSCG00000001930	PKM	hsa-miR-1246_L+1R+2
ENSSSCG00000001931	ENSSSCG00000001931	ssc-miR-424-5p_R-3
ENSSSCG00000001931	ENSSSCG00000001931	ssc-miR-424-5p_R-3
ENSSSCG00000001942	SLC25A21	PC-3p-21836_71
ENSSSCG00000001942	SLC25A21	PC-5p-9551_196
ENSSSCG00000001942	SLC25A21	ssc-miR-1839-3p_R+2
ENSSSCG00000001942	SLC25A21	ssc-miR-192
ENSSSCG00000001942	SLC25A21	ssc-miR-215_R+1
ENSSSCG00000001943	PAX9	PC-3p-21836_71
ENSSSCG00000001943	PAX9	hsa-miR-1246_L+1R+2
ENSSSCG00000001943	PAX9	hsa-miR-129-1-3p
ENSSSCG00000001943	PAX9	mmu-let-7j_R-2
ENSSSCG00000001943	PAX9	ssc-miR-181d-5p

ENSSSCG00000001943	PAX9	ssc-miR-192
ENSSSCG00000001943	PAX9	ssc-miR-215_R+1
ENSSSCG00000001945	NKX2-1	hsa-miR-873-3p_L-1_1ss11GA
ENSSSCG00000001945	NKX2-1	mmu-miR-107-5p
ENSSSCG00000001947	BRMS1L	hsa-miR-141-3p_R+1
ENSSSCG00000001947	BRMS1L	hsa-miR-590-5p_1ss19GA
ENSSSCG00000001947	BRMS1L	sha-miR-21_L+2R-2
ENSSSCG00000001947	BRMS1L	ssc-miR-19b
ENSSSCG00000001947	BRMS1L	ssc-miR-339_R-2
ENSSSCG00000001955	SRP54	bta-mir-2478-p3_1ss20GA
ENSSSCG00000001959	CFL2	PC-5p-6765_300
ENSSSCG00000001959	CFL2	hsa-miR-141-3p_R+1
ENSSSCG00000001959	CFL2	hsa-miR-222-5p_L+2R-1
ENSSSCG00000001959	CFL2	mmu-miR-107-5p
ENSSSCG00000001959	CFL2	ssc-miR-181d-5p
ENSSSCG00000001959	CFL2	ssc-miR-19b
ENSSSCG00000001960	EAPP	hsa-miR-141-3p_R+1
ENSSSCG00000001963	EGLN3	PC-5p-9551_196
ENSSSCG00000001963	EGLN3	hsa-miR-129-1-3p
ENSSSCG00000001963	EGLN3	ssc-miR-19b
ENSSSCG00000001963	EGLN3	ssc-miR-339_R-2
ENSSSCG00000001970	HEATR5A	mmu-miR-26a-2-3p_1ss4GA
ENSSSCG00000001974	G2E3	hsa-miR-590-5p_1ss19GA
ENSSSCG00000001974	G2E3	sha-miR-21_L+2R-2
ENSSSCG00000001984	KHNYN	hsa-miR-1246_L+1R+2
ENSSSCG00000001985	CBLN3	PC-3p-39317_30
ENSSSCG00000001985	CBLN3	PC-5p-9551_196
ENSSSCG00000001985	CBLN3	hsa-miR-222-5p_L+2R-1
ENSSSCG00000001985	CBLN3	ssc-miR-181d-5p
ENSSSCG00000001986	NFATC4	PC-3p-39317_30
ENSSSCG00000001989	CIDEB	ssc-miR-424-5p_R-3
ENSSSCG00000001989	CIDEB	hsa-miR-222-5p_L+2R-1
ENSSSCG00000001989	CIDEB	ssc-miR-424-5p_R-3
ENSSSCG00000001990	NOP9	PC-5p-6765_300
ENSSSCG00000001990	NOP9	hsa-miR-141-3p_R+1
ENSSSCG00000001990	NOP9	hsa-miR-222-5p_L+2R-1
ENSSSCG00000001993	TGM1	ssc-miR-339_R-2
ENSSSCG00000002001	REC8	PC-3p-39317_30
ENSSSCG00000002001	REC8	hsa-miR-129-1-3p
ENSSSCG00000002004	PSME2	bta-mir-2478-p3_1ss20GA
ENSSSCG00000002005	EMC9	PC-3p-39317_30
ENSSSCG00000002005	EMC9	bta-mir-2478-p3_1ss20GA
ENSSSCG00000002005	EMC9	hsa-miR-4792_1ss9GT
ENSSSCG00000002008	DCAF11	PC-3p-21836_71

ENSSSCG00000002008	DCAF11	PC-5p-9551_196
ENSSSCG00000002008	DCAF11	hsa-miR-222-5p_L+2R-1
ENSSSCG00000002008	DCAF11	ssc-miR-1839-3p_R+2
ENSSSCG00000002014	JPH4	PC-3p-39317_30
ENSSSCG00000002014	JPH4	hsa-miR-129-1-3p
ENSSSCG00000002016	THTPA	PC-3p-39317_30
ENSSSCG00000002016	THTPA	PC-5p-12443_142
ENSSSCG00000002016	THTPA	hsa-miR-4792_1ss9GT
ENSSSCG00000002025	SLC22A17	hsa-miR-23b-5p
ENSSSCG00000002025	SLC22A17	ssc-miR-339_R-2
ENSSSCG00000002034	ACIN1	hsa-miR-4792_1ss9GT
ENSSSCG00000002036	NR5A1	ssc-miR-27b-5p_R-1
ENSSSCG00000002039	NR5A1	PC-3p-21836_71
ENSSSCG00000002039	NR5A1	hsa-miR-141-3p_R+1
ENSSSCG00000002040	NR5A1	PC-5p-9551_196
ENSSSCG00000002041	NR5A1	PC-3p-21836_71
ENSSSCG00000002041	NR5A1	bta-mir-2478-p3_1ss20GA
ENSSSCG00000002041	NR5A1	hsa-miR-4792_1ss9GT
ENSSSCG00000002041	NR5A1	mmu-let-7j_R-2
ENSSSCG00000002041	NR5A1	ssc-miR-181d-5p
ENSSSCG00000002051	ABHD4	PC-3p-39317_30
ENSSSCG00000002081	ENSSSCG0000000208 1	hsa-miR-222-5p_L+2R-1
ENSSSCG00000002131	ENSSSCG0000000213 1	ssc-miR-339_R-2
ENSSSCG00000002136	TMEM55B	PC-3p-39317_30
ENSSSCG00000002136	TMEM55B	PC-5p-9551_196
ENSSSCG00000002136	TMEM55B	ssc-miR-424-5p_R-3
ENSSSCG00000002145	TTC5	hsa-miR-141-3p_R+1
ENSSSCG00000002145	TTC5	sha-mir-24-1-p3_1ss2GC
ENSSSCG00000002245	KATNBL1	PC-5p-6765_300
ENSSSCG00000002251	ENSSSCG0000000225 1	bta-mir-2478-p3_1ss20GA
ENSSSCG00000002252	ARRDC4	mmu-miR-107-5p
ENSSSCG00000002252	ARRDC4	ssc-miR-181d-5p
ENSSSCG00000002252	ARRDC4	ssc-miR-19b
ENSSSCG00000002252	ARRDC4	ssc-miR-424-5p_R-3
ENSSSCG00000002259	ENSSSCG0000000225 9	PC-3p-39317_30
ENSSSCG00000002259	ENSSSCG0000000225 9	hsa-miR-222-5p_L+2R-1
ENSSSCG00000002262	SV2B	PC-5p-12443_142
ENSSSCG00000002262	SV2B	hsa-miR-4792_1ss9GT
ENSSSCG00000002262	SV2B	ssc-miR-424-5p_R-3

ENSSSCG00000002265	FAM174B	PC-5p-9551_196
ENSSSCG00000002265	FAM174B	hsa-miR-4792_1ss9GT
ENSSSCG00000002265	FAM174B	ssc-miR-339_R-2
ENSSSCG00000002269	MTHFD1	bta-mir-2478-p3_1ss20GA
ENSSSCG00000002269	MTHFD1	hsa-miR-873-3p_L-1_1ss11GA
ENSSSCG00000002269	MTHFD1	mmu-miR-107-5p
ENSSSCG00000002274	HSPA2	ssc-miR-19b
ENSSSCG00000002275	PPP1R36	ssc-miR-27b-5p_R-1
ENSSSCG00000002276	PLEKHG3	PC-3p-39317_30
ENSSSCG00000002276	PLEKHG3	hsa-miR-4792_1ss9GT
ENSSSCG00000002276	PLEKHG3	mmu-miR-107-5p
ENSSSCG00000002276	PLEKHG3	ssc-miR-27b-5p_R-1
ENSSSCG00000002277	SPTB	PC-3p-39317_30
ENSSSCG00000002277	SPTB	PC-5p-9551_196
ENSSSCG00000002279	GPX2	hsa-miR-222-5p_L+2R-1
ENSSSCG00000002281	CHURC1-FNTB	PC-3p-21836_71
ENSSSCG00000002281	CHURC1-FNTB	hsa-miR-873-3p_L-1_1ss11GA
ENSSSCG00000002281	CHURC1-FNTB	ssc-miR-181d-5p
ENSSSCG00000002281	CHURC1-FNTB	ssc-miR-19b
ENSSSCG00000002283	FUT8	hsa-miR-129-1-3p
ENSSSCG00000002285	GPHN	hsa-miR-141-3p_R+1
ENSSSCG00000002285	GPHN	ssc-miR-190b
ENSSSCG00000002285	GPHN	ssc-mir-4332-p5_1ss18CA
ENSSSCG00000002288	ATP6V1D	PC-5p-9551_196
ENSSSCG00000002288	ATP6V1D	hsa-miR-141-3p_R+1
ENSSSCG00000002288	ATP6V1D	hsa-miR-590-5p_1ss19GA
ENSSSCG00000002288	ATP6V1D	ssc-miR-190b
ENSSSCG00000002288	ATP6V1D	ssc-miR-424-5p_R-3
ENSSSCG00000002290	PLEK2	hsa-miR-1246_L+1R+2
ENSSSCG00000002290	PLEK2	hsa-miR-222-5p_L+2R-1
ENSSSCG00000002290	PLEK2	mmu-miR-107-5p
ENSSSCG00000002290	PLEK2	ssc-miR-215_R+1
ENSSSCG00000002292	PLEKHH1	PC-3p-21836_71
ENSSSCG00000002292	PLEKHH1	hsa-miR-222-5p_L+2R-1
ENSSSCG00000002292	PLEKHH1	hsa-miR-23b-5p
ENSSSCG00000002292	PLEKHH1	mmu-let-7j_R-2
ENSSSCG00000002292	PLEKHH1	ssc-miR-192
ENSSSCG00000002292	PLEKHH1	ssc-miR-19b
ENSSSCG00000002292	PLEKHH1	ssc-miR-215_R+1
ENSSSCG00000002292	PLEKHH1	ssc-miR-339_R-2
ENSSSCG00000002294	ARG2	PC-3p-39317_30
ENSSSCG00000002296	RDH11	PC-3p-21836_71
ENSSSCG00000002296	RDH11	mmu-miR-107-5p
ENSSSCG00000002296	RDH11	ssc-miR-424-5p_R-3

ENSSSCG00000002297	RDH12	PC-5p-12443_142
ENSSSCG00000002297	RDH12	cfa-miR-8903_R-2_1ss21GA
ENSSSCG00000002297	RDH12	hsa-miR-141-3p_R+1
ENSSSCG00000002297	RDH12	hsa-miR-23b-5p
ENSSSCG00000002297	RDH12	mmu-miR-26a-2-3p_1ss4GA
ENSSSCG00000002297	RDH12	ssc-miR-1839-3p_R+2
ENSSSCG00000002297	RDH12	ssc-miR-424-5p_R-3
ENSSSCG00000002298	ZFYVE26	PC-5p-6765_300
ENSSSCG00000002298	ZFYVE26	hsa-miR-375
ENSSSCG00000002298	ZFYVE26	ssc-miR-181d-5p
ENSSSCG00000002298	ZFYVE26	ssc-miR-192
ENSSSCG00000002298	ZFYVE26	ssc-miR-19b
ENSSSCG00000002298	ZFYVE26	ssc-miR-215_R+1
ENSSSCG00000002305	EXD2	PC-3p-39317_30
ENSSSCG00000002305	EXD2	hsa-miR-1246_L+1R+2
ENSSSCG00000002305	EXD2	ssc-miR-27b-5p_R-1
ENSSSCG00000002306	GALNT16	PC-3p-39317_30
ENSSSCG00000002306	GALNT16	hsa-miR-4792_1ss9GT
ENSSSCG00000002306	GALNT16	ssc-miR-339_R-2
ENSSSCG00000002311	SUSD6	PC-5p-9551_196
ENSSSCG00000002311	SUSD6	bta-mir-2478-p3_1ss20GA
ENSSSCG00000002311	SUSD6	hsa-miR-222-5p_L+2R-1
ENSSSCG00000002311	SUSD6	mmu-miR-107-5p
ENSSSCG00000002311	SUSD6	ssc-miR-181d-5p
ENSSSCG00000002315	SLC8A3	cfa-miR-8903_R-2_1ss21GA
ENSSSCG00000002315	SLC8A3	hsa-miR-23b-5p
ENSSSCG00000002315	SLC8A3	ssc-miR-339_R-2
ENSSSCG00000002316	COX16	hsa-miR-1246_L+1R+2
ENSSSCG00000002330	PCNX1	PC-3p-21836_71
ENSSSCG00000002330	PCNX1	hsa-miR-1246_L+1R+2
ENSSSCG00000002330	PCNX1	hsa-miR-873-3p_L-1_1ss11GA
ENSSSCG00000002330	PCNX1	ssc-miR-424-5p_R-3
ENSSSCG00000002332	SIPA1L1	PC-3p-21836_71
ENSSSCG00000002332	SIPA1L1	PC-5p-9551_196
ENSSSCG00000002332	SIPA1L1	ssc-miR-181d-5p
ENSSSCG00000002337	ZFYVE1	ssc-miR-424-5p_R-3
ENSSSCG00000002340	PSEN1	PC-5p-6765_300
ENSSSCG00000002345	ENSSSCG0000000234 5	PC-5p-6765_300
ENSSSCG00000002345	ENSSSCG0000000234 5	cfa-miR-8903_R-2_1ss21GA
ENSSSCG00000002345	ENSSSCG0000000234 5	hsa-miR-129-1-3p

ENSSSCG00000002345	ENSSSCG00000002345	ssc-miR-181d-5p
ENSSSCG00000002345	ENSSSCG00000002345	ssc-miR-190b
ENSSSCG00000002345	ENSSSCG00000002345	ssc-miR-424-5p_R-3
ENSSSCG00000002348	ENSSSCG00000002348	hsa-miR-590-5p_1ss19GA
ENSSSCG00000002349	ACOT4	PC-3p-21836_71
ENSSSCG00000002349	ACOT4	ssc-miR-190b
ENSSSCG00000002351	ENSSSCG00000002351	ssc-miR-19b
ENSSSCG00000002352	ZNF410	PC-3p-21836_71
ENSSSCG00000002352	ZNF410	PC-5p-9551_196
ENSSSCG00000002352	ZNF410	bta-mir-2478-p3_1ss20GA
ENSSSCG00000002352	ZNF410	hsa-miR-222-5p_L+2R-1
ENSSSCG00000002352	ZNF410	hsa-miR-4792_1ss9GT
ENSSSCG00000002352	ZNF410	hsa-miR-873-3p_L-1_1ss11GA
ENSSSCG00000002353	FAM161B	PC-3p-21836_71
ENSSSCG00000002353	FAM161B	bta-mir-2478-p3_1ss20GA
ENSSSCG00000002353	FAM161B	cfa-miR-8903_R-2_1ss21GA
ENSSSCG00000002353	FAM161B	hsa-miR-141-3p_R+1
ENSSSCG00000002353	FAM161B	hsa-miR-873-3p_L-1_1ss11GA
ENSSSCG00000002353	FAM161B	mmu-miR-107-5p
ENSSSCG00000002354	COQ6	hsa-miR-873-3p_L-1_1ss11GA
ENSSSCG00000002354	COQ6	ssc-miR-1839-3p_R+2
ENSSSCG00000002359	ENSSSCG00000002359	PC-3p-21836_71
ENSSSCG00000002361	VRTN	hsa-miR-129-1-3p
ENSSSCG00000002361	VRTN	hsa-miR-23b-5p
ENSSSCG00000002370	AREL1	ssc-miR-181d-5p
ENSSSCG00000002370	AREL1	ssc-mir-4332-p5_1ss18CA
ENSSSCG00000002374	DLST	PC-5p-9551_196
ENSSSCG00000002381	NEK9	hsa-miR-31-3p_R+1
ENSSSCG00000002381	NEK9	hsa-miR-4792_1ss9GT
ENSSSCG00000002381	NEK9	ssc-miR-181d-5p
ENSSSCG00000002381	NEK9	ssc-miR-424-5p_R-3
ENSSSCG00000002389	VASH1	PC-3p-39317_30
ENSSSCG00000002389	VASH1	PC-5p-9551_196
ENSSSCG00000002389	VASH1	mmu-miR-107-5p
ENSSSCG00000002396	GSTZ1	cfa-miR-8903_R-2_1ss21GA
ENSSSCG00000002396	GSTZ1	ssc-miR-181d-5p
ENSSSCG00000002403	VIPAS39	PC-3p-21836_71
ENSSSCG00000002403	VIPAS39	hsa-miR-222-5p_L+2R-1

ENSSSCG00000002404	SPTLC2	PC-5p-9551_196
ENSSSCG00000002404	SPTLC2	hsa-miR-1246_L+1R+2
ENSSSCG00000002404	SPTLC2	mmu-miR-26a-2-3p_1ss4GA
ENSSSCG00000002404	SPTLC2	sha-miR-21_L+2R-2
ENSSSCG00000002411	GTF2A1	PC-3p-21836_71
ENSSSCG00000002411	GTF2A1	mmu-let-7j_R-2
ENSSSCG00000002411	GTF2A1	ssc-miR-19b
ENSSSCG00000002411	GTF2A1	ssc-miR-424-5p_R-3
ENSSSCG00000002427	EML5	hsa-miR-141-3p_R+1
ENSSSCG00000002427	EML5	mmu-miR-107-5p
ENSSSCG00000002431	ENSSSCG00000002431	mmu-miR-26a-2-3p_1ss4GA
ENSSSCG00000002432	KCNK13	PC-3p-39317_30
ENSSSCG00000002432	KCNK13	hsa-miR-873-3p_L-1_1ss11GA
ENSSSCG00000002432	KCNK13	ssc-miR-424-5p_R-3
ENSSSCG00000002436	ENSSSCG00000002436	mmu-miR-107-5p
ENSSSCG00000002441	PPP4R3A	PC-5p-6765_300
ENSSSCG00000002444	FBLN5	PC-5p-9551_196
ENSSSCG00000002446	ENSSSCG00000002446	PC-3p-21836_71
ENSSSCG00000002446	ENSSSCG00000002446	bta-mir-2478-p3_1ss20GA
ENSSSCG00000002446	ENSSSCG00000002446	hsa-miR-31-3p_R+1
ENSSSCG00000002446	ENSSSCG00000002446	hsa-miR-590-5p_1ss19GA
ENSSSCG00000002446	ENSSSCG00000002446	ssc-miR-181d-5p
ENSSSCG00000002448	ENSSSCG00000002448	PC-3p-21836_71
ENSSSCG00000002448	ENSSSCG00000002448	cfa-miR-8903_R-2_1ss21GA
ENSSSCG00000002448	ENSSSCG00000002448	hsa-miR-129-1-3p
ENSSSCG00000002448	ENSSSCG00000002448	ssc-miR-27b-5p_R-1
ENSSSCG00000002451	ENSSSCG00000002451	hsa-miR-222-5p_L+2R-1
ENSSSCG00000002452	ENSSSCG00000002452	PC-5p-9551_196
ENSSSCG00000002452	ENSSSCG00000002452	hsa-miR-4792_1ss9GT

ENSSSCG00000002452	ENSSSCG00000002452	ssc-miR-192
ENSSSCG00000002452	ENSSSCG00000002452	ssc-miR-215_R+1
ENSSSCG00000002456	CHGA	PC-3p-39317_30
ENSSSCG00000002458	TMEM251	PC-5p-12443_142
ENSSSCG00000002460	ENSSSCG00000002460	PC-3p-39317_30
ENSSSCG00000002460	ENSSSCG00000002460	hsa-miR-141-3p_R+1
ENSSSCG00000002461	BTBD7	hsa-miR-129-1-3p
ENSSSCG00000002461	BTBD7	hsa-miR-222-5p_L+2R-1
ENSSSCG00000002461	BTBD7	hsa-miR-23b-5p
ENSSSCG00000002461	BTBD7	hsa-miR-873-3p_L-1_1ss11GA
ENSSSCG00000002461	BTBD7	ssc-miR-19b
ENSSSCG00000002469	OTUB2	PC-5p-12443_142
ENSSSCG00000002470	ENSSSCG00000002470	hsa-miR-141-3p_R+1
ENSSSCG00000002474	PPP4R4	PC-3p-21836_71
ENSSSCG00000002474	PPP4R4	hsa-miR-141-3p_R+1
ENSSSCG00000002479	ENSSSCG00000002479	PC-3p-39317_30
ENSSSCG00000002481	SERPINA5	PC-3p-39317_30
ENSSSCG00000002481	SERPINA5	bta-mir-2478-p3_1ss20GA
ENSSSCG00000002481	SERPINA5	hsa-miR-222-5p_L+2R-1
ENSSSCG00000002483	ENSSSCG00000002483	hsa-miR-4792_1ss9GT
ENSSSCG00000030371	ENSSSCG00000030371	hsa-miR-4792_1ss9GT
ENSSSCG00000002494	CLMN	PC-5p-6765_300
ENSSSCG00000002494	CLMN	cfa-miR-8903_R-2_1ss21GA
ENSSSCG00000002494	CLMN	hsa-miR-23b-5p
ENSSSCG00000002494	CLMN	hsa-miR-4792_1ss9GT
ENSSSCG00000002494	CLMN	mmu-miR-107-5p
ENSSSCG00000002494	CLMN	mmu-miR-26a-2-3p_1ss4GA
ENSSSCG00000002494	CLMN	ssc-miR-1839-3p_R+2
ENSSSCG00000002496	GLRX5	PC-5p-9551_196
ENSSSCG00000002496	GLRX5	hsa-miR-873-3p_L-1_1ss11GA
ENSSSCG00000002496	GLRX5	mmu-let-7j_R-2
ENSSSCG00000002496	GLRX5	ssc-miR-1839-3p_R+2
ENSSSCG00000002497	TCL1B	ssc-miR-424-5p_R-3
ENSSSCG00000002509	CCNK	PC-5p-9551_196
ENSSSCG00000002509	CCNK	hsa-miR-222-5p_L+2R-1
ENSSSCG00000002509	CCNK	ssc-miR-339_R-2

ENSSSCG00000002510	CYP46A1	ssc-miR-181d-5p
ENSSSCG00000002511	ENSSSCG00000002511	PC-3p-39317_30
ENSSSCG00000002511	ENSSSCG00000002511	cfa-miR-8903_R-2_1ss21GA
ENSSSCG00000002511	ENSSSCG00000002511	hsa-miR-129-1-3p
ENSSSCG00000002511	ENSSSCG00000002511	hsa-miR-31-3p_R+1
ENSSSCG00000002520	SLC25A29	ssc-miR-19b
ENSSSCG00000002523	CDC42BPB	ssc-miR-339_R-2
ENSSSCG00000002525	TRAF3	ssc-mir-4332-p5_1ss18CA
ENSSSCG00000002544	PPP1R13B	PC-5p-9551_196
ENSSSCG00000002554	PLD4	PC-5p-9551_196
ENSSSCG00000002622	TMEM14A	PC-3p-21836_71
ENSSSCG00000002623	ENSSSCG00000002623	hsa-miR-873-3p_L-1_1ss11GA
ENSSSCG00000002627	GSTA4	PC-5p-12443_142
ENSSSCG00000002627	GSTA4	ssc-miR-424-5p_R-3
ENSSSCG00000002628	ICK	mmu-let-7j_R-2
ENSSSCG00000002629	FBXO9	hsa-miR-31-3p_R+1
ENSSSCG00000002633	NR5A1	ssc-miR-339_R-2
ENSSSCG00000002633	NR5A1	ssc-mir-4332-p5_1ss18CA
ENSSSCG00000002637	NR5A1	PC-5p-9551_196
ENSSSCG00000002637	NR5A1	hsa-miR-4792_1ss9GT
ENSSSCG00000002637	NR5A1	ssc-miR-339_R-2
ENSSSCG00000002648	CBFA2T3	bta-mir-2478-p3_1ss20GA
ENSSSCG00000002648	CBFA2T3	ssc-miR-181d-5p
ENSSSCG00000002648	CBFA2T3	ssc-miR-424-5p_R-3
ENSSSCG00000002651	CDT1	PC-3p-39317_30
ENSSSCG00000002655	MAP1LC3B	ssc-miR-192
ENSSSCG00000002655	MAP1LC3B	ssc-miR-215_R+1
ENSSSCG00000002655	MAP1LC3B	ssc-miR-339_R-2
ENSSSCG00000002667	ZDHHC7	hsa-miR-222-5p_L+2R-1
ENSSSCG00000002667	ZDHHC7	mmu-miR-107-5p
ENSSSCG00000002667	ZDHHC7	ssc-miR-181d-5p
ENSSSCG00000002667	ZDHHC7	ssc-mir-4332-p5_1ss18CA
ENSSSCG00000002670	ENSSSCG00000002670	PC-3p-21836_71
ENSSSCG00000002671	ATP2C2	ssc-mir-4332-p5_1ss18CA
ENSSSCG00000002681	HSDL1	PC-5p-12443_142
ENSSSCG00000002681	HSDL1	bta-mir-2478-p3_1ss20GA
ENSSSCG00000002681	HSDL1	ssc-miR-181d-5p
ENSSSCG00000002681	HSDL1	ssc-miR-339_R-2

ENSSSCG00000002682	MBTPS1	PC-3p-39317_30
ENSSSCG00000002682	MBTPS1	PC-5p-9551_196
ENSSSCG00000002682	MBTPS1	mmu-miR-107-5p
ENSSSCG00000002682	MBTPS1	sha-miR-21_L+2R-2
ENSSSCG00000002682	MBTPS1	ssc-miR-424-5p_R-3
ENSSSCG00000002689	CMIP	hsa-miR-1246_L+1R+2
ENSSSCG00000002689	CMIP	hsa-miR-873-3p_L-1_1ss11GA
ENSSSCG00000002689	CMIP	mmu-miR-107-5p
ENSSSCG00000002690	GAN	bta-mir-2478-p3_1ss20GA
ENSSSCG00000002690	GAN	hsa-miR-141-3p_R+1
ENSSSCG00000002690	GAN	ssc-miR-190b
ENSSSCG00000002696	VAT1L	cfa-miR-8903_R-2_1ss21GA
ENSSSCG00000002696	VAT1L	hsa-miR-31-3p_R+1
ENSSSCG00000002696	VAT1L	mmu-miR-26a-2-3p_1ss4GA
ENSSSCG00000002696	VAT1L	ssc-miR-181d-5p
ENSSSCG00000002697	ENSSSCG00000002697	hsa-miR-141-3p_R+1
ENSSSCG00000002703	TERF2IP	hsa-miR-873-3p_L-1_1ss11GA
ENSSSCG00000002703	TERF2IP	ssc-miR-181d-5p
ENSSSCG00000002703	TERF2IP	ssc-miR-424-5p_R-3
ENSSSCG00000002704	KARS	cfa-miR-8903_R-2_1ss21GA
ENSSSCG00000002707	GABARAPL2	PC-5p-9551_196
ENSSSCG00000002707	GABARAPL2	hsa-miR-222-5p_L+2R-1
ENSSSCG00000002708	NR5A1	PC-3p-21836_71
ENSSSCG00000002708	NR5A1	PC-5p-6765_300
ENSSSCG00000002708	NR5A1	mmu-miR-107-5p
ENSSSCG00000002708	NR5A1	ssc-miR-192
ENSSSCG00000002708	NR5A1	ssc-miR-215_R+1
ENSSSCG00000002712	NR5A1	PC-3p-39317_30
ENSSSCG00000002715	RFWD3	PC-5p-12443_142
ENSSSCG00000002715	RFWD3	hsa-miR-1246_L+1R+2
ENSSSCG00000002718	FA2H	PC-3p-39317_30
ENSSSCG00000002718	FA2H	mmu-miR-107-5p
ENSSSCG00000002720	CLEC18A	PC-3p-39317_30
ENSSSCG00000002720	CLEC18A	hsa-miR-4792_1ss9GT
ENSSSCG00000002720	CLEC18A	ssc-miR-339_R-2
ENSSSCG00000002721	ENSSSCG00000002721	PC-3p-21836_71
ENSSSCG00000002721	ENSSSCG00000002721	PC-3p-39317_30
ENSSSCG00000002721	ENSSSCG00000002721	hsa-miR-129-1-3p
ENSSSCG00000002721	ENSSSCG00000002721	ssc-miR-424-5p_R-3

ENSSSCG00000002725	ENSSSCG00000002725	hsa-miR-141-3p_R+1
ENSSSCG00000002734	MARVELD3	hsa-miR-141-3p_R+1
ENSSSCG00000002734	MARVELD3	mmu-let-7j_R-2
ENSSSCG00000002736	TAT	mmu-let-7j_R-2
ENSSSCG00000002736	TAT	ssc-mir-4332-p5_1ss18CA
ENSSSCG00000002739	ENSSSCG00000002739	hsa-miR-590-5p_1ss19GA
ENSSSCG00000002740	AP1G1	ssc-miR-181d-5p
ENSSSCG00000002740	AP1G1	ssc-miR-424-5p_R-3
ENSSSCG00000002743	ENSSSCG00000002743	ssc-miR-19b
ENSSSCG00000002748	ENSSSCG00000002748	bta-mir-2478-p3_1ss20GA
ENSSSCG00000002753	NOB1	cfa-miR-8903_R-2_1ss21GA
ENSSSCG00000002754	NQO1	PC-3p-21836_71
ENSSSCG00000002754	NQO1	mmu-let-7j_R-2
ENSSSCG00000002759	ENSSSCG00000002759	ssc-mir-4332-p5_1ss18CA
ENSSSCG00000002760	NIP7	mmu-miR-26a-2-3p_1ss4GA
ENSSSCG00000002776	ATP6V0D1	PC-5p-6765_300
ENSSSCG00000002776	ATP6V0D1	cfa-miR-8903_R-2_1ss21GA
ENSSSCG00000002776	ATP6V0D1	hsa-miR-23b-5p
ENSSSCG00000002776	ATP6V0D1	hsa-miR-4792_1ss9GT
ENSSSCG00000002780	TPPP3	PC-5p-12443_142
ENSSSCG00000002780	TPPP3	ssc-miR-424-5p_R-3
ENSSSCG00000002795	NR5A1	mmu-miR-107-5p
ENSSSCG00000002795	NR5A1	ssc-miR-192
ENSSSCG00000002795	NR5A1	ssc-miR-19b
ENSSSCG00000002795	NR5A1	ssc-miR-215_R+1
ENSSSCG00000002795	NR5A1	ssc-miR-27b-5p_R-1
ENSSSCG00000002795	NR5A1	ssc-miR-424-5p_R-3
ENSSSCG00000002799	CNOT1	PC-5p-12443_142
ENSSSCG00000002799	CNOT1	mmu-miR-107-5p
ENSSSCG00000002799	CNOT1	mmu-miR-26a-2-3p_1ss4GA
ENSSSCG00000002799	CNOT1	sha-miR-21_L+2R-2
ENSSSCG00000002800	ENSSSCG00000002800	PC-5p-9551_196
ENSSSCG00000002800	ENSSSCG00000002800	bta-mir-2478-p3_1ss20GA
ENSSSCG00000002800	ENSSSCG00000002800	cfa-miR-8903_R-2_1ss21GA
ENSSSCG00000002800	ENSSSCG00000002800	hsa-miR-222-5p_L+2R-1

ENSSSCG00000002800	ENSSSCG00000002800	mmu-miR-107-5p
ENSSSCG00000002802	GINS3	PC-5p-9551_196
ENSSSCG00000002802	GINS3	hsa-miR-1246_L+1R+2
ENSSSCG00000002802	GINS3	hsa-miR-873-3p_L-1_1ss11GA
ENSSSCG00000002804	CSNK2A2	PC-5p-9551_196
ENSSSCG00000002804	CSNK2A2	hsa-miR-222-5p_L+2R-1
ENSSSCG00000002804	CSNK2A2	ssc-mir-4332-p5_1ss18CA
ENSSSCG00000002806	MMP15	cfa-miR-8903_R-2_1ss21GA
ENSSSCG00000002806	MMP15	ssc-miR-339_R-2
ENSSSCG00000002807	ENSSSCG00000002807	ssc-miR-339_R-2
ENSSSCG00000002807	ENSSSCG00000002807	ssc-miR-424-5p_R-3
ENSSSCG00000002813	KATNB1	hsa-miR-222-5p_L+2R-1
ENSSSCG00000002813	KATNB1	ssc-miR-424-5p_R-3
ENSSSCG00000002814	ADGRG3	cfa-miR-8903_R-2_1ss21GA
ENSSSCG00000002814	ADGRG3	hsa-miR-4792_1ss9GT
ENSSSCG00000002818	PLLP	hsa-miR-4792_1ss9GT
ENSSSCG00000002819	ARL2BP	PC-3p-39317_30
ENSSSCG00000002819	ARL2BP	hsa-miR-23b-5p
ENSSSCG00000002819	ARL2BP	hsa-miR-873-3p_L-1_1ss11GA
ENSSSCG00000002819	ARL2BP	ssc-miR-424-5p_R-3
ENSSSCG00000002822	ENSSSCG00000002822	PC-5p-9551_196
ENSSSCG00000002822	ENSSSCG00000002822	hsa-miR-31-3p_R+1
ENSSSCG00000002828	LPCAT2	PC-5p-6765_300
ENSSSCG00000002828	LPCAT2	sha-miR-21_L+2R-2
ENSSSCG00000002828	LPCAT2	ssc-miR-181d-5p
ENSSSCG00000002830	IRX6	hsa-miR-222-5p_L+2R-1
ENSSSCG00000002830	IRX6	hsa-miR-23b-5p
ENSSSCG00000002830	IRX6	hsa-miR-4792_1ss9GT
ENSSSCG00000002841	N4BP1	PC-3p-21836_71
ENSSSCG00000002841	N4BP1	hsa-miR-129-1-3p
ENSSSCG00000002841	N4BP1	ssc-miR-424-5p_R-3
ENSSSCG00000002844	PHKB	hsa-miR-141-3p_R+1
ENSSSCG00000002854	POP4	bta-mir-2478-p3_1ss20GA
ENSSSCG00000002854	POP4	ssc-miR-1839-3p_R+2
ENSSSCG00000002859	ANKRD27	hsa-miR-222-5p_L+2R-1
ENSSSCG00000002860	PDCD5	hsa-miR-1246_L+1R+2
ENSSSCG00000002860	PDCD5	ssc-miR-19b
ENSSSCG00000002861	ENSSSCG00000002861	PC-3p-21836_71

ENSSSCG00000002861	ENSSSCG00000002861	PC-5p-9551_196
ENSSSCG00000002861	ENSSSCG00000002861	hsa-miR-129-1-3p
ENSSSCG00000002861	ENSSSCG00000002861	hsa-miR-222-5p_L+2R-1
ENSSSCG00000002863	LRP3	PC-5p-9551_196
ENSSSCG00000002867	CEBPG	hsa-miR-1246_L+1R+2
ENSSSCG00000002867	CEBPG	ssc-mir-4332-p5_1ss18CA
ENSSSCG00000002872	KIAA0355	PC-5p-12443_142
ENSSSCG00000002872	KIAA0355	hsa-miR-222-5p_L+2R-1
ENSSSCG00000002872	KIAA0355	sha-miR-21_L+2R-2
ENSSSCG00000002872	KIAA0355	ssc-miR-190b
ENSSSCG00000002877	ENSSSCG00000002877	PC-5p-6765_300
ENSSSCG00000002877	ENSSSCG00000002877	mmu-miR-107-5p
ENSSSCG00000002877	ENSSSCG00000002877	ssc-miR-181d-5p
ENSSSCG00000002879	GRAMD1A	PC-5p-9551_196
ENSSSCG00000002880	ZNF792	PC-5p-9551_196
ENSSSCG00000002880	ZNF792	hsa-miR-222-5p_L+2R-1
ENSSSCG00000002885	USF2	PC-3p-39317_30
ENSSSCG00000002885	USF2	hsa-miR-23b-5p
ENSSSCG00000002893	ENSSSCG00000002893	PC-3p-21836_71
ENSSSCG00000002893	ENSSSCG00000002893	PC-5p-12443_142
ENSSSCG00000002893	ENSSSCG00000002893	PC-5p-9551_196
ENSSSCG00000002893	ENSSSCG00000002893	cfa-miR-8903_R-2_1ss21GA
ENSSSCG00000002893	ENSSSCG00000002893	ssc-miR-424-5p_R-3
ENSSSCG00000002895	KMT2B	hsa-miR-23b-5p
ENSSSCG00000002898	ENSSSCG00000002898	ssc-miR-339_R-2
ENSSSCG00000002898	ENSSSCG00000002898	ssc-miR-424-5p_R-3
ENSSSCG00000002904	HAUS5	PC-5p-6765_300
ENSSSCG00000002904	HAUS5	hsa-miR-222-5p_L+2R-1
ENSSSCG00000002904	HAUS5	hsa-miR-23b-5p
ENSSSCG00000002904	HAUS5	ssc-miR-181d-5p
ENSSSCG00000002904	HAUS5	ssc-miR-339_R-2

ENSSSCG00000002905	RBM42	hsa-miR-222-5p_L+2R-1
ENSSSCG00000002910	ALKBH6	hsa-miR-23b-5p
ENSSSCG00000002917	NFKBID	hsa-miR-23b-5p
ENSSSCG00000002921	CLIP3	PC-5p-9551_196
ENSSSCG00000002921	CLIP3	hsa-miR-23b-5p
ENSSSCG00000002921	CLIP3	mmu-miR-26a-2-3p_1ss4GA
ENSSSCG00000029170	ENSSSCG00000029170	cfa-miR-8903_R-2_1ss21GA
ENSSSCG00000029170	ENSSSCG00000029170	hsa-miR-873-3p_L-1_1ss11GA
ENSSSCG00000002950	ENSSSCG00000002950	ssc-miR-339_R-2
ENSSSCG00000002952	YIF1B	bta-mir-2478-p3_1ss20GA
ENSSSCG00000002952	YIF1B	hsa-miR-129-1-3p
ENSSSCG00000002952	YIF1B	ssc-mir-4332-p5_1ss18CA
ENSSSCG00000002956	PSMD8	hsa-miR-23b-5p
ENSSSCG00000002986	SELV	hsa-miR-129-1-3p
ENSSSCG00000002996	ENSSSCG00000002996	PC-3p-39317_30
ENSSSCG00000002996	ENSSSCG00000002996	hsa-miR-4792_1ss9GT
ENSSSCG00000002999	C19orf54	PC-5p-6765_300
ENSSSCG00000002999	C19orf54	ssc-miR-339_R-2
ENSSSCG00000003000	ITPKC	PC-5p-9551_196
ENSSSCG00000003000	ITPKC	hsa-miR-222-5p_L+2R-1
ENSSSCG00000003000	ITPKC	ssc-mir-4332-p5_1ss18CA
ENSSSCG00000003013	B9D2	mmu-miR-107-5p
ENSSSCG00000003013	B9D2	ssc-miR-19b
ENSSSCG00000003016	ENSSSCG00000003016	PC-3p-39317_30
ENSSSCG00000003017	TGFB1	hsa-miR-222-5p_L+2R-1
ENSSSCG00000003018	LIPE	cfa-miR-8903_R-2_1ss21GA
ENSSSCG00000003021	MEGF8	PC-5p-9551_196
ENSSSCG00000003021	MEGF8	bta-mir-2478-p3_1ss20GA
ENSSSCG00000003021	MEGF8	ssc-miR-339_R-2
ENSSSCG00000003030	CIC	PC-3p-39317_30
ENSSSCG00000003042	ENSSSCG00000003042	cfa-miR-8903_R-2_1ss21GA
ENSSSCG00000003042	ENSSSCG00000003042	ssc-miR-1839-3p_R+2
ENSSSCG00000003042	ENSSSCG00000003042	ssc-miR-339_R-2
ENSSSCG00000003046	RABAC1	PC-5p-9551_196
ENSSSCG00000003061	CADM4	PC-5p-9551_196

ENSSSCG00000003061	CADM4	hsa-miR-4792_1ss9GT
ENSSSCG00000003062	LYPD3	PC-5p-9551_196
ENSSSCG00000003063	PHLDB3	PC-3p-39317_30
ENSSSCG00000003068	SMG9	bta-mir-2478-p3_1ss20GA
ENSSSCG00000003068	SMG9	ssc-miR-339_R-2
ENSSSCG00000003069	KCNN4	PC-3p-21836_71
ENSSSCG00000003069	KCNN4	ssc-miR-424-5p_R-3
ENSSSCG00000003091	IRF2BP1	ssc-miR-424-5p_R-3
ENSSSCG00000003092	FOXA3	ssc-miR-27b-5p_R-1
ENSSSCG00000003093	SYMPK	PC-3p-21836_71
ENSSSCG00000003093	SYMPK	PC-3p-39317_30
ENSSSCG00000003093	SYMPK	hsa-miR-4792_1ss9GT
ENSSSCG00000003095	NOVA2	PC-3p-39317_30
ENSSSCG00000003098	PPP5C	PC-3p-39317_30
ENSSSCG00000003106	AP2S1	mmu-mir-6240-p5_1ss13AG
ENSSSCG00000003107	ARHGAP35	ssc-miR-339_R-2
ENSSSCG00000003107	ARHGAP35	ssc-miR-424-5p_R-3
ENSSSCG00000003110	ENSSSCG00000003110	mmu-miR-107-5p
ENSSSCG00000003111	SAE1	PC-3p-21836_71
ENSSSCG00000003111	SAE1	bta-mir-2478-p3_1ss20GA
ENSSSCG00000003111	SAE1	hsa-miR-4792_1ss9GT
ENSSSCG00000003114	DHX34	PC-5p-9551_196
ENSSSCG00000003119	ZNF541	ssc-miR-192
ENSSSCG00000003119	ZNF541	ssc-miR-215_R+1
ENSSSCG00000003119	ZNF541	ssc-miR-192
ENSSSCG00000003119	ZNF541	ssc-miR-215_R+1
ENSSSCG00000003120	NAPA	bta-mir-2478-p3_1ss20GA
ENSSSCG00000003125	ELSPBP1	PC-3p-39317_30
ENSSSCG00000003125	ELSPBP1	hsa-miR-873-3p_L-1_1ss11GA
ENSSSCG00000003130	SULT2A1	hsa-miR-4792_1ss9GT
ENSSSCG00000003132	KDELR1	PC-3p-39317_30
ENSSSCG00000003132	KDELR1	cfa-miR-8903_R-2_1ss21GA
ENSSSCG00000003134	GRWD1	PC-5p-9551_196
ENSSSCG00000003135	KCNJ14	PC-5p-9551_196
ENSSSCG00000003136	ENSSSCG00000003136	hsa-miR-1246_L+1R+2
ENSSSCG00000003139	BCAT2	PC-5p-9551_196
ENSSSCG00000003139	BCAT2	ssc-miR-1839-3p_R+2
ENSSSCG00000003141	FUT1	PC-5p-9551_196
ENSSSCG00000003141	FUT1	hsa-miR-4792_1ss9GT
ENSSSCG00000003143	MAMSTR	PC-3p-39317_30
ENSSSCG00000003143	MAMSTR	ssc-miR-424-5p_R-3

ENSSSCG00000003147	ENSSSCG00000003147	cfa-miR-8903_R-2_1ss21GA
ENSSSCG00000003147	ENSSSCG00000003147	sha-miR-21_L+2R-2
ENSSSCG00000023153	ENSSSCG00000023153	cfa-miR-8903_R-2_1ss21GA
ENSSSCG00000023153	ENSSSCG00000023153	sha-miR-21_L+2R-2
ENSSSCG00000003148	ENSSSCG00000003148	PC-5p-9551_196
ENSSSCG00000003150	NUCB1	PC-3p-39317_30
ENSSSCG00000003150	NUCB1	PC-5p-12443_142
ENSSSCG00000003150	NUCB1	cfa-miR-8903_R-2_1ss21GA
ENSSSCG00000003150	NUCB1	hsa-miR-873-3p_L-1_1ss11GA
ENSSSCG00000003150	NUCB1	ssc-miR-339_R-2
ENSSSCG00000003154	GYS1	PC-5p-9551_196
ENSSSCG00000003154	GYS1	mmu-miR-107-5p
ENSSSCG00000003154	GYS1	ssc-miR-27b-5p_R-1
ENSSSCG00000003161	PRRG2	PC-3p-21836_71
ENSSSCG00000003161	PRRG2	hsa-miR-222-5p_L+2R-1
ENSSSCG00000003165	RPS11	ssc-miR-339_R-2
ENSSSCG00000003169	PIH1D1	PC-3p-39317_30
ENSSSCG00000003170	NR5A1	PC-5p-9551_196
ENSSSCG00000003170	NR5A1	cfa-miR-8903_R-2_1ss21GA
ENSSSCG00000003170	NR5A1	mmu-miR-107-5p
ENSSSCG00000003177	BCL2L12	PC-3p-21836_71
ENSSSCG00000003177	BCL2L12	PC-5p-9551_196
ENSSSCG00000003193	NR5A1	PC-3p-39317_30
ENSSSCG00000003194	NR5A1	hsa-miR-129-1-3p
ENSSSCG00000003194	NR5A1	ssc-miR-192
ENSSSCG00000003194	NR5A1	ssc-miR-215_R+1
ENSSSCG00000003199	AP2A1	PC-5p-9551_196
ENSSSCG00000003199	AP2A1	ssc-miR-339_R-2
ENSSSCG00000003199	AP2A1	ssc-miR-424-5p_R-3
ENSSSCG00000003201	ATF5	hsa-miR-141-3p_R+1
ENSSSCG00000003204	VRK3	PC-3p-39317_30
ENSSSCG00000003204	VRK3	PC-5p-9551_196
ENSSSCG00000003204	VRK3	hsa-miR-873-3p_L-1_1ss11GA
ENSSSCG00000003211	NR1H2	ssc-miR-192
ENSSSCG00000003211	NR1H2	ssc-miR-215_R+1
ENSSSCG00000003214	KCNC3	PC-5p-12443_142
ENSSSCG00000003214	KCNC3	hsa-miR-23b-5p
ENSSSCG00000003214	KCNC3	ssc-miR-19b
ENSSSCG00000003214	KCNC3	ssc-miR-339_R-2

ENSSSCG00000003214	KCNC3	ssc-miR-424-5p_R-3
ENSSSCG00000003215	SHANK1	ssc-miR-19b
ENSSSCG00000003215	SHANK1	ssc-miR-19b
ENSSSCG00000003222	SYT3	PC-3p-39317_30
ENSSSCG00000003222	SYT3	ssc-miR-424-5p_R-3
ENSSSCG00000003236	SIGLEC5	PC-3p-21836_71

Supplementary Table S4. Pathways regulated by 13 differentially expressed miRNAs

Gene ID	Symbol	miRNA ID	KEGG pathways
ENSSSCG00000028420	EIF4E	hsa-miR-141-3p_R+1	ko03013
ENSSSCG00000008090	IL1A	hsa-miR-141-3p_R+1	ko04010
ENSSSCG00000018016	MAP2K4	hsa-miR-141-3p_R+1	ko04010
ENSSSCG00000020744	DUSP3	hsa-miR-141-3p_R+1	ko04010
ENSSSCG00000007151	NR5A1	hsa-miR-141-3p_R+1	ko04010
ENSSSCG00000008881	RAPGEF2	hsa-miR-141-3p_R+1	ko04010
ENSSSCG00000016991	DUSP1	hsa-miR-141-3p_R+1	ko04010
ENSSSCG00000005382	TGFBR1	hsa-miR-141-3p_R+1	ko04010
ENSSSCG00000004110	TAB2	hsa-miR-141-3p_R+1	ko04010
ENSSSCG00000027443	MRAS	hsa-miR-141-3p_R+1	ko04010
ENSSSCG00000022331	FGF13	hsa-miR-141-3p_R+1	ko04010
ENSSSCG00000003471	EPHA2	hsa-miR-141-3p_R+1	ko04014
ENSSSCG00000003756	LPAR3	hsa-miR-141-3p_R+1	ko04014
ENSSSCG00000004622	GNB5	hsa-miR-141-3p_R+1	ko04014
ENSSSCG00000007356	PLCG1	hsa-miR-141-3p_R+1	ko04014
ENSSSCG00000009048	GAB1	hsa-miR-141-3p_R+1	ko04014
ENSSSCG00000009567	RASA3	hsa-miR-141-3p_R+1	ko04014
ENSSSCG00000015383	RAPGEF5	hsa-miR-141-3p_R+1	ko04014
ENSSSCG00000015403	HGF	hsa-miR-141-3p_R+1	ko04014
ENSSSCG00000015770	VEGFC	hsa-miR-141-3p_R+1	ko04014
ENSSSCG00000004622	GNB5	hsa-miR-141-3p_R+1	ko04014
ENSSSCG00000012034	TIAM1	hsa-miR-141-3p_R+1	ko04014
ENSSSCG00000027443	MRAS	hsa-miR-141-3p_R+1	ko04014
ENSSSCG00000022331	FGF13	hsa-miR-141-3p_R+1	ko04014
ENSSSCG00000030289	PTPN11	hsa-miR-141-3p_R+1	ko04014
ENSSSCG00000009048	GAB1	hsa-miR-141-3p_R+1	ko04014
ENSSSCG00000009048	GAB1	hsa-miR-141-3p_R+1	ko04014
ENSSSCG00000009048	GAB1	hsa-miR-141-3p_R+1	ko04014
ENSSSCG00000008881	RAPGEF2	hsa-miR-141-3p_R+1	ko04015
ENSSSCG00000011274	CTNNB1	hsa-miR-141-3p_R+1	ko04015
ENSSSCG00000015383	RAPGEF5	hsa-miR-141-3p_R+1	ko04015
ENSSSCG00000012034	TIAM1	hsa-miR-141-3p_R+1	ko04015
ENSSSCG00000027443	MRAS	hsa-miR-141-3p_R+1	ko04015

ENSSSCG00000015407	GNAI1	hsa-miR-141-3p_R+1	ko04015
ENSSSCG00000017101	ADCY2	hsa-miR-141-3p_R+1	ko04015
ENSSSCG00000003471	EPHA2	hsa-miR-141-3p_R+1	ko04015
ENSSSCG00000003756	LPAR3	hsa-miR-141-3p_R+1	ko04015
ENSSSCG00000007356	PLCG1	hsa-miR-141-3p_R+1	ko04015
ENSSSCG00000015403	HGF	hsa-miR-141-3p_R+1	ko04015
ENSSSCG00000015770	VEGFC	hsa-miR-141-3p_R+1	ko04015
ENSSSCG00000022331	FGF13	hsa-miR-141-3p_R+1	ko04015
ENSSSCG00000001009	RIPK1	hsa-miR-141-3p_R+1	ko04064
ENSSSCG00000004110	TAB2	hsa-miR-141-3p_R+1	ko04064
ENSSSCG00000006862	VCAM1	hsa-miR-141-3p_R+1	ko04064
ENSSSCG00000007356	PLCG1	hsa-miR-141-3p_R+1	ko04064
ENSSSCG00000025028	XIAP	hsa-miR-141-3p_R+1	ko04064
ENSSSCG00000010414	CXCL12	hsa-miR-141-3p_R+1	ko04064
ENSSSCG00000015407	GNAI1	hsa-miR-141-3p_R+1	ko04071
ENSSSCG00000025028	XIAP	hsa-miR-141-3p_R+1	ko04120
ENSSSCG00000005382	TGFBR1	hsa-miR-141-3p_R+1	ko04144
ENSSSCG00000028420	EIF4E	hsa-miR-141-3p_R+1	ko04150
ENSSSCG00000006927	PKN2	hsa-miR-141-3p_R+1	ko04151
ENSSSCG00000007692	YWHAG	hsa-miR-141-3p_R+1	ko04151
ENSSSCG00000028420	EIF4E	hsa-miR-141-3p_R+1	ko04151
ENSSSCG00000016887	ITGA2	hsa-miR-141-3p_R+1	ko04151
ENSSSCG00000016027	ITGAV	hsa-miR-141-3p_R+1	ko04151
ENSSSCG00000003471	EPHA2	hsa-miR-141-3p_R+1	ko04151
ENSSSCG00000003756	LPAR3	hsa-miR-141-3p_R+1	ko04151
ENSSSCG00000004622	GNB5	hsa-miR-141-3p_R+1	ko04151
ENSSSCG00000004896	PHLPP1	hsa-miR-141-3p_R+1	ko04151
ENSSSCG00000006062	YWHAZ	hsa-miR-141-3p_R+1	ko04151
ENSSSCG00000006095	CCNE2	hsa-miR-141-3p_R+1	ko04151
ENSSSCG00000015403	HGF	hsa-miR-141-3p_R+1	ko04151
ENSSSCG00000015770	VEGFC	hsa-miR-141-3p_R+1	ko04151
ENSSSCG00000004622	GNB5	hsa-miR-141-3p_R+1	ko04151
ENSSSCG00000022331	FGF13	hsa-miR-141-3p_R+1	ko04151
ENSSSCG00000008090	IL1A	hsa-miR-141-3p_R+1	ko04210
ENSSSCG00000020906	TNFSF10	hsa-miR-141-3p_R+1	ko04210
ENSSSCG00000025028	XIAP	hsa-miR-141-3p_R+1	ko04210
ENSSSCG00000001009	RIPK1	hsa-miR-141-3p_R+1	ko04210
ENSSSCG00000009131	PITX2	hsa-miR-141-3p_R+1	ko04350
ENSSSCG00000011014	BAMBI	hsa-miR-141-3p_R+1	ko04350
ENSSSCG00000016113	BMPR2	hsa-miR-141-3p_R+1	ko04350
ENSSSCG00000005382	TGFBR1	hsa-miR-141-3p_R+1	ko04350
ENSSSCG00000004110	TAB2	hsa-miR-141-3p_R+1	ko04380
ENSSSCG00000008090	IL1A	hsa-miR-141-3p_R+1	ko04380
ENSSSCG00000025396	YAP1	hsa-miR-141-3p_R+1	ko04390

ENSSSCG00000007692	YWHAG	hsa-miR-141-3p_R+1	ko04390
ENSSSCG00000008241	TCF7L1	hsa-miR-141-3p_R+1	ko04390
ENSSSCG00000029852	WNT5A	hsa-miR-141-3p_R+1	ko04390
ENSSSCG00000016113	BMPR2	hsa-miR-141-3p_R+1	ko04390
ENSSSCG00000006062	YWHAZ	hsa-miR-141-3p_R+1	ko04390
ENSSSCG00000011274	CTNNB1	hsa-miR-141-3p_R+1	ko04390
ENSSSCG00000005382	TGFBR1	hsa-miR-141-3p_R+1	ko04390
ENSSSCG00000016887	ITGA2	hsa-miR-141-3p_R+1	ko04510
ENSSSCG00000016027	ITGAV	hsa-miR-141-3p_R+1	ko04510
ENSSSCG00000011274	CTNNB1	hsa-miR-141-3p_R+1	ko04510
ENSSSCG00000025028	XIAP	hsa-miR-141-3p_R+1	ko04510
ENSSSCG00000015403	HGF	hsa-miR-141-3p_R+1	ko04510
ENSSSCG00000015770	VEGFC	hsa-miR-141-3p_R+1	ko04510
ENSSSCG00000008977	CXCL10	hsa-miR-141-3p_R+1	ko04622
ENSSSCG00000008977	CXCL10	hsa-miR-141-3p_R+1	ko04622
ENSSSCG00000001009	RIPK1	hsa-miR-141-3p_R+1	ko04622
ENSSSCG00000008977	CXCL10	hsa-miR-141-3p_R+1	ko04623
ENSSSCG00000008977	CXCL10	hsa-miR-141-3p_R+1	ko04623
ENSSSCG00000001009	RIPK1	hsa-miR-141-3p_R+1	ko04623
ENSSSCG00000007356	PLCG1	hsa-miR-141-3p_R+1	ko04660
ENSSSCG00000001009	RIPK1	hsa-miR-141-3p_R+1	ko04668
ENSSSCG00000004110	TAB2	hsa-miR-141-3p_R+1	ko04668
ENSSSCG00000006286	SELE	hsa-miR-141-3p_R+1	ko04668
ENSSSCG00000006862	VCAM1	hsa-miR-141-3p_R+1	ko04668
ENSSSCG00000008977	CXCL10	hsa-miR-141-3p_R+1	ko04668
ENSSSCG00000018016	MAP2K4	hsa-miR-141-3p_R+1	ko04668
ENSSSCG00000006286	SELE	hsa-miR-141-3p_R+1	ko04668
ENSSSCG00000008977	CXCL10	hsa-miR-141-3p_R+1	ko04668
ENSSSCG00000017101	ADCY2	hsa-miR-141-3p_R+1	ko04923
ENSSSCG00000015407	GNAI1	hsa-miR-141-3p_R+1	ko04923
ENSSSCG00000025396	YAP1	hsa-miR-141-3p_R+1	ko05166
ENSSSCG00000006862	VCAM1	hsa-miR-141-3p_R+1	ko05166
ENSSSCG00000018016	MAP2K4	hsa-miR-141-3p_R+1	ko05166
ENSSSCG00000029852	WNT5A	hsa-miR-141-3p_R+1	ko05166
ENSSSCG00000017101	ADCY2	hsa-miR-141-3p_R+1	ko05166
ENSSSCG00000027443	MRAS	hsa-miR-141-3p_R+1	ko05166
ENSSSCG00000005382	TGFBR1	hsa-miR-141-3p_R+1	ko05166
ENSSSCG00000011274	CTNNB1	hsa-miR-141-3p_R+1	ko05166
ENSSSCG00000025028	XIAP	hsa-miR-141-3p_R+1	ko05166
ENSSSCG00000006095	CCNE2	hsa-miR-141-3p_R+1	ko05200
ENSSSCG00000008241	TCF7L1	hsa-miR-141-3p_R+1	ko05200
ENSSSCG00000029852	WNT5A	hsa-miR-141-3p_R+1	ko05200
ENSSSCG00000017101	ADCY2	hsa-miR-141-3p_R+1	ko05200
ENSSSCG00000016887	ITGA2	hsa-miR-141-3p_R+1	ko05200

ENSSSCG00000010414	CXCL12	hsa-miR-141-3p_R+1	ko05200
ENSSSCG00000016027	ITGAV	hsa-miR-141-3p_R+1	ko05200
ENSSSCG00000004622	GNB5	hsa-miR-141-3p_R+1	ko05200
ENSSSCG00000011274	CTNNB1	hsa-miR-141-3p_R+1	ko05200
ENSSSCG00000004622	GNB5	hsa-miR-141-3p_R+1	ko05200
ENSSSCG00000025028	XIAP	hsa-miR-141-3p_R+1	ko05200
ENSSSCG00000003756	LPAR3	hsa-miR-141-3p_R+1	ko05200
ENSSSCG00000007356	PLCG1	hsa-miR-141-3p_R+1	ko05200
ENSSSCG00000015403	HGF	hsa-miR-141-3p_R+1	ko05200
ENSSSCG00000015407	GNAI1	hsa-miR-141-3p_R+1	ko05200
ENSSSCG00000015770	VEGFC	hsa-miR-141-3p_R+1	ko05200
ENSSSCG00000005382	TGFBR1	hsa-miR-141-3p_R+1	ko05200
ENSSSCG00000022331	FGF13	hsa-miR-141-3p_R+1	ko05200
ENSSSCG00000009048	GAB1	hsa-miR-141-3p_R+1	ko05205
ENSSSCG00000030289	PTPN11	hsa-miR-141-3p_R+1	ko05205
ENSSSCG00000009048	GAB1	hsa-miR-141-3p_R+1	ko05205
ENSSSCG00000009048	GAB1	hsa-miR-141-3p_R+1	ko05205
ENSSSCG00000009048	GAB1	hsa-miR-141-3p_R+1	ko05205
ENSSSCG00000029852	WNT5A	hsa-miR-141-3p_R+1	ko05205
ENSSSCG00000016887	ITGA2	hsa-miR-141-3p_R+1	ko05205
ENSSSCG00000016027	ITGAV	hsa-miR-141-3p_R+1	ko05205
ENSSSCG00000027443	MRAS	hsa-miR-141-3p_R+1	ko05205
ENSSSCG00000007356	PLCG1	hsa-miR-141-3p_R+1	ko05205
ENSSSCG00000015403	HGF	hsa-miR-141-3p_R+1	ko05205
ENSSSCG00000011274	CTNNB1	hsa-miR-141-3p_R+1	ko05205
ENSSSCG00000008241	TCF7L1	hsa-miR-141-3p_R+1	ko05210
ENSSSCG00000011274	CTNNB1	hsa-miR-141-3p_R+1	ko05210
ENSSSCG00000005382	TGFBR1	hsa-miR-141-3p_R+1	ko05210
ENSSSCG00000009048	GAB1	hsa-miR-141-3p_R+1	ko05211
ENSSSCG00000030289	PTPN11	hsa-miR-141-3p_R+1	ko05211
ENSSSCG00000009048	GAB1	hsa-miR-141-3p_R+1	ko05211
ENSSSCG00000009048	GAB1	hsa-miR-141-3p_R+1	ko05211
ENSSSCG00000009048	GAB1	hsa-miR-141-3p_R+1	ko05211
ENSSSCG00000015403	HGF	hsa-miR-141-3p_R+1	ko05211
ENSSSCG00000005382	TGFBR1	hsa-miR-141-3p_R+1	ko05212
ENSSSCG00000007356	PLCG1	hsa-miR-141-3p_R+1	ko05214
ENSSSCG00000006095	CCNE2	hsa-miR-141-3p_R+1	ko05215
ENSSSCG00000008241	TCF7L1	hsa-miR-141-3p_R+1	ko05215
ENSSSCG00000011274	CTNNB1	hsa-miR-141-3p_R+1	ko05215
ENSSSCG00000007356	PLCG1	hsa-miR-141-3p_R+1	ko05223
ENSSSCG00000016887	ITGA2	hsa-miR-141-3p_R+1	ko05410
ENSSSCG00000016027	ITGAV	hsa-miR-141-3p_R+1	ko05410
ENSSSCG00000001901	CYP1A2	hsa-miR-222-5p_L+2R-1	ko00140
ENSSSCG00000017748	NF1	hsa-miR-222-5p_L+2R-1	ko04010

ENSSSCG00000017748	NF1	hsa-miR-222-5p_L+2R-1	ko04010
ENSSSCG00000003017	TGFB1	hsa-miR-222-5p_L+2R-1	ko04010
ENSSSCG00000022482	DDIT3	hsa-miR-222-5p_L+2R-1	ko04010
ENSSSCG00000024813	MAP3K3	hsa-miR-222-5p_L+2R-1	ko04010
ENSSSCG00000003017	TGFB1	hsa-miR-222-5p_L+2R-1	ko04010
ENSSSCG00000009228	MAPK10	hsa-miR-222-5p_L+2R-1	ko04010
ENSSSCG00000015815	NR5A1	hsa-miR-222-5p_L+2R-1	ko04010
ENSSSCG00000016878	FGF10	hsa-miR-222-5p_L+2R-1	ko04010
ENSSSCG00000026969	KRAS	hsa-miR-222-5p_L+2R-1	ko04010
ENSSSCG00000027443	MRAS	hsa-miR-222-5p_L+2R-1	ko04010
ENSSSCG00000024954	FGF1	hsa-miR-222-5p_L+2R-1	ko04010
ENSSSCG00000015815	NR5A1	hsa-miR-222-5p_L+2R-1	ko04010
ENSSSCG00000015815	NR5A1	hsa-miR-222-5p_L+2R-1	ko04010
ENSSSCG00000015815	NR5A1	hsa-miR-222-5p_L+2R-1	ko04010
ENSSSCG00000005738	RALGDS	hsa-miR-222-5p_L+2R-1	ko04014
ENSSSCG00000007356	PLCG1	hsa-miR-222-5p_L+2R-1	ko04014
ENSSSCG00000009228	MAPK10	hsa-miR-222-5p_L+2R-1	ko04014
ENSSSCG00000009855	KSR2	hsa-miR-222-5p_L+2R-1	ko04014
ENSSSCG00000014891	NR5A1	hsa-miR-222-5p_L+2R-1	ko04014
ENSSSCG00000015815	NR5A1	hsa-miR-222-5p_L+2R-1	ko04014
ENSSSCG00000016878	FGF10	hsa-miR-222-5p_L+2R-1	ko04014
ENSSSCG00000017748	NF1	hsa-miR-222-5p_L+2R-1	ko04014
ENSSSCG00000017748	NF1	hsa-miR-222-5p_L+2R-1	ko04014
ENSSSCG00000012034	TIAM1	hsa-miR-222-5p_L+2R-1	ko04014
ENSSSCG00000026969	KRAS	hsa-miR-222-5p_L+2R-1	ko04014
ENSSSCG00000027443	MRAS	hsa-miR-222-5p_L+2R-1	ko04014
ENSSSCG00000024954	FGF1	hsa-miR-222-5p_L+2R-1	ko04014
ENSSSCG00000015815	NR5A1	hsa-miR-222-5p_L+2R-1	ko04014
ENSSSCG00000015815	NR5A1	hsa-miR-222-5p_L+2R-1	ko04014
ENSSSCG00000015815	NR5A1	hsa-miR-222-5p_L+2R-1	ko04014
ENSSSCG00000015815	NR5A1	hsa-miR-222-5p_L+2R-1	ko04014
ENSSSCG00000012034	TIAM1	hsa-miR-222-5p_L+2R-1	ko04015
ENSSSCG00000027443	MRAS	hsa-miR-222-5p_L+2R-1	ko04015
ENSSSCG00000007356	PLCG1	hsa-miR-222-5p_L+2R-1	ko04015
ENSSSCG00000015815	NR5A1	hsa-miR-222-5p_L+2R-1	ko04015
ENSSSCG00000005738	RALGDS	hsa-miR-222-5p_L+2R-1	ko04015
ENSSSCG00000015815	NR5A1	hsa-miR-222-5p_L+2R-1	ko04015
ENSSSCG00000016878	FGF10	hsa-miR-222-5p_L+2R-1	ko04015
ENSSSCG00000024954	FGF1	hsa-miR-222-5p_L+2R-1	ko04015
ENSSSCG00000015815	NR5A1	hsa-miR-222-5p_L+2R-1	ko04015
ENSSSCG00000015815	NR5A1	hsa-miR-222-5p_L+2R-1	ko04015
ENSSSCG00000015815	NR5A1	hsa-miR-222-5p_L+2R-1	ko04015
ENSSSCG00000026969	KRAS	hsa-miR-222-5p_L+2R-1	ko04015
ENSSSCG00000007356	PLCG1	hsa-miR-222-5p_L+2R-1	ko04064

ENSSSCG00000013655	ICAM1	hsa-miR-222-5p_L+2R-1	ko04064
ENSSSCG00000016101	CFLAR	hsa-miR-222-5p_L+2R-1	ko04064
ENSSSCG00000021068	TRAF5	hsa-miR-222-5p_L+2R-1	ko04064
ENSSSCG00000013655	ICAM1	hsa-miR-222-5p_L+2R-1	ko04064
ENSSSCG00000016101	CFLAR	hsa-miR-222-5p_L+2R-1	ko04064
ENSSSCG00000002804	CSNK2A2	hsa-miR-222-5p_L+2R-1	ko04064
ENSSSCG00000014891	NR5A1	hsa-miR-222-5p_L+2R-1	ko04071
ENSSSCG00000009228	MAPK10	hsa-miR-222-5p_L+2R-1	ko04071
ENSSSCG00000026969	KRAS	hsa-miR-222-5p_L+2R-1	ko04071
ENSSSCG00000030153	SMURF1	hsa-miR-222-5p_L+2R-1	ko04120
ENSSSCG00000022849	IL2RA	hsa-miR-222-5p_L+2R-1	ko04144
ENSSSCG00000030153	SMURF1	hsa-miR-222-5p_L+2R-1	ko04144
ENSSSCG00000004952	SMAD3	hsa-miR-222-5p_L+2R-1	ko04144
ENSSSCG00000003017	TGFB1	hsa-miR-222-5p_L+2R-1	ko04144
ENSSSCG00000003017	TGFB1	hsa-miR-222-5p_L+2R-1	ko04144
ENSSSCG00000006927	PKN2	hsa-miR-222-5p_L+2R-1	ko04151
ENSSSCG00000013888	JAK3	hsa-miR-222-5p_L+2R-1	ko04151
ENSSSCG00000025092	CDK4	hsa-miR-222-5p_L+2R-1	ko04151
ENSSSCG00000022849	IL2RA	hsa-miR-222-5p_L+2R-1	ko04151
ENSSSCG00000016887	ITGA2	hsa-miR-222-5p_L+2R-1	ko04151
ENSSSCG00000013888	JAK3	hsa-miR-222-5p_L+2R-1	ko04151
ENSSSCG00000005738	RALGDS	hsa-miR-222-5p_L+2R-1	ko04151
ENSSSCG00000015815	NR5A1	hsa-miR-222-5p_L+2R-1	ko04151
ENSSSCG00000016878	FGF10	hsa-miR-222-5p_L+2R-1	ko04151
ENSSSCG00000026969	KRAS	hsa-miR-222-5p_L+2R-1	ko04151
ENSSSCG00000024954	FGF1	hsa-miR-222-5p_L+2R-1	ko04151
ENSSSCG00000015815	NR5A1	hsa-miR-222-5p_L+2R-1	ko04151
ENSSSCG00000015815	NR5A1	hsa-miR-222-5p_L+2R-1	ko04151
ENSSSCG00000015815	NR5A1	hsa-miR-222-5p_L+2R-1	ko04151
ENSSSCG00000015815	NR5A1	hsa-miR-222-5p_L+2R-1	ko04151
ENSSSCG00000016101	CFLAR	hsa-miR-222-5p_L+2R-1	ko04210
ENSSSCG00000016101	CFLAR	hsa-miR-222-5p_L+2R-1	ko04210
ENSSSCG00000004952	SMAD3	hsa-miR-222-5p_L+2R-1	ko04350
ENSSSCG00000006145	E2F5	hsa-miR-222-5p_L+2R-1	ko04350
ENSSSCG00000011014	BAMBI	hsa-miR-222-5p_L+2R-1	ko04350
ENSSSCG00000024312	ID4	hsa-miR-222-5p_L+2R-1	ko04350
ENSSSCG00000030153	SMURF1	hsa-miR-222-5p_L+2R-1	ko04350
ENSSSCG00000003017	TGFB1	hsa-miR-222-5p_L+2R-1	ko04350
ENSSSCG00000003017	TGFB1	hsa-miR-222-5p_L+2R-1	ko04350
ENSSSCG00000014891	NR5A1	hsa-miR-222-5p_L+2R-1	ko04380
ENSSSCG00000009228	MAPK10	hsa-miR-222-5p_L+2R-1	ko04380
ENSSSCG00000004192	CTGF	hsa-miR-222-5p_L+2R-1	ko04390
ENSSSCG00000012591	AMOT	hsa-miR-222-5p_L+2R-1	ko04390
ENSSSCG00000012591	AMOT	hsa-miR-222-5p_L+2R-1	ko04390

ENSSSCG00000004952	SMAD3	hsa-miR-222-5p_L+2R-1	ko04390
ENSSSCG00000003017	TGFB1	hsa-miR-222-5p_L+2R-1	ko04390
ENSSSCG00000024954	FGF1	hsa-miR-222-5p_L+2R-1	ko04390
ENSSSCG00000003017	TGFB1	hsa-miR-222-5p_L+2R-1	ko04390
ENSSSCG00000016887	ITGA2	hsa-miR-222-5p_L+2R-1	ko04510
ENSSSCG00000009228	MAPK10	hsa-miR-222-5p_L+2R-1	ko04510
ENSSSCG00000017705	CCL5	hsa-miR-222-5p_L+2R-1	ko04623
ENSSSCG00000025092	CDK4	hsa-miR-222-5p_L+2R-1	ko04660
ENSSSCG00000007356	PLCG1	hsa-miR-222-5p_L+2R-1	ko04660
ENSSSCG00000013655	ICAM1	hsa-miR-222-5p_L+2R-1	ko04668
ENSSSCG00000016101	CFLAR	hsa-miR-222-5p_L+2R-1	ko04668
ENSSSCG00000021068	TRAF5	hsa-miR-222-5p_L+2R-1	ko04668
ENSSSCG00000027426	NR5A1	hsa-miR-222-5p_L+2R-1	ko04668
ENSSSCG00000013655	ICAM1	hsa-miR-222-5p_L+2R-1	ko04668
ENSSSCG00000017705	CCL5	hsa-miR-222-5p_L+2R-1	ko04668
ENSSSCG00000016101	CFLAR	hsa-miR-222-5p_L+2R-1	ko04668
ENSSSCG00000009228	MAPK10	hsa-miR-222-5p_L+2R-1	ko04668
ENSSSCG00000009228	MAPK10	hsa-miR-222-5p_L+2R-1	ko04930
ENSSSCG00000013888	JAK3	hsa-miR-222-5p_L+2R-1	ko05166
ENSSSCG00000013888	JAK3	hsa-miR-222-5p_L+2R-1	ko05166
ENSSSCG00000024813	MAP3K3	hsa-miR-222-5p_L+2R-1	ko05166
ENSSSCG00000013655	ICAM1	hsa-miR-222-5p_L+2R-1	ko05166
ENSSSCG00000013655	ICAM1	hsa-miR-222-5p_L+2R-1	ko05166
ENSSSCG00000004952	SMAD3	hsa-miR-222-5p_L+2R-1	ko05166
ENSSSCG00000027443	MRAS	hsa-miR-222-5p_L+2R-1	ko05166
ENSSSCG00000025092	CDK4	hsa-miR-222-5p_L+2R-1	ko05166
ENSSSCG00000003017	TGFB1	hsa-miR-222-5p_L+2R-1	ko05166
ENSSSCG00000003017	TGFB1	hsa-miR-222-5p_L+2R-1	ko05166
ENSSSCG00000016887	ITGA2	hsa-miR-222-5p_L+2R-1	ko05200
ENSSSCG00000004952	SMAD3	hsa-miR-222-5p_L+2R-1	ko05200
ENSSSCG00000021068	TRAF5	hsa-miR-222-5p_L+2R-1	ko05200
ENSSSCG00000025092	CDK4	hsa-miR-222-5p_L+2R-1	ko05200
ENSSSCG00000003017	TGFB1	hsa-miR-222-5p_L+2R-1	ko05200
ENSSSCG00000007356	PLCG1	hsa-miR-222-5p_L+2R-1	ko05200
ENSSSCG00000003017	TGFB1	hsa-miR-222-5p_L+2R-1	ko05200
ENSSSCG00000015815	NR5A1	hsa-miR-222-5p_L+2R-1	ko05200
ENSSSCG00000005738	RALGDS	hsa-miR-222-5p_L+2R-1	ko05200
ENSSSCG00000009228	MAPK10	hsa-miR-222-5p_L+2R-1	ko05200
ENSSSCG00000015815	NR5A1	hsa-miR-222-5p_L+2R-1	ko05200
ENSSSCG00000016878	FGF10	hsa-miR-222-5p_L+2R-1	ko05200
ENSSSCG00000024954	FGF1	hsa-miR-222-5p_L+2R-1	ko05200
ENSSSCG00000015815	NR5A1	hsa-miR-222-5p_L+2R-1	ko05200
ENSSSCG00000015815	NR5A1	hsa-miR-222-5p_L+2R-1	ko05200
ENSSSCG00000015815	NR5A1	hsa-miR-222-5p_L+2R-1	ko05200

ENSSSCG00000026969	KRAS	hsa-miR-222-5p_L+2R-1	ko05200
ENSSSCG00000001518	ITPR3	hsa-miR-222-5p_L+2R-1	ko05205
ENSSSCG00000012034	TIAM1	hsa-miR-222-5p_L+2R-1	ko05205
ENSSSCG00000016887	ITGA2	hsa-miR-222-5p_L+2R-1	ko05205
ENSSSCG00000027443	MRAS	hsa-miR-222-5p_L+2R-1	ko05205
ENSSSCG00000015815	NR5A1	hsa-miR-222-5p_L+2R-1	ko05205
ENSSSCG00000007356	PLCG1	hsa-miR-222-5p_L+2R-1	ko05205
ENSSSCG00000015815	NR5A1	hsa-miR-222-5p_L+2R-1	ko05205
ENSSSCG00000015815	NR5A1	hsa-miR-222-5p_L+2R-1	ko05205
ENSSSCG00000015815	NR5A1	hsa-miR-222-5p_L+2R-1	ko05205
ENSSSCG00000015815	NR5A1	hsa-miR-222-5p_L+2R-1	ko05205
ENSSSCG00000003017	TGFB1	hsa-miR-222-5p_L+2R-1	ko05205
ENSSSCG00000026969	KRAS	hsa-miR-222-5p_L+2R-1	ko05205
ENSSSCG00000003017	TGFB1	hsa-miR-222-5p_L+2R-1	ko05205
ENSSSCG00000005738	RALGDS	hsa-miR-222-5p_L+2R-1	ko05210
ENSSSCG00000004952	SMAD3	hsa-miR-222-5p_L+2R-1	ko05210
ENSSSCG00000026969	KRAS	hsa-miR-222-5p_L+2R-1	ko05210
ENSSSCG00000009228	MAPK10	hsa-miR-222-5p_L+2R-1	ko05210
ENSSSCG00000003017	TGFB1	hsa-miR-222-5p_L+2R-1	ko05210
ENSSSCG00000003017	TGFB1	hsa-miR-222-5p_L+2R-1	ko05210
ENSSSCG00000003017	TGFB1	hsa-miR-222-5p_L+2R-1	ko05211
ENSSSCG00000003017	TGFB1	hsa-miR-222-5p_L+2R-1	ko05211
ENSSSCG00000004952	SMAD3	hsa-miR-222-5p_L+2R-1	ko05212
ENSSSCG00000025092	CDK4	hsa-miR-222-5p_L+2R-1	ko05212
ENSSSCG00000003017	TGFB1	hsa-miR-222-5p_L+2R-1	ko05212
ENSSSCG00000005738	RALGDS	hsa-miR-222-5p_L+2R-1	ko05212
ENSSSCG00000003017	TGFB1	hsa-miR-222-5p_L+2R-1	ko05212
ENSSSCG00000009228	MAPK10	hsa-miR-222-5p_L+2R-1	ko05212
ENSSSCG00000026969	KRAS	hsa-miR-222-5p_L+2R-1	ko05212
ENSSSCG00000007356	PLCG1	hsa-miR-222-5p_L+2R-1	ko05214
ENSSSCG00000025092	CDK4	hsa-miR-222-5p_L+2R-1	ko05214
ENSSSCG00000015815	NR5A1	hsa-miR-222-5p_L+2R-1	ko05215
ENSSSCG00000015815	NR5A1	hsa-miR-222-5p_L+2R-1	ko05215
ENSSSCG00000015815	NR5A1	hsa-miR-222-5p_L+2R-1	ko05215
ENSSSCG00000015815	NR5A1	hsa-miR-222-5p_L+2R-1	ko05215
ENSSSCG00000015815	NR5A1	hsa-miR-222-5p_L+2R-1	ko05215
ENSSSCG00000026969	KRAS	hsa-miR-222-5p_L+2R-1	ko05215
ENSSSCG00000025092	CDK4	hsa-miR-222-5p_L+2R-1	ko05223
ENSSSCG00000007356	PLCG1	hsa-miR-222-5p_L+2R-1	ko05223
ENSSSCG00000016887	ITGA2	hsa-miR-222-5p_L+2R-1	ko05410
ENSSSCG00000010132	COMT	hsa-miR-4792_1ss9GT	ko00140
ENSSSCG00000008162	IL1R1	hsa-miR-4792_1ss9GT	ko04010
ENSSSCG00000002960	RASGRP4	hsa-miR-4792_1ss9GT	ko04010
ENSSSCG00000004022	RPS6KA2	hsa-miR-4792_1ss9GT	ko04010

ENSSSCG00000011412	CACNA2D2	hsa-miR-4792_1ss9GT	ko04010
ENSSSCG00000011415	MAPKAPK3	hsa-miR-4792_1ss9GT	ko04010
ENSSSCG00000027668	NLK	hsa-miR-4792_1ss9GT	ko04010
ENSSSCG00000022689	GADD45B	hsa-miR-4792_1ss9GT	ko04010
ENSSSCG00000015850	DUSP4	hsa-miR-4792_1ss9GT	ko04010
ENSSSCG00000005838	TRAF2	hsa-miR-4792_1ss9GT	ko04010
ENSSSCG00000008842	KIT	hsa-miR-4792_1ss9GT	ko04014
ENSSSCG00000009855	KSR2	hsa-miR-4792_1ss9GT	ko04014
ENSSSCG00000012399	FOXO4	hsa-miR-4792_1ss9GT	ko04014
ENSSSCG00000002960	RASGRP4	hsa-miR-4792_1ss9GT	ko04014
ENSSSCG00000013564	INSR	hsa-miR-4792_1ss9GT	ko04014
ENSSSCG00000017357	ITGA2B	hsa-miR-4792_1ss9GT	ko04015
ENSSSCG00000017306	ITGB3	hsa-miR-4792_1ss9GT	ko04015
ENSSSCG00000011101	ITGB1	hsa-miR-4792_1ss9GT	ko04015
ENSSSCG00000017306	ITGB3	hsa-miR-4792_1ss9GT	ko04015
ENSSSCG00000007520	GNAS	hsa-miR-4792_1ss9GT	ko04015
ENSSSCG00000008842	KIT	hsa-miR-4792_1ss9GT	ko04015
ENSSSCG00000013564	INSR	hsa-miR-4792_1ss9GT	ko04015
ENSSSCG00000008162	IL1R1	hsa-miR-4792_1ss9GT	ko04064
ENSSSCG00000022066	PIAS4	hsa-miR-4792_1ss9GT	ko04064
ENSSSCG00000012495	BTK	hsa-miR-4792_1ss9GT	ko04064
ENSSSCG00000012495	BTK	hsa-miR-4792_1ss9GT	ko04064
ENSSSCG00000012495	BTK	hsa-miR-4792_1ss9GT	ko04064
ENSSSCG00000012495	BTK	hsa-miR-4792_1ss9GT	ko04064
ENSSSCG00000005838	TRAF2	hsa-miR-4792_1ss9GT	ko04064
ENSSSCG00000005838	TRAF2	hsa-miR-4792_1ss9GT	ko04071
ENSSSCG00000022066	PIAS4	hsa-miR-4792_1ss9GT	ko04120
ENSSSCG00000004507	SMAD7	hsa-miR-4792_1ss9GT	ko04144
ENSSSCG00000008842	KIT	hsa-miR-4792_1ss9GT	ko04144
ENSSSCG00000001898	ULK3	hsa-miR-4792_1ss9GT	ko04150
ENSSSCG00000004022	RPS6KA2	hsa-miR-4792_1ss9GT	ko04150
ENSSSCG00000006927	PKN2	hsa-miR-4792_1ss9GT	ko04151
ENSSSCG00000007673	NR5A1	hsa-miR-4792_1ss9GT	ko04151
ENSSSCG00000017357	ITGA2B	hsa-miR-4792_1ss9GT	ko04151
ENSSSCG00000017306	ITGB3	hsa-miR-4792_1ss9GT	ko04151
ENSSSCG00000011101	ITGB1	hsa-miR-4792_1ss9GT	ko04151
ENSSSCG00000017306	ITGB3	hsa-miR-4792_1ss9GT	ko04151
ENSSSCG00000005838	TRAF2	hsa-miR-4792_1ss9GT	ko04151
ENSSSCG00000008842	KIT	hsa-miR-4792_1ss9GT	ko04151
ENSSSCG00000013564	INSR	hsa-miR-4792_1ss9GT	ko04151
ENSSSCG00000008162	IL1R1	hsa-miR-4792_1ss9GT	ko04210
ENSSSCG00000005838	TRAF2	hsa-miR-4792_1ss9GT	ko04210
ENSSSCG00000025374	DVL3	hsa-miR-4792_1ss9GT	ko04330
ENSSSCG00000004507	SMAD7	hsa-miR-4792_1ss9GT	ko04350

ENSSSCG00000012495	BTK	hsa-miR-4792_1ss9GT	ko04380
ENSSSCG00000012495	BTK	hsa-miR-4792_1ss9GT	ko04380
ENSSSCG00000012495	BTK	hsa-miR-4792_1ss9GT	ko04380
ENSSSCG00000012495	BTK	hsa-miR-4792_1ss9GT	ko04380
ENSSSCG00000017306	ITGB3	hsa-miR-4792_1ss9GT	ko04380
ENSSSCG00000017306	ITGB3	hsa-miR-4792_1ss9GT	ko04380
ENSSSCG00000008162	IL1R1	hsa-miR-4792_1ss9GT	ko04380
ENSSSCG00000005838	TRAF2	hsa-miR-4792_1ss9GT	ko04380
ENSSSCG00000016140	FZD5	hsa-miR-4792_1ss9GT	ko04390
ENSSSCG00000025374	DVL3	hsa-miR-4792_1ss9GT	ko04390
ENSSSCG00000017306	ITGB3	hsa-miR-4792_1ss9GT	ko04510
ENSSSCG00000017306	ITGB3	hsa-miR-4792_1ss9GT	ko04510
ENSSSCG00000017357	ITGA2B	hsa-miR-4792_1ss9GT	ko04510
ENSSSCG00000011101	ITGB1	hsa-miR-4792_1ss9GT	ko04510
ENSSSCG00000005838	TRAF2	hsa-miR-4792_1ss9GT	ko04622
ENSSSCG00000012495	BTK	hsa-miR-4792_1ss9GT	ko04662
ENSSSCG00000012495	BTK	hsa-miR-4792_1ss9GT	ko04662
ENSSSCG00000012495	BTK	hsa-miR-4792_1ss9GT	ko04662
ENSSSCG00000012495	BTK	hsa-miR-4792_1ss9GT	ko04662
ENSSSCG00000005838	TRAF2	hsa-miR-4792_1ss9GT	ko04668
ENSSSCG00000007520	GNAS	hsa-miR-4792_1ss9GT	ko04923
ENSSSCG00000013564	INSR	hsa-miR-4792_1ss9GT	ko04923
ENSSSCG00000013564	INSR	hsa-miR-4792_1ss9GT	ko04930
ENSSSCG00000016140	FZD5	hsa-miR-4792_1ss9GT	ko05166
ENSSSCG00000025374	DVL3	hsa-miR-4792_1ss9GT	ko05166
ENSSSCG00000008162	IL1R1	hsa-miR-4792_1ss9GT	ko05166
ENSSSCG00000016140	FZD5	hsa-miR-4792_1ss9GT	ko05200
ENSSSCG00000025374	DVL3	hsa-miR-4792_1ss9GT	ko05200
ENSSSCG00000007520	GNAS	hsa-miR-4792_1ss9GT	ko05200
ENSSSCG00000017357	ITGA2B	hsa-miR-4792_1ss9GT	ko05200
ENSSSCG00000011101	ITGB1	hsa-miR-4792_1ss9GT	ko05200
ENSSSCG00000002960	RASGRP4	hsa-miR-4792_1ss9GT	ko05200
ENSSSCG00000008842	KIT	hsa-miR-4792_1ss9GT	ko05200
ENSSSCG00000005838	TRAF2	hsa-miR-4792_1ss9GT	ko05200
ENSSSCG00000001518	ITPR3	hsa-miR-4792_1ss9GT	ko05205
ENSSSCG00000022066	PIAS4	hsa-miR-4792_1ss9GT	ko05205
ENSSSCG00000016140	FZD5	hsa-miR-4792_1ss9GT	ko05205
ENSSSCG00000017306	ITGB3	hsa-miR-4792_1ss9GT	ko05205
ENSSSCG00000011101	ITGB1	hsa-miR-4792_1ss9GT	ko05205
ENSSSCG00000017306	ITGB3	hsa-miR-4792_1ss9GT	ko05205
ENSSSCG00000011412	CACNA2D2	hsa-miR-4792_1ss9GT	ko05410
ENSSSCG00000017357	ITGA2B	hsa-miR-4792_1ss9GT	ko05410
ENSSSCG00000017306	ITGB3	hsa-miR-4792_1ss9GT	ko05410
ENSSSCG00000011101	ITGB1	hsa-miR-4792_1ss9GT	ko05410

ENSSSCG00000017306	ITGB3	hsa-miR-4792_1ss9GT	ko05410
ENSSSCG00000008164	MAP4K4	PC-5p-9551_196	ko04010
ENSSSCG00000014146	RASA1	PC-5p-9551_196	ko04010
ENSSSCG00000020744	DUSP3	PC-5p-9551_196	ko04010
ENSSSCG00000001661	SRF	PC-5p-9551_196	ko04010
ENSSSCG00000011415	MAPKAPK3	PC-5p-9551_196	ko04010
ENSSSCG00000013020	MAP4K2	PC-5p-9551_196	ko04010
ENSSSCG00000016578	FLNC	PC-5p-9551_196	ko04010
ENSSSCG00000017562	CACNA1G	PC-5p-9551_196	ko04010
ENSSSCG00000024813	MAP3K3	PC-5p-9551_196	ko04010
ENSSSCG00000005382	TGFBR1	PC-5p-9551_196	ko04010
ENSSSCG00000001404	NR5A1	PC-5p-9551_196	ko04010
ENSSSCG00000009228	MAPK10	PC-5p-9551_196	ko04010
ENSSSCG00000017330	MAP3K14	PC-5p-9551_196	ko04010
ENSSSCG00000015815	NR5A1	PC-5p-9551_196	ko04010
ENSSSCG00000009228	MAPK10	PC-5p-9551_196	ko04010
ENSSSCG00000010872	AKT3	PC-5p-9551_196	ko04010
ENSSSCG00000002989	AKT2	PC-5p-9551_196	ko04010
ENSSSCG00000006539	SHC1	PC-5p-9551_196	ko04014
ENSSSCG00000009228	MAPK10	PC-5p-9551_196	ko04014
ENSSSCG00000009567	RASA3	PC-5p-9551_196	ko04014
ENSSSCG00000009874	NR5A1	PC-5p-9551_196	ko04014
ENSSSCG00000014146	RASA1	PC-5p-9551_196	ko04014
ENSSSCG00000015383	RAPGEF5	PC-5p-9551_196	ko04014
ENSSSCG00000025768	CALM1	PC-5p-9551_196	ko04014
ENSSSCG00000022545	CALM3	PC-5p-9551_196	ko04014
ENSSSCG00000015815	NR5A1	PC-5p-9551_196	ko04014
ENSSSCG00000006539	SHC1	PC-5p-9551_196	ko04014
ENSSSCG00000010872	AKT3	PC-5p-9551_196	ko04014
ENSSSCG00000002989	AKT2	PC-5p-9551_196	ko04014
ENSSSCG00000009228	MAPK10	PC-5p-9551_196	ko04014
ENSSSCG00000008844	KDR	PC-5p-9551_196	ko04014
ENSSSCG00000011101	ITGB1	PC-5p-9551_196	ko04015
ENSSSCG00000015383	RAPGEF5	PC-5p-9551_196	ko04015
ENSSSCG00000025768	CALM1	PC-5p-9551_196	ko04015
ENSSSCG00000022545	CALM3	PC-5p-9551_196	ko04015
ENSSSCG00000011101	ITGB1	PC-5p-9551_196	ko04015
ENSSSCG00000011101	ITGB1	PC-5p-9551_196	ko04015
ENSSSCG00000027952	ADCY5	PC-5p-9551_196	ko04015
ENSSSCG00000004789	THBS1	PC-5p-9551_196	ko04015
ENSSSCG00000008844	KDR	PC-5p-9551_196	ko04015
ENSSSCG00000015815	NR5A1	PC-5p-9551_196	ko04015
ENSSSCG00000002989	AKT2	PC-5p-9551_196	ko04015

ENSSSCG00000010872	AKT3	PC-5p-9551_196	ko04015
ENSSSCG00000002804	CSNK2A2	PC-5p-9551_196	ko04064
ENSSSCG00000001404	NR5A1	PC-5p-9551_196	ko04064
ENSSSCG00000004154	TNFAIP3	PC-5p-9551_196	ko04064
ENSSSCG00000017330	MAP3K14	PC-5p-9551_196	ko04064
ENSSSCG00000004154	TNFAIP3	PC-5p-9551_196	ko04064
ENSSSCG00000014216	TICAM2	PC-5p-9551_196	ko04064
ENSSSCG00000009228	MAPK10	PC-5p-9551_196	ko04071
ENSSSCG00000009228	MAPK10	PC-5p-9551_196	ko04071
ENSSSCG00000001404	NR5A1	PC-5p-9551_196	ko04071
ENSSSCG00000000293	ITGA5	PC-5p-9551_196	ko04071
ENSSSCG00000030153	SMURF1	PC-5p-9551_196	ko04120
ENSSSCG00000030153	SMURF1	PC-5p-9551_196	ko04144
ENSSSCG00000008844	KDR	PC-5p-9551_196	ko04144
ENSSSCG00000005382	TGFBR1	PC-5p-9551_196	ko04144
ENSSSCG00000008040	TSC2	PC-5p-9551_196	ko04150
ENSSSCG00000001404	NR5A1	PC-5p-9551_196	ko04150
ENSSSCG00000010872	AKT3	PC-5p-9551_196	ko04150
ENSSSCG00000002989	AKT2	PC-5p-9551_196	ko04150
ENSSSCG00000000293	ITGA5	PC-5p-9551_196	ko04151
ENSSSCG00000003154	GYS1	PC-5p-9551_196	ko04151
ENSSSCG00000008040	TSC2	PC-5p-9551_196	ko04151
ENSSSCG00000011101	ITGB1	PC-5p-9551_196	ko04151
ENSSSCG00000017578	ITGA3	PC-5p-9551_196	ko04151
ENSSSCG00000011101	ITGB1	PC-5p-9551_196	ko04151
ENSSSCG00000017578	ITGA3	PC-5p-9551_196	ko04151
ENSSSCG00000011101	ITGB1	PC-5p-9551_196	ko04151
ENSSSCG00000004789	THBS1	PC-5p-9551_196	ko04151
ENSSSCG00000015815	NR5A1	PC-5p-9551_196	ko04151
ENSSSCG00000008844	KDR	PC-5p-9551_196	ko04151
ENSSSCG00000010872	AKT3	PC-5p-9551_196	ko04151
ENSSSCG00000002989	AKT2	PC-5p-9551_196	ko04151
ENSSSCG00000005661	NR5A1	PC-5p-9551_196	ko04210
ENSSSCG00000012657	AIFM1	PC-5p-9551_196	ko04210
ENSSSCG00000017330	MAP3K14	PC-5p-9551_196	ko04210
ENSSSCG00000001404	NR5A1	PC-5p-9551_196	ko04210
ENSSSCG00000010872	AKT3	PC-5p-9551_196	ko04210
ENSSSCG00000025374	DVL3	PC-5p-9551_196	ko04330
ENSSSCG00000004789	THBS1	PC-5p-9551_196	ko04350
ENSSSCG00000009040	SMAD1	PC-5p-9551_196	ko04350
ENSSSCG00000030153	SMURF1	PC-5p-9551_196	ko04350
ENSSSCG00000005382	TGFBR1	PC-5p-9551_196	ko04350
ENSSSCG00000001404	NR5A1	PC-5p-9551_196	ko04350
ENSSSCG00000017330	MAP3K14	PC-5p-9551_196	ko04380

ENSSSCG00000009228	MAPK10	PC-5p-9551_196	ko04380
ENSSSCG00000009228	MAPK10	PC-5p-9551_196	ko04380
ENSSSCG00000001404	NR5A1	PC-5p-9551_196	ko04380
ENSSSCG00000009040	SMAD1	PC-5p-9551_196	ko04390
ENSSSCG00000012913	PPP1CA	PC-5p-9551_196	ko04390
ENSSSCG00000016628	WNT2	PC-5p-9551_196	ko04390
ENSSSCG00000029852	WNT5A	PC-5p-9551_196	ko04390
ENSSSCG00000006780	WNT2B	PC-5p-9551_196	ko04390
ENSSSCG00000025374	DVL3	PC-5p-9551_196	ko04390
ENSSSCG00000005382	TGFBR1	PC-5p-9551_196	ko04390
ENSSSCG00000012913	PPP1CA	PC-5p-9551_196	ko04510
ENSSSCG00000000293	ITGA5	PC-5p-9551_196	ko04510
ENSSSCG00000006539	SHC1	PC-5p-9551_196	ko04510
ENSSSCG00000016578	FLNC	PC-5p-9551_196	ko04510
ENSSSCG00000006539	SHC1	PC-5p-9551_196	ko04510
ENSSSCG00000017578	ITGA3	PC-5p-9551_196	ko04510
ENSSSCG00000017578	ITGA3	PC-5p-9551_196	ko04510
ENSSSCG00000004789	THBS1	PC-5p-9551_196	ko04510
ENSSSCG00000011101	ITGB1	PC-5p-9551_196	ko04510
ENSSSCG00000011101	ITGB1	PC-5p-9551_196	ko04510
ENSSSCG00000011101	ITGB1	PC-5p-9551_196	ko04510
ENSSSCG00000008844	KDR	PC-5p-9551_196	ko04510
ENSSSCG00000009228	MAPK10	PC-5p-9551_196	ko04510
ENSSSCG00000009228	MAPK10	PC-5p-9551_196	ko04510
ENSSSCG00000002989	AKT2	PC-5p-9551_196	ko04510
ENSSSCG00000010872	AKT3	PC-5p-9551_196	ko04510
ENSSSCG00000014220	ATG12	PC-5p-9551_196	ko04622
ENSSSCG00000017330	MAP3K14	PC-5p-9551_196	ko04660
ENSSSCG00000001404	NR5A1	PC-5p-9551_196	ko04668
ENSSSCG00000004154	TNFAIP3	PC-5p-9551_196	ko04668
ENSSSCG00000017330	MAP3K14	PC-5p-9551_196	ko04668
ENSSSCG00000027426	NR5A1	PC-5p-9551_196	ko04668
ENSSSCG00000004154	TNFAIP3	PC-5p-9551_196	ko04668
ENSSSCG00000009228	MAPK10	PC-5p-9551_196	ko04668
ENSSSCG00000010872	AKT3	PC-5p-9551_196	ko04668
ENSSSCG00000002989	AKT2	PC-5p-9551_196	ko04668
ENSSSCG00000009228	MAPK10	PC-5p-9551_196	ko04668
ENSSSCG00000027952	ADCY5	PC-5p-9551_196	ko04923
ENSSSCG00000002989	AKT2	PC-5p-9551_196	ko04923
ENSSSCG00000010872	AKT3	PC-5p-9551_196	ko04923
ENSSSCG00000017562	CACNA1G	PC-5p-9551_196	ko04930
ENSSSCG00000009228	MAPK10	PC-5p-9551_196	ko04930
ENSSSCG00000009228	MAPK10	PC-5p-9551_196	ko04930
ENSSSCG00000001404	NR5A1	PC-5p-9551_196	ko04930

ENSSSCG00000001661	SRF	PC-5p-9551_196	ko05166
ENSSSCG00000006780	WNT2B	PC-5p-9551_196	ko05166
ENSSSCG00000025374	DVL3	PC-5p-9551_196	ko05166
ENSSSCG00000024813	MAP3K3	PC-5p-9551_196	ko05166
ENSSSCG00000016628	WNT2	PC-5p-9551_196	ko05166
ENSSSCG00000029852	WNT5A	PC-5p-9551_196	ko05166
ENSSSCG00000027952	ADCY5	PC-5p-9551_196	ko05166
ENSSSCG00000005382	TGFBR1	PC-5p-9551_196	ko05166
ENSSSCG00000017330	MAP3K14	PC-5p-9551_196	ko05166
ENSSSCG00000001404	NR5A1	PC-5p-9551_196	ko05166
ENSSSCG00000002989	AKT2	PC-5p-9551_196	ko05166
ENSSSCG00000000293	ITGA5	PC-5p-9551_196	ko05166
ENSSSCG00000006780	WNT2B	PC-5p-9551_196	ko05200
ENSSSCG00000016628	WNT2	PC-5p-9551_196	ko05200
ENSSSCG00000025374	DVL3	PC-5p-9551_196	ko05200
ENSSSCG00000029852	WNT5A	PC-5p-9551_196	ko05200
ENSSSCG00000009370	FOXO1	PC-5p-9551_196	ko05200
ENSSSCG00000017578	ITGA3	PC-5p-9551_196	ko05200
ENSSSCG00000027952	ADCY5	PC-5p-9551_196	ko05200
ENSSSCG00000017578	ITGA3	PC-5p-9551_196	ko05200
ENSSSCG00000012275	ARAF	PC-5p-9551_196	ko05200
ENSSSCG00000011101	ITGB1	PC-5p-9551_196	ko05200
ENSSSCG00000011101	ITGB1	PC-5p-9551_196	ko05200
ENSSSCG00000011101	ITGB1	PC-5p-9551_196	ko05200
ENSSSCG00000005382	TGFBR1	PC-5p-9551_196	ko05200
ENSSSCG00000009228	MAPK10	PC-5p-9551_196	ko05200
ENSSSCG00000009370	FOXO1	PC-5p-9551_196	ko05200
ENSSSCG00000015815	NR5A1	PC-5p-9551_196	ko05200
ENSSSCG00000009228	MAPK10	PC-5p-9551_196	ko05200
ENSSSCG00000002989	AKT2	PC-5p-9551_196	ko05200
ENSSSCG00000010872	AKT3	PC-5p-9551_196	ko05200
ENSSSCG00000000293	ITGA5	PC-5p-9551_196	ko05205
ENSSSCG00000012913	PPP1CA	PC-5p-9551_196	ko05205
ENSSSCG00000016578	FLNC	PC-5p-9551_196	ko05205
ENSSSCG00000012275	ARAF	PC-5p-9551_196	ko05205
ENSSSCG00000006780	WNT2B	PC-5p-9551_196	ko05205
ENSSSCG00000016628	WNT2	PC-5p-9551_196	ko05205
ENSSSCG00000029852	WNT5A	PC-5p-9551_196	ko05205
ENSSSCG00000004789	THBS1	PC-5p-9551_196	ko05205
ENSSSCG00000011101	ITGB1	PC-5p-9551_196	ko05205
ENSSSCG00000011101	ITGB1	PC-5p-9551_196	ko05205
ENSSSCG00000011101	ITGB1	PC-5p-9551_196	ko05205
ENSSSCG00000015815	NR5A1	PC-5p-9551_196	ko05205
ENSSSCG00000008844	KDR	PC-5p-9551_196	ko05205

ENSSSCG00000001404	NR5A1	PC-5p-9551_196	ko05205
ENSSSCG00000000293	ITGA5	PC-5p-9551_196	ko05205
ENSSSCG00000012275	ARAF	PC-5p-9551_196	ko05210
ENSSSCG00000009228	MAPK10	PC-5p-9551_196	ko05210
ENSSSCG00000005382	TGFBR1	PC-5p-9551_196	ko05210
ENSSSCG00000012275	ARAF	PC-5p-9551_196	ko05212
ENSSSCG00000005382	TGFBR1	PC-5p-9551_196	ko05212
ENSSSCG00000009228	MAPK10	PC-5p-9551_196	ko05212
ENSSSCG00000009228	MAPK10	PC-5p-9551_196	ko05212
ENSSSCG00000002989	AKT2	PC-5p-9551_196	ko05212
ENSSSCG00000010872	AKT3	PC-5p-9551_196	ko05212
ENSSSCG00000006539	SHC1	PC-5p-9551_196	ko05214
ENSSSCG00000025768	CALM1	PC-5p-9551_196	ko05214
ENSSSCG00000022545	CALM3	PC-5p-9551_196	ko05214
ENSSSCG00000006539	SHC1	PC-5p-9551_196	ko05214
ENSSSCG00000009370	FOXO1	PC-5p-9551_196	ko05215
ENSSSCG00000009370	FOXO1	PC-5p-9551_196	ko05215
ENSSSCG00000012275	ARAF	PC-5p-9551_196	ko05215
ENSSSCG00000015815	NR5A1	PC-5p-9551_196	ko05215
ENSSSCG00000000293	ITGA5	PC-5p-9551_196	ko05215
ENSSSCG00000009840	PRKAB1	PC-5p-9551_196	ko05410
ENSSSCG00000000293	ITGA5	PC-5p-9551_196	ko05410
ENSSSCG00000017578	ITGA3	PC-5p-9551_196	ko05410
ENSSSCG00000017578	ITGA3	PC-5p-9551_196	ko05410
ENSSSCG00000011101	ITGB1	PC-5p-9551_196	ko05410
ENSSSCG00000011101	ITGB1	PC-5p-9551_196	ko05410
ENSSSCG00000011101	ITGB1	PC-5p-9551_196	ko05410
ENSSSCG00000001404	NR5A1	sha-mir-24-1-p3_1ss2GC	ko04010
ENSSSCG00000017412	RAB5C	sha-mir-24-1-p3_1ss2GC	ko04014
ENSSSCG00000001404	NR5A1	sha-mir-24-1-p3_1ss2GC	ko04064
ENSSSCG00000001404	NR5A1	sha-mir-24-1-p3_1ss2GC	ko04071
ENSSSCG00000017412	RAB5C	sha-mir-24-1-p3_1ss2GC	ko04144
ENSSSCG00000001404	NR5A1	sha-mir-24-1-p3_1ss2GC	ko04150
ENSSSCG00000001404	NR5A1	sha-mir-24-1-p3_1ss2GC	ko04210
ENSSSCG00000000233	ACVR1B	sha-mir-24-1-p3_1ss2GC	ko04350
ENSSSCG00000001404	NR5A1	sha-mir-24-1-p3_1ss2GC	ko04350
ENSSSCG00000001404	NR5A1	sha-mir-24-1-p3_1ss2GC	ko04380
ENSSSCG00000000233	ACVR1B	sha-mir-24-1-p3_1ss2GC	ko04622
ENSSSCG00000000233	ACVR1B	sha-mir-24-1-p3_1ss2GC	ko04623
ENSSSCG00000000233	ACVR1B	sha-mir-24-1-p3_1ss2GC	ko04662
ENSSSCG00000001404	NR5A1	sha-mir-24-1-p3_1ss2GC	ko04668
ENSSSCG00000001404	NR5A1	sha-mir-24-1-p3_1ss2GC	ko04930
ENSSSCG00000000233	ACVR1B	sha-mir-24-1-p3_1ss2GC	ko04930
ENSSSCG00000000233	ACVR1B	sha-mir-24-1-p3_1ss2GC	ko05166

ENSSSCG00000001404	NR5A1	sha-mir-24-1-p3_1ss2GC	ko05166
ENSSSCG00000001404	NR5A1	sha-mir-24-1-p3_1ss2GC	ko05205
ENSSSCG00000008845	SRD5A3	ssc-miR-139-3p	ko00140
ENSSSCG00000008845	SRD5A3	ssc-miR-139-3p	ko00140
ENSSSCG00000008845	SRD5A3	ssc-miR-139-3p	ko00140
ENSSSCG00000021560	UBE2I	ssc-miR-139-3p	ko03013
ENSSSCG00000010448	FAS	ssc-miR-139-3p	ko04010
ENSSSCG00000018016	MAP2K4	ssc-miR-139-3p	ko04010
ENSSSCG00000016991	DUSP1	ssc-miR-139-3p	ko04010
ENSSSCG00000015383	RAPGEF5	ssc-miR-139-3p	ko04014
ENSSSCG00000012034	TIAM1	ssc-miR-139-3p	ko04014
ENSSSCG00000015383	RAPGEF5	ssc-miR-139-3p	ko04015
ENSSSCG00000012034	TIAM1	ssc-miR-139-3p	ko04015
ENSSSCG00000020672	F2R	ssc-miR-139-3p	ko04015
ENSSSCG00000021068	TRAF5	ssc-miR-139-3p	ko04064
ENSSSCG00000030172	CD40LG	ssc-miR-139-3p	ko04064
ENSSSCG00000021560	UBE2I	ssc-miR-139-3p	ko04064
ENSSSCG00000012495	BTK	ssc-miR-139-3p	ko04064
ENSSSCG00000012495	BTK	ssc-miR-139-3p	ko04064
ENSSSCG00000012495	BTK	ssc-miR-139-3p	ko04064
ENSSSCG00000021560	UBE2I	ssc-miR-139-3p	ko04120
ENSSSCG00000020672	F2R	ssc-miR-139-3p	ko04144
ENSSSCG00000020672	F2R	ssc-miR-139-3p	ko04151
ENSSSCG00000016887	ITGA2	ssc-miR-139-3p	ko04151
ENSSSCG00000006145	E2F5	ssc-miR-139-3p	ko04151
ENSSSCG00000010448	FAS	ssc-miR-139-3p	ko04210
ENSSSCG00000006145	E2F5	ssc-miR-139-3p	ko04350
ENSSSCG00000012495	BTK	ssc-miR-139-3p	ko04380
ENSSSCG00000012495	BTK	ssc-miR-139-3p	ko04380
ENSSSCG00000012495	BTK	ssc-miR-139-3p	ko04380
ENSSSCG00000012913	PPP1CA	ssc-miR-139-3p	ko04390
ENSSSCG00000005751	COL5A1	ssc-miR-139-3p	ko04510
ENSSSCG00000012913	PPP1CA	ssc-miR-139-3p	ko04510
ENSSSCG00000016887	ITGA2	ssc-miR-139-3p	ko04510
ENSSSCG00000030172	CD40LG	ssc-miR-139-3p	ko04660
ENSSSCG00000012495	BTK	ssc-miR-139-3p	ko04662
ENSSSCG00000012495	BTK	ssc-miR-139-3p	ko04662
ENSSSCG00000012495	BTK	ssc-miR-139-3p	ko04662
ENSSSCG00000010448	FAS	ssc-miR-139-3p	ko04668
ENSSSCG00000018016	MAP2K4	ssc-miR-139-3p	ko04668
ENSSSCG00000018016	MAP2K4	ssc-miR-139-3p	ko05166
ENSSSCG00000021068	TRAF5	ssc-miR-139-3p	ko05200
ENSSSCG00000016887	ITGA2	ssc-miR-139-3p	ko05200
ENSSSCG00000020672	F2R	ssc-miR-139-3p	ko05200

ENSSSCG00000010448	FAS	ssc-miR-139-3p	ko05200
ENSSSCG00000012913	PPP1CA	ssc-miR-139-3p	ko05205
ENSSSCG00000012034	TIAM1	ssc-miR-139-3p	ko05205
ENSSSCG00000016887	ITGA2	ssc-miR-139-3p	ko05205
ENSSSCG00000010448	FAS	ssc-miR-139-3p	ko05205
ENSSSCG00000016887	ITGA2	ssc-miR-139-3p	ko05410
ENSSSCG00000003520	CDC42	ssc-miR-1839-3p_R+2	ko04010
ENSSSCG00000008164	MAP4K4	ssc-miR-1839-3p_R+2	ko04010
ENSSSCG00000014146	RASA1	ssc-miR-1839-3p_R+2	ko04010
ENSSSCG00000003520	CDC42	ssc-miR-1839-3p_R+2	ko04010
ENSSSCG00000022482	DDIT3	ssc-miR-1839-3p_R+2	ko04010
ENSSSCG00000004110	TAB2	ssc-miR-1839-3p_R+2	ko04010
ENSSSCG00000028968	RAC1	ssc-miR-1839-3p_R+2	ko04010
ENSSSCG00000003520	CDC42	ssc-miR-1839-3p_R+2	ko04014
ENSSSCG00000006530	EFNA1	ssc-miR-1839-3p_R+2	ko04014
ENSSSCG00000014146	RASA1	ssc-miR-1839-3p_R+2	ko04014
ENSSSCG00000028968	RAC1	ssc-miR-1839-3p_R+2	ko04014
ENSSSCG00000025455	RALB	ssc-miR-1839-3p_R+2	ko04014
ENSSSCG00000025768	CALM1	ssc-miR-1839-3p_R+2	ko04014
ENSSSCG00000003520	CDC42	ssc-miR-1839-3p_R+2	ko04014
ENSSSCG00000006530	EFNA1	ssc-miR-1839-3p_R+2	ko04015
ENSSSCG00000025768	CALM1	ssc-miR-1839-3p_R+2	ko04015
ENSSSCG00000025455	RALB	ssc-miR-1839-3p_R+2	ko04015
ENSSSCG00000003520	CDC42	ssc-miR-1839-3p_R+2	ko04015
ENSSSCG00000003520	CDC42	ssc-miR-1839-3p_R+2	ko04015
ENSSSCG00000028968	RAC1	ssc-miR-1839-3p_R+2	ko04015
ENSSSCG00000004110	TAB2	ssc-miR-1839-3p_R+2	ko04064
ENSSSCG00000028968	RAC1	ssc-miR-1839-3p_R+2	ko04071
ENSSSCG00000000529	DNM1L	ssc-miR-1839-3p_R+2	ko04071
ENSSSCG00000003520	CDC42	ssc-miR-1839-3p_R+2	ko04144
ENSSSCG00000003520	CDC42	ssc-miR-1839-3p_R+2	ko04144
ENSSSCG00000010509	PIK3AP1	ssc-miR-1839-3p_R+2	ko04151
ENSSSCG00000016027	ITGAV	ssc-miR-1839-3p_R+2	ko04151
ENSSSCG00000028968	RAC1	ssc-miR-1839-3p_R+2	ko04151
ENSSSCG00000004110	TAB2	ssc-miR-1839-3p_R+2	ko04380
ENSSSCG00000028968	RAC1	ssc-miR-1839-3p_R+2	ko04380
ENSSSCG00000010894	TP53BP2	ssc-miR-1839-3p_R+2	ko04390
ENSSSCG00000006780	WNT2B	ssc-miR-1839-3p_R+2	ko04390
ENSSSCG00000016027	ITGAV	ssc-miR-1839-3p_R+2	ko04510
ENSSSCG00000003520	CDC42	ssc-miR-1839-3p_R+2	ko04510
ENSSSCG00000003520	CDC42	ssc-miR-1839-3p_R+2	ko04510
ENSSSCG00000028968	RAC1	ssc-miR-1839-3p_R+2	ko04510
ENSSSCG00000003520	CDC42	ssc-miR-1839-3p_R+2	ko04660
ENSSSCG00000010509	PIK3AP1	ssc-miR-1839-3p_R+2	ko04662

ENSSSCG00000028968	RAC1	ssc-miR-1839-3p_R+2	ko04662
ENSSSCG00000000529	DNM1L	ssc-miR-1839-3p_R+2	ko04668
ENSSSCG00000004110	TAB2	ssc-miR-1839-3p_R+2	ko04668
ENSSSCG00000000529	DNM1L	ssc-miR-1839-3p_R+2	ko04668
ENSSSCG00000000529	DNM1L	ssc-miR-1839-3p_R+2	ko04930
ENSSSCG00000006780	WNT2B	ssc-miR-1839-3p_R+2	ko05166
ENSSSCG00000000529	DNM1L	ssc-miR-1839-3p_R+2	ko05166
ENSSSCG00000006780	WNT2B	ssc-miR-1839-3p_R+2	ko05200
ENSSSCG00000016027	ITGAV	ssc-miR-1839-3p_R+2	ko05200
ENSSSCG00000025455	RALB	ssc-miR-1839-3p_R+2	ko05200
ENSSSCG00000003520	CDC42	ssc-miR-1839-3p_R+2	ko05200
ENSSSCG00000003520	CDC42	ssc-miR-1839-3p_R+2	ko05200
ENSSSCG00000028968	RAC1	ssc-miR-1839-3p_R+2	ko05200
ENSSSCG00000006780	WNT2B	ssc-miR-1839-3p_R+2	ko05205
ENSSSCG00000014362	HBEGF	ssc-miR-1839-3p_R+2	ko05205
ENSSSCG00000016027	ITGAV	ssc-miR-1839-3p_R+2	ko05205
ENSSSCG00000003520	CDC42	ssc-miR-1839-3p_R+2	ko05205
ENSSSCG00000003520	CDC42	ssc-miR-1839-3p_R+2	ko05205
ENSSSCG00000028968	RAC1	ssc-miR-1839-3p_R+2	ko05205
ENSSSCG00000000529	DNM1L	ssc-miR-1839-3p_R+2	ko05205
ENSSSCG00000003520	CDC42	ssc-miR-1839-3p_R+2	ko05211
ENSSSCG00000025455	RALB	ssc-miR-1839-3p_R+2	ko05212
ENSSSCG00000003520	CDC42	ssc-miR-1839-3p_R+2	ko05212
ENSSSCG00000003520	CDC42	ssc-miR-1839-3p_R+2	ko05212
ENSSSCG00000028968	RAC1	ssc-miR-1839-3p_R+2	ko05212
ENSSSCG00000025768	CALM1	ssc-miR-1839-3p_R+2	ko05214
ENSSSCG00000016027	ITGAV	ssc-miR-1839-3p_R+2	ko05410
ENSSSCG00000012163	RPS6KA3	ssc-miR-190b	ko04010
ENSSSCG00000016878	FGF10	ssc-miR-190b	ko04010
ENSSSCG00000015403	HGF	ssc-miR-190b	ko04014
ENSSSCG00000015770	VEGFC	ssc-miR-190b	ko04014
ENSSSCG00000016878	FGF10	ssc-miR-190b	ko04014
ENSSSCG00000007356	PLCG1	ssc-miR-190b	ko04015
ENSSSCG00000015403	HGF	ssc-miR-190b	ko04015
ENSSSCG00000015770	VEGFC	ssc-miR-190b	ko04015
ENSSSCG00000016878	FGF10	ssc-miR-190b	ko04015
ENSSSCG00000004154	TNFAIP3	ssc-miR-190b	ko04064
ENSSSCG00000004154	TNFAIP3	ssc-miR-190b	ko04064
ENSSSCG00000007356	PLCG1	ssc-miR-190b	ko04064
ENSSSCG00000000529	DNM1L	ssc-miR-190b	ko04071
ENSSSCG00000012163	RPS6KA3	ssc-miR-190b	ko04150
ENSSSCG00000017670	RPS6KB1	ssc-miR-190b	ko04150
ENSSSCG00000017670	RPS6KB1	ssc-miR-190b	ko04151
ENSSSCG00000004896	PHLPP1	ssc-miR-190b	ko04151

ENSSSCG00000015403	HGF	ssc-miR-190b	ko04151
ENSSSCG00000015770	VEGFC	ssc-miR-190b	ko04151
ENSSSCG00000016878	FGF10	ssc-miR-190b	ko04151
ENSSSCG00000020906	TNFSF10	ssc-miR-190b	ko04210
ENSSSCG00000017670	RPS6KB1	ssc-miR-190b	ko04350
ENSSSCG00000006780	WNT2B	ssc-miR-190b	ko04390
ENSSSCG00000015403	HGF	ssc-miR-190b	ko04510
ENSSSCG00000015770	VEGFC	ssc-miR-190b	ko04510
ENSSSCG00000007356	PLCG1	ssc-miR-190b	ko04660
ENSSSCG00000000529	DNM1L	ssc-miR-190b	ko04668
ENSSSCG00000004154	TNFAIP3	ssc-miR-190b	ko04668
ENSSSCG00000000529	DNM1L	ssc-miR-190b	ko04668
ENSSSCG00000004154	TNFAIP3	ssc-miR-190b	ko04668
ENSSSCG00000000529	DNM1L	ssc-miR-190b	ko04930
ENSSSCG00000006780	WNT2B	ssc-miR-190b	ko05166
ENSSSCG00000020906	TNFSF10	ssc-miR-190b	ko05166
ENSSSCG00000006780	WNT2B	ssc-miR-190b	ko05200
ENSSSCG00000007356	PLCG1	ssc-miR-190b	ko05200
ENSSSCG00000015403	HGF	ssc-miR-190b	ko05200
ENSSSCG00000015770	VEGFC	ssc-miR-190b	ko05200
ENSSSCG00000016878	FGF10	ssc-miR-190b	ko05200
ENSSSCG00000025777	ESR1	ssc-miR-190b	ko05205
ENSSSCG00000025777	ESR1	ssc-miR-190b	ko05205
ENSSSCG00000006780	WNT2B	ssc-miR-190b	ko05205
ENSSSCG00000017670	RPS6KB1	ssc-miR-190b	ko05205
ENSSSCG00000007356	PLCG1	ssc-miR-190b	ko05205
ENSSSCG00000015403	HGF	ssc-miR-190b	ko05205
ENSSSCG00000000529	DNM1L	ssc-miR-190b	ko05205
ENSSSCG00000015403	HGF	ssc-miR-190b	ko05211
ENSSSCG00000007356	PLCG1	ssc-miR-190b	ko05214
ENSSSCG00000007356	PLCG1	ssc-miR-190b	ko05223
ENSSSCG00000028420	EIF4E	ssc-miR-192	ko03013
ENSSSCG00000016918	MAP3K1	ssc-miR-192	ko04010
ENSSSCG00000029828	RAB5B	ssc-miR-192	ko04014
ENSSSCG00000016918	MAP3K1	ssc-miR-192	ko04120
ENSSSCG00000029828	RAB5B	ssc-miR-192	ko04144
ENSSSCG00000003194	NR5A1	ssc-miR-192	ko04150
ENSSSCG00000028420	EIF4E	ssc-miR-192	ko04150
ENSSSCG00000016832	IL7R	ssc-miR-192	ko04151
ENSSSCG00000028420	EIF4E	ssc-miR-192	ko04151
ENSSSCG00000009088	IL2	ssc-miR-192	ko04151
ENSSSCG00000016628	WNT2	ssc-miR-192	ko04390
ENSSSCG00000029852	WNT5A	ssc-miR-192	ko04390
ENSSSCG00000016918	MAP3K1	ssc-miR-192	ko04622

ENSSSCG00000009088	IL2	ssc-miR-192	ko04660
ENSSSCG00000009051	IL15	ssc-miR-192	ko04668
ENSSSCG00000009051	IL15	ssc-miR-192	ko05166
ENSSSCG00000009088	IL2	ssc-miR-192	ko05166
ENSSSCG00000016628	WNT2	ssc-miR-192	ko05166
ENSSSCG00000016918	MAP3K1	ssc-miR-192	ko05166
ENSSSCG00000029852	WNT5A	ssc-miR-192	ko05166
ENSSSCG00000016628	WNT2	ssc-miR-192	ko05200
ENSSSCG00000029852	WNT5A	ssc-miR-192	ko05200
ENSSSCG00000014362	HBEGF	ssc-miR-192	ko05205
ENSSSCG00000016628	WNT2	ssc-miR-192	ko05205
ENSSSCG00000029852	WNT5A	ssc-miR-192	ko05205
ENSSSCG00000014127	RASGRF2	ssc-miR-194a_R+2	ko04010
ENSSSCG00000014878	PAK1	ssc-miR-194a_R+2	ko04010
ENSSSCG00000014878	PAK1	ssc-miR-194a_R+2	ko04010
ENSSSCG00000014878	PAK1	ssc-miR-194a_R+2	ko04010
ENSSSCG00000016991	DUSP1	ssc-miR-194a_R+2	ko04010
ENSSSCG00000028968	RAC1	ssc-miR-194a_R+2	ko04010
ENSSSCG00000027443	MRAS	ssc-miR-194a_R+2	ko04010
ENSSSCG00000004018	AFDN	ssc-miR-194a_R+2	ko04014
ENSSSCG00000014127	RASGRF2	ssc-miR-194a_R+2	ko04014
ENSSSCG00000014878	PAK1	ssc-miR-194a_R+2	ko04014
ENSSSCG00000028968	RAC1	ssc-miR-194a_R+2	ko04014
ENSSSCG00000027443	MRAS	ssc-miR-194a_R+2	ko04014
ENSSSCG00000014878	PAK1	ssc-miR-194a_R+2	ko04014
ENSSSCG00000014878	PAK1	ssc-miR-194a_R+2	ko04014
ENSSSCG00000007058	PLCB4	ssc-miR-194a_R+2	ko04015
ENSSSCG00000004018	AFDN	ssc-miR-194a_R+2	ko04015
ENSSSCG00000027443	MRAS	ssc-miR-194a_R+2	ko04015
ENSSSCG00000020672	F2R	ssc-miR-194a_R+2	ko04015
ENSSSCG00000004789	THBS1	ssc-miR-194a_R+2	ko04015
ENSSSCG00000028968	RAC1	ssc-miR-194a_R+2	ko04015
ENSSSCG00000004917	MALT1	ssc-miR-194a_R+2	ko04064
ENSSSCG00000007058	PLCB4	ssc-miR-194a_R+2	ko04071
ENSSSCG00000028968	RAC1	ssc-miR-194a_R+2	ko04071
ENSSSCG00000000529	DNM1L	ssc-miR-194a_R+2	ko04071
ENSSSCG00000003863	ZFYVE9	ssc-miR-194a_R+2	ko04144
ENSSSCG00000020672	F2R	ssc-miR-194a_R+2	ko04144
ENSSSCG00000016832	IL7R	ssc-miR-194a_R+2	ko04151
ENSSSCG00000020672	F2R	ssc-miR-194a_R+2	ko04151
ENSSSCG00000004789	THBS1	ssc-miR-194a_R+2	ko04151
ENSSSCG00000006095	CCNE2	ssc-miR-194a_R+2	ko04151
ENSSSCG00000028968	RAC1	ssc-miR-194a_R+2	ko04151
ENSSSCG00000003863	ZFYVE9	ssc-miR-194a_R+2	ko04350

ENSSSCG00000004789	THBS1	ssc-miR-194a_R+2	ko04350
ENSSSCG00000007501	BMP7	ssc-miR-194a_R+2	ko04350
ENSSSCG00000028968	RAC1	ssc-miR-194a_R+2	ko04380
ENSSSCG00000007501	BMP7	ssc-miR-194a_R+2	ko04390
ENSSSCG00000029852	WNT5A	ssc-miR-194a_R+2	ko04390
ENSSSCG00000014878	PAK1	ssc-miR-194a_R+2	ko04510
ENSSSCG00000014878	PAK1	ssc-miR-194a_R+2	ko04510
ENSSSCG00000014878	PAK1	ssc-miR-194a_R+2	ko04510
ENSSSCG00000004789	THBS1	ssc-miR-194a_R+2	ko04510
ENSSSCG00000028968	RAC1	ssc-miR-194a_R+2	ko04510
ENSSSCG00000004917	MALT1	ssc-miR-194a_R+2	ko04660
ENSSSCG00000014878	PAK1	ssc-miR-194a_R+2	ko04660
ENSSSCG00000014878	PAK1	ssc-miR-194a_R+2	ko04660
ENSSSCG00000014878	PAK1	ssc-miR-194a_R+2	ko04660
ENSSSCG00000028968	RAC1	ssc-miR-194a_R+2	ko04662
ENSSSCG00000000529	DNM1L	ssc-miR-194a_R+2	ko04668
ENSSSCG00000001050	EDN1	ssc-miR-194a_R+2	ko04668
ENSSSCG00000000529	DNM1L	ssc-miR-194a_R+2	ko04668
ENSSSCG00000029852	WNT5A	ssc-miR-194a_R+2	ko05166
ENSSSCG00000027443	MRAS	ssc-miR-194a_R+2	ko05166
ENSSSCG00000006095	CCNE2	ssc-miR-194a_R+2	ko05200
ENSSSCG00000029852	WNT5A	ssc-miR-194a_R+2	ko05200
ENSSSCG00000007058	PLCB4	ssc-miR-194a_R+2	ko05200
ENSSSCG00000020672	F2R	ssc-miR-194a_R+2	ko05200
ENSSSCG00000028968	RAC1	ssc-miR-194a_R+2	ko05200
ENSSSCG00000014878	PAK1	ssc-miR-194a_R+2	ko05205
ENSSSCG00000029852	WNT5A	ssc-miR-194a_R+2	ko05205
ENSSSCG00000014878	PAK1	ssc-miR-194a_R+2	ko05205
ENSSSCG00000014878	PAK1	ssc-miR-194a_R+2	ko05205
ENSSSCG00000004789	THBS1	ssc-miR-194a_R+2	ko05205
ENSSSCG00000027443	MRAS	ssc-miR-194a_R+2	ko05205
ENSSSCG00000028968	RAC1	ssc-miR-194a_R+2	ko05205
ENSSSCG00000000529	DNM1L	ssc-miR-194a_R+2	ko05205
ENSSSCG00000014878	PAK1	ssc-miR-194a_R+2	ko05211
ENSSSCG00000014878	PAK1	ssc-miR-194a_R+2	ko05211
ENSSSCG00000028968	RAC1	ssc-miR-194a_R+2	ko05212
ENSSSCG00000006095	CCNE2	ssc-miR-194a_R+2	ko05215
ENSSSCG00000000529	DNM1L	ssc-miR-194a_R+2	ko05215
ENSSSCG00000001050	EDN1	ssc-miR-194a_R+2	ko05410
ENSSSCG00000014127	RASGRF2	ssc-miR-194b-5p_1ss10GA	ko04010
ENSSSCG00000014878	PAK1	ssc-miR-194b-5p_1ss10GA	ko04010
ENSSSCG00000014878	PAK1	ssc-miR-194b-5p_1ss10GA	ko04010
ENSSSCG00000028968	RAC1	ssc-miR-194b-5p_1ss10GA	ko04010
ENSSSCG00000027443	MRAS	ssc-miR-194b-5p_1ss10GA	ko04010

ENSSSCG00000004018	AFDN	ssc-miR-194b-5p_1ss10GA	ko04014
ENSSSCG00000014127	RASGRF2	ssc-miR-194b-5p_1ss10GA	ko04014
ENSSSCG00000014878	PAK1	ssc-miR-194b-5p_1ss10GA	ko04014
ENSSSCG00000027443	MRAS	ssc-miR-194b-5p_1ss10GA	ko04014
ENSSSCG00000014878	PAK1	ssc-miR-194b-5p_1ss10GA	ko04014
ENSSSCG00000014878	PAK1	ssc-miR-194b-5p_1ss10GA	ko04014
ENSSSCG00000007058	PLCB4	ssc-miR-194b-5p_1ss10GA	ko04015
ENSSSCG00000004018	AFDN	ssc-miR-194b-5p_1ss10GA	ko04015
ENSSSCG00000027443	MRAS	ssc-miR-194b-5p_1ss10GA	ko04015
ENSSSCG00000020672	F2R	ssc-miR-194b-5p_1ss10GA	ko04015
ENSSSCG00000004789	THBS1	ssc-miR-194b-5p_1ss10GA	ko04015
ENSSSCG00000028968	RAC1	ssc-miR-194b-5p_1ss10GA	ko04015
ENSSSCG00000004917	MALT1	ssc-miR-194b-5p_1ss10GA	ko04064
ENSSSCG00000007058	PLCB4	ssc-miR-194b-5p_1ss10GA	ko04071
ENSSSCG00000028968	RAC1	ssc-miR-194b-5p_1ss10GA	ko04071
ENSSSCG00000020672	F2R	ssc-miR-194b-5p_1ss10GA	ko04144
ENSSSCG00000016832	IL7R	ssc-miR-194b-5p_1ss10GA	ko04151
ENSSSCG00000020672	F2R	ssc-miR-194b-5p_1ss10GA	ko04151
ENSSSCG00000004789	THBS1	ssc-miR-194b-5p_1ss10GA	ko04151
ENSSSCG00000028968	RAC1	ssc-miR-194b-5p_1ss10GA	ko04151
ENSSSCG00000003863	ZFYVE9	ssc-miR-194b-5p_1ss10GA	ko04350
ENSSSCG00000004789	THBS1	ssc-miR-194b-5p_1ss10GA	ko04350
ENSSSCG00000007501	BMP7	ssc-miR-194b-5p_1ss10GA	ko04350
ENSSSCG00000028968	RAC1	ssc-miR-194b-5p_1ss10GA	ko04380
ENSSSCG00000007501	BMP7	ssc-miR-194b-5p_1ss10GA	ko04390
ENSSSCG00000029852	WNT5A	ssc-miR-194b-5p_1ss10GA	ko04390
ENSSSCG00000014878	PAK1	ssc-miR-194b-5p_1ss10GA	ko04510
ENSSSCG00000014878	PAK1	ssc-miR-194b-5p_1ss10GA	ko04510
ENSSSCG00000014878	PAK1	ssc-miR-194b-5p_1ss10GA	ko04510
ENSSSCG00000004789	THBS1	ssc-miR-194b-5p_1ss10GA	ko04510
ENSSSCG00000028968	RAC1	ssc-miR-194b-5p_1ss10GA	ko04510
ENSSSCG00000004917	MALT1	ssc-miR-194b-5p_1ss10GA	ko04660
ENSSSCG00000014878	PAK1	ssc-miR-194b-5p_1ss10GA	ko04660
ENSSSCG00000014878	PAK1	ssc-miR-194b-5p_1ss10GA	ko04660
ENSSSCG00000028968	RAC1	ssc-miR-194b-5p_1ss10GA	ko04662
ENSSSCG00000000529	DNM1L	ssc-miR-194b-5p_1ss10GA	ko04668
ENSSSCG00000001050	EDN1	ssc-miR-194b-5p_1ss10GA	ko04668
ENSSSCG00000028968	RAC1	ssc-miR-194b-5p_1ss10GA	ko04668
ENSSSCG00000000529	DNM1L	ssc-miR-194b-5p_1ss10GA	ko04668
ENSSSCG00000029852	WNT5A	ssc-miR-194b-5p_1ss10GA	ko05166
ENSSSCG00000027443	MRAS	ssc-miR-194b-5p_1ss10GA	ko05166
ENSSSCG00000006095	CCNE2	ssc-miR-194b-5p_1ss10GA	ko05200
ENSSSCG00000029852	WNT5A	ssc-miR-194b-5p_1ss10GA	ko05200
ENSSSCG00000007058	PLCB4	ssc-miR-194b-5p_1ss10GA	ko05200

ENSSSCG00000020672	F2R	ssc-miR-194b-5p_1ss10GA	ko05200
ENSSSCG00000028968	RAC1	ssc-miR-194b-5p_1ss10GA	ko05200
ENSSSCG00000014878	PAK1	ssc-miR-194b-5p_1ss10GA	ko05205
ENSSSCG00000029852	WNT5A	ssc-miR-194b-5p_1ss10GA	ko05205
ENSSSCG00000014878	PAK1	ssc-miR-194b-5p_1ss10GA	ko05205
ENSSSCG00000014878	PAK1	ssc-miR-194b-5p_1ss10GA	ko05205
ENSSSCG00000004789	THBS1	ssc-miR-194b-5p_1ss10GA	ko05205
ENSSSCG00000027443	MRAS	ssc-miR-194b-5p_1ss10GA	ko05205
ENSSSCG00000028968	RAC1	ssc-miR-194b-5p_1ss10GA	ko05205
ENSSSCG00000014878	PAK1	ssc-miR-194b-5p_1ss10GA	ko05211
ENSSSCG00000014878	PAK1	ssc-miR-194b-5p_1ss10GA	ko05211
ENSSSCG00000014878	PAK1	ssc-miR-194b-5p_1ss10GA	ko05211
ENSSSCG00000028968	RAC1	ssc-miR-194b-5p_1ss10GA	ko05212
ENSSSCG00000006095	CCNE2	ssc-miR-194b-5p_1ss10GA	ko05215
ENSSSCG00000001050	EDN1	ssc-miR-194b-5p_1ss10GA	ko05410
ENSSSCG00000028420	EIF4E	ssc-miR-215_R+1	ko03013
ENSSSCG00000016918	MAP3K1	ssc-miR-215_R+1	ko04010
ENSSSCG00000004110	TAB2	ssc-miR-215_R+1	ko04010
ENSSSCG00000029828	RAB5B	ssc-miR-215_R+1	ko04014
ENSSSCG00000004110	TAB2	ssc-miR-215_R+1	ko04064
ENSSSCG00000016918	MAP3K1	ssc-miR-215_R+1	ko04120
ENSSSCG00000029828	RAB5B	ssc-miR-215_R+1	ko04144
ENSSSCG00000003194	NR5A1	ssc-miR-215_R+1	ko04150
ENSSSCG00000028420	EIF4E	ssc-miR-215_R+1	ko04150
ENSSSCG00000016832	IL7R	ssc-miR-215_R+1	ko04151
ENSSSCG00000028420	EIF4E	ssc-miR-215_R+1	ko04151
ENSSSCG00000009088	IL2	ssc-miR-215_R+1	ko04151
ENSSSCG00000004110	TAB2	ssc-miR-215_R+1	ko04380
ENSSSCG00000016628	WNT2	ssc-miR-215_R+1	ko04390
ENSSSCG00000029852	WNT5A	ssc-miR-215_R+1	ko04390
ENSSSCG00000016918	MAP3K1	ssc-miR-215_R+1	ko04622
ENSSSCG00000009088	IL2	ssc-miR-215_R+1	ko04660
ENSSSCG00000004110	TAB2	ssc-miR-215_R+1	ko04668
ENSSSCG00000009051	IL15	ssc-miR-215_R+1	ko04668
ENSSSCG00000009051	IL15	ssc-miR-215_R+1	ko05166
ENSSSCG00000009088	IL2	ssc-miR-215_R+1	ko05166
ENSSSCG00000016628	WNT2	ssc-miR-215_R+1	ko05166
ENSSSCG00000016918	MAP3K1	ssc-miR-215_R+1	ko05166
ENSSSCG00000029852	WNT5A	ssc-miR-215_R+1	ko05166
ENSSSCG00000016628	WNT2	ssc-miR-215_R+1	ko05200
ENSSSCG00000029852	WNT5A	ssc-miR-215_R+1	ko05200
ENSSSCG00000016628	WNT2	ssc-miR-215_R+1	ko05205
ENSSSCG00000029852	WNT5A	ssc-miR-215_R+1	ko05205
ENSSSCG00000004022	RPS6KA2	ssc-mir-4332-p5_1ss18CA	ko04010

ENSSSCG00000013269	MAPK8IP1	ssc-mir-4332-p5_1ss18CA	ko04010
ENSSSCG00000010872	AKT3	ssc-mir-4332-p5_1ss18CA	ko04010
ENSSSCG00000007030	IKBKB	ssc-mir-4332-p5_1ss18CA	ko04010
ENSSSCG00000012981	NR5A1	ssc-mir-4332-p5_1ss18CA	ko04010
ENSSSCG00000001506	NR5A1	ssc-mir-4332-p5_1ss18CA	ko04014
ENSSSCG00000007030	IKBKB	ssc-mir-4332-p5_1ss18CA	ko04014
ENSSSCG00000012981	NR5A1	ssc-mir-4332-p5_1ss18CA	ko04014
ENSSSCG00000013033	BAD	ssc-mir-4332-p5_1ss18CA	ko04014
ENSSSCG00000021865	INHBA	ssc-mir-4332-p5_1ss18CA	ko04014
ENSSSCG00000010872	AKT3	ssc-mir-4332-p5_1ss18CA	ko04014
ENSSSCG00000007520	GNAS	ssc-mir-4332-p5_1ss18CA	ko04015
ENSSSCG00000007520	GNAS	ssc-mir-4332-p5_1ss18CA	ko04015
ENSSSCG00000007520	GNAS	ssc-mir-4332-p5_1ss18CA	ko04015
ENSSSCG00000007520	GNAS	ssc-mir-4332-p5_1ss18CA	ko04015
ENSSSCG00000007520	GNAS	ssc-mir-4332-p5_1ss18CA	ko04015
ENSSSCG00000007520	GNAS	ssc-mir-4332-p5_1ss18CA	ko04015
ENSSSCG00000007520	GNAS	ssc-mir-4332-p5_1ss18CA	ko04015
ENSSSCG00000010872	AKT3	ssc-mir-4332-p5_1ss18CA	ko04015
ENSSSCG00000001009	RIPK1	ssc-mir-4332-p5_1ss18CA	ko04064
ENSSSCG00000002804	CSNK2A2	ssc-mir-4332-p5_1ss18CA	ko04064
ENSSSCG00000007030	IKBKB	ssc-mir-4332-p5_1ss18CA	ko04064
ENSSSCG00000012981	NR5A1	ssc-mir-4332-p5_1ss18CA	ko04064
ENSSSCG00000000233	ACVR1B	ssc-mir-4332-p5_1ss18CA	ko04071
ENSSSCG00000004022	RPS6KA2	ssc-mir-4332-p5_1ss18CA	ko04150
ENSSSCG00000010872	AKT3	ssc-mir-4332-p5_1ss18CA	ko04150
ENSSSCG00000007030	IKBKB	ssc-mir-4332-p5_1ss18CA	ko04150
ENSSSCG00000009545	COL4A2	ssc-mir-4332-p5_1ss18CA	ko04151
ENSSSCG00000017482	CSF3	ssc-mir-4332-p5_1ss18CA	ko04151
ENSSSCG00000000293	ITGA5	ssc-mir-4332-p5_1ss18CA	ko04151
ENSSSCG00000000293	ITGA5	ssc-mir-4332-p5_1ss18CA	ko04151
ENSSSCG00000013033	BAD	ssc-mir-4332-p5_1ss18CA	ko04151
ENSSSCG00000007030	IKBKB	ssc-mir-4332-p5_1ss18CA	ko04151
ENSSSCG00000012981	NR5A1	ssc-mir-4332-p5_1ss18CA	ko04151
ENSSSCG00000010872	AKT3	ssc-mir-4332-p5_1ss18CA	ko04151
ENSSSCG00000005661	NR5A1	ssc-mir-4332-p5_1ss18CA	ko04210
ENSSSCG00000001009	RIPK1	ssc-mir-4332-p5_1ss18CA	ko04210
ENSSSCG00000013033	BAD	ssc-mir-4332-p5_1ss18CA	ko04210
ENSSSCG00000012981	NR5A1	ssc-mir-4332-p5_1ss18CA	ko04210
ENSSSCG00000010872	AKT3	ssc-mir-4332-p5_1ss18CA	ko04210
ENSSSCG00000007030	IKBKB	ssc-mir-4332-p5_1ss18CA	ko04210
ENSSSCG00000000233	ACVR1B	ssc-mir-4332-p5_1ss18CA	ko04350
ENSSSCG00000001027	BMP6	ssc-mir-4332-p5_1ss18CA	ko04350
ENSSSCG00000021865	INHBA	ssc-mir-4332-p5_1ss18CA	ko04350
ENSSSCG00000007030	IKBKB	ssc-mir-4332-p5_1ss18CA	ko04380

ENSSSCG00000000107	CSNK1E	ssc-mir-4332-p5_1ss18CA	ko04390
ENSSSCG00000001027	BMP6	ssc-mir-4332-p5_1ss18CA	ko04390
ENSSSCG00000014339	CTNNA1	ssc-mir-4332-p5_1ss18CA	ko04390
ENSSSCG00000000293	ITGA5	ssc-mir-4332-p5_1ss18CA	ko04510
ENSSSCG00000000293	ITGA5	ssc-mir-4332-p5_1ss18CA	ko04510
ENSSSCG00000009545	COL4A2	ssc-mir-4332-p5_1ss18CA	ko04510
ENSSSCG00000013033	BAD	ssc-mir-4332-p5_1ss18CA	ko04510
ENSSSCG00000010872	AKT3	ssc-mir-4332-p5_1ss18CA	ko04510
ENSSSCG00000001009	RIPK1	ssc-mir-4332-p5_1ss18CA	ko04622
ENSSSCG00000001009	RIPK1	ssc-mir-4332-p5_1ss18CA	ko04623
ENSSSCG00000012981	NR5A1	ssc-mir-4332-p5_1ss18CA	ko04623
ENSSSCG00000012981	NR5A1	ssc-mir-4332-p5_1ss18CA	ko04660
ENSSSCG00000001009	RIPK1	ssc-mir-4332-p5_1ss18CA	ko04668
ENSSSCG00000007030	IKBKB	ssc-mir-4332-p5_1ss18CA	ko04668
ENSSSCG00000012981	NR5A1	ssc-mir-4332-p5_1ss18CA	ko04668
ENSSSCG00000010872	AKT3	ssc-mir-4332-p5_1ss18CA	ko04668
ENSSSCG00000007520	GNAS	ssc-mir-4332-p5_1ss18CA	ko04923
ENSSSCG00000007520	GNAS	ssc-mir-4332-p5_1ss18CA	ko04923
ENSSSCG00000007520	GNAS	ssc-mir-4332-p5_1ss18CA	ko04923
ENSSSCG00000007520	GNAS	ssc-mir-4332-p5_1ss18CA	ko04923
ENSSSCG00000007520	GNAS	ssc-mir-4332-p5_1ss18CA	ko04923
ENSSSCG00000007520	GNAS	ssc-mir-4332-p5_1ss18CA	ko04923
ENSSSCG00000007520	GNAS	ssc-mir-4332-p5_1ss18CA	ko04923
ENSSSCG00000010872	AKT3	ssc-mir-4332-p5_1ss18CA	ko04923
ENSSSCG00000012981	NR5A1	ssc-mir-4332-p5_1ss18CA	ko05166
ENSSSCG00000007030	IKBKB	ssc-mir-4332-p5_1ss18CA	ko05166
ENSSSCG00000000233	ACVR1B	ssc-mir-4332-p5_1ss18CA	ko05166
ENSSSCG00000014339	CTNNA1	ssc-mir-4332-p5_1ss18CA	ko05200
ENSSSCG00000007520	GNAS	ssc-mir-4332-p5_1ss18CA	ko05200
ENSSSCG00000007520	GNAS	ssc-mir-4332-p5_1ss18CA	ko05200
ENSSSCG00000007520	GNAS	ssc-mir-4332-p5_1ss18CA	ko05200
ENSSSCG00000007520	GNAS	ssc-mir-4332-p5_1ss18CA	ko05200
ENSSSCG00000007520	GNAS	ssc-mir-4332-p5_1ss18CA	ko05200
ENSSSCG00000007520	GNAS	ssc-mir-4332-p5_1ss18CA	ko05200
ENSSSCG00000007520	GNAS	ssc-mir-4332-p5_1ss18CA	ko05200
ENSSSCG00000009545	COL4A2	ssc-mir-4332-p5_1ss18CA	ko05200
ENSSSCG00000013033	BAD	ssc-mir-4332-p5_1ss18CA	ko05200
ENSSSCG00000012981	NR5A1	ssc-mir-4332-p5_1ss18CA	ko05200
ENSSSCG00000007030	IKBKB	ssc-mir-4332-p5_1ss18CA	ko05200
ENSSSCG00000010872	AKT3	ssc-mir-4332-p5_1ss18CA	ko05200
ENSSSCG00000000293	ITGA5	ssc-mir-4332-p5_1ss18CA	ko05205
ENSSSCG00000000293	ITGA5	ssc-mir-4332-p5_1ss18CA	ko05205
ENSSSCG00000000233	ACVR1B	ssc-mir-4332-p5_1ss18CA	ko05205
ENSSSCG00000013033	BAD	ssc-mir-4332-p5_1ss18CA	ko05210

ENSSSCG00000013033	BAD	ssc-mir-4332-p5_1ss18CA	ko05212
ENSSSCG00000012981	NR5A1	ssc-mir-4332-p5_1ss18CA	ko05212
ENSSSCG00000010872	AKT3	ssc-mir-4332-p5_1ss18CA	ko05212
ENSSSCG00000007030	IKBKB	ssc-mir-4332-p5_1ss18CA	ko05212
ENSSSCG00000013033	BAD	ssc-mir-4332-p5_1ss18CA	ko05215
ENSSSCG00000000233	ACVR1B	ssc-mir-4332-p5_1ss18CA	ko05215
ENSSSCG00000000293	ITGA5	ssc-mir-4332-p5_1ss18CA	ko05410
ENSSSCG00000000293	ITGA5	ssc-mir-4332-p5_1ss18CA	ko05410

Supplementary Table S5. Genes regulated by 13 differentially expressed miRNAs

Gene ID	Symbol	miRNA ID
ENSSSCG00000001009	RIPK1	hsa-miR-141-3p_R+1
ENSSSCG00000001009	RIPK1	hsa-miR-141-3p_R+1
ENSSSCG00000001009	RIPK1	hsa-miR-141-3p_R+1
ENSSSCG00000001009	RIPK1	hsa-miR-141-3p_R+1
ENSSSCG00000001009	RIPK1	hsa-miR-141-3p_R+1
ENSSSCG00000003471	EPHA2	hsa-miR-141-3p_R+1
ENSSSCG00000003471	EPHA2	hsa-miR-141-3p_R+1
ENSSSCG00000003471	EPHA2	hsa-miR-141-3p_R+1
ENSSSCG00000003756	LPAR3	hsa-miR-141-3p_R+1
ENSSSCG00000003756	LPAR3	hsa-miR-141-3p_R+1
ENSSSCG00000003756	LPAR3	hsa-miR-141-3p_R+1
ENSSSCG00000003756	LPAR3	hsa-miR-141-3p_R+1
ENSSSCG00000004110	TAB2	hsa-miR-141-3p_R+1
ENSSSCG00000004110	TAB2	hsa-miR-141-3p_R+1
ENSSSCG00000004110	TAB2	hsa-miR-141-3p_R+1
ENSSSCG00000004110	TAB2	hsa-miR-141-3p_R+1
ENSSSCG00000004622	GNB5	hsa-miR-141-3p_R+1
ENSSSCG00000004622	GNB5	hsa-miR-141-3p_R+1
ENSSSCG00000004622	GNB5	hsa-miR-141-3p_R+1
ENSSSCG00000004896	PHLPP1	hsa-miR-141-3p_R+1
ENSSSCG00000006062	YWHAZ	hsa-miR-141-3p_R+1
ENSSSCG00000006062	YWHAZ	hsa-miR-141-3p_R+1
ENSSSCG00000006095	CCNE2	hsa-miR-141-3p_R+1
ENSSSCG00000006095	CCNE2	hsa-miR-141-3p_R+1
ENSSSCG00000006095	CCNE2	hsa-miR-141-3p_R+1
ENSSSCG00000006286	SELE	hsa-miR-141-3p_R+1
ENSSSCG00000006862	VCAM1	hsa-miR-141-3p_R+1
ENSSSCG00000006862	VCAM1	hsa-miR-141-3p_R+1
ENSSSCG00000006862	VCAM1	hsa-miR-141-3p_R+1
ENSSSCG00000006927	PKN2	hsa-miR-141-3p_R+1
ENSSSCG00000007151	NR5A1	hsa-miR-141-3p_R+1

ENSSSCG00000007356	PLCG1	hsa-miR-141-3p_R+1
ENSSSCG00000007356	PLCG1	hsa-miR-141-3p_R+1
ENSSSCG00000007356	PLCG1	hsa-miR-141-3p_R+1
ENSSSCG00000007356	PLCG1	hsa-miR-141-3p_R+1
ENSSSCG00000007356	PLCG1	hsa-miR-141-3p_R+1
ENSSSCG00000007356	PLCG1	hsa-miR-141-3p_R+1
ENSSSCG00000007356	PLCG1	hsa-miR-141-3p_R+1
ENSSSCG00000007356	PLCG1	hsa-miR-141-3p_R+1
ENSSSCG00000007692	YWHAG	hsa-miR-141-3p_R+1
ENSSSCG00000007692	YWHAG	hsa-miR-141-3p_R+1
ENSSSCG00000008090	IL1A	hsa-miR-141-3p_R+1
ENSSSCG00000008090	IL1A	hsa-miR-141-3p_R+1
ENSSSCG00000008090	IL1A	hsa-miR-141-3p_R+1
ENSSSCG00000008241	TCF7L1	hsa-miR-141-3p_R+1
ENSSSCG00000008241	TCF7L1	hsa-miR-141-3p_R+1
ENSSSCG00000008241	TCF7L1	hsa-miR-141-3p_R+1
ENSSSCG00000008241	TCF7L1	hsa-miR-141-3p_R+1
ENSSSCG00000008881	RAPGEF2	hsa-miR-141-3p_R+1
ENSSSCG00000008881	RAPGEF2	hsa-miR-141-3p_R+1
ENSSSCG00000008977	CXCL10	hsa-miR-141-3p_R+1
ENSSSCG00000008977	CXCL10	hsa-miR-141-3p_R+1
ENSSSCG00000008977	CXCL10	hsa-miR-141-3p_R+1
ENSSSCG00000009048	GAB1	hsa-miR-141-3p_R+1
ENSSSCG00000009048	GAB1	hsa-miR-141-3p_R+1
ENSSSCG00000009048	GAB1	hsa-miR-141-3p_R+1
ENSSSCG00000009131	PITX2	hsa-miR-141-3p_R+1
ENSSSCG00000009567	RASA3	hsa-miR-141-3p_R+1
ENSSSCG00000011014	BAMBI	hsa-miR-141-3p_R+1
ENSSSCG00000011274	CTNNB1	hsa-miR-141-3p_R+1
ENSSSCG00000011274	CTNNB1	hsa-miR-141-3p_R+1
ENSSSCG00000011274	CTNNB1	hsa-miR-141-3p_R+1
ENSSSCG00000011274	CTNNB1	hsa-miR-141-3p_R+1
ENSSSCG00000011274	CTNNB1	hsa-miR-141-3p_R+1
ENSSSCG00000011274	CTNNB1	hsa-miR-141-3p_R+1
ENSSSCG00000011274	CTNNB1	hsa-miR-141-3p_R+1
ENSSSCG00000011274	CTNNB1	hsa-miR-141-3p_R+1
ENSSSCG00000015383	RAPGEF5	hsa-miR-141-3p_R+1
ENSSSCG00000015383	RAPGEF5	hsa-miR-141-3p_R+1
ENSSSCG00000015403	HGF	hsa-miR-141-3p_R+1
ENSSSCG00000015403	HGF	hsa-miR-141-3p_R+1
ENSSSCG00000015403	HGF	hsa-miR-141-3p_R+1
ENSSSCG00000015403	HGF	hsa-miR-141-3p_R+1
ENSSSCG00000015403	HGF	hsa-miR-141-3p_R+1
ENSSSCG00000015403	HGF	hsa-miR-141-3p_R+1

ENSSSCG00000015403	HGF	hsa-miR-141-3p_R+1
ENSSSCG00000015407	GNAI1	hsa-miR-141-3p_R+1
ENSSSCG00000015407	GNAI1	hsa-miR-141-3p_R+1
ENSSSCG00000015407	GNAI1	hsa-miR-141-3p_R+1
ENSSSCG00000015407	GNAI1	hsa-miR-141-3p_R+1
ENSSSCG00000015770	VEGFC	hsa-miR-141-3p_R+1
ENSSSCG00000015770	VEGFC	hsa-miR-141-3p_R+1
ENSSSCG00000015770	VEGFC	hsa-miR-141-3p_R+1
ENSSSCG00000015770	VEGFC	hsa-miR-141-3p_R+1
ENSSSCG00000015770	VEGFC	hsa-miR-141-3p_R+1
ENSSSCG00000016991	DUSP1	hsa-miR-141-3p_R+1
ENSSSCG00000017101	ADCY2	hsa-miR-141-3p_R+1
ENSSSCG00000017101	ADCY2	hsa-miR-141-3p_R+1
ENSSSCG00000017101	ADCY2	hsa-miR-141-3p_R+1
ENSSSCG00000017101	ADCY2	hsa-miR-141-3p_R+1
ENSSSCG00000018016	MAP2K4	hsa-miR-141-3p_R+1
ENSSSCG00000018016	MAP2K4	hsa-miR-141-3p_R+1
ENSSSCG00000018016	MAP2K4	hsa-miR-141-3p_R+1
ENSSSCG00000025396	YAP1	hsa-miR-141-3p_R+1
ENSSSCG00000025396	YAP1	hsa-miR-141-3p_R+1
ENSSSCG00000020744	DUSP3	hsa-miR-141-3p_R+1
ENSSSCG00000004622	GNB5	hsa-miR-141-3p_R+1
ENSSSCG00000004622	GNB5	hsa-miR-141-3p_R+1
ENSSSCG00000004622	GNB5	hsa-miR-141-3p_R+1
ENSSSCG00000012034	TIAM1	hsa-miR-141-3p_R+1
ENSSSCG00000012034	TIAM1	hsa-miR-141-3p_R+1
ENSSSCG00000020906	TNFSF10	hsa-miR-141-3p_R+1
ENSSSCG00000027443	MRAS	hsa-miR-141-3p_R+1
ENSSSCG00000027443	MRAS	hsa-miR-141-3p_R+1
ENSSSCG00000027443	MRAS	hsa-miR-141-3p_R+1
ENSSSCG00000027443	MRAS	hsa-miR-141-3p_R+1
ENSSSCG00000027443	MRAS	hsa-miR-141-3p_R+1
ENSSSCG00000029852	WNT5A	hsa-miR-141-3p_R+1
ENSSSCG00000029852	WNT5A	hsa-miR-141-3p_R+1
ENSSSCG00000029852	WNT5A	hsa-miR-141-3p_R+1
ENSSSCG00000029852	WNT5A	hsa-miR-141-3p_R+1
ENSSSCG00000022331	FGF13	hsa-miR-141-3p_R+1
ENSSSCG00000022331	FGF13	hsa-miR-141-3p_R+1
ENSSSCG00000022331	FGF13	hsa-miR-141-3p_R+1
ENSSSCG00000022331	FGF13	hsa-miR-141-3p_R+1
ENSSSCG00000022331	FGF13	hsa-miR-141-3p_R+1
ENSSSCG00000025028	XIAP	hsa-miR-141-3p_R+1
ENSSSCG00000025028	XIAP	hsa-miR-141-3p_R+1
ENSSSCG00000025028	XIAP	hsa-miR-141-3p_R+1

ENSSSCG00000025028	XIAP	hsa-miR-141-3p_R+1
ENSSSCG00000025028	XIAP	hsa-miR-141-3p_R+1
ENSSSCG00000025028	XIAP	hsa-miR-141-3p_R+1
ENSSSCG00000030289	PTPN11	hsa-miR-141-3p_R+1
ENSSSCG00000030289	PTPN11	hsa-miR-141-3p_R+1
ENSSSCG00000030289	PTPN11	hsa-miR-141-3p_R+1
ENSSSCG00000028420	EIF4E	hsa-miR-141-3p_R+1
ENSSSCG00000028420	EIF4E	hsa-miR-141-3p_R+1
ENSSSCG00000028420	EIF4E	hsa-miR-141-3p_R+1
ENSSSCG00000009048	GAB1	hsa-miR-141-3p_R+1
ENSSSCG00000009048	GAB1	hsa-miR-141-3p_R+1
ENSSSCG00000009048	GAB1	hsa-miR-141-3p_R+1
ENSSSCG00000016113	BMPR2	hsa-miR-141-3p_R+1
ENSSSCG00000016113	BMPR2	hsa-miR-141-3p_R+1
ENSSSCG00000016887	ITGA2	hsa-miR-141-3p_R+1
ENSSSCG00000016887	ITGA2	hsa-miR-141-3p_R+1
ENSSSCG00000016887	ITGA2	hsa-miR-141-3p_R+1
ENSSSCG00000016887	ITGA2	hsa-miR-141-3p_R+1
ENSSSCG00000016887	ITGA2	hsa-miR-141-3p_R+1
ENSSSCG00000009048	GAB1	hsa-miR-141-3p_R+1
ENSSSCG00000009048	GAB1	hsa-miR-141-3p_R+1
ENSSSCG00000009048	GAB1	hsa-miR-141-3p_R+1
ENSSSCG00000006286	SELE	hsa-miR-141-3p_R+1
ENSSSCG00000008977	CXCL10	hsa-miR-141-3p_R+1
ENSSSCG00000008977	CXCL10	hsa-miR-141-3p_R+1
ENSSSCG00000008977	CXCL10	hsa-miR-141-3p_R+1
ENSSSCG00000010414	CXCL12	hsa-miR-141-3p_R+1
ENSSSCG00000010414	CXCL12	hsa-miR-141-3p_R+1
ENSSSCG00000016027	ITGAV	hsa-miR-141-3p_R+1
ENSSSCG00000016027	ITGAV	hsa-miR-141-3p_R+1
ENSSSCG00000016027	ITGAV	hsa-miR-141-3p_R+1
ENSSSCG00000016027	ITGAV	hsa-miR-141-3p_R+1
ENSSSCG00000016027	ITGAV	hsa-miR-141-3p_R+1
ENSSSCG00000009048	GAB1	hsa-miR-141-3p_R+1
ENSSSCG00000009048	GAB1	hsa-miR-141-3p_R+1
ENSSSCG00000009048	GAB1	hsa-miR-141-3p_R+1
ENSSSCG00000005382	TGFBR1	hsa-miR-141-3p_R+1
ENSSSCG00000005382	TGFBR1	hsa-miR-141-3p_R+1
ENSSSCG00000005382	TGFBR1	hsa-miR-141-3p_R+1
ENSSSCG00000005382	TGFBR1	hsa-miR-141-3p_R+1
ENSSSCG00000005382	TGFBR1	hsa-miR-141-3p_R+1
ENSSSCG00000005382	TGFBR1	hsa-miR-141-3p_R+1
ENSSSCG00000005382	TGFBR1	hsa-miR-141-3p_R+1
ENSSSCG00000005382	TGFBR1	hsa-miR-141-3p_R+1

ENSSSCG00000001518	ITPR3	hsa-miR-222-5p_L+2R-1
ENSSSCG00000001901	CYP1A2	hsa-miR-222-5p_L+2R-1
ENSSSCG00000002804	CSNK2A2	hsa-miR-222-5p_L+2R-1
ENSSSCG00000003017	TGFB1	hsa-miR-222-5p_L+2R-1
ENSSSCG00000003017	TGFB1	hsa-miR-222-5p_L+2R-1
ENSSSCG00000003017	TGFB1	hsa-miR-222-5p_L+2R-1
ENSSSCG00000003017	TGFB1	hsa-miR-222-5p_L+2R-1
ENSSSCG00000003017	TGFB1	hsa-miR-222-5p_L+2R-1
ENSSSCG00000003017	TGFB1	hsa-miR-222-5p_L+2R-1
ENSSSCG00000003017	TGFB1	hsa-miR-222-5p_L+2R-1
ENSSSCG00000003017	TGFB1	hsa-miR-222-5p_L+2R-1
ENSSSCG00000003017	TGFB1	hsa-miR-222-5p_L+2R-1
ENSSSCG00000003017	TGFB1	hsa-miR-222-5p_L+2R-1
ENSSSCG00000004192	CTGF	hsa-miR-222-5p_L+2R-1
ENSSSCG00000004952	SMAD3	hsa-miR-222-5p_L+2R-1
ENSSSCG00000004952	SMAD3	hsa-miR-222-5p_L+2R-1
ENSSSCG00000004952	SMAD3	hsa-miR-222-5p_L+2R-1
ENSSSCG00000004952	SMAD3	hsa-miR-222-5p_L+2R-1
ENSSSCG00000004952	SMAD3	hsa-miR-222-5p_L+2R-1
ENSSSCG00000004952	SMAD3	hsa-miR-222-5p_L+2R-1
ENSSSCG00000004952	SMAD3	hsa-miR-222-5p_L+2R-1
ENSSSCG00000005738	RALGDS	hsa-miR-222-5p_L+2R-1
ENSSSCG00000005738	RALGDS	hsa-miR-222-5p_L+2R-1
ENSSSCG00000005738	RALGDS	hsa-miR-222-5p_L+2R-1
ENSSSCG00000005738	RALGDS	hsa-miR-222-5p_L+2R-1
ENSSSCG00000005738	RALGDS	hsa-miR-222-5p_L+2R-1
ENSSSCG00000005738	RALGDS	hsa-miR-222-5p_L+2R-1
ENSSSCG00000006145	E2F5	hsa-miR-222-5p_L+2R-1
ENSSSCG00000006927	PKN2	hsa-miR-222-5p_L+2R-1
ENSSSCG00000007356	PLCG1	hsa-miR-222-5p_L+2R-1
ENSSSCG00000007356	PLCG1	hsa-miR-222-5p_L+2R-1
ENSSSCG00000007356	PLCG1	hsa-miR-222-5p_L+2R-1
ENSSSCG00000007356	PLCG1	hsa-miR-222-5p_L+2R-1
ENSSSCG00000007356	PLCG1	hsa-miR-222-5p_L+2R-1
ENSSSCG00000007356	PLCG1	hsa-miR-222-5p_L+2R-1
ENSSSCG00000007356	PLCG1	hsa-miR-222-5p_L+2R-1
ENSSSCG00000007356	PLCG1	hsa-miR-222-5p_L+2R-1
ENSSSCG00000009228	MAPK10	hsa-miR-222-5p_L+2R-1
ENSSSCG00000009228	MAPK10	hsa-miR-222-5p_L+2R-1
ENSSSCG00000009228	MAPK10	hsa-miR-222-5p_L+2R-1
ENSSSCG00000009228	MAPK10	hsa-miR-222-5p_L+2R-1
ENSSSCG00000009228	MAPK10	hsa-miR-222-5p_L+2R-1
ENSSSCG00000009228	MAPK10	hsa-miR-222-5p_L+2R-1
ENSSSCG00000009228	MAPK10	hsa-miR-222-5p_L+2R-1

ENSSSCG00000009228	MAPK10	hsa-miR-222-5p_L+2R-1
ENSSSCG00000009228	MAPK10	hsa-miR-222-5p_L+2R-1
ENSSSCG00000009228	MAPK10	hsa-miR-222-5p_L+2R-1
ENSSSCG00000009855	KSR2	hsa-miR-222-5p_L+2R-1
ENSSSCG00000011014	BAMBI	hsa-miR-222-5p_L+2R-1
ENSSSCG00000013655	ICAM1	hsa-miR-222-5p_L+2R-1
ENSSSCG00000013655	ICAM1	hsa-miR-222-5p_L+2R-1
ENSSSCG00000013655	ICAM1	hsa-miR-222-5p_L+2R-1
ENSSSCG00000013888	JAK3	hsa-miR-222-5p_L+2R-1
ENSSSCG00000013888	JAK3	hsa-miR-222-5p_L+2R-1
ENSSSCG00000014891	NR5A1	hsa-miR-222-5p_L+2R-1
ENSSSCG00000014891	NR5A1	hsa-miR-222-5p_L+2R-1
ENSSSCG00000014891	NR5A1	hsa-miR-222-5p_L+2R-1
ENSSSCG00000015815	NR5A1	hsa-miR-222-5p_L+2R-1
ENSSSCG00000015815	NR5A1	hsa-miR-222-5p_L+2R-1
ENSSSCG00000015815	NR5A1	hsa-miR-222-5p_L+2R-1
ENSSSCG00000015815	NR5A1	hsa-miR-222-5p_L+2R-1
ENSSSCG00000015815	NR5A1	hsa-miR-222-5p_L+2R-1
ENSSSCG00000015815	NR5A1	hsa-miR-222-5p_L+2R-1
ENSSSCG00000015815	NR5A1	hsa-miR-222-5p_L+2R-1
ENSSSCG00000016101	CFLAR	hsa-miR-222-5p_L+2R-1
ENSSSCG00000016101	CFLAR	hsa-miR-222-5p_L+2R-1
ENSSSCG00000016101	CFLAR	hsa-miR-222-5p_L+2R-1
ENSSSCG00000016878	FGF10	hsa-miR-222-5p_L+2R-1
ENSSSCG00000016878	FGF10	hsa-miR-222-5p_L+2R-1
ENSSSCG00000016878	FGF10	hsa-miR-222-5p_L+2R-1
ENSSSCG00000016878	FGF10	hsa-miR-222-5p_L+2R-1
ENSSSCG00000016878	FGF10	hsa-miR-222-5p_L+2R-1
ENSSSCG00000017748	NF1	hsa-miR-222-5p_L+2R-1
ENSSSCG00000017748	NF1	hsa-miR-222-5p_L+2R-1
ENSSSCG00000021068	TRAF5	hsa-miR-222-5p_L+2R-1
ENSSSCG00000021068	TRAF5	hsa-miR-222-5p_L+2R-1
ENSSSCG00000021068	TRAF5	hsa-miR-222-5p_L+2R-1
ENSSSCG00000017748	NF1	hsa-miR-222-5p_L+2R-1
ENSSSCG00000017748	NF1	hsa-miR-222-5p_L+2R-1
ENSSSCG00000012034	TIAM1	hsa-miR-222-5p_L+2R-1
ENSSSCG00000012034	TIAM1	hsa-miR-222-5p_L+2R-1
ENSSSCG00000012034	TIAM1	hsa-miR-222-5p_L+2R-1
ENSSSCG00000026969	KRAS	hsa-miR-222-5p_L+2R-1
ENSSSCG00000026969	KRAS	hsa-miR-222-5p_L+2R-1
ENSSSCG00000026969	KRAS	hsa-miR-222-5p_L+2R-1
ENSSSCG00000026969	KRAS	hsa-miR-222-5p_L+2R-1
ENSSSCG00000026969	KRAS	hsa-miR-222-5p_L+2R-1
ENSSSCG00000026969	KRAS	hsa-miR-222-5p_L+2R-1

ENSSSCG00000026969	KRAS	hsa-miR-222-5p_L+2R-1
ENSSSCG00000026969	KRAS	hsa-miR-222-5p_L+2R-1
ENSSSCG00000026969	KRAS	hsa-miR-222-5p_L+2R-1
ENSSSCG00000026969	KRAS	hsa-miR-222-5p_L+2R-1
ENSSSCG00000027443	MRAS	hsa-miR-222-5p_L+2R-1
ENSSSCG00000027443	MRAS	hsa-miR-222-5p_L+2R-1
ENSSSCG00000027443	MRAS	hsa-miR-222-5p_L+2R-1
ENSSSCG00000027443	MRAS	hsa-miR-222-5p_L+2R-1
ENSSSCG00000027443	MRAS	hsa-miR-222-5p_L+2R-1
ENSSSCG00000022482	DDIT3	hsa-miR-222-5p_L+2R-1
ENSSSCG00000024312	ID4	hsa-miR-222-5p_L+2R-1
ENSSSCG00000030153	SMURF1	hsa-miR-222-5p_L+2R-1
ENSSSCG00000030153	SMURF1	hsa-miR-222-5p_L+2R-1
ENSSSCG00000030153	SMURF1	hsa-miR-222-5p_L+2R-1
ENSSSCG00000025092	CDK4	hsa-miR-222-5p_L+2R-1
ENSSSCG00000025092	CDK4	hsa-miR-222-5p_L+2R-1
ENSSSCG00000025092	CDK4	hsa-miR-222-5p_L+2R-1
ENSSSCG00000025092	CDK4	hsa-miR-222-5p_L+2R-1
ENSSSCG00000025092	CDK4	hsa-miR-222-5p_L+2R-1
ENSSSCG00000025092	CDK4	hsa-miR-222-5p_L+2R-1
ENSSSCG00000025092	CDK4	hsa-miR-222-5p_L+2R-1
ENSSSCG00000027426	NR5A1	hsa-miR-222-5p_L+2R-1
ENSSSCG00000024954	FGF1	hsa-miR-222-5p_L+2R-1
ENSSSCG00000024954	FGF1	hsa-miR-222-5p_L+2R-1
ENSSSCG00000024954	FGF1	hsa-miR-222-5p_L+2R-1
ENSSSCG00000024954	FGF1	hsa-miR-222-5p_L+2R-1
ENSSSCG00000024954	FGF1	hsa-miR-222-5p_L+2R-1
ENSSSCG00000024954	FGF1	hsa-miR-222-5p_L+2R-1
ENSSSCG00000022849	IL2RA	hsa-miR-222-5p_L+2R-1
ENSSSCG00000022849	IL2RA	hsa-miR-222-5p_L+2R-1
ENSSSCG00000013655	ICAM1	hsa-miR-222-5p_L+2R-1
ENSSSCG00000013655	ICAM1	hsa-miR-222-5p_L+2R-1
ENSSSCG00000013655	ICAM1	hsa-miR-222-5p_L+2R-1
ENSSSCG00000016887	ITGA2	hsa-miR-222-5p_L+2R-1
ENSSSCG00000016887	ITGA2	hsa-miR-222-5p_L+2R-1
ENSSSCG00000016887	ITGA2	hsa-miR-222-5p_L+2R-1
ENSSSCG00000016887	ITGA2	hsa-miR-222-5p_L+2R-1
ENSSSCG00000016887	ITGA2	hsa-miR-222-5p_L+2R-1
ENSSSCG00000015815	NR5A1	hsa-miR-222-5p_L+2R-1
ENSSSCG00000015815	NR5A1	hsa-miR-222-5p_L+2R-1
ENSSSCG00000015815	NR5A1	hsa-miR-222-5p_L+2R-1
ENSSSCG00000015815	NR5A1	hsa-miR-222-5p_L+2R-1
ENSSSCG00000015815	NR5A1	hsa-miR-222-5p_L+2R-1
ENSSSCG00000015815	NR5A1	hsa-miR-222-5p_L+2R-1

ENSSSCG00000015815	NR5A1	hsa-miR-222-5p_L+2R-1
ENSSSCG00000012591	AMOT	hsa-miR-222-5p_L+2R-1
ENSSSCG00000012591	AMOT	hsa-miR-222-5p_L+2R-1
ENSSSCG00000013888	JAK3	hsa-miR-222-5p_L+2R-1
ENSSSCG00000013888	JAK3	hsa-miR-222-5p_L+2R-1
ENSSSCG00000015815	NR5A1	hsa-miR-222-5p_L+2R-1
ENSSSCG00000015815	NR5A1	hsa-miR-222-5p_L+2R-1
ENSSSCG00000015815	NR5A1	hsa-miR-222-5p_L+2R-1
ENSSSCG00000015815	NR5A1	hsa-miR-222-5p_L+2R-1
ENSSSCG00000015815	NR5A1	hsa-miR-222-5p_L+2R-1
ENSSSCG00000015815	NR5A1	hsa-miR-222-5p_L+2R-1
ENSSSCG00000015815	NR5A1	hsa-miR-222-5p_L+2R-1
ENSSSCG00000015815	NR5A1	hsa-miR-222-5p_L+2R-1
ENSSSCG00000015815	NR5A1	hsa-miR-222-5p_L+2R-1
ENSSSCG00000015815	NR5A1	hsa-miR-222-5p_L+2R-1
ENSSSCG00000015815	NR5A1	hsa-miR-222-5p_L+2R-1
ENSSSCG00000015815	NR5A1	hsa-miR-222-5p_L+2R-1
ENSSSCG00000015815	NR5A1	hsa-miR-222-5p_L+2R-1
ENSSSCG00000015815	NR5A1	hsa-miR-222-5p_L+2R-1
ENSSSCG00000017705	CCL5	hsa-miR-222-5p_L+2R-1
ENSSSCG00000017705	CCL5	hsa-miR-222-5p_L+2R-1
ENSSSCG00000024813	MAP3K3	hsa-miR-222-5p_L+2R-1
ENSSSCG00000024813	MAP3K3	hsa-miR-222-5p_L+2R-1
ENSSSCG00000016101	CFLAR	hsa-miR-222-5p_L+2R-1
ENSSSCG00000016101	CFLAR	hsa-miR-222-5p_L+2R-1
ENSSSCG00000016101	CFLAR	hsa-miR-222-5p_L+2R-1
ENSSSCG00000003017	TGFB1	hsa-miR-222-5p_L+2R-1
ENSSSCG00000003017	TGFB1	hsa-miR-222-5p_L+2R-1
ENSSSCG00000003017	TGFB1	hsa-miR-222-5p_L+2R-1
ENSSSCG00000003017	TGFB1	hsa-miR-222-5p_L+2R-1
ENSSSCG00000003017	TGFB1	hsa-miR-222-5p_L+2R-1
ENSSSCG00000003017	TGFB1	hsa-miR-222-5p_L+2R-1
ENSSSCG00000003017	TGFB1	hsa-miR-222-5p_L+2R-1
ENSSSCG00000003017	TGFB1	hsa-miR-222-5p_L+2R-1
ENSSSCG00000003017	TGFB1	hsa-miR-222-5p_L+2R-1
ENSSSCG00000003017	TGFB1	hsa-miR-222-5p_L+2R-1
ENSSSCG00000015815	NR5A1	hsa-miR-222-5p_L+2R-1
ENSSSCG00000015815	NR5A1	hsa-miR-222-5p_L+2R-1
ENSSSCG00000015815	NR5A1	hsa-miR-222-5p_L+2R-1
ENSSSCG00000015815	NR5A1	hsa-miR-222-5p_L+2R-1
ENSSSCG00000015815	NR5A1	hsa-miR-222-5p_L+2R-1
ENSSSCG00000015815	NR5A1	hsa-miR-222-5p_L+2R-1
ENSSSCG00000001518	ITPR3	hsa-miR-4792_1ss9GT
ENSSSCG00000001898	ULK3	hsa-miR-4792_1ss9GT

ENSSSCG00000004022	RPS6KA2	hsa-miR-4792_1ss9GT
ENSSSCG00000004022	RPS6KA2	hsa-miR-4792_1ss9GT
ENSSSCG00000004507	SMAD7	hsa-miR-4792_1ss9GT
ENSSSCG00000004507	SMAD7	hsa-miR-4792_1ss9GT
ENSSSCG00000005838	TRAF2	hsa-miR-4792_1ss9GT
ENSSSCG00000005838	TRAF2	hsa-miR-4792_1ss9GT
ENSSSCG00000005838	TRAF2	hsa-miR-4792_1ss9GT
ENSSSCG00000005838	TRAF2	hsa-miR-4792_1ss9GT
ENSSSCG00000005838	TRAF2	hsa-miR-4792_1ss9GT
ENSSSCG00000005838	TRAF2	hsa-miR-4792_1ss9GT
ENSSSCG00000005838	TRAF2	hsa-miR-4792_1ss9GT
ENSSSCG00000005838	TRAF2	hsa-miR-4792_1ss9GT
ENSSSCG00000005838	TRAF2	hsa-miR-4792_1ss9GT
ENSSSCG00000006927	PKN2	hsa-miR-4792_1ss9GT
ENSSSCG00000007673	NR5A1	hsa-miR-4792_1ss9GT
ENSSSCG00000008162	IL1R1	hsa-miR-4792_1ss9GT
ENSSSCG00000008162	IL1R1	hsa-miR-4792_1ss9GT
ENSSSCG00000008162	IL1R1	hsa-miR-4792_1ss9GT
ENSSSCG00000008162	IL1R1	hsa-miR-4792_1ss9GT
ENSSSCG00000008162	IL1R1	hsa-miR-4792_1ss9GT
ENSSSCG00000008842	KIT	hsa-miR-4792_1ss9GT
ENSSSCG00000008842	KIT	hsa-miR-4792_1ss9GT
ENSSSCG00000008842	KIT	hsa-miR-4792_1ss9GT
ENSSSCG00000008842	KIT	hsa-miR-4792_1ss9GT
ENSSSCG00000008842	KIT	hsa-miR-4792_1ss9GT
ENSSSCG00000009855	KSR2	hsa-miR-4792_1ss9GT
ENSSSCG00000010132	COMT	hsa-miR-4792_1ss9GT
ENSSSCG00000011412	CACNA2D2	hsa-miR-4792_1ss9GT
ENSSSCG00000011412	CACNA2D2	hsa-miR-4792_1ss9GT
ENSSSCG00000011415	MAPKAPK3	hsa-miR-4792_1ss9GT
ENSSSCG00000012399	FOXO4	hsa-miR-4792_1ss9GT
ENSSSCG00000015850	DUSP4	hsa-miR-4792_1ss9GT
ENSSSCG00000016140	FZD5	hsa-miR-4792_1ss9GT
ENSSSCG00000016140	FZD5	hsa-miR-4792_1ss9GT
ENSSSCG00000016140	FZD5	hsa-miR-4792_1ss9GT
ENSSSCG00000016140	FZD5	hsa-miR-4792_1ss9GT
ENSSSCG00000017357	ITGA2B	hsa-miR-4792_1ss9GT
ENSSSCG00000017357	ITGA2B	hsa-miR-4792_1ss9GT
ENSSSCG00000017357	ITGA2B	hsa-miR-4792_1ss9GT
ENSSSCG00000017357	ITGA2B	hsa-miR-4792_1ss9GT
ENSSSCG00000017357	ITGA2B	hsa-miR-4792_1ss9GT
ENSSSCG00000025374	DVL3	hsa-miR-4792_1ss9GT
ENSSSCG00000025374	DVL3	hsa-miR-4792_1ss9GT
ENSSSCG00000025374	DVL3	hsa-miR-4792_1ss9GT

ENSSSCG00000025374	DVL3	hsa-miR-4792_1ss9GT
ENSSSCG00000022066	PIAS4	hsa-miR-4792_1ss9GT
ENSSSCG00000022066	PIAS4	hsa-miR-4792_1ss9GT
ENSSSCG00000022066	PIAS4	hsa-miR-4792_1ss9GT
ENSSSCG00000027668	NLK	hsa-miR-4792_1ss9GT
ENSSSCG00000022689	GADD45B	hsa-miR-4792_1ss9GT
ENSSSCG00000017306	ITGB3	hsa-miR-4792_1ss9GT
ENSSSCG00000017306	ITGB3	hsa-miR-4792_1ss9GT
ENSSSCG00000017306	ITGB3	hsa-miR-4792_1ss9GT
ENSSSCG00000017306	ITGB3	hsa-miR-4792_1ss9GT
ENSSSCG00000017306	ITGB3	hsa-miR-4792_1ss9GT
ENSSSCG00000017306	ITGB3	hsa-miR-4792_1ss9GT
ENSSSCG00000011101	ITGB1	hsa-miR-4792_1ss9GT
ENSSSCG00000011101	ITGB1	hsa-miR-4792_1ss9GT
ENSSSCG00000011101	ITGB1	hsa-miR-4792_1ss9GT
ENSSSCG00000011101	ITGB1	hsa-miR-4792_1ss9GT
ENSSSCG00000011101	ITGB1	hsa-miR-4792_1ss9GT
ENSSSCG00000011101	ITGB1	hsa-miR-4792_1ss9GT
ENSSSCG00000012495	BTK	hsa-miR-4792_1ss9GT
ENSSSCG00000012495	BTK	hsa-miR-4792_1ss9GT
ENSSSCG00000012495	BTK	hsa-miR-4792_1ss9GT
ENSSSCG00000002960	RASGRP4	hsa-miR-4792_1ss9GT
ENSSSCG00000002960	RASGRP4	hsa-miR-4792_1ss9GT
ENSSSCG00000002960	RASGRP4	hsa-miR-4792_1ss9GT
ENSSSCG00000012495	BTK	hsa-miR-4792_1ss9GT
ENSSSCG00000012495	BTK	hsa-miR-4792_1ss9GT
ENSSSCG00000012495	BTK	hsa-miR-4792_1ss9GT
ENSSSCG00000012495	BTK	hsa-miR-4792_1ss9GT
ENSSSCG00000012495	BTK	hsa-miR-4792_1ss9GT
ENSSSCG00000012495	BTK	hsa-miR-4792_1ss9GT
ENSSSCG00000012495	BTK	hsa-miR-4792_1ss9GT
ENSSSCG00000012495	BTK	hsa-miR-4792_1ss9GT
ENSSSCG00000012495	BTK	hsa-miR-4792_1ss9GT
ENSSSCG00000013564	INSR	hsa-miR-4792_1ss9GT
ENSSSCG00000013564	INSR	hsa-miR-4792_1ss9GT
ENSSSCG00000013564	INSR	hsa-miR-4792_1ss9GT
ENSSSCG00000013564	INSR	hsa-miR-4792_1ss9GT
ENSSSCG00000013564	INSR	hsa-miR-4792_1ss9GT
ENSSSCG00000007520	GNAS	hsa-miR-4792_1ss9GT
ENSSSCG00000007520	GNAS	hsa-miR-4792_1ss9GT
ENSSSCG00000007520	GNAS	hsa-miR-4792_1ss9GT
ENSSSCG00000017306	ITGB3	hsa-miR-4792_1ss9GT
ENSSSCG00000017306	ITGB3	hsa-miR-4792_1ss9GT
ENSSSCG00000017306	ITGB3	hsa-miR-4792_1ss9GT

ENSSSCG00000017306	ITGB3	hsa-miR-4792_1ss9GT
ENSSSCG00000017306	ITGB3	hsa-miR-4792_1ss9GT
ENSSSCG00000017306	ITGB3	hsa-miR-4792_1ss9GT
ENSSSCG00000000293	ITGA5	PC-5p-9551_196
ENSSSCG00000000293	ITGA5	PC-5p-9551_196
ENSSSCG00000000293	ITGA5	PC-5p-9551_196
ENSSSCG00000000293	ITGA5	PC-5p-9551_196
ENSSSCG00000000293	ITGA5	PC-5p-9551_196
ENSSSCG00000000293	ITGA5	PC-5p-9551_196
ENSSSCG00000000293	ITGA5	PC-5p-9551_196
ENSSSCG00000000293	ITGA5	PC-5p-9551_196
ENSSSCG00000001404	NR5A1	PC-5p-9551_196
ENSSSCG00000001404	NR5A1	PC-5p-9551_196
ENSSSCG00000001404	NR5A1	PC-5p-9551_196
ENSSSCG00000001404	NR5A1	PC-5p-9551_196
ENSSSCG00000001404	NR5A1	PC-5p-9551_196
ENSSSCG00000001404	NR5A1	PC-5p-9551_196
ENSSSCG00000001404	NR5A1	PC-5p-9551_196
ENSSSCG00000001404	NR5A1	PC-5p-9551_196
ENSSSCG00000001404	NR5A1	PC-5p-9551_196
ENSSSCG00000001404	NR5A1	PC-5p-9551_196
ENSSSCG00000001404	NR5A1	PC-5p-9551_196
ENSSSCG00000001661	SRF	PC-5p-9551_196
ENSSSCG00000001661	SRF	PC-5p-9551_196
ENSSSCG00000002804	CSNK2A2	PC-5p-9551_196
ENSSSCG00000003154	GYS1	PC-5p-9551_196
ENSSSCG00000004154	TNFAIP3	PC-5p-9551_196
ENSSSCG00000004154	TNFAIP3	PC-5p-9551_196
ENSSSCG00000004789	THBS1	PC-5p-9551_196
ENSSSCG00000004789	THBS1	PC-5p-9551_196
ENSSSCG00000004789	THBS1	PC-5p-9551_196
ENSSSCG00000004789	THBS1	PC-5p-9551_196
ENSSSCG00000004789	THBS1	PC-5p-9551_196
ENSSSCG00000005661	NR5A1	PC-5p-9551_196
ENSSSCG00000006539	SHC1	PC-5p-9551_196
ENSSSCG00000006539	SHC1	PC-5p-9551_196
ENSSSCG00000006539	SHC1	PC-5p-9551_196
ENSSSCG00000006780	WNT2B	PC-5p-9551_196
ENSSSCG00000006780	WNT2B	PC-5p-9551_196
ENSSSCG00000006780	WNT2B	PC-5p-9551_196
ENSSSCG00000006780	WNT2B	PC-5p-9551_196
ENSSSCG00000008040	TSC2	PC-5p-9551_196
ENSSSCG00000008040	TSC2	PC-5p-9551_196

ENSSSCG00000008164	MAP4K4	PC-5p-9551_196
ENSSSCG00000009040	SMAD1	PC-5p-9551_196
ENSSSCG00000009040	SMAD1	PC-5p-9551_196
ENSSSCG00000009228	MAPK10	PC-5p-9551_196
ENSSSCG00000009228	MAPK10	PC-5p-9551_196
ENSSSCG00000009228	MAPK10	PC-5p-9551_196
ENSSSCG00000009228	MAPK10	PC-5p-9551_196
ENSSSCG00000009228	MAPK10	PC-5p-9551_196
ENSSSCG00000009228	MAPK10	PC-5p-9551_196
ENSSSCG00000009228	MAPK10	PC-5p-9551_196
ENSSSCG00000009228	MAPK10	PC-5p-9551_196
ENSSSCG00000009228	MAPK10	PC-5p-9551_196
ENSSSCG00000009228	MAPK10	PC-5p-9551_196
ENSSSCG00000009370	FOXO1	PC-5p-9551_196
ENSSSCG00000009370	FOXO1	PC-5p-9551_196
ENSSSCG00000009567	RASA3	PC-5p-9551_196
ENSSSCG00000009840	PRKAB1	PC-5p-9551_196
ENSSSCG00000009874	NR5A1	PC-5p-9551_196
ENSSSCG00000011101	ITGB1	PC-5p-9551_196
ENSSSCG00000011101	ITGB1	PC-5p-9551_196
ENSSSCG00000011101	ITGB1	PC-5p-9551_196
ENSSSCG00000011101	ITGB1	PC-5p-9551_196
ENSSSCG00000011101	ITGB1	PC-5p-9551_196
ENSSSCG00000011101	ITGB1	PC-5p-9551_196
ENSSSCG00000011415	MAPKAPK3	PC-5p-9551_196
ENSSSCG00000012657	AIFM1	PC-5p-9551_196
ENSSSCG00000012913	PPP1CA	PC-5p-9551_196
ENSSSCG00000012913	PPP1CA	PC-5p-9551_196
ENSSSCG00000012913	PPP1CA	PC-5p-9551_196
ENSSSCG00000013020	MAP4K2	PC-5p-9551_196
ENSSSCG00000014146	RASA1	PC-5p-9551_196
ENSSSCG00000014146	RASA1	PC-5p-9551_196
ENSSSCG00000014216	TICAM2	PC-5p-9551_196
ENSSSCG00000014220	ATG12	PC-5p-9551_196
ENSSSCG00000015383	RAPGEF5	PC-5p-9551_196
ENSSSCG00000015383	RAPGEF5	PC-5p-9551_196
ENSSSCG00000016578	FLNC	PC-5p-9551_196
ENSSSCG00000016578	FLNC	PC-5p-9551_196
ENSSSCG00000016578	FLNC	PC-5p-9551_196
ENSSSCG00000016628	WNT2	PC-5p-9551_196
ENSSSCG00000016628	WNT2	PC-5p-9551_196
ENSSSCG00000016628	WNT2	PC-5p-9551_196
ENSSSCG00000016628	WNT2	PC-5p-9551_196
ENSSSCG00000017330	MAP3K14	PC-5p-9551_196

ENSSSCG00000017330	MAP3K14	PC-5p-9551_196
ENSSSCG00000017330	MAP3K14	PC-5p-9551_196
ENSSSCG00000017330	MAP3K14	PC-5p-9551_196
ENSSSCG00000017330	MAP3K14	PC-5p-9551_196
ENSSSCG00000017330	MAP3K14	PC-5p-9551_196
ENSSSCG00000017330	MAP3K14	PC-5p-9551_196
ENSSSCG00000017562	CACNA1G	PC-5p-9551_196
ENSSSCG00000017562	CACNA1G	PC-5p-9551_196
ENSSSCG00000017578	ITGA3	PC-5p-9551_196
ENSSSCG00000017578	ITGA3	PC-5p-9551_196
ENSSSCG00000017578	ITGA3	PC-5p-9551_196
ENSSSCG00000017578	ITGA3	PC-5p-9551_196
ENSSSCG00000020744	DUSP3	PC-5p-9551_196
ENSSSCG00000025374	DVL3	PC-5p-9551_196
ENSSSCG00000025374	DVL3	PC-5p-9551_196
ENSSSCG00000025374	DVL3	PC-5p-9551_196
ENSSSCG00000025374	DVL3	PC-5p-9551_196
ENSSSCG00000029852	WNT5A	PC-5p-9551_196
ENSSSCG00000029852	WNT5A	PC-5p-9551_196
ENSSSCG00000029852	WNT5A	PC-5p-9551_196
ENSSSCG00000029852	WNT5A	PC-5p-9551_196
ENSSSCG00000030153	SMURF1	PC-5p-9551_196
ENSSSCG00000030153	SMURF1	PC-5p-9551_196
ENSSSCG00000030153	SMURF1	PC-5p-9551_196
ENSSSCG00000024813	MAP3K3	PC-5p-9551_196
ENSSSCG00000024813	MAP3K3	PC-5p-9551_196
ENSSSCG00000027426	NR5A1	PC-5p-9551_196
ENSSSCG00000027952	ADCY5	PC-5p-9551_196
ENSSSCG00000027952	ADCY5	PC-5p-9551_196
ENSSSCG00000027952	ADCY5	PC-5p-9551_196
ENSSSCG00000027952	ADCY5	PC-5p-9551_196
ENSSSCG00000025768	CALM1	PC-5p-9551_196
ENSSSCG00000025768	CALM1	PC-5p-9551_196
ENSSSCG00000025768	CALM1	PC-5p-9551_196
ENSSSCG00000022545	CALM3	PC-5p-9551_196
ENSSSCG00000022545	CALM3	PC-5p-9551_196
ENSSSCG00000022545	CALM3	PC-5p-9551_196
ENSSSCG00000005382	TGFBR1	PC-5p-9551_196
ENSSSCG00000005382	TGFBR1	PC-5p-9551_196
ENSSSCG00000005382	TGFBR1	PC-5p-9551_196
ENSSSCG00000005382	TGFBR1	PC-5p-9551_196
ENSSSCG00000005382	TGFBR1	PC-5p-9551_196
ENSSSCG00000005382	TGFBR1	PC-5p-9551_196
ENSSSCG00000005382	TGFBR1	PC-5p-9551_196

ENSSSCG00000005382	TGFBR1	PC-5p-9551_196
ENSSSCG00000011101	ITGB1	PC-5p-9551_196
ENSSSCG00000011101	ITGB1	PC-5p-9551_196
ENSSSCG00000011101	ITGB1	PC-5p-9551_196
ENSSSCG00000011101	ITGB1	PC-5p-9551_196
ENSSSCG00000011101	ITGB1	PC-5p-9551_196
ENSSSCG00000011101	ITGB1	PC-5p-9551_196
ENSSSCG00000015815	NR5A1	PC-5p-9551_196
ENSSSCG00000015815	NR5A1	PC-5p-9551_196
ENSSSCG00000015815	NR5A1	PC-5p-9551_196
ENSSSCG00000015815	NR5A1	PC-5p-9551_196
ENSSSCG00000015815	NR5A1	PC-5p-9551_196
ENSSSCG00000015815	NR5A1	PC-5p-9551_196
ENSSSCG00000015815	NR5A1	PC-5p-9551_196
ENSSSCG00000017578	ITGA3	PC-5p-9551_196
ENSSSCG00000017578	ITGA3	PC-5p-9551_196
ENSSSCG00000017578	ITGA3	PC-5p-9551_196
ENSSSCG00000017578	ITGA3	PC-5p-9551_196
ENSSSCG00000004154	TNFAIP3	PC-5p-9551_196
ENSSSCG00000004154	TNFAIP3	PC-5p-9551_196
ENSSSCG00000006539	SHC1	PC-5p-9551_196
ENSSSCG00000006539	SHC1	PC-5p-9551_196
ENSSSCG00000006539	SHC1	PC-5p-9551_196
ENSSSCG00000012275	ARAF	PC-5p-9551_196
ENSSSCG00000012275	ARAF	PC-5p-9551_196
ENSSSCG00000012275	ARAF	PC-5p-9551_196
ENSSSCG00000012275	ARAF	PC-5p-9551_196
ENSSSCG00000012275	ARAF	PC-5p-9551_196
ENSSSCG00000010872	AKT3	PC-5p-9551_196
ENSSSCG00000010872	AKT3	PC-5p-9551_196
ENSSSCG00000010872	AKT3	PC-5p-9551_196
ENSSSCG00000010872	AKT3	PC-5p-9551_196
ENSSSCG00000010872	AKT3	PC-5p-9551_196
ENSSSCG00000010872	AKT3	PC-5p-9551_196
ENSSSCG00000010872	AKT3	PC-5p-9551_196
ENSSSCG00000010872	AKT3	PC-5p-9551_196
ENSSSCG00000010872	AKT3	PC-5p-9551_196
ENSSSCG00000010872	AKT3	PC-5p-9551_196
ENSSSCG00000010872	AKT3	PC-5p-9551_196
ENSSSCG00000009370	FOXO1	PC-5p-9551_196
ENSSSCG00000009370	FOXO1	PC-5p-9551_196
ENSSSCG00000011101	ITGB1	PC-5p-9551_196
ENSSSCG00000011101	ITGB1	PC-5p-9551_196
ENSSSCG00000011101	ITGB1	PC-5p-9551_196

ENSSSCG00000011101	ITGB1	PC-5p-9551_196
ENSSSCG00000011101	ITGB1	PC-5p-9551_196
ENSSSCG00000011101	ITGB1	PC-5p-9551_196
ENSSSCG00000002989	AKT2	PC-5p-9551_196
ENSSSCG00000002989	AKT2	PC-5p-9551_196
ENSSSCG00000002989	AKT2	PC-5p-9551_196
ENSSSCG00000002989	AKT2	PC-5p-9551_196
ENSSSCG00000002989	AKT2	PC-5p-9551_196
ENSSSCG00000002989	AKT2	PC-5p-9551_196
ENSSSCG00000002989	AKT2	PC-5p-9551_196
ENSSSCG00000002989	AKT2	PC-5p-9551_196
ENSSSCG00000002989	AKT2	PC-5p-9551_196
ENSSSCG00000002989	AKT2	PC-5p-9551_196
ENSSSCG00000002989	AKT2	PC-5p-9551_196
ENSSSCG00000009228	MAPK10	PC-5p-9551_196
ENSSSCG00000009228	MAPK10	PC-5p-9551_196
ENSSSCG00000009228	MAPK10	PC-5p-9551_196
ENSSSCG00000009228	MAPK10	PC-5p-9551_196
ENSSSCG00000009228	MAPK10	PC-5p-9551_196
ENSSSCG00000009228	MAPK10	PC-5p-9551_196
ENSSSCG00000009228	MAPK10	PC-5p-9551_196
ENSSSCG00000009228	MAPK10	PC-5p-9551_196
ENSSSCG00000009228	MAPK10	PC-5p-9551_196
ENSSSCG00000008844	KDR	PC-5p-9551_196
ENSSSCG00000008844	KDR	PC-5p-9551_196
ENSSSCG00000008844	KDR	PC-5p-9551_196
ENSSSCG00000008844	KDR	PC-5p-9551_196
ENSSSCG00000008844	KDR	PC-5p-9551_196
ENSSSCG00000008844	KDR	PC-5p-9551_196
ENSSSCG00000000233	ACVR1B	sha-mir-24-1-p3_1ss2GC
ENSSSCG00000000233	ACVR1B	sha-mir-24-1-p3_1ss2GC
ENSSSCG00000000233	ACVR1B	sha-mir-24-1-p3_1ss2GC
ENSSSCG00000000233	ACVR1B	sha-mir-24-1-p3_1ss2GC
ENSSSCG00000000233	ACVR1B	sha-mir-24-1-p3_1ss2GC
ENSSSCG00000000233	ACVR1B	sha-mir-24-1-p3_1ss2GC
ENSSSCG00000001404	NR5A1	sha-mir-24-1-p3_1ss2GC
ENSSSCG00000001404	NR5A1	sha-mir-24-1-p3_1ss2GC
ENSSSCG00000001404	NR5A1	sha-mir-24-1-p3_1ss2GC
ENSSSCG00000001404	NR5A1	sha-mir-24-1-p3_1ss2GC
ENSSSCG00000001404	NR5A1	sha-mir-24-1-p3_1ss2GC
ENSSSCG00000001404	NR5A1	sha-mir-24-1-p3_1ss2GC
ENSSSCG00000001404	NR5A1	sha-mir-24-1-p3_1ss2GC
ENSSSCG00000001404	NR5A1	sha-mir-24-1-p3_1ss2GC
ENSSSCG00000001404	NR5A1	sha-mir-24-1-p3_1ss2GC

ENSSSCG00000001404	NR5A1	sha-mir-24-1-p3_1ss2GC
ENSSSCG00000001404	NR5A1	sha-mir-24-1-p3_1ss2GC
ENSSSCG00000017412	RAB5C	sha-mir-24-1-p3_1ss2GC
ENSSSCG00000017412	RAB5C	sha-mir-24-1-p3_1ss2GC
ENSSSCG00000005751	COL5A1	ssc-miR-139-3p
ENSSSCG00000006145	E2F5	ssc-miR-139-3p
ENSSSCG00000006145	E2F5	ssc-miR-139-3p
ENSSSCG00000008845	SRD5A3	ssc-miR-139-3p
ENSSSCG00000010448	FAS	ssc-miR-139-3p
ENSSSCG00000010448	FAS	ssc-miR-139-3p
ENSSSCG00000010448	FAS	ssc-miR-139-3p
ENSSSCG00000010448	FAS	ssc-miR-139-3p
ENSSSCG00000010448	FAS	ssc-miR-139-3p
ENSSSCG00000012913	PPP1CA	ssc-miR-139-3p
ENSSSCG00000012913	PPP1CA	ssc-miR-139-3p
ENSSSCG00000012913	PPP1CA	ssc-miR-139-3p
ENSSSCG00000015383	RAPGEF5	ssc-miR-139-3p
ENSSSCG00000015383	RAPGEF5	ssc-miR-139-3p
ENSSSCG00000018016	MAP2K4	ssc-miR-139-3p
ENSSSCG00000018016	MAP2K4	ssc-miR-139-3p
ENSSSCG00000018016	MAP2K4	ssc-miR-139-3p
ENSSSCG00000021068	TRAF5	ssc-miR-139-3p
ENSSSCG00000021068	TRAF5	ssc-miR-139-3p
ENSSSCG00000012034	TIAM1	ssc-miR-139-3p
ENSSSCG00000012034	TIAM1	ssc-miR-139-3p
ENSSSCG00000012034	TIAM1	ssc-miR-139-3p
ENSSSCG00000008845	SRD5A3	ssc-miR-139-3p
ENSSSCG00000030172	CD40LG	ssc-miR-139-3p
ENSSSCG00000030172	CD40LG	ssc-miR-139-3p
ENSSSCG00000020672	F2R	ssc-miR-139-3p
ENSSSCG00000020672	F2R	ssc-miR-139-3p
ENSSSCG00000020672	F2R	ssc-miR-139-3p
ENSSSCG00000020672	F2R	ssc-miR-139-3p
ENSSSCG00000008845	SRD5A3	ssc-miR-139-3p
ENSSSCG00000021560	UBE2I	ssc-miR-139-3p
ENSSSCG00000021560	UBE2I	ssc-miR-139-3p
ENSSSCG00000021560	UBE2I	ssc-miR-139-3p
ENSSSCG00000012495	BTK	ssc-miR-139-3p
ENSSSCG00000012495	BTK	ssc-miR-139-3p
ENSSSCG00000012495	BTK	ssc-miR-139-3p
ENSSSCG00000012495	BTK	ssc-miR-139-3p
ENSSSCG00000012495	BTK	ssc-miR-139-3p
ENSSSCG00000012495	BTK	ssc-miR-139-3p
ENSSSCG00000012495	BTK	ssc-miR-139-3p

ENSSSCG00000012495	BTK	ssc-miR-139-3p
ENSSSCG00000012495	BTK	ssc-miR-139-3p
ENSSSCG00000016887	ITGA2	ssc-miR-139-3p
ENSSSCG00000016887	ITGA2	ssc-miR-139-3p
ENSSSCG00000016887	ITGA2	ssc-miR-139-3p
ENSSSCG00000016887	ITGA2	ssc-miR-139-3p
ENSSSCG00000016887	ITGA2	ssc-miR-139-3p
ENSSSCG00000016991	DUSP1	ssc-miR-139-3p
ENSSSCG00000000529	DNM1L	ssc-miR-1839-3p_R+2
ENSSSCG00000000529	DNM1L	ssc-miR-1839-3p_R+2
ENSSSCG00000000529	DNM1L	ssc-miR-1839-3p_R+2
ENSSSCG00000000529	DNM1L	ssc-miR-1839-3p_R+2
ENSSSCG00000003520	CDC42	ssc-miR-1839-3p_R+2
ENSSSCG00000003520	CDC42	ssc-miR-1839-3p_R+2
ENSSSCG00000003520	CDC42	ssc-miR-1839-3p_R+2
ENSSSCG00000003520	CDC42	ssc-miR-1839-3p_R+2
ENSSSCG00000003520	CDC42	ssc-miR-1839-3p_R+2
ENSSSCG00000003520	CDC42	ssc-miR-1839-3p_R+2
ENSSSCG00000003520	CDC42	ssc-miR-1839-3p_R+2
ENSSSCG00000003520	CDC42	ssc-miR-1839-3p_R+2
ENSSSCG00000003520	CDC42	ssc-miR-1839-3p_R+2
ENSSSCG00000004110	TAB2	ssc-miR-1839-3p_R+2
ENSSSCG00000004110	TAB2	ssc-miR-1839-3p_R+2
ENSSSCG00000004110	TAB2	ssc-miR-1839-3p_R+2
ENSSSCG00000004110	TAB2	ssc-miR-1839-3p_R+2
ENSSSCG00000006530	EFNA1	ssc-miR-1839-3p_R+2
ENSSSCG00000006530	EFNA1	ssc-miR-1839-3p_R+2
ENSSSCG00000006780	WNT2B	ssc-miR-1839-3p_R+2
ENSSSCG00000006780	WNT2B	ssc-miR-1839-3p_R+2
ENSSSCG00000006780	WNT2B	ssc-miR-1839-3p_R+2
ENSSSCG00000006780	WNT2B	ssc-miR-1839-3p_R+2
ENSSSCG00000008164	MAP4K4	ssc-miR-1839-3p_R+2
ENSSSCG00000010509	PIK3AP1	ssc-miR-1839-3p_R+2
ENSSSCG00000010509	PIK3AP1	ssc-miR-1839-3p_R+2
ENSSSCG00000010894	TP53BP2	ssc-miR-1839-3p_R+2
ENSSSCG00000014146	RASA1	ssc-miR-1839-3p_R+2
ENSSSCG00000014146	RASA1	ssc-miR-1839-3p_R+2
ENSSSCG00000014362	HBEGF	ssc-miR-1839-3p_R+2
ENSSSCG00000028968	RAC1	ssc-miR-1839-3p_R+2
ENSSSCG00000028968	RAC1	ssc-miR-1839-3p_R+2
ENSSSCG00000028968	RAC1	ssc-miR-1839-3p_R+2
ENSSSCG00000028968	RAC1	ssc-miR-1839-3p_R+2
ENSSSCG00000028968	RAC1	ssc-miR-1839-3p_R+2
ENSSSCG00000028968	RAC1	ssc-miR-1839-3p_R+2

ENSSSCG00000028968	RAC1	ssc-miR-1839-3p_R+2
ENSSSCG00000028968	RAC1	ssc-miR-1839-3p_R+2
ENSSSCG00000028968	RAC1	ssc-miR-1839-3p_R+2
ENSSSCG00000028968	RAC1	ssc-miR-1839-3p_R+2
ENSSSCG00000028968	RAC1	ssc-miR-1839-3p_R+2
ENSSSCG00000022482	DDIT3	ssc-miR-1839-3p_R+2
ENSSSCG00000000529	DNM1L	ssc-miR-1839-3p_R+2
ENSSSCG00000000529	DNM1L	ssc-miR-1839-3p_R+2
ENSSSCG00000025455	RALB	ssc-miR-1839-3p_R+2
ENSSSCG00000025455	RALB	ssc-miR-1839-3p_R+2
ENSSSCG00000025455	RALB	ssc-miR-1839-3p_R+2
ENSSSCG00000025455	RALB	ssc-miR-1839-3p_R+2
ENSSSCG00000025768	CALM1	ssc-miR-1839-3p_R+2
ENSSSCG00000025768	CALM1	ssc-miR-1839-3p_R+2
ENSSSCG00000025768	CALM1	ssc-miR-1839-3p_R+2
ENSSSCG00000003520	CDC42	ssc-miR-1839-3p_R+2
ENSSSCG00000003520	CDC42	ssc-miR-1839-3p_R+2
ENSSSCG00000003520	CDC42	ssc-miR-1839-3p_R+2
ENSSSCG00000003520	CDC42	ssc-miR-1839-3p_R+2
ENSSSCG00000003520	CDC42	ssc-miR-1839-3p_R+2
ENSSSCG00000003520	CDC42	ssc-miR-1839-3p_R+2
ENSSSCG00000003520	CDC42	ssc-miR-1839-3p_R+2
ENSSSCG00000003520	CDC42	ssc-miR-1839-3p_R+2
ENSSSCG00000003520	CDC42	ssc-miR-1839-3p_R+2
ENSSSCG00000016027	ITGAV	ssc-miR-1839-3p_R+2
ENSSSCG00000016027	ITGAV	ssc-miR-1839-3p_R+2
ENSSSCG00000016027	ITGAV	ssc-miR-1839-3p_R+2
ENSSSCG00000016027	ITGAV	ssc-miR-1839-3p_R+2
ENSSSCG00000016027	ITGAV	ssc-miR-1839-3p_R+2
ENSSSCG00000000529	DNM1L	ssc-miR-190b
ENSSSCG00000000529	DNM1L	ssc-miR-190b
ENSSSCG00000000529	DNM1L	ssc-miR-190b
ENSSSCG00000000529	DNM1L	ssc-miR-190b
ENSSSCG00000004154	TNFAIP3	ssc-miR-190b
ENSSSCG00000004154	TNFAIP3	ssc-miR-190b
ENSSSCG00000004896	PHLPP1	ssc-miR-190b
ENSSSCG00000006780	WNT2B	ssc-miR-190b
ENSSSCG00000006780	WNT2B	ssc-miR-190b
ENSSSCG00000006780	WNT2B	ssc-miR-190b
ENSSSCG00000006780	WNT2B	ssc-miR-190b
ENSSSCG00000007356	PLCG1	ssc-miR-190b
ENSSSCG00000007356	PLCG1	ssc-miR-190b
ENSSSCG00000007356	PLCG1	ssc-miR-190b
ENSSSCG00000007356	PLCG1	ssc-miR-190b

ENSSSCG00000007356	PLCG1	ssc-miR-190b
ENSSSCG00000007356	PLCG1	ssc-miR-190b
ENSSSCG00000007356	PLCG1	ssc-miR-190b
ENSSSCG00000012163	RPS6KA3	ssc-miR-190b
ENSSSCG00000012163	RPS6KA3	ssc-miR-190b
ENSSSCG00000015403	HGF	ssc-miR-190b
ENSSSCG00000015403	HGF	ssc-miR-190b
ENSSSCG00000015403	HGF	ssc-miR-190b
ENSSSCG00000015403	HGF	ssc-miR-190b
ENSSSCG00000015403	HGF	ssc-miR-190b
ENSSSCG00000015403	HGF	ssc-miR-190b
ENSSSCG00000015403	HGF	ssc-miR-190b
ENSSSCG00000015770	VEGFC	ssc-miR-190b
ENSSSCG00000015770	VEGFC	ssc-miR-190b
ENSSSCG00000015770	VEGFC	ssc-miR-190b
ENSSSCG00000015770	VEGFC	ssc-miR-190b
ENSSSCG00000015770	VEGFC	ssc-miR-190b
ENSSSCG00000016878	FGF10	ssc-miR-190b
ENSSSCG00000016878	FGF10	ssc-miR-190b
ENSSSCG00000016878	FGF10	ssc-miR-190b
ENSSSCG00000016878	FGF10	ssc-miR-190b
ENSSSCG00000016878	FGF10	ssc-miR-190b
ENSSSCG00000017670	RPS6KB1	ssc-miR-190b
ENSSSCG00000017670	RPS6KB1	ssc-miR-190b
ENSSSCG00000017670	RPS6KB1	ssc-miR-190b
ENSSSCG00000017670	RPS6KB1	ssc-miR-190b
ENSSSCG00000025777	ESR1	ssc-miR-190b
ENSSSCG00000020906	TNFSF10	ssc-miR-190b
ENSSSCG00000020906	TNFSF10	ssc-miR-190b
ENSSSCG00000000529	DNM1L	ssc-miR-190b
ENSSSCG00000004154	TNFAIP3	ssc-miR-190b
ENSSSCG00000004154	TNFAIP3	ssc-miR-190b
ENSSSCG00000025777	ESR1	ssc-miR-190b
ENSSSCG00000003194	NR5A1	ssc-miR-192
ENSSSCG00000009051	IL15	ssc-miR-192
ENSSSCG00000009051	IL15	ssc-miR-192
ENSSSCG00000014362	HBEGF	ssc-miR-192
ENSSSCG00000016628	WNT2	ssc-miR-192
ENSSSCG00000016628	WNT2	ssc-miR-192
ENSSSCG00000016628	WNT2	ssc-miR-192
ENSSSCG00000016628	WNT2	ssc-miR-192
ENSSSCG00000016832	IL7R	ssc-miR-192
ENSSSCG00000016918	MAP3K1	ssc-miR-192
ENSSSCG00000016918	MAP3K1	ssc-miR-192

ENSSSCG00000016918	MAP3K1	ssc-miR-192
ENSSSCG00000016918	MAP3K1	ssc-miR-192
ENSSSCG00000029852	WNT5A	ssc-miR-192
ENSSSCG00000029852	WNT5A	ssc-miR-192
ENSSSCG00000029852	WNT5A	ssc-miR-192
ENSSSCG00000029852	WNT5A	ssc-miR-192
ENSSSCG00000029828	RAB5B	ssc-miR-192
ENSSSCG00000029828	RAB5B	ssc-miR-192
ENSSSCG00000028420	EIF4E	ssc-miR-192
ENSSSCG00000028420	EIF4E	ssc-miR-192
ENSSSCG00000028420	EIF4E	ssc-miR-192
ENSSSCG00000009088	IL2	ssc-miR-192
ENSSSCG00000009088	IL2	ssc-miR-192
ENSSSCG00000009088	IL2	ssc-miR-192
ENSSSCG00000000529	DNM1L	ssc-miR-194a_R+2
ENSSSCG00000000529	DNM1L	ssc-miR-194a_R+2
ENSSSCG00000000529	DNM1L	ssc-miR-194a_R+2
ENSSSCG00000000529	DNM1L	ssc-miR-194a_R+2
ENSSSCG00000001050	EDN1	ssc-miR-194a_R+2
ENSSSCG00000001050	EDN1	ssc-miR-194a_R+2
ENSSSCG00000003863	ZFYVE9	ssc-miR-194a_R+2
ENSSSCG00000003863	ZFYVE9	ssc-miR-194a_R+2
ENSSSCG00000004018	AFDN	ssc-miR-194a_R+2
ENSSSCG00000004018	AFDN	ssc-miR-194a_R+2
ENSSSCG00000004789	THBS1	ssc-miR-194a_R+2
ENSSSCG00000004789	THBS1	ssc-miR-194a_R+2
ENSSSCG00000004789	THBS1	ssc-miR-194a_R+2
ENSSSCG00000004789	THBS1	ssc-miR-194a_R+2
ENSSSCG00000004789	THBS1	ssc-miR-194a_R+2
ENSSSCG00000006095	CCNE2	ssc-miR-194a_R+2
ENSSSCG00000006095	CCNE2	ssc-miR-194a_R+2
ENSSSCG00000006095	CCNE2	ssc-miR-194a_R+2
ENSSSCG00000007058	PLCB4	ssc-miR-194a_R+2
ENSSSCG00000007058	PLCB4	ssc-miR-194a_R+2
ENSSSCG00000007058	PLCB4	ssc-miR-194a_R+2
ENSSSCG00000007501	BMP7	ssc-miR-194a_R+2
ENSSSCG00000007501	BMP7	ssc-miR-194a_R+2
ENSSSCG00000014127	RASGRF2	ssc-miR-194a_R+2
ENSSSCG00000014127	RASGRF2	ssc-miR-194a_R+2
ENSSSCG00000014878	PAK1	ssc-miR-194a_R+2
ENSSSCG00000014878	PAK1	ssc-miR-194a_R+2
ENSSSCG00000014878	PAK1	ssc-miR-194a_R+2
ENSSSCG00000014878	PAK1	ssc-miR-194a_R+2
ENSSSCG00000014878	PAK1	ssc-miR-194a_R+2

ENSSSCG00000014878	PAK1	ssc-miR-194a_R+2
ENSSSCG00000016832	IL7R	ssc-miR-194a_R+2
ENSSSCG00000016991	DUSP1	ssc-miR-194a_R+2
ENSSSCG00000028968	RAC1	ssc-miR-194a_R+2
ENSSSCG00000028968	RAC1	ssc-miR-194a_R+2
ENSSSCG00000028968	RAC1	ssc-miR-194a_R+2
ENSSSCG00000028968	RAC1	ssc-miR-194a_R+2
ENSSSCG00000028968	RAC1	ssc-miR-194a_R+2
ENSSSCG00000028968	RAC1	ssc-miR-194a_R+2
ENSSSCG00000028968	RAC1	ssc-miR-194a_R+2
ENSSSCG00000028968	RAC1	ssc-miR-194a_R+2
ENSSSCG00000028968	RAC1	ssc-miR-194a_R+2
ENSSSCG00000028968	RAC1	ssc-miR-194a_R+2
ENSSSCG00000028968	RAC1	ssc-miR-194a_R+2
ENSSSCG00000027443	MRAS	ssc-miR-194a_R+2
ENSSSCG00000027443	MRAS	ssc-miR-194a_R+2
ENSSSCG00000027443	MRAS	ssc-miR-194a_R+2
ENSSSCG00000027443	MRAS	ssc-miR-194a_R+2
ENSSSCG00000027443	MRAS	ssc-miR-194a_R+2
ENSSSCG00000029852	WNT5A	ssc-miR-194a_R+2
ENSSSCG00000029852	WNT5A	ssc-miR-194a_R+2
ENSSSCG00000029852	WNT5A	ssc-miR-194a_R+2
ENSSSCG00000029852	WNT5A	ssc-miR-194a_R+2
ENSSSCG00000000529	DNM1L	ssc-miR-194a_R+2
ENSSSCG00000020672	F2R	ssc-miR-194a_R+2
ENSSSCG00000020672	F2R	ssc-miR-194a_R+2
ENSSSCG00000020672	F2R	ssc-miR-194a_R+2
ENSSSCG00000020672	F2R	ssc-miR-194a_R+2
ENSSSCG00000004917	MALT1	ssc-miR-194a_R+2
ENSSSCG00000004917	MALT1	ssc-miR-194a_R+2
ENSSSCG00000014878	PAK1	ssc-miR-194a_R+2
ENSSSCG00000014878	PAK1	ssc-miR-194a_R+2
ENSSSCG00000014878	PAK1	ssc-miR-194a_R+2
ENSSSCG00000014878	PAK1	ssc-miR-194a_R+2
ENSSSCG00000014878	PAK1	ssc-miR-194a_R+2
ENSSSCG00000014878	PAK1	ssc-miR-194a_R+2
ENSSSCG00000014878	PAK1	ssc-miR-194a_R+2
ENSSSCG00000014878	PAK1	ssc-miR-194a_R+2
ENSSSCG00000014878	PAK1	ssc-miR-194a_R+2
ENSSSCG00000014878	PAK1	ssc-miR-194a_R+2
ENSSSCG00000014878	PAK1	ssc-miR-194a_R+2
ENSSSCG00000000529	DNM1L	ssc-miR-194b-5p_1ss10GA
ENSSSCG00000001050	EDN1	ssc-miR-194b-5p_1ss10GA
ENSSSCG00000001050	EDN1	ssc-miR-194b-5p_1ss10GA

ENSSSCG00000003863	ZFYVE9	ssc-miR-194b-5p_1ss10GA
ENSSSCG00000004018	AFDN	ssc-miR-194b-5p_1ss10GA
ENSSSCG00000004018	AFDN	ssc-miR-194b-5p_1ss10GA
ENSSSCG00000004789	THBS1	ssc-miR-194b-5p_1ss10GA
ENSSSCG00000004789	THBS1	ssc-miR-194b-5p_1ss10GA
ENSSSCG00000004789	THBS1	ssc-miR-194b-5p_1ss10GA
ENSSSCG00000004789	THBS1	ssc-miR-194b-5p_1ss10GA
ENSSSCG00000004789	THBS1	ssc-miR-194b-5p_1ss10GA
ENSSSCG00000006095	CCNE2	ssc-miR-194b-5p_1ss10GA
ENSSSCG00000006095	CCNE2	ssc-miR-194b-5p_1ss10GA
ENSSSCG00000007058	PLCB4	ssc-miR-194b-5p_1ss10GA
ENSSSCG00000007058	PLCB4	ssc-miR-194b-5p_1ss10GA
ENSSSCG00000007058	PLCB4	ssc-miR-194b-5p_1ss10GA
ENSSSCG00000007501	BMP7	ssc-miR-194b-5p_1ss10GA
ENSSSCG00000007501	BMP7	ssc-miR-194b-5p_1ss10GA
ENSSSCG00000014127	RASGRF2	ssc-miR-194b-5p_1ss10GA
ENSSSCG00000014127	RASGRF2	ssc-miR-194b-5p_1ss10GA
ENSSSCG00000014878	PAK1	ssc-miR-194b-5p_1ss10GA
ENSSSCG00000014878	PAK1	ssc-miR-194b-5p_1ss10GA
ENSSSCG00000014878	PAK1	ssc-miR-194b-5p_1ss10GA
ENSSSCG00000014878	PAK1	ssc-miR-194b-5p_1ss10GA
ENSSSCG00000014878	PAK1	ssc-miR-194b-5p_1ss10GA
ENSSSCG00000016832	IL7R	ssc-miR-194b-5p_1ss10GA
ENSSSCG00000028968	RAC1	ssc-miR-194b-5p_1ss10GA
ENSSSCG00000028968	RAC1	ssc-miR-194b-5p_1ss10GA
ENSSSCG00000028968	RAC1	ssc-miR-194b-5p_1ss10GA
ENSSSCG00000028968	RAC1	ssc-miR-194b-5p_1ss10GA
ENSSSCG00000028968	RAC1	ssc-miR-194b-5p_1ss10GA
ENSSSCG00000028968	RAC1	ssc-miR-194b-5p_1ss10GA
ENSSSCG00000028968	RAC1	ssc-miR-194b-5p_1ss10GA
ENSSSCG00000028968	RAC1	ssc-miR-194b-5p_1ss10GA
ENSSSCG00000028968	RAC1	ssc-miR-194b-5p_1ss10GA
ENSSSCG00000028968	RAC1	ssc-miR-194b-5p_1ss10GA
ENSSSCG00000028968	RAC1	ssc-miR-194b-5p_1ss10GA
ENSSSCG00000027443	MRAS	ssc-miR-194b-5p_1ss10GA
ENSSSCG00000027443	MRAS	ssc-miR-194b-5p_1ss10GA
ENSSSCG00000027443	MRAS	ssc-miR-194b-5p_1ss10GA
ENSSSCG00000027443	MRAS	ssc-miR-194b-5p_1ss10GA
ENSSSCG00000027443	MRAS	ssc-miR-194b-5p_1ss10GA
ENSSSCG00000029852	WNT5A	ssc-miR-194b-5p_1ss10GA
ENSSSCG00000029852	WNT5A	ssc-miR-194b-5p_1ss10GA
ENSSSCG00000029852	WNT5A	ssc-miR-194b-5p_1ss10GA
ENSSSCG00000029852	WNT5A	ssc-miR-194b-5p_1ss10GA
ENSSSCG00000000529	DNM1L	ssc-miR-194b-5p_1ss10GA

ENSSSCG00000020672	F2R	ssc-miR-194b-5p_1ss10GA
ENSSSCG00000020672	F2R	ssc-miR-194b-5p_1ss10GA
ENSSSCG00000020672	F2R	ssc-miR-194b-5p_1ss10GA
ENSSSCG00000020672	F2R	ssc-miR-194b-5p_1ss10GA
ENSSSCG00000004917	MALT1	ssc-miR-194b-5p_1ss10GA
ENSSSCG00000004917	MALT1	ssc-miR-194b-5p_1ss10GA
ENSSSCG00000014878	PAK1	ssc-miR-194b-5p_1ss10GA
ENSSSCG00000014878	PAK1	ssc-miR-194b-5p_1ss10GA
ENSSSCG00000014878	PAK1	ssc-miR-194b-5p_1ss10GA
ENSSSCG00000014878	PAK1	ssc-miR-194b-5p_1ss10GA
ENSSSCG00000014878	PAK1	ssc-miR-194b-5p_1ss10GA
ENSSSCG00000014878	PAK1	ssc-miR-194b-5p_1ss10GA
ENSSSCG00000014878	PAK1	ssc-miR-194b-5p_1ss10GA
ENSSSCG00000014878	PAK1	ssc-miR-194b-5p_1ss10GA
ENSSSCG00000014878	PAK1	ssc-miR-194b-5p_1ss10GA
ENSSSCG00000014878	PAK1	ssc-miR-194b-5p_1ss10GA
ENSSSCG00000014878	PAK1	ssc-miR-194b-5p_1ss10GA
ENSSSCG00000003194	NR5A1	ssc-miR-215_R+1
ENSSSCG00000004110	TAB2	ssc-miR-215_R+1
ENSSSCG00000004110	TAB2	ssc-miR-215_R+1
ENSSSCG00000004110	TAB2	ssc-miR-215_R+1
ENSSSCG00000004110	TAB2	ssc-miR-215_R+1
ENSSSCG00000009051	IL15	ssc-miR-215_R+1
ENSSSCG00000009051	IL15	ssc-miR-215_R+1
ENSSSCG00000016628	WNT2	ssc-miR-215_R+1
ENSSSCG00000016628	WNT2	ssc-miR-215_R+1
ENSSSCG00000016628	WNT2	ssc-miR-215_R+1
ENSSSCG00000016628	WNT2	ssc-miR-215_R+1
ENSSSCG00000016832	IL7R	ssc-miR-215_R+1
ENSSSCG00000016918	MAP3K1	ssc-miR-215_R+1
ENSSSCG00000016918	MAP3K1	ssc-miR-215_R+1
ENSSSCG00000016918	MAP3K1	ssc-miR-215_R+1
ENSSSCG00000016918	MAP3K1	ssc-miR-215_R+1
ENSSSCG00000029852	WNT5A	ssc-miR-215_R+1
ENSSSCG00000029852	WNT5A	ssc-miR-215_R+1
ENSSSCG00000029852	WNT5A	ssc-miR-215_R+1
ENSSSCG00000029852	WNT5A	ssc-miR-215_R+1
ENSSSCG00000029828	RAB5B	ssc-miR-215_R+1
ENSSSCG00000029828	RAB5B	ssc-miR-215_R+1
ENSSSCG00000028420	EIF4E	ssc-miR-215_R+1
ENSSSCG00000028420	EIF4E	ssc-miR-215_R+1
ENSSSCG00000028420	EIF4E	ssc-miR-215_R+1
ENSSSCG00000009088	IL2	ssc-miR-215_R+1
ENSSSCG00000009088	IL2	ssc-miR-215_R+1

ENSSSCG00000009088	IL2	ssc-miR-215_R+1
ENSSSCG00000000233	ACVR1B	ssc-mir-4332-p5_1ss18CA
ENSSSCG00000000233	ACVR1B	ssc-mir-4332-p5_1ss18CA
ENSSSCG00000000233	ACVR1B	ssc-mir-4332-p5_1ss18CA
ENSSSCG00000000233	ACVR1B	ssc-mir-4332-p5_1ss18CA
ENSSSCG00000000233	ACVR1B	ssc-mir-4332-p5_1ss18CA
ENSSSCG00000001009	RIPK1	ssc-mir-4332-p5_1ss18CA
ENSSSCG00000001009	RIPK1	ssc-mir-4332-p5_1ss18CA
ENSSSCG00000001009	RIPK1	ssc-mir-4332-p5_1ss18CA
ENSSSCG00000001009	RIPK1	ssc-mir-4332-p5_1ss18CA
ENSSSCG00000001009	RIPK1	ssc-mir-4332-p5_1ss18CA
ENSSSCG00000001027	BMP6	ssc-mir-4332-p5_1ss18CA
ENSSSCG00000001027	BMP6	ssc-mir-4332-p5_1ss18CA
ENSSSCG00000001506	NR5A1	ssc-mir-4332-p5_1ss18CA
ENSSSCG00000002804	CSNK2A2	ssc-mir-4332-p5_1ss18CA
ENSSSCG00000004022	RPS6KA2	ssc-mir-4332-p5_1ss18CA
ENSSSCG00000004022	RPS6KA2	ssc-mir-4332-p5_1ss18CA
ENSSSCG00000005661	NR5A1	ssc-mir-4332-p5_1ss18CA
ENSSSCG00000007030	IKBKB	ssc-mir-4332-p5_1ss18CA
ENSSSCG00000007030	IKBKB	ssc-mir-4332-p5_1ss18CA
ENSSSCG00000007030	IKBKB	ssc-mir-4332-p5_1ss18CA
ENSSSCG00000007030	IKBKB	ssc-mir-4332-p5_1ss18CA
ENSSSCG00000007030	IKBKB	ssc-mir-4332-p5_1ss18CA
ENSSSCG00000007030	IKBKB	ssc-mir-4332-p5_1ss18CA
ENSSSCG00000007030	IKBKB	ssc-mir-4332-p5_1ss18CA
ENSSSCG00000007030	IKBKB	ssc-mir-4332-p5_1ss18CA
ENSSSCG00000007030	IKBKB	ssc-mir-4332-p5_1ss18CA
ENSSSCG00000007030	IKBKB	ssc-mir-4332-p5_1ss18CA
ENSSSCG00000007030	IKBKB	ssc-mir-4332-p5_1ss18CA
ENSSSCG00000009545	COL4A2	ssc-mir-4332-p5_1ss18CA
ENSSSCG00000009545	COL4A2	ssc-mir-4332-p5_1ss18CA
ENSSSCG00000009545	COL4A2	ssc-mir-4332-p5_1ss18CA
ENSSSCG00000012981	NR5A1	ssc-mir-4332-p5_1ss18CA
ENSSSCG00000012981	NR5A1	ssc-mir-4332-p5_1ss18CA
ENSSSCG00000012981	NR5A1	ssc-mir-4332-p5_1ss18CA
ENSSSCG00000012981	NR5A1	ssc-mir-4332-p5_1ss18CA
ENSSSCG00000012981	NR5A1	ssc-mir-4332-p5_1ss18CA
ENSSSCG00000012981	NR5A1	ssc-mir-4332-p5_1ss18CA
ENSSSCG00000012981	NR5A1	ssc-mir-4332-p5_1ss18CA
ENSSSCG00000012981	NR5A1	ssc-mir-4332-p5_1ss18CA
ENSSSCG00000012981	NR5A1	ssc-mir-4332-p5_1ss18CA
ENSSSCG00000012981	NR5A1	ssc-mir-4332-p5_1ss18CA
ENSSSCG00000012981	NR5A1	ssc-mir-4332-p5_1ss18CA
ENSSSCG00000013033	BAD	ssc-mir-4332-p5_1ss18CA

ENSSSCG00000013033	BAD	ssc-mir-4332-p5_1ss18CA
ENSSSCG00000013033	BAD	ssc-mir-4332-p5_1ss18CA
ENSSSCG00000013033	BAD	ssc-mir-4332-p5_1ss18CA
ENSSSCG00000013033	BAD	ssc-mir-4332-p5_1ss18CA
ENSSSCG00000013033	BAD	ssc-mir-4332-p5_1ss18CA
ENSSSCG00000013033	BAD	ssc-mir-4332-p5_1ss18CA
ENSSSCG00000013033	BAD	ssc-mir-4332-p5_1ss18CA
ENSSSCG00000013269	MAPK8IP1	ssc-mir-4332-p5_1ss18CA
ENSSSCG00000014339	CTNNA1	ssc-mir-4332-p5_1ss18CA
ENSSSCG00000014339	CTNNA1	ssc-mir-4332-p5_1ss18CA
ENSSSCG00000000107	CSNK1E	ssc-mir-4332-p5_1ss18CA
ENSSSCG00000021865	INHBA	ssc-mir-4332-p5_1ss18CA
ENSSSCG00000021865	INHBA	ssc-mir-4332-p5_1ss18CA
ENSSSCG00000007520	GNAS	ssc-mir-4332-p5_1ss18CA
ENSSSCG00000007520	GNAS	ssc-mir-4332-p5_1ss18CA
ENSSSCG00000007520	GNAS	ssc-mir-4332-p5_1ss18CA
ENSSSCG00000017482	CSF3	ssc-mir-4332-p5_1ss18CA
ENSSSCG00000007520	GNAS	ssc-mir-4332-p5_1ss18CA
ENSSSCG00000007520	GNAS	ssc-mir-4332-p5_1ss18CA
ENSSSCG00000007520	GNAS	ssc-mir-4332-p5_1ss18CA
ENSSSCG00000007520	GNAS	ssc-mir-4332-p5_1ss18CA
ENSSSCG00000007520	GNAS	ssc-mir-4332-p5_1ss18CA
ENSSSCG00000007520	GNAS	ssc-mir-4332-p5_1ss18CA
ENSSSCG00000007520	GNAS	ssc-mir-4332-p5_1ss18CA
ENSSSCG00000007520	GNAS	ssc-mir-4332-p5_1ss18CA
ENSSSCG00000007520	GNAS	ssc-mir-4332-p5_1ss18CA
ENSSSCG00000000293	ITGA5	ssc-mir-4332-p5_1ss18CA
ENSSSCG00000000293	ITGA5	ssc-mir-4332-p5_1ss18CA
ENSSSCG00000000293	ITGA5	ssc-mir-4332-p5_1ss18CA
ENSSSCG00000000293	ITGA5	ssc-mir-4332-p5_1ss18CA
ENSSSCG00000007520	GNAS	ssc-mir-4332-p5_1ss18CA
ENSSSCG00000007520	GNAS	ssc-mir-4332-p5_1ss18CA
ENSSSCG00000007520	GNAS	ssc-mir-4332-p5_1ss18CA
ENSSSCG00000007520	GNAS	ssc-mir-4332-p5_1ss18CA
ENSSSCG00000007520	GNAS	ssc-mir-4332-p5_1ss18CA
ENSSSCG00000007520	GNAS	ssc-mir-4332-p5_1ss18CA
ENSSSCG00000010872	AKT3	ssc-mir-4332-p5_1ss18CA
ENSSSCG00000010872	AKT3	ssc-mir-4332-p5_1ss18CA
ENSSSCG00000010872	AKT3	ssc-mir-4332-p5_1ss18CA
ENSSSCG00000010872	AKT3	ssc-mir-4332-p5_1ss18CA
ENSSSCG00000010872	AKT3	ssc-mir-4332-p5_1ss18CA
ENSSSCG00000010872	AKT3	ssc-mir-4332-p5_1ss18CA
ENSSSCG00000010872	AKT3	ssc-mir-4332-p5_1ss18CA
ENSSSCG00000010872	AKT3	ssc-mir-4332-p5_1ss18CA

ENSSSCG00000010872	AKT3	ssc-mir-4332-p5_1ss18CA
ENSSSCG00000010872	AKT3	ssc-mir-4332-p5_1ss18CA
ENSSSCG00000010872	AKT3	ssc-mir-4332-p5_1ss18CA
ENSSSCG00000007520	GNAS	ssc-mir-4332-p5_1ss18CA
ENSSSCG00000007520	GNAS	ssc-mir-4332-p5_1ss18CA
ENSSSCG00000007520	GNAS	ssc-mir-4332-p5_1ss18CA
ENSSSCG00000000293	ITGA5	ssc-mir-4332-p5_1ss18CA
ENSSSCG00000000293	ITGA5	ssc-mir-4332-p5_1ss18CA
ENSSSCG00000000293	ITGA5	ssc-mir-4332-p5_1ss18CA
ENSSSCG00000000293	ITGA5	ssc-mir-4332-p5_1ss18CA

Supplementary Table S6. Biological processes regulated by 13 differentially expressed miRNAs

Gene ID	Symbol	miRNA ID	GO
ENSSSCG00000003756	LPAR3	hsa-miR-141-3p_R+1	GO:0000187
ENSSSCG00000009048	GAB1	hsa-miR-141-3p_R+1	GO:0000187
ENSSSCG00000015403	HGF	hsa-miR-141-3p_R+1	GO:0000187
ENSSSCG00000030289	PTPN11	hsa-miR-141-3p_R+1	GO:0000187
ENSSSCG00000009048	GAB1	hsa-miR-141-3p_R+1	GO:0000187
ENSSSCG00000009048	GAB1	hsa-miR-141-3p_R+1	GO:0000187
ENSSSCG00000009048	GAB1	hsa-miR-141-3p_R+1	GO:0000187
ENSSSCG00000007692	YWHAG	hsa-miR-141-3p_R+1	GO:0003723
ENSSSCG00000006062	YWHAZ	hsa-miR-141-3p_R+1	GO:0003723
ENSSSCG00000006062	YWHAZ	hsa-miR-141-3p_R+1	GO:0003723
ENSSSCG00000006927	PKN2	hsa-miR-141-3p_R+1	GO:0003723
ENSSSCG00000023995	ENSSSCG00000023995	hsa-miR-141-3p_R+1	GO:0005025
ENSSSCG00000005382	TGFBR1	hsa-miR-141-3p_R+1	GO:0005025
ENSSSCG00000006095	CCNE2	hsa-miR-141-3p_R+1	GO:0005634
ENSSSCG00000006062	YWHAZ	hsa-miR-141-3p_R+1	GO:0005634
ENSSSCG00000007151	NR5A1	hsa-miR-141-3p_R+1	GO:0005634
ENSSSCG00000009131	PITX2	hsa-miR-141-3p_R+1	GO:0005634
ENSSSCG00000016991	DUSP1	hsa-miR-141-3p_R+1	GO:0005634
ENSSSCG00000008241	TCF7L1	hsa-miR-141-3p_R+1	GO:0005634
ENSSSCG00000022331	FGF13	hsa-miR-141-3p_R+1	GO:0005634
ENSSSCG00000030289	PTPN11	hsa-miR-141-3p_R+1	GO:0005634
ENSSSCG00000006927	PKN2	hsa-miR-141-3p_R+1	GO:0005634
ENSSSCG00000015407	GNAI1	hsa-miR-141-3p_R+1	GO:0005634
ENSSSCG00000020744	DUSP3	hsa-miR-141-3p_R+1	GO:0005634
ENSSSCG00000025028	XIAP	hsa-miR-141-3p_R+1	GO:0005634
ENSSSCG00000001009	RIPK1	hsa-miR-141-3p_R+1	GO:0005634
ENSSSCG00000016113	BMPR2	hsa-miR-141-3p_R+1	GO:0005654
ENSSSCG00000008241	TCF7L1	hsa-miR-141-3p_R+1	GO:0005654

ENSSSCG00000020744	DUSP3	hsa-miR-141-3p_R+1	GO:0005654
ENSSSCG00000015383	RAPGEF5	hsa-miR-141-3p_R+1	GO:0005654
ENSSSCG00000015407	GNAI1	hsa-miR-141-3p_R+1	GO:0005730
ENSSSCG00000004896	PHLPP1	hsa-miR-141-3p_R+1	GO:0005737
ENSSSCG00000007151	NR5A1	hsa-miR-141-3p_R+1	GO:0005737
ENSSSCG00000009131	PITX2	hsa-miR-141-3p_R+1	GO:0005737
ENSSSCG00000015407	GNAI1	hsa-miR-141-3p_R+1	GO:0005737
ENSSSCG00000016991	DUSP1	hsa-miR-141-3p_R+1	GO:0005737
ENSSSCG00000018016	MAP2K4	hsa-miR-141-3p_R+1	GO:0005737
ENSSSCG00000006286	SELE	hsa-miR-141-3p_R+1	GO:0005737
ENSSSCG00000006927	PKN2	hsa-miR-141-3p_R+1	GO:0005737
ENSSSCG00000008090	IL1A	hsa-miR-141-3p_R+1	GO:0005737
ENSSSCG00000009048	GAB1	hsa-miR-141-3p_R+1	GO:0005737
ENSSSCG00000009567	RASA3	hsa-miR-141-3p_R+1	GO:0005737
ENSSSCG00000017101	ADCY2	hsa-miR-141-3p_R+1	GO:0005737
ENSSSCG00000022331	FGF13	hsa-miR-141-3p_R+1	GO:0005737
ENSSSCG00000030289	PTPN11	hsa-miR-141-3p_R+1	GO:0005737
ENSSSCG00000028420	EIF4E	hsa-miR-141-3p_R+1	GO:0005737
ENSSSCG00000009048	GAB1	hsa-miR-141-3p_R+1	GO:0005737
ENSSSCG00000009048	GAB1	hsa-miR-141-3p_R+1	GO:0005737
ENSSSCG00000007356	PLCG1	hsa-miR-141-3p_R+1	GO:0005737
ENSSSCG00000008881	RAPGEF2	hsa-miR-141-3p_R+1	GO:0005737
ENSSSCG00000011014	BAMBI	hsa-miR-141-3p_R+1	GO:0005737
ENSSSCG00000011274	CTNNB1	hsa-miR-141-3p_R+1	GO:0005737
ENSSSCG00000025028	XIAP	hsa-miR-141-3p_R+1	GO:0005737
ENSSSCG00000001009	RIPK1	hsa-miR-141-3p_R+1	GO:0005739
ENSSSCG00000001009	RIPK1	hsa-miR-141-3p_R+1	GO:0005829
ENSSSCG00000003471	EPHA2	hsa-miR-141-3p_R+1	GO:0005829
ENSSSCG00000004110	TAB2	hsa-miR-141-3p_R+1	GO:0005829
ENSSSCG00000004622	GNB5	hsa-miR-141-3p_R+1	GO:0005829
ENSSSCG00000007356	PLCG1	hsa-miR-141-3p_R+1	GO:0005829
ENSSSCG00000008090	IL1A	hsa-miR-141-3p_R+1	GO:0005829
ENSSSCG00000008241	TCF7L1	hsa-miR-141-3p_R+1	GO:0005829
ENSSSCG00000008881	RAPGEF2	hsa-miR-141-3p_R+1	GO:0005829
ENSSSCG00000011274	CTNNB1	hsa-miR-141-3p_R+1	GO:0005829
ENSSSCG00000025396	YAP1	hsa-miR-141-3p_R+1	GO:0005829
ENSSSCG00000020744	DUSP3	hsa-miR-141-3p_R+1	GO:0005829
ENSSSCG00000004622	GNB5	hsa-miR-141-3p_R+1	GO:0005829
ENSSSCG00000012034	TIAM1	hsa-miR-141-3p_R+1	GO:0005829
ENSSSCG00000022331	FGF13	hsa-miR-141-3p_R+1	GO:0005829
ENSSSCG00000028420	EIF4E	hsa-miR-141-3p_R+1	GO:0005829
ENSSSCG00000016027	ITGAV	hsa-miR-141-3p_R+1	GO:0005829
ENSSSCG00000001009	RIPK1	hsa-miR-141-3p_R+1	GO:0006915
ENSSSCG00000025028	XIAP	hsa-miR-141-3p_R+1	GO:0006974

ENSSSCG00000025396	YAP1	hsa-miR-141-3p_R+1	GO:0006974
ENSSSCG00000005382	TGFBR1	hsa-miR-141-3p_R+1	GO:0007179
ENSSSCG00000015383	RAPGEF5	hsa-miR-141-3p_R+1	GO:0007264
ENSSSCG00000027443	MRAS	hsa-miR-141-3p_R+1	GO:0007264
ENSSSCG00000008881	RAPGEF2	hsa-miR-141-3p_R+1	GO:0007264
ENSSSCG00000015770	VEGFC	hsa-miR-141-3p_R+1	GO:0008284
ENSSSCG00000029852	WNT5A	hsa-miR-141-3p_R+1	GO:0008284
ENSSSCG00000011014	BAMBI	hsa-miR-141-3p_R+1	GO:0008284
ENSSSCG00000015403	HGF	hsa-miR-141-3p_R+1	GO:0008284
ENSSSCG00000012034	TIAM1	hsa-miR-141-3p_R+1	GO:0008284
ENSSSCG00000016027	ITGAV	hsa-miR-141-3p_R+1	GO:0008284
ENSSSCG00000025396	YAP1	hsa-miR-141-3p_R+1	GO:0008284
ENSSSCG00000008977	CXCL10	hsa-miR-141-3p_R+1	GO:0009897
ENSSSCG00000016887	ITGA2	hsa-miR-141-3p_R+1	GO:0009897
ENSSSCG00000008977	CXCL10	hsa-miR-141-3p_R+1	GO:0009897
ENSSSCG00000006862	VCAM1	hsa-miR-141-3p_R+1	GO:0009897
ENSSSCG00000010414	CXCL12	hsa-miR-141-3p_R+1	GO:0009897
ENSSSCG00000016027	ITGAV	hsa-miR-141-3p_R+1	GO:0009897
ENSSSCG00000003471	EPHA2	hsa-miR-141-3p_R+1	GO:0016477
ENSSSCG00000029852	WNT5A	hsa-miR-141-3p_R+1	GO:0016477
ENSSSCG00000007356	PLCG1	hsa-miR-141-3p_R+1	GO:0016477
ENSSSCG00000011014	BAMBI	hsa-miR-141-3p_R+1	GO:0016477
ENSSSCG00000003471	EPHA2	hsa-miR-141-3p_R+1	GO:0016477
ENSSSCG00000003471	EPHA2	hsa-miR-141-3p_R+1	GO:0016740
ENSSSCG00000016113	BMPR2	hsa-miR-141-3p_R+1	GO:0016740
ENSSSCG00000006927	PKN2	hsa-miR-141-3p_R+1	GO:0016740
ENSSSCG00000005382	TGFBR1	hsa-miR-141-3p_R+1	GO:0016740
ENSSSCG00000003756	LPAR3	hsa-miR-141-3p_R+1	GO:0016740
ENSSSCG00000006927	PKN2	hsa-miR-141-3p_R+1	GO:0035556
ENSSSCG00000009567	RASA3	hsa-miR-141-3p_R+1	GO:0035556
ENSSSCG00000017101	ADCY2	hsa-miR-141-3p_R+1	GO:0035556
ENSSSCG00000005382	TGFBR1	hsa-miR-141-3p_R+1	GO:0035556
ENSSSCG00000007356	PLCG1	hsa-miR-141-3p_R+1	GO:0035556
ENSSSCG00000015383	RAPGEF5	hsa-miR-141-3p_R+1	GO:0035556
ENSSSCG00000012034	TIAM1	hsa-miR-141-3p_R+1	GO:0035556
ENSSSCG00000006062	YWHAZ	hsa-miR-141-3p_R+1	GO:0042802
ENSSSCG00000020906	TNFSF10	hsa-miR-141-3p_R+1	GO:0043065
ENSSSCG00000001009	RIPK1	hsa-miR-141-3p_R+1	GO:0043065
ENSSSCG00000011274	CTNNB1	hsa-miR-141-3p_R+1	GO:0043065
ENSSSCG00000004110	TAB2	hsa-miR-141-3p_R+1	GO:0043123
ENSSSCG00000020906	TNFSF10	hsa-miR-141-3p_R+1	GO:0043123
ENSSSCG00000001009	RIPK1	hsa-miR-141-3p_R+1	GO:0043123
ENSSSCG00000011274	CTNNB1	hsa-miR-141-3p_R+1	GO:0043123
ENSSSCG00000008090	IL1A	hsa-miR-141-3p_R+1	GO:0045944

ENSSSCG00000009131	PITX2	hsa-miR-141-3p_R+1	GO:0045944
ENSSSCG00000016113	BMPR2	hsa-miR-141-3p_R+1	GO:0045944
ENSSSCG00000029852	WNT5A	hsa-miR-141-3p_R+1	GO:0045944
ENSSSCG00000004110	TAB2	hsa-miR-141-3p_R+1	GO:0045944
ENSSSCG00000011274	CTNNB1	hsa-miR-141-3p_R+1	GO:0045944
ENSSSCG00000025396	YAP1	hsa-miR-141-3p_R+1	GO:0045944
ENSSSCG00000006286	SELE	hsa-miR-141-3p_R+1	GO:0048471
ENSSSCG00000006286	SELE	hsa-miR-141-3p_R+1	GO:0048471
ENSSSCG00000008881	RAPGEF2	hsa-miR-141-3p_R+1	GO:0048471
ENSSSCG00000028420	EIF4E	hsa-miR-141-3p_R+1	GO:0048471
ENSSSCG00000004622	GNB5	hsa-miR-141-3p_R+1	GO:0048471
ENSSSCG00000004896	PHLPP1	hsa-miR-141-3p_R+1	GO:0048471
ENSSSCG00000006062	YWHAZ	hsa-miR-141-3p_R+1	GO:0048471
ENSSSCG00000015407	GNAI1	hsa-miR-141-3p_R+1	GO:0070062
ENSSSCG00000027443	MRAS	hsa-miR-141-3p_R+1	GO:0070062
ENSSSCG00000006062	YWHAZ	hsa-miR-141-3p_R+1	GO:0070062
ENSSSCG00000006862	VCAM1	hsa-miR-141-3p_R+1	GO:0070062
ENSSSCG00000007692	YWHAG	hsa-miR-141-3p_R+1	GO:0070062
ENSSSCG00000010414	CXCL12	hsa-miR-141-3p_R+1	GO:0070062
ENSSSCG00000020744	DUSP3	hsa-miR-141-3p_R+1	GO:0070062
ENSSSCG00000020906	TNFSF10	hsa-miR-141-3p_R+1	GO:0070062
ENSSSCG00000028420	EIF4E	hsa-miR-141-3p_R+1	GO:0070062
ENSSSCG00000016027	ITGAV	hsa-miR-141-3p_R+1	GO:0070062
ENSSSCG00000006095	CCNE2	hsa-miR-141-3p_R+1	GO:0070062
ENSSSCG00000029852	WNT5A	hsa-miR-141-3p_R+1	GO:0071560
ENSSSCG00000025396	YAP1	hsa-miR-141-3p_R+1	GO:0090263
ENSSSCG00000011014	BAMBI	hsa-miR-141-3p_R+1	GO:0090263
ENSSSCG00000025028	XIAP	hsa-miR-141-3p_R+1	GO:0090263
ENSSSCG00000016027	ITGAV	hsa-miR-141-3p_R+1	GO:2001237
ENSSSCG00000024954	FGF1	hsa-miR-222-5p_L+2R-1	GO:0000187
ENSSSCG00000016878	FGF10	hsa-miR-222-5p_L+2R-1	GO:0000187
ENSSSCG00000006145	E2F5	hsa-miR-222-5p_L+2R-1	GO:0001650
ENSSSCG00000006927	PKN2	hsa-miR-222-5p_L+2R-1	GO:0003723
ENSSSCG00000023995	ENSSSCG00000023995	hsa-miR-222-5p_L+2R-1	GO:0005025
ENSSSCG00000002804	CSNK2A2	hsa-miR-222-5p_L+2R-1	GO:0005634
ENSSSCG00000006145	E2F5	hsa-miR-222-5p_L+2R-1	GO:0005634
ENSSSCG00000001518	ITPR3	hsa-miR-222-5p_L+2R-1	GO:0005634
ENSSSCG00000005738	RALGDS	hsa-miR-222-5p_L+2R-1	GO:0005634
ENSSSCG00000024312	ID4	hsa-miR-222-5p_L+2R-1	GO:0005634
ENSSSCG00000006927	PKN2	hsa-miR-222-5p_L+2R-1	GO:0005634
ENSSSCG00000009228	MAPK10	hsa-miR-222-5p_L+2R-1	GO:0005634
ENSSSCG00000016878	FGF10	hsa-miR-222-5p_L+2R-1	GO:0005634
ENSSSCG00000022482	DDIT3	hsa-miR-222-5p_L+2R-1	GO:0005634
ENSSSCG00000001518	ITPR3	hsa-miR-222-5p_L+2R-1	GO:0005654

ENSSSCG00000009228	MAPK10	hsa-miR-222-5p_L+2R-1	GO:0005654
ENSSSCG00000027426	NR5A1	hsa-miR-222-5p_L+2R-1	GO:0005654
ENSSSCG00000004952	SMAD3	hsa-miR-222-5p_L+2R-1	GO:0005654
ENSSSCG00000024954	FGF1	hsa-miR-222-5p_L+2R-1	GO:0005654
ENSSSCG00000015815	NR5A1	hsa-miR-222-5p_L+2R-1	GO:0005730
ENSSSCG00000015815	NR5A1	hsa-miR-222-5p_L+2R-1	GO:0005730
ENSSSCG00000015815	NR5A1	hsa-miR-222-5p_L+2R-1	GO:0005730
ENSSSCG00000015815	NR5A1	hsa-miR-222-5p_L+2R-1	GO:0005730
ENSSSCG00000015815	NR5A1	hsa-miR-222-5p_L+2R-1	GO:0005730
ENSSSCG00000001518	ITPR3	hsa-miR-222-5p_L+2R-1	GO:0005737
ENSSSCG00000001901	CYP1A2	hsa-miR-222-5p_L+2R-1	GO:0005737
ENSSSCG00000006145	E2F5	hsa-miR-222-5p_L+2R-1	GO:0005737
ENSSSCG00000009228	MAPK10	hsa-miR-222-5p_L+2R-1	GO:0005737
ENSSSCG00000030153	SMURF1	hsa-miR-222-5p_L+2R-1	GO:0005737
ENSSSCG00000002707	GABARAPL2	hsa-miR-222-5p_L+2R-1	GO:0005737
ENSSSCG00000006927	PKN2	hsa-miR-222-5p_L+2R-1	GO:0005737
ENSSSCG00000014891	NR5A1	hsa-miR-222-5p_L+2R-1	GO:0005737
ENSSSCG00000024312	ID4	hsa-miR-222-5p_L+2R-1	GO:0005737
ENSSSCG00000027426	NR5A1	hsa-miR-222-5p_L+2R-1	GO:0005737
ENSSSCG00000004952	SMAD3	hsa-miR-222-5p_L+2R-1	GO:0005737
ENSSSCG00000007356	PLCG1	hsa-miR-222-5p_L+2R-1	GO:0005737
ENSSSCG00000011014	BAMBI	hsa-miR-222-5p_L+2R-1	GO:0005737
ENSSSCG00000026969	KRAS	hsa-miR-222-5p_L+2R-1	GO:0005737
ENSSSCG00000022482	DDIT3	hsa-miR-222-5p_L+2R-1	GO:0005737
ENSSSCG00000025092	CDK4	hsa-miR-222-5p_L+2R-1	GO:0005737
ENSSSCG00000024813	MAP3K3	hsa-miR-222-5p_L+2R-1	GO:0005737
ENSSSCG00000016101	CFLAR	hsa-miR-222-5p_L+2R-1	GO:0005737
ENSSSCG00000003017	TGFB1	hsa-miR-222-5p_L+2R-1	GO:0005737
ENSSSCG00000016101	CFLAR	hsa-miR-222-5p_L+2R-1	GO:0005737
ENSSSCG00000017748	NF1	hsa-miR-222-5p_L+2R-1	GO:0005737
ENSSSCG00000003017	TGFB1	hsa-miR-222-5p_L+2R-1	GO:0005737
ENSSSCG00000030153	SMURF1	hsa-miR-222-5p_L+2R-1	GO:0005739
ENSSSCG00000001518	ITPR3	hsa-miR-222-5p_L+2R-1	GO:0005739
ENSSSCG00000002707	GABARAPL2	hsa-miR-222-5p_L+2R-1	GO:0005776
ENSSSCG00000004952	SMAD3	hsa-miR-222-5p_L+2R-1	GO:0005829
ENSSSCG00000005738	RALGDS	hsa-miR-222-5p_L+2R-1	GO:0005829
ENSSSCG00000007356	PLCG1	hsa-miR-222-5p_L+2R-1	GO:0005829
ENSSSCG00000009855	KSR2	hsa-miR-222-5p_L+2R-1	GO:0005829
ENSSSCG00000012034	TIAM1	hsa-miR-222-5p_L+2R-1	GO:0005829
ENSSSCG00000025092	CDK4	hsa-miR-222-5p_L+2R-1	GO:0005829
ENSSSCG00000027426	NR5A1	hsa-miR-222-5p_L+2R-1	GO:0005829
ENSSSCG00000024954	FGF1	hsa-miR-222-5p_L+2R-1	GO:0005829
ENSSSCG00000022482	DDIT3	hsa-miR-222-5p_L+2R-1	GO:0006915
ENSSSCG00000016101	CFLAR	hsa-miR-222-5p_L+2R-1	GO:0006915

ENSSSCG00000016101	CFLAR	hsa-miR-222-5p_L+2R-1	GO:0006915
ENSSSCG00000022482	DDIT3	hsa-miR-222-5p_L+2R-1	GO:0006915
ENSSSCG00000003017	TGFB1	hsa-miR-222-5p_L+2R-1	GO:0007179
ENSSSCG00000004952	SMAD3	hsa-miR-222-5p_L+2R-1	GO:0007179
ENSSSCG00000003017	TGFB1	hsa-miR-222-5p_L+2R-1	GO:0007179
ENSSSCG00000026969	KRAS	hsa-miR-222-5p_L+2R-1	GO:0007264
ENSSSCG00000027443	MRAS	hsa-miR-222-5p_L+2R-1	GO:0007264
ENSSSCG00000005738	RALGDS	hsa-miR-222-5p_L+2R-1	GO:0007264
ENSSSCG00000003017	TGFB1	hsa-miR-222-5p_L+2R-1	GO:0008284
ENSSSCG00000014891	NR5A1	hsa-miR-222-5p_L+2R-1	GO:0008284
ENSSSCG00000015815	NR5A1	hsa-miR-222-5p_L+2R-1	GO:0008284
ENSSSCG00000024312	ID4	hsa-miR-222-5p_L+2R-1	GO:0008284
ENSSSCG00000015815	NR5A1	hsa-miR-222-5p_L+2R-1	GO:0008284
ENSSSCG00000015815	NR5A1	hsa-miR-222-5p_L+2R-1	GO:0008284
ENSSSCG00000015815	NR5A1	hsa-miR-222-5p_L+2R-1	GO:0008284
ENSSSCG00000003017	TGFB1	hsa-miR-222-5p_L+2R-1	GO:0008284
ENSSSCG00000015815	NR5A1	hsa-miR-222-5p_L+2R-1	GO:0008284
ENSSSCG00000011014	BAMBI	hsa-miR-222-5p_L+2R-1	GO:0008284
ENSSSCG00000016878	FGF10	hsa-miR-222-5p_L+2R-1	GO:0008284
ENSSSCG00000012034	TIAM1	hsa-miR-222-5p_L+2R-1	GO:0008284
ENSSSCG00000026969	KRAS	hsa-miR-222-5p_L+2R-1	GO:0008284
ENSSSCG00000025092	CDK4	hsa-miR-222-5p_L+2R-1	GO:0008284
ENSSSCG00000024954	FGF1	hsa-miR-222-5p_L+2R-1	GO:0008284
ENSSSCG00000022849	IL2RA	hsa-miR-222-5p_L+2R-1	GO:0009897
ENSSSCG00000016887	ITGA2	hsa-miR-222-5p_L+2R-1	GO:0009897
ENSSSCG00000012591	AMOT	hsa-miR-222-5p_L+2R-1	GO:0009897
ENSSSCG00000012591	AMOT	hsa-miR-222-5p_L+2R-1	GO:0009897
ENSSSCG00000013655	ICAM1	hsa-miR-222-5p_L+2R-1	GO:0009897
ENSSSCG00000013655	ICAM1	hsa-miR-222-5p_L+2R-1	GO:0009897
ENSSSCG00000001901	CYP1A2	hsa-miR-222-5p_L+2R-1	GO:0009897
ENSSSCG00000004192	CTGF	hsa-miR-222-5p_L+2R-1	GO:0016477
ENSSSCG00000013888	JAK3	hsa-miR-222-5p_L+2R-1	GO:0016477
ENSSSCG00000013888	JAK3	hsa-miR-222-5p_L+2R-1	GO:0016477
ENSSSCG00000007356	PLCG1	hsa-miR-222-5p_L+2R-1	GO:0016477
ENSSSCG00000011014	BAMBI	hsa-miR-222-5p_L+2R-1	GO:0016477
ENSSSCG00000002707	GABARAPL2	hsa-miR-222-5p_L+2R-1	GO:0016477
ENSSSCG00000002804	CSNK2A2	hsa-miR-222-5p_L+2R-1	GO:0016477
ENSSSCG00000003017	TGFB1	hsa-miR-222-5p_L+2R-1	GO:0016477
ENSSSCG00000013888	JAK3	hsa-miR-222-5p_L+2R-1	GO:0016740
ENSSSCG00000015815	NR5A1	hsa-miR-222-5p_L+2R-1	GO:0016740
ENSSSCG00000015815	NR5A1	hsa-miR-222-5p_L+2R-1	GO:0016740
ENSSSCG00000013888	JAK3	hsa-miR-222-5p_L+2R-1	GO:0016740
ENSSSCG00000015815	NR5A1	hsa-miR-222-5p_L+2R-1	GO:0016740
ENSSSCG00000015815	NR5A1	hsa-miR-222-5p_L+2R-1	GO:0016740

ENSSSCG00000015815	NR5A1	hsa-miR-222-5p_L+2R-1	GO:0016740
ENSSSCG00000006927	PKN2	hsa-miR-222-5p_L+2R-1	GO:0016740
ENSSSCG00000009228	MAPK10	hsa-miR-222-5p_L+2R-1	GO:0016740
ENSSSCG00000030153	SMURF1	hsa-miR-222-5p_L+2R-1	GO:0016740
ENSSSCG00000025092	CDK4	hsa-miR-222-5p_L+2R-1	GO:0016740
ENSSSCG00000006927	PKN2	hsa-miR-222-5p_L+2R-1	GO:0035556
ENSSSCG00000013888	JAK3	hsa-miR-222-5p_L+2R-1	GO:0035556
ENSSSCG00000013888	JAK3	hsa-miR-222-5p_L+2R-1	GO:0035556
ENSSSCG00000024813	MAP3K3	hsa-miR-222-5p_L+2R-1	GO:0035556
ENSSSCG00000007356	PLCG1	hsa-miR-222-5p_L+2R-1	GO:0035556
ENSSSCG00000009855	KSR2	hsa-miR-222-5p_L+2R-1	GO:0035556
ENSSSCG00000012034	TIAM1	hsa-miR-222-5p_L+2R-1	GO:0035556
ENSSSCG00000015815	NR5A1	hsa-miR-222-5p_L+2R-1	GO:0042802
ENSSSCG00000015815	NR5A1	hsa-miR-222-5p_L+2R-1	GO:0042802
ENSSSCG00000015815	NR5A1	hsa-miR-222-5p_L+2R-1	GO:0042802
ENSSSCG00000015815	NR5A1	hsa-miR-222-5p_L+2R-1	GO:0042802
ENSSSCG00000017748	NF1	hsa-miR-222-5p_L+2R-1	GO:0043065
ENSSSCG00000017748	NF1	hsa-miR-222-5p_L+2R-1	GO:0043065
ENSSSCG00000016101	CFLAR	hsa-miR-222-5p_L+2R-1	GO:0043123
ENSSSCG00000021068	TRAF5	hsa-miR-222-5p_L+2R-1	GO:0043123
ENSSSCG00000024813	MAP3K3	hsa-miR-222-5p_L+2R-1	GO:0043123
ENSSSCG00000004192	CTGF	hsa-miR-222-5p_L+2R-1	GO:0043231
ENSSSCG00000001901	CYP1A2	hsa-miR-222-5p_L+2R-1	GO:0043231
ENSSSCG00000027426	NR5A1	hsa-miR-222-5p_L+2R-1	GO:0043231
ENSSSCG00000024312	ID4	hsa-miR-222-5p_L+2R-1	GO:0045944
ENSSSCG00000004192	CTGF	hsa-miR-222-5p_L+2R-1	GO:0045944
ENSSSCG00000003017	TGFB1	hsa-miR-222-5p_L+2R-1	GO:0045944
ENSSSCG00000004952	SMAD3	hsa-miR-222-5p_L+2R-1	GO:0045944
ENSSSCG00000022482	DDIT3	hsa-miR-222-5p_L+2R-1	GO:0045944
ENSSSCG00000027426	NR5A1	hsa-miR-222-5p_L+2R-1	GO:0045944
ENSSSCG00000024954	FGF1	hsa-miR-222-5p_L+2R-1	GO:0045944
ENSSSCG00000003017	TGFB1	hsa-miR-222-5p_L+2R-1	GO:0045944
ENSSSCG00000016878	FGF10	hsa-miR-222-5p_L+2R-1	GO:0046579
ENSSSCG00000004952	SMAD3	hsa-miR-222-5p_L+2R-1	GO:0048471
ENSSSCG00000005738	RALGDS	hsa-miR-222-5p_L+2R-1	GO:0048471
ENSSSCG00000027443	MRAS	hsa-miR-222-5p_L+2R-1	GO:0070062
ENSSSCG00000030153	SMURF1	hsa-miR-222-5p_L+2R-1	GO:0070062
ENSSSCG00000013655	ICAM1	hsa-miR-222-5p_L+2R-1	GO:0070062
ENSSSCG00000013655	ICAM1	hsa-miR-222-5p_L+2R-1	GO:0070062
ENSSSCG00000003017	TGFB1	hsa-miR-222-5p_L+2R-1	GO:0071560
ENSSSCG00000003017	TGFB1	hsa-miR-222-5p_L+2R-1	GO:0071560
ENSSSCG00000011014	BAMBI	hsa-miR-222-5p_L+2R-1	GO:0090263
ENSSSCG00000017748	NF1	hsa-miR-222-5p_L+2R-1	GO:1902043
ENSSSCG00000017748	NF1	hsa-miR-222-5p_L+2R-1	GO:1902043

ENSSSCG00000016101	CFLAR	hsa-miR-222-5p_L+2R-1	GO:2001237
ENSSSCG00000016101	CFLAR	hsa-miR-222-5p_L+2R-1	GO:2001237
ENSSSCG00000006145	E2F5	hsa-miR-222-5p_L+2R-1	GO:2001237
ENSSSCG00000017748	NF1	hsa-miR-222-5p_L+2R-1	GO:2001241
ENSSSCG00000017748	NF1	hsa-miR-222-5p_L+2R-1	GO:2001241
ENSSSCG00000008842	KIT	hsa-miR-4792_1ss9GT	GO:0000187
ENSSSCG00000004507	SMAD7	hsa-miR-4792_1ss9GT	GO:0001650
ENSSSCG00000006927	PKN2	hsa-miR-4792_1ss9GT	GO:0003723
ENSSSCG00000015850	DUSP4	hsa-miR-4792_1ss9GT	GO:0005634
ENSSSCG00000001518	ITPR3	hsa-miR-4792_1ss9GT	GO:0005634
ENSSSCG00000011415	MAPKAPK3	hsa-miR-4792_1ss9GT	GO:0005634
ENSSSCG00000012399	FOXO4	hsa-miR-4792_1ss9GT	GO:0005634
ENSSSCG00000022066	PIAS4	hsa-miR-4792_1ss9GT	GO:0005634
ENSSSCG00000022689	GADD45B	hsa-miR-4792_1ss9GT	GO:0005634
ENSSSCG00000004022	RPS6KA2	hsa-miR-4792_1ss9GT	GO:0005634
ENSSSCG00000006927	PKN2	hsa-miR-4792_1ss9GT	GO:0005634
ENSSSCG00000012399	FOXO4	hsa-miR-4792_1ss9GT	GO:0005634
ENSSSCG00000015850	DUSP4	hsa-miR-4792_1ss9GT	GO:0005654
ENSSSCG00000001518	ITPR3	hsa-miR-4792_1ss9GT	GO:0005654
ENSSSCG00000011415	MAPKAPK3	hsa-miR-4792_1ss9GT	GO:0005654
ENSSSCG00000022066	PIAS4	hsa-miR-4792_1ss9GT	GO:0005654
ENSSSCG00000001518	ITPR3	hsa-miR-4792_1ss9GT	GO:0005737
ENSSSCG00000001898	ULK3	hsa-miR-4792_1ss9GT	GO:0005737
ENSSSCG00000011415	MAPKAPK3	hsa-miR-4792_1ss9GT	GO:0005737
ENSSSCG00000022066	PIAS4	hsa-miR-4792_1ss9GT	GO:0005737
ENSSSCG00000004022	RPS6KA2	hsa-miR-4792_1ss9GT	GO:0005737
ENSSSCG00000006927	PKN2	hsa-miR-4792_1ss9GT	GO:0005737
ENSSSCG00000012399	FOXO4	hsa-miR-4792_1ss9GT	GO:0005737
ENSSSCG00000022689	GADD45B	hsa-miR-4792_1ss9GT	GO:0005737
ENSSSCG00000005838	TRAF2	hsa-miR-4792_1ss9GT	GO:0005737
ENSSSCG00000025374	DVL3	hsa-miR-4792_1ss9GT	GO:0005737
ENSSSCG00000027668	NLK	hsa-miR-4792_1ss9GT	GO:0005737
ENSSSCG00000011101	ITGB1	hsa-miR-4792_1ss9GT	GO:0005737
ENSSSCG00000004507	SMAD7	hsa-miR-4792_1ss9GT	GO:0005737
ENSSSCG00000010132	COMT	hsa-miR-4792_1ss9GT	GO:0005739
ENSSSCG00000001518	ITPR3	hsa-miR-4792_1ss9GT	GO:0005739
ENSSSCG00000004507	SMAD7	hsa-miR-4792_1ss9GT	GO:0005829
ENSSSCG00000005838	TRAF2	hsa-miR-4792_1ss9GT	GO:0005829
ENSSSCG00000009855	KSR2	hsa-miR-4792_1ss9GT	GO:0005829
ENSSSCG00000012399	FOXO4	hsa-miR-4792_1ss9GT	GO:0005829
ENSSSCG00000025374	DVL3	hsa-miR-4792_1ss9GT	GO:0005829
ENSSSCG00000007520	GNAS	hsa-miR-4792_1ss9GT	GO:0005829
ENSSSCG00000007673	NR5A1	hsa-miR-4792_1ss9GT	GO:0006915
ENSSSCG00000004507	SMAD7	hsa-miR-4792_1ss9GT	GO:0007179

ENSSSCG00000027668	NLK	hsa-miR-4792_1ss9GT	GO:0007179
ENSSSCG00000002960	RASGRP4	hsa-miR-4792_1ss9GT	GO:0007264
ENSSSCG00000007673	NR5A1	hsa-miR-4792_1ss9GT	GO:0008284
ENSSSCG00000008842	KIT	hsa-miR-4792_1ss9GT	GO:0008284
ENSSSCG00000011101	ITGB1	hsa-miR-4792_1ss9GT	GO:0008284
ENSSSCG00000017306	ITGB3	hsa-miR-4792_1ss9GT	GO:0009897
ENSSSCG00000017306	ITGB3	hsa-miR-4792_1ss9GT	GO:0009897
ENSSSCG00000017357	ITGA2B	hsa-miR-4792_1ss9GT	GO:0009897
ENSSSCG00000001898	ULK3	hsa-miR-4792_1ss9GT	GO:0009897
ENSSSCG00000011101	ITGB1	hsa-miR-4792_1ss9GT	GO:0016477
ENSSSCG00000012399	FOXO4	hsa-miR-4792_1ss9GT	GO:0016607
ENSSSCG00000013564	INSR	hsa-miR-4792_1ss9GT	GO:0016740
ENSSSCG00000012495	BTK	hsa-miR-4792_1ss9GT	GO:0016740
ENSSSCG00000012495	BTK	hsa-miR-4792_1ss9GT	GO:0016740
ENSSSCG00000012495	BTK	hsa-miR-4792_1ss9GT	GO:0016740
ENSSSCG00000012495	BTK	hsa-miR-4792_1ss9GT	GO:0016740
ENSSSCG00000004022	RPS6KA2	hsa-miR-4792_1ss9GT	GO:0016740
ENSSSCG00000006927	PKN2	hsa-miR-4792_1ss9GT	GO:0016740
ENSSSCG00000008842	KIT	hsa-miR-4792_1ss9GT	GO:0016740
ENSSSCG00000027668	NLK	hsa-miR-4792_1ss9GT	GO:0016740
ENSSSCG00000004022	RPS6KA2	hsa-miR-4792_1ss9GT	GO:0035556
ENSSSCG00000006927	PKN2	hsa-miR-4792_1ss9GT	GO:0035556
ENSSSCG00000027668	NLK	hsa-miR-4792_1ss9GT	GO:0035556
ENSSSCG00000012495	BTK	hsa-miR-4792_1ss9GT	GO:0035556
ENSSSCG00000012495	BTK	hsa-miR-4792_1ss9GT	GO:0035556
ENSSSCG00000012495	BTK	hsa-miR-4792_1ss9GT	GO:0035556
ENSSSCG00000012495	BTK	hsa-miR-4792_1ss9GT	GO:0035556
ENSSSCG00000009855	KSR2	hsa-miR-4792_1ss9GT	GO:0035556
ENSSSCG00000025374	DVL3	hsa-miR-4792_1ss9GT	GO:0035556
ENSSSCG00000002960	RASGRP4	hsa-miR-4792_1ss9GT	GO:0035556
ENSSSCG00000008842	KIT	hsa-miR-4792_1ss9GT	GO:0035556
ENSSSCG00000017306	ITGB3	hsa-miR-4792_1ss9GT	GO:0042802
ENSSSCG00000017306	ITGB3	hsa-miR-4792_1ss9GT	GO:0042802
ENSSSCG00000017357	ITGA2B	hsa-miR-4792_1ss9GT	GO:0042802
ENSSSCG00000005838	TRAF2	hsa-miR-4792_1ss9GT	GO:0042802
ENSSSCG00000022689	GADD45B	hsa-miR-4792_1ss9GT	GO:0043065
ENSSSCG00000011101	ITGB1	hsa-miR-4792_1ss9GT	GO:0043065
ENSSSCG00000005838	TRAF2	hsa-miR-4792_1ss9GT	GO:0043123
ENSSSCG00000008162	IL1R1	hsa-miR-4792_1ss9GT	GO:0043231
ENSSSCG00000016140	FZD5	hsa-miR-4792_1ss9GT	GO:0045944
ENSSSCG00000004022	RPS6KA2	hsa-miR-4792_1ss9GT	GO:0045944
ENSSSCG00000007673	NR5A1	hsa-miR-4792_1ss9GT	GO:0046579
ENSSSCG00000002960	RASGRP4	hsa-miR-4792_1ss9GT	GO:0046579
ENSSSCG00000016140	FZD5	hsa-miR-4792_1ss9GT	GO:0048471

ENSSSCG00000004507	SMAD7	hsa-miR-4792_1ss9GT	GO:0048471
ENSSSCG00000005838	TRAF2	hsa-miR-4792_1ss9GT	GO:0048471
ENSSSCG00000007520	GNAS	hsa-miR-4792_1ss9GT	GO:0070062
ENSSSCG00000010132	COMT	hsa-miR-4792_1ss9GT	GO:0070062
ENSSSCG00000017357	ITGA2B	hsa-miR-4792_1ss9GT	GO:0070062
ENSSSCG00000011101	ITGB1	hsa-miR-4792_1ss9GT	GO:0070062
ENSSSCG00000004507	SMAD7	hsa-miR-4792_1ss9GT	GO:0071560
ENSSSCG00000001404	NR5A1	PC-5p-9551_196	GO:0000187
ENSSSCG00000004789	THBS1	PC-5p-9551_196	GO:0000187
ENSSSCG00000006539	SHC1	PC-5p-9551_196	GO:0000187
ENSSSCG00000006539	SHC1	PC-5p-9551_196	GO:0000187
ENSSSCG00000017330	MAP3K14	PC-5p-9551_196	GO:0001650
ENSSSCG00000000175	ENSSSCG00000000175	PC-5p-9551_196	GO:0003723
ENSSSCG00000000271	AMHR2	PC-5p-9551_196	GO:0003723
ENSSSCG00000005382	TGFBR1	PC-5p-9551_196	GO:0005025
ENSSSCG00000002804	CSNK2A2	PC-5p-9551_196	GO:0005634
ENSSSCG00000009840	PRKAB1	PC-5p-9551_196	GO:0005634
ENSSSCG00000001661	SRF	PC-5p-9551_196	GO:0005634
ENSSSCG00000009040	SMAD1	PC-5p-9551_196	GO:0005634
ENSSSCG00000025768	CALM1	PC-5p-9551_196	GO:0005634
ENSSSCG00000022545	CALM3	PC-5p-9551_196	GO:0005634
ENSSSCG00000004154	TNFAIP3	PC-5p-9551_196	GO:0005634
ENSSSCG00000008040	TSC2	PC-5p-9551_196	GO:0005634
ENSSSCG00000011415	MAPKAPK3	PC-5p-9551_196	GO:0005634
ENSSSCG00000004154	TNFAIP3	PC-5p-9551_196	GO:0005634
ENSSSCG00000003928	PLK3	PC-5p-9551_196	GO:0005634
ENSSSCG00000009228	MAPK10	PC-5p-9551_196	GO:0005634
ENSSSCG00000009370	FOXO1	PC-5p-9551_196	GO:0005634
ENSSSCG00000020744	DUSP3	PC-5p-9551_196	GO:0005634
ENSSSCG00000009370	FOXO1	PC-5p-9551_196	GO:0005634
ENSSSCG00000009228	MAPK10	PC-5p-9551_196	GO:0005634
ENSSSCG00000000293	ITGA5	PC-5p-9551_196	GO:0005634
ENSSSCG00000001404	NR5A1	PC-5p-9551_196	GO:0005634
ENSSSCG00000009228	MAPK10	PC-5p-9551_196	GO:0005654
ENSSSCG00000011415	MAPKAPK3	PC-5p-9551_196	GO:0005654
ENSSSCG00000020744	DUSP3	PC-5p-9551_196	GO:0005654
ENSSSCG00000009228	MAPK10	PC-5p-9551_196	GO:0005654
ENSSSCG00000015383	RAPGEF5	PC-5p-9551_196	GO:0005654
ENSSSCG00000012913	PPP1CA	PC-5p-9551_196	GO:0005654
ENSSSCG00000027426	NR5A1	PC-5p-9551_196	GO:0005654
ENSSSCG00000030396	SETD7	PC-5p-9551_196	GO:0005730
ENSSSCG00000015815	NR5A1	PC-5p-9551_196	GO:0005730
ENSSSCG00000001661	SRF	PC-5p-9551_196	GO:0005737
ENSSSCG00000003154	GYS1	PC-5p-9551_196	GO:0005737

ENSSSCG00000004154	TNFAIP3	PC-5p-9551_196	GO:0005737
ENSSSCG00000008164	MAP4K4	PC-5p-9551_196	GO:0005737
ENSSSCG00000009040	SMAD1	PC-5p-9551_196	GO:0005737
ENSSSCG00000009228	MAPK10	PC-5p-9551_196	GO:0005737
ENSSSCG00000011415	MAPKAPK3	PC-5p-9551_196	GO:0005737
ENSSSCG00000014146	RASA1	PC-5p-9551_196	GO:0005737
ENSSSCG00000014220	ATG12	PC-5p-9551_196	GO:0005737
ENSSSCG00000017562	CACNA1G	PC-5p-9551_196	GO:0005737
ENSSSCG00000030153	SMURF1	PC-5p-9551_196	GO:0005737
ENSSSCG00000004154	TNFAIP3	PC-5p-9551_196	GO:0005737
ENSSSCG00000009228	MAPK10	PC-5p-9551_196	GO:0005737
ENSSSCG00000003928	PLK3	PC-5p-9551_196	GO:0005737
ENSSSCG00000008040	TSC2	PC-5p-9551_196	GO:0005737
ENSSSCG00000008164	MAP4K4	PC-5p-9551_196	GO:0005737
ENSSSCG00000009567	RASA3	PC-5p-9551_196	GO:0005737
ENSSSCG00000013020	MAP4K2	PC-5p-9551_196	GO:0005737
ENSSSCG00000016578	FLNC	PC-5p-9551_196	GO:0005737
ENSSSCG00000009370	FOXO1	PC-5p-9551_196	GO:0005737
ENSSSCG00000009874	NR5A1	PC-5p-9551_196	GO:0005737
ENSSSCG00000011101	ITGB1	PC-5p-9551_196	GO:0005737
ENSSSCG00000012913	PPP1CA	PC-5p-9551_196	GO:0005737
ENSSSCG00000017330	MAP3K14	PC-5p-9551_196	GO:0005737
ENSSSCG00000025374	DVL3	PC-5p-9551_196	GO:0005737
ENSSSCG00000024813	MAP3K3	PC-5p-9551_196	GO:0005737
ENSSSCG00000027426	NR5A1	PC-5p-9551_196	GO:0005737
ENSSSCG00000011101	ITGB1	PC-5p-9551_196	GO:0005737
ENSSSCG00000009370	FOXO1	PC-5p-9551_196	GO:0005737
ENSSSCG00000011101	ITGB1	PC-5p-9551_196	GO:0005737
ENSSSCG00000012657	AIFM1	PC-5p-9551_196	GO:0005737
ENSSSCG00000016628	WNT2	PC-5p-9551_196	GO:0005737
ENSSSCG00000005661	NR5A1	PC-5p-9551_196	GO:0005739
ENSSSCG00000030153	SMURF1	PC-5p-9551_196	GO:0005739
ENSSSCG00000012275	ARAF	PC-5p-9551_196	GO:0005739
ENSSSCG00000012657	AIFM1	PC-5p-9551_196	GO:0005739
ENSSSCG00000002707	GABARAPL2	PC-5p-9551_196	GO:0005776
ENSSSCG00000008040	TSC2	PC-5p-9551_196	GO:0005829
ENSSSCG00000009874	NR5A1	PC-5p-9551_196	GO:0005829
ENSSSCG00000012657	AIFM1	PC-5p-9551_196	GO:0005829
ENSSSCG00000012913	PPP1CA	PC-5p-9551_196	GO:0005829
ENSSSCG00000016578	FLNC	PC-5p-9551_196	GO:0005829
ENSSSCG00000017330	MAP3K14	PC-5p-9551_196	GO:0005829
ENSSSCG00000020744	DUSP3	PC-5p-9551_196	GO:0005829
ENSSSCG00000025374	DVL3	PC-5p-9551_196	GO:0005829
ENSSSCG00000027426	NR5A1	PC-5p-9551_196	GO:0005829

ENSSSCG00000012275	ARAF	PC-5p-9551_196	GO:0005829
ENSSSCG00000002989	AKT2	PC-5p-9551_196	GO:0005829
ENSSSCG00000012657	AIFM1	PC-5p-9551_196	GO:0006915
ENSSSCG00000003928	PLK3	PC-5p-9551_196	GO:0006974
ENSSSCG00000030396	SETD7	PC-5p-9551_196	GO:0006974
ENSSSCG00000009370	FOXO1	PC-5p-9551_196	GO:0006974
ENSSSCG00000027426	NR5A1	PC-5p-9551_196	GO:0006974
ENSSSCG00000009370	FOXO1	PC-5p-9551_196	GO:0006974
ENSSSCG00000005382	TGFBR1	PC-5p-9551_196	GO:0007179
ENSSSCG00000015383	RAPGEF5	PC-5p-9551_196	GO:0007264
ENSSSCG00000029852	WNT5A	PC-5p-9551_196	GO:0008284
ENSSSCG00000015815	NR5A1	PC-5p-9551_196	GO:0008284
ENSSSCG00000008844	KDR	PC-5p-9551_196	GO:0008284
ENSSSCG00000004789	THBS1	PC-5p-9551_196	GO:0008284
ENSSSCG00000011101	ITGB1	PC-5p-9551_196	GO:0008284
ENSSSCG00000016628	WNT2	PC-5p-9551_196	GO:0008284
ENSSSCG00000011101	ITGB1	PC-5p-9551_196	GO:0008284
ENSSSCG00000011101	ITGB1	PC-5p-9551_196	GO:0008284
ENSSSCG00000000293	ITGA5	PC-5p-9551_196	GO:0009897
ENSSSCG00000017578	ITGA3	PC-5p-9551_196	GO:0009897
ENSSSCG00000017578	ITGA3	PC-5p-9551_196	GO:0009897
ENSSSCG00000001661	SRF	PC-5p-9551_196	GO:0009897
ENSSSCG00000004789	THBS1	PC-5p-9551_196	GO:0009897
ENSSSCG00000012657	AIFM1	PC-5p-9551_196	GO:0010942
ENSSSCG00000029852	WNT5A	PC-5p-9551_196	GO:0016477
ENSSSCG00000002707	GABARAPL2	PC-5p-9551_196	GO:0016477
ENSSSCG00000002804	CSNK2A2	PC-5p-9551_196	GO:0016477
ENSSSCG00000003154	GYS1	PC-5p-9551_196	GO:0016477
ENSSSCG00000011101	ITGB1	PC-5p-9551_196	GO:0016477
ENSSSCG00000011101	ITGB1	PC-5p-9551_196	GO:0016477
ENSSSCG00000011101	ITGB1	PC-5p-9551_196	GO:0016477
ENSSSCG00000000271	AMHR2	PC-5p-9551_196	GO:0016740
ENSSSCG00000003154	GYS1	PC-5p-9551_196	GO:0016740
ENSSSCG00000030396	SETD7	PC-5p-9551_196	GO:0016740
ENSSSCG00000015815	NR5A1	PC-5p-9551_196	GO:0016740
ENSSSCG00000010872	AKT3	PC-5p-9551_196	GO:0016740
ENSSSCG00000008844	KDR	PC-5p-9551_196	GO:0016740
ENSSSCG00000003928	PLK3	PC-5p-9551_196	GO:0016740
ENSSSCG00000009228	MAPK10	PC-5p-9551_196	GO:0016740
ENSSSCG00000013020	MAP4K2	PC-5p-9551_196	GO:0016740
ENSSSCG00000030153	SMURF1	PC-5p-9551_196	GO:0016740
ENSSSCG00000012275	ARAF	PC-5p-9551_196	GO:0016740
ENSSSCG00000002989	AKT2	PC-5p-9551_196	GO:0016740
ENSSSCG00000009228	MAPK10	PC-5p-9551_196	GO:0016740

ENSSSCG00000005382	TGFBR1	PC-5p-9551_196	GO:0016740
ENSSSCG00000009567	RASA3	PC-5p-9551_196	GO:0035556
ENSSSCG00000013020	MAP4K2	PC-5p-9551_196	GO:0035556
ENSSSCG00000024813	MAP3K3	PC-5p-9551_196	GO:0035556
ENSSSCG00000027952	ADCY5	PC-5p-9551_196	GO:0035556
ENSSSCG00000005382	TGFBR1	PC-5p-9551_196	GO:0035556
ENSSSCG00000010872	AKT3	PC-5p-9551_196	GO:0035556
ENSSSCG00000006539	SHC1	PC-5p-9551_196	GO:0035556
ENSSSCG00000009874	NR5A1	PC-5p-9551_196	GO:0035556
ENSSSCG00000015383	RAPGEF5	PC-5p-9551_196	GO:0035556
ENSSSCG00000025374	DVL3	PC-5p-9551_196	GO:0035556
ENSSSCG00000006539	SHC1	PC-5p-9551_196	GO:0035556
ENSSSCG00000012275	ARAF	PC-5p-9551_196	GO:0035556
ENSSSCG00000002989	AKT2	PC-5p-9551_196	GO:0035556
ENSSSCG00000004154	TNFAIP3	PC-5p-9551_196	GO:0042802
ENSSSCG00000009040	SMAD1	PC-5p-9551_196	GO:0042802
ENSSSCG00000015815	NR5A1	PC-5p-9551_196	GO:0042802
ENSSSCG00000004154	TNFAIP3	PC-5p-9551_196	GO:0042802
ENSSSCG00000005661	NR5A1	PC-5p-9551_196	GO:0043065
ENSSSCG00000009370	FOXO1	PC-5p-9551_196	GO:0043065
ENSSSCG00000011101	ITGB1	PC-5p-9551_196	GO:0043065
ENSSSCG00000011101	ITGB1	PC-5p-9551_196	GO:0043065
ENSSSCG00000009370	FOXO1	PC-5p-9551_196	GO:0043065
ENSSSCG00000011101	ITGB1	PC-5p-9551_196	GO:0043065
ENSSSCG00000001404	NR5A1	PC-5p-9551_196	GO:0043065
ENSSSCG00000014216	TICAM2	PC-5p-9551_196	GO:0043123
ENSSSCG00000017330	MAP3K14	PC-5p-9551_196	GO:0043123
ENSSSCG00000024813	MAP3K3	PC-5p-9551_196	GO:0043123
ENSSSCG00000001404	NR5A1	PC-5p-9551_196	GO:0043123
ENSSSCG00000006780	WNT2B	PC-5p-9551_196	GO:0043231
ENSSSCG00000017330	MAP3K14	PC-5p-9551_196	GO:0043231
ENSSSCG00000002989	AKT2	PC-5p-9551_196	GO:0043231
ENSSSCG00000003928	PLK3	PC-5p-9551_196	GO:0043231
ENSSSCG00000001661	SRF	PC-5p-9551_196	GO:0045944
ENSSSCG00000009040	SMAD1	PC-5p-9551_196	GO:0045944
ENSSSCG00000029852	WNT5A	PC-5p-9551_196	GO:0045944
ENSSSCG00000004154	TNFAIP3	PC-5p-9551_196	GO:0045944
ENSSSCG00000001404	NR5A1	PC-5p-9551_196	GO:0045944
ENSSSCG00000027426	NR5A1	PC-5p-9551_196	GO:0045944
ENSSSCG00000008040	TSC2	PC-5p-9551_196	GO:0048471
ENSSSCG00000004789	THBS1	PC-5p-9551_196	GO:0048471
ENSSSCG00000005661	NR5A1	PC-5p-9551_196	GO:0048471
ENSSSCG00000004154	TNFAIP3	PC-5p-9551_196	GO:0070062
ENSSSCG00000030153	SMURF1	PC-5p-9551_196	GO:0070062

ENSSSCG00000004154	TNFAIP3	PC-5p-9551_196	GO:0070062
ENSSSCG00000017578	ITGA3	PC-5p-9551_196	GO:0070062
ENSSSCG00000025768	CALM1	PC-5p-9551_196	GO:0070062
ENSSSCG00000022545	CALM3	PC-5p-9551_196	GO:0070062
ENSSSCG00000017578	ITGA3	PC-5p-9551_196	GO:0070062
ENSSSCG00000020744	DUSP3	PC-5p-9551_196	GO:0070062
ENSSSCG00000004789	THBS1	PC-5p-9551_196	GO:0070062
ENSSSCG00000011101	ITGB1	PC-5p-9551_196	GO:0070062
ENSSSCG00000011101	ITGB1	PC-5p-9551_196	GO:0070062
ENSSSCG00000011101	ITGB1	PC-5p-9551_196	GO:0070062
ENSSSCG00000029852	WNT5A	PC-5p-9551_196	GO:0071560
ENSSSCG00000016628	WNT2	PC-5p-9551_196	GO:0071560
ENSSSCG00000006780	WNT2B	PC-5p-9551_196	GO:0090263
ENSSSCG00000016628	WNT2	PC-5p-9551_196	GO:0090263
ENSSSCG00000001404	NR5A1	PC-5p-9551_196	GO:0097190
ENSSSCG00000004789	THBS1	PC-5p-9551_196	GO:1902043
ENSSSCG00000012913	PPP1CA	PC-5p-9551_196	GO:2001241
ENSSSCG00000001404	NR5A1	sha-mir-24-1-p3_1ss2GC	GO:0000187
ENSSSCG00000000233	ACVR1B	sha-mir-24-1-p3_1ss2GC	GO:0003723
ENSSSCG00000001404	NR5A1	sha-mir-24-1-p3_1ss2GC	GO:0005654
ENSSSCG00000000233	ACVR1B	sha-mir-24-1-p3_1ss2GC	GO:0005829
ENSSSCG00000017412	RAB5C	sha-mir-24-1-p3_1ss2GC	GO:0007264
ENSSSCG00000000233	ACVR1B	sha-mir-24-1-p3_1ss2GC	GO:0016740
ENSSSCG00000001404	NR5A1	sha-mir-24-1-p3_1ss2GC	GO:0043065
ENSSSCG00000001404	NR5A1	sha-mir-24-1-p3_1ss2GC	GO:0043123
ENSSSCG00000001404	NR5A1	sha-mir-24-1-p3_1ss2GC	GO:0045944
ENSSSCG00000000233	ACVR1B	sha-mir-24-1-p3_1ss2GC	GO:0070062
ENSSSCG00000001404	NR5A1	sha-mir-24-1-p3_1ss2GC	GO:0097190
ENSSSCG00000020672	F2R	ssc-miR-139-3p	GO:0000187
ENSSSCG00000006145	E2F5	ssc-miR-139-3p	GO:0001650
ENSSSCG00000006145	E2F5	ssc-miR-139-3p	GO:0005634
ENSSSCG00000016991	DUSP1	ssc-miR-139-3p	GO:0005634
ENSSSCG00000021560	UBE2I	ssc-miR-139-3p	GO:0005634
ENSSSCG00000015383	RAPGEF5	ssc-miR-139-3p	GO:0005654
ENSSSCG00000012913	PPP1CA	ssc-miR-139-3p	GO:0005654
ENSSSCG00000006145	E2F5	ssc-miR-139-3p	GO:0005737
ENSSSCG00000008845	SRD5A3	ssc-miR-139-3p	GO:0005737
ENSSSCG00000018016	MAP2K4	ssc-miR-139-3p	GO:0005737
ENSSSCG00000008845	SRD5A3	ssc-miR-139-3p	GO:0005737
ENSSSCG00000008845	SRD5A3	ssc-miR-139-3p	GO:0005737
ENSSSCG00000016991	DUSP1	ssc-miR-139-3p	GO:0005737
ENSSSCG00000016991	DUSP1	ssc-miR-139-3p	GO:0005737
ENSSSCG00000012913	PPP1CA	ssc-miR-139-3p	GO:0005737
ENSSSCG00000021560	UBE2I	ssc-miR-139-3p	GO:0005737

ENSSSCG00000024388	BNIP3	ssc-miR-139-3p	GO:0005739
ENSSSCG00000010448	FAS	ssc-miR-139-3p	GO:0005829
ENSSSCG00000012913	PPP1CA	ssc-miR-139-3p	GO:0005829
ENSSSCG00000012034	TIAM1	ssc-miR-139-3p	GO:0005829
ENSSSCG00000021560	UBE2I	ssc-miR-139-3p	GO:0005829
ENSSSCG00000024388	BNIP3	ssc-miR-139-3p	GO:0006915
ENSSSCG00000010448	FAS	ssc-miR-139-3p	GO:0006915
ENSSSCG00000015383	RAPGEF5	ssc-miR-139-3p	GO:0007264
ENSSSCG00000012034	TIAM1	ssc-miR-139-3p	GO:0008284
ENSSSCG00000020672	F2R	ssc-miR-139-3p	GO:0008284
ENSSSCG00000030172	CD40LG	ssc-miR-139-3p	GO:0009897
ENSSSCG00000016887	ITGA2	ssc-miR-139-3p	GO:0009897
ENSSSCG00000010448	FAS	ssc-miR-139-3p	GO:0009897
ENSSSCG00000005751	COL5A1	ssc-miR-139-3p	GO:0016477
ENSSSCG00000012495	BTK	ssc-miR-139-3p	GO:0016740
ENSSSCG00000012495	BTK	ssc-miR-139-3p	GO:0016740
ENSSSCG00000012495	BTK	ssc-miR-139-3p	GO:0016740
ENSSSCG00000012495	BTK	ssc-miR-139-3p	GO:0035556
ENSSSCG00000012495	BTK	ssc-miR-139-3p	GO:0035556
ENSSSCG00000012495	BTK	ssc-miR-139-3p	GO:0035556
ENSSSCG00000015383	RAPGEF5	ssc-miR-139-3p	GO:0035556
ENSSSCG00000012034	TIAM1	ssc-miR-139-3p	GO:0035556
ENSSSCG00000024388	BNIP3	ssc-miR-139-3p	GO:0042802
ENSSSCG00000016991	DUSP1	ssc-miR-139-3p	GO:0042802
ENSSSCG00000024388	BNIP3	ssc-miR-139-3p	GO:0043065
ENSSSCG00000010448	FAS	ssc-miR-139-3p	GO:0043065
ENSSSCG00000021068	TRAF5	ssc-miR-139-3p	GO:0043123
ENSSSCG00000021068	TRAF5	ssc-miR-139-3p	GO:0043123
ENSSSCG00000021560	UBE2I	ssc-miR-139-3p	GO:0043123
ENSSSCG00000020672	F2R	ssc-miR-139-3p	GO:0043123
ENSSSCG00000005751	COL5A1	ssc-miR-139-3p	GO:0048471
ENSSSCG00000005751	COL5A1	ssc-miR-139-3p	GO:0070062
ENSSSCG00000006145	E2F5	ssc-miR-139-3p	GO:2001237
ENSSSCG00000012913	PPP1CA	ssc-miR-139-3p	GO:2001241
ENSSSCG00000010448	FAS	ssc-miR-139-3p	GO:2001241
ENSSSCG00000006530	EFNA1	ssc-miR-1839-3p_R+2	GO:0000187
ENSSSCG00000010894	TP53BP2	ssc-miR-1839-3p_R+2	GO:0005634
ENSSSCG00000025768	CALM1	ssc-miR-1839-3p_R+2	GO:0005634
ENSSSCG00000022482	DDIT3	ssc-miR-1839-3p_R+2	GO:0005634
ENSSSCG00000028968	RAC1	ssc-miR-1839-3p_R+2	GO:0005634
ENSSSCG00000000529	DNM1L	ssc-miR-1839-3p_R+2	GO:0005634
ENSSSCG00000030396	SETD7	ssc-miR-1839-3p_R+2	GO:0005730
ENSSSCG00000008164	MAP4K4	ssc-miR-1839-3p_R+2	GO:0005737
ENSSSCG00000010894	TP53BP2	ssc-miR-1839-3p_R+2	GO:0005737

ENSSSCG00000014146	RASA1	ssc-miR-1839-3p_R+2	GO:0005737
ENSSSCG00000003520	CDC42	ssc-miR-1839-3p_R+2	GO:0005737
ENSSSCG00000010509	PIK3AP1	ssc-miR-1839-3p_R+2	GO:0005737
ENSSSCG00000028968	RAC1	ssc-miR-1839-3p_R+2	GO:0005737
ENSSSCG00000022482	DDIT3	ssc-miR-1839-3p_R+2	GO:0005737
ENSSSCG00000000529	DNM1L	ssc-miR-1839-3p_R+2	GO:0005737
ENSSSCG00000000529	DNM1L	ssc-miR-1839-3p_R+2	GO:0005737
ENSSSCG00000003520	CDC42	ssc-miR-1839-3p_R+2	GO:0005737
ENSSSCG00000000529	DNM1L	ssc-miR-1839-3p_R+2	GO:0005739
ENSSSCG00000000529	DNM1L	ssc-miR-1839-3p_R+2	GO:0005739
ENSSSCG00000025455	RALB	ssc-miR-1839-3p_R+2	GO:0005776
ENSSSCG00000000529	DNM1L	ssc-miR-1839-3p_R+2	GO:0005829
ENSSSCG00000004110	TAB2	ssc-miR-1839-3p_R+2	GO:0005829
ENSSSCG00000010509	PIK3AP1	ssc-miR-1839-3p_R+2	GO:0005829
ENSSSCG00000000529	DNM1L	ssc-miR-1839-3p_R+2	GO:0005829
ENSSSCG00000003520	CDC42	ssc-miR-1839-3p_R+2	GO:0005829
ENSSSCG00000016027	ITGAV	ssc-miR-1839-3p_R+2	GO:0005829
ENSSSCG00000022482	DDIT3	ssc-miR-1839-3p_R+2	GO:0006915
ENSSSCG00000030396	SETD7	ssc-miR-1839-3p_R+2	GO:0006974
ENSSSCG00000003520	CDC42	ssc-miR-1839-3p_R+2	GO:0007264
ENSSSCG00000028968	RAC1	ssc-miR-1839-3p_R+2	GO:0007264
ENSSSCG00000025455	RALB	ssc-miR-1839-3p_R+2	GO:0007264
ENSSSCG00000003520	CDC42	ssc-miR-1839-3p_R+2	GO:0007264
ENSSSCG00000014362	HBEGF	ssc-miR-1839-3p_R+2	GO:0008284
ENSSSCG00000016027	ITGAV	ssc-miR-1839-3p_R+2	GO:0008284
ENSSSCG00000016027	ITGAV	ssc-miR-1839-3p_R+2	GO:0009897
ENSSSCG00000014362	HBEGF	ssc-miR-1839-3p_R+2	GO:0016477
ENSSSCG00000006530	EFNA1	ssc-miR-1839-3p_R+2	GO:0016477
ENSSSCG00000003520	CDC42	ssc-miR-1839-3p_R+2	GO:0016477
ENSSSCG00000030396	SETD7	ssc-miR-1839-3p_R+2	GO:0016740
ENSSSCG00000003520	CDC42	ssc-miR-1839-3p_R+2	GO:0042802
ENSSSCG00000010894	TP53BP2	ssc-miR-1839-3p_R+2	GO:0042802
ENSSSCG00000000529	DNM1L	ssc-miR-1839-3p_R+2	GO:0043065
ENSSSCG00000000529	DNM1L	ssc-miR-1839-3p_R+2	GO:0043065
ENSSSCG00000004110	TAB2	ssc-miR-1839-3p_R+2	GO:0043123
ENSSSCG00000006780	WNT2B	ssc-miR-1839-3p_R+2	GO:0043231
ENSSSCG00000000529	DNM1L	ssc-miR-1839-3p_R+2	GO:0043231
ENSSSCG00000000529	DNM1L	ssc-miR-1839-3p_R+2	GO:0043231
ENSSSCG00000022482	DDIT3	ssc-miR-1839-3p_R+2	GO:0045944
ENSSSCG00000004110	TAB2	ssc-miR-1839-3p_R+2	GO:0045944
ENSSSCG00000010894	TP53BP2	ssc-miR-1839-3p_R+2	GO:0048471
ENSSSCG00000006530	EFNA1	ssc-miR-1839-3p_R+2	GO:0070062
ENSSSCG00000025768	CALM1	ssc-miR-1839-3p_R+2	GO:0070062
ENSSSCG00000003520	CDC42	ssc-miR-1839-3p_R+2	GO:0070062

ENSSSCG00000028968	RAC1	ssc-miR-1839-3p_R+2	GO:0070062
ENSSSCG00000025455	RALB	ssc-miR-1839-3p_R+2	GO:0070062
ENSSSCG00000003520	CDC42	ssc-miR-1839-3p_R+2	GO:0070062
ENSSSCG00000016027	ITGAV	ssc-miR-1839-3p_R+2	GO:0070062
ENSSSCG00000006780	WNT2B	ssc-miR-1839-3p_R+2	GO:0090263
ENSSSCG00000016027	ITGAV	ssc-miR-1839-3p_R+2	GO:2001237
ENSSSCG00000015403	HGF	ssc-miR-190b	GO:0000187
ENSSSCG00000016878	FGF10	ssc-miR-190b	GO:0000187
ENSSSCG00000025777	ESR1	ssc-miR-190b	GO:0005634
ENSSSCG00000025777	ESR1	ssc-miR-190b	GO:0005634
ENSSSCG00000004154	TNFAIP3	ssc-miR-190b	GO:0005634
ENSSSCG00000004154	TNFAIP3	ssc-miR-190b	GO:0005634
ENSSSCG00000016878	FGF10	ssc-miR-190b	GO:0005634
ENSSSCG00000000529	DNM1L	ssc-miR-190b	GO:0005634
ENSSSCG00000017670	RPS6KB1	ssc-miR-190b	GO:0005654
ENSSSCG00000004154	TNFAIP3	ssc-miR-190b	GO:0005737
ENSSSCG00000004896	PHLPP1	ssc-miR-190b	GO:0005737
ENSSSCG00000004154	TNFAIP3	ssc-miR-190b	GO:0005737
ENSSSCG00000000529	DNM1L	ssc-miR-190b	GO:0005737
ENSSSCG00000007356	PLCG1	ssc-miR-190b	GO:0005737
ENSSSCG00000012163	RPS6KA3	ssc-miR-190b	GO:0005737
ENSSSCG00000017670	RPS6KB1	ssc-miR-190b	GO:0005737
ENSSSCG00000000529	DNM1L	ssc-miR-190b	GO:0005737
ENSSSCG00000000529	DNM1L	ssc-miR-190b	GO:0005739
ENSSSCG00000017670	RPS6KB1	ssc-miR-190b	GO:0005739
ENSSSCG00000000529	DNM1L	ssc-miR-190b	GO:0005739
ENSSSCG00000000529	DNM1L	ssc-miR-190b	GO:0005829
ENSSSCG00000007356	PLCG1	ssc-miR-190b	GO:0005829
ENSSSCG00000000529	DNM1L	ssc-miR-190b	GO:0005829
ENSSSCG00000012163	RPS6KA3	ssc-miR-190b	GO:0006915
ENSSSCG00000015770	VEGFC	ssc-miR-190b	GO:0008284
ENSSSCG00000015403	HGF	ssc-miR-190b	GO:0008284
ENSSSCG00000016878	FGF10	ssc-miR-190b	GO:0008284
ENSSSCG00000007356	PLCG1	ssc-miR-190b	GO:0016477
ENSSSCG00000012163	RPS6KA3	ssc-miR-190b	GO:0016740
ENSSSCG00000012163	RPS6KA3	ssc-miR-190b	GO:0035556
ENSSSCG00000017670	RPS6KB1	ssc-miR-190b	GO:0035556
ENSSSCG00000007356	PLCG1	ssc-miR-190b	GO:0035556
ENSSSCG00000004154	TNFAIP3	ssc-miR-190b	GO:0042802
ENSSSCG00000004154	TNFAIP3	ssc-miR-190b	GO:0042802
ENSSSCG00000020906	TNFSF10	ssc-miR-190b	GO:0043065
ENSSSCG00000000529	DNM1L	ssc-miR-190b	GO:0043065
ENSSSCG00000000529	DNM1L	ssc-miR-190b	GO:0043065
ENSSSCG00000020906	TNFSF10	ssc-miR-190b	GO:0043123

ENSSSCG00000006780	WNT2B	ssc-miR-190b	GO:0043231
ENSSSCG00000000529	DNM1L	ssc-miR-190b	GO:0043231
ENSSSCG00000000529	DNM1L	ssc-miR-190b	GO:0043231
ENSSSCG00000004154	TNFAIP3	ssc-miR-190b	GO:0045944
ENSSSCG00000012163	RPS6KA3	ssc-miR-190b	GO:0045944
ENSSSCG00000016878	FGF10	ssc-miR-190b	GO:0046579
ENSSSCG00000004896	PHLPP1	ssc-miR-190b	GO:0048471
ENSSSCG00000004154	TNFAIP3	ssc-miR-190b	GO:0070062
ENSSSCG00000004154	TNFAIP3	ssc-miR-190b	GO:0070062
ENSSSCG00000020906	TNFSF10	ssc-miR-190b	GO:0070062
ENSSSCG00000006780	WNT2B	ssc-miR-190b	GO:0090263
ENSSSCG00000017670	RPS6KB1	ssc-miR-190b	GO:2001237
ENSSSCG00000009051	IL15	ssc-miR-192	GO:0005654
ENSSSCG00000003194	NR5A1	ssc-miR-192	GO:0005737
ENSSSCG00000009051	IL15	ssc-miR-192	GO:0005737
ENSSSCG00000016918	MAP3K1	ssc-miR-192	GO:0005737
ENSSSCG00000028420	EIF4E	ssc-miR-192	GO:0005737
ENSSSCG00000016628	WNT2	ssc-miR-192	GO:0005737
ENSSSCG00000003194	NR5A1	ssc-miR-192	GO:0005829
ENSSSCG00000009051	IL15	ssc-miR-192	GO:0005829
ENSSSCG00000028420	EIF4E	ssc-miR-192	GO:0005829
ENSSSCG00000016918	MAP3K1	ssc-miR-192	GO:0006915
ENSSSCG00000029828	RAB5B	ssc-miR-192	GO:0007264
ENSSSCG00000014362	HBEGF	ssc-miR-192	GO:0008284
ENSSSCG00000029852	WNT5A	ssc-miR-192	GO:0008284
ENSSSCG00000016628	WNT2	ssc-miR-192	GO:0008284
ENSSSCG00000016832	IL7R	ssc-miR-192	GO:0009897
ENSSSCG00000014362	HBEGF	ssc-miR-192	GO:0016477
ENSSSCG00000029852	WNT5A	ssc-miR-192	GO:0016477
ENSSSCG00000003194	NR5A1	ssc-miR-192	GO:0016477
ENSSSCG00000009051	IL15	ssc-miR-192	GO:0016607
ENSSSCG00000029828	RAB5B	ssc-miR-192	GO:0043231
ENSSSCG00000009088	IL2	ssc-miR-192	GO:0045944
ENSSSCG00000029852	WNT5A	ssc-miR-192	GO:0045944
ENSSSCG00000028420	EIF4E	ssc-miR-192	GO:0048471
ENSSSCG00000029828	RAB5B	ssc-miR-192	GO:0070062
ENSSSCG00000028420	EIF4E	ssc-miR-192	GO:0070062
ENSSSCG00000029852	WNT5A	ssc-miR-192	GO:0071560
ENSSSCG00000016628	WNT2	ssc-miR-192	GO:0071560
ENSSSCG00000016628	WNT2	ssc-miR-192	GO:0090263
ENSSSCG00000004789	THBS1	ssc-miR-194a_R+2	GO:0000187
ENSSSCG00000020672	F2R	ssc-miR-194a_R+2	GO:0000187
ENSSSCG00000004917	MALT1	ssc-miR-194a_R+2	GO:0001650
ENSSSCG00000006095	CCNE2	ssc-miR-194a_R+2	GO:0005634

ENSSSCG00000007058	PLCB4	ssc-miR-194a_R+2	GO:0005634
ENSSSCG00000016991	DUSP1	ssc-miR-194a_R+2	GO:0005634
ENSSSCG00000028968	RAC1	ssc-miR-194a_R+2	GO:0005634
ENSSSCG00000000529	DNM1L	ssc-miR-194a_R+2	GO:0005634
ENSSSCG00000001050	EDN1	ssc-miR-194a_R+2	GO:0005634
ENSSSCG00000004018	AFDN	ssc-miR-194a_R+2	GO:0005654
ENSSSCG00000004917	MALT1	ssc-miR-194a_R+2	GO:0005730
ENSSSCG00000016991	DUSP1	ssc-miR-194a_R+2	GO:0005737
ENSSSCG00000001050	EDN1	ssc-miR-194a_R+2	GO:0005737
ENSSSCG00000004018	AFDN	ssc-miR-194a_R+2	GO:0005737
ENSSSCG00000014878	PAK1	ssc-miR-194a_R+2	GO:0005737
ENSSSCG00000028968	RAC1	ssc-miR-194a_R+2	GO:0005737
ENSSSCG00000000529	DNM1L	ssc-miR-194a_R+2	GO:0005737
ENSSSCG00000000529	DNM1L	ssc-miR-194a_R+2	GO:0005737
ENSSSCG00000004917	MALT1	ssc-miR-194a_R+2	GO:0005737
ENSSSCG00000014878	PAK1	ssc-miR-194a_R+2	GO:0005737
ENSSSCG00000014878	PAK1	ssc-miR-194a_R+2	GO:0005737
ENSSSCG00000000529	DNM1L	ssc-miR-194a_R+2	GO:0005739
ENSSSCG00000000529	DNM1L	ssc-miR-194a_R+2	GO:0005739
ENSSSCG00000000529	DNM1L	ssc-miR-194a_R+2	GO:0005829
ENSSSCG00000003863	ZFYVE9	ssc-miR-194a_R+2	GO:0005829
ENSSSCG00000004018	AFDN	ssc-miR-194a_R+2	GO:0005829
ENSSSCG00000014878	PAK1	ssc-miR-194a_R+2	GO:0005829
ENSSSCG00000000529	DNM1L	ssc-miR-194a_R+2	GO:0005829
ENSSSCG00000004917	MALT1	ssc-miR-194a_R+2	GO:0005829
ENSSSCG00000014878	PAK1	ssc-miR-194a_R+2	GO:0005829
ENSSSCG00000014878	PAK1	ssc-miR-194a_R+2	GO:0005829
ENSSSCG00000003863	ZFYVE9	ssc-miR-194a_R+2	GO:0007179
ENSSSCG00000014127	RASGRF2	ssc-miR-194a_R+2	GO:0007264
ENSSSCG00000028968	RAC1	ssc-miR-194a_R+2	GO:0007264
ENSSSCG00000027443	MRAS	ssc-miR-194a_R+2	GO:0007264
ENSSSCG00000001050	EDN1	ssc-miR-194a_R+2	GO:0008284
ENSSSCG00000029852	WNT5A	ssc-miR-194a_R+2	GO:0008284
ENSSSCG00000004789	THBS1	ssc-miR-194a_R+2	GO:0008284
ENSSSCG00000020672	F2R	ssc-miR-194a_R+2	GO:0008284
ENSSSCG00000014878	PAK1	ssc-miR-194a_R+2	GO:0008284
ENSSSCG00000014878	PAK1	ssc-miR-194a_R+2	GO:0008284
ENSSSCG00000016832	IL7R	ssc-miR-194a_R+2	GO:0009897
ENSSSCG00000004789	THBS1	ssc-miR-194a_R+2	GO:0009897
ENSSSCG00000007501	BMP7	ssc-miR-194a_R+2	GO:0010942
ENSSSCG00000029852	WNT5A	ssc-miR-194a_R+2	GO:0016477
ENSSSCG00000004018	AFDN	ssc-miR-194a_R+2	GO:0016607
ENSSSCG00000014878	PAK1	ssc-miR-194a_R+2	GO:0016740
ENSSSCG00000014878	PAK1	ssc-miR-194a_R+2	GO:0016740

ENSSSCG00000014878	PAK1	ssc-miR-194a_R+2	GO:0016740
ENSSSCG00000007058	PLCB4	ssc-miR-194a_R+2	GO:0035556
ENSSSCG00000003863	ZFYVE9	ssc-miR-194a_R+2	GO:0042802
ENSSSCG00000007501	BMP7	ssc-miR-194a_R+2	GO:0043065
ENSSSCG00000000529	DNM1L	ssc-miR-194a_R+2	GO:0043065
ENSSSCG00000000529	DNM1L	ssc-miR-194a_R+2	GO:0043065
ENSSSCG00000004917	MALT1	ssc-miR-194a_R+2	GO:0043123
ENSSSCG00000020672	F2R	ssc-miR-194a_R+2	GO:0043123
ENSSSCG00000003863	ZFYVE9	ssc-miR-194a_R+2	GO:0043231
ENSSSCG00000000529	DNM1L	ssc-miR-194a_R+2	GO:0043231
ENSSSCG00000000529	DNM1L	ssc-miR-194a_R+2	GO:0043231
ENSSSCG00000001050	EDN1	ssc-miR-194a_R+2	GO:0045944
ENSSSCG00000007501	BMP7	ssc-miR-194a_R+2	GO:0045944
ENSSSCG00000029852	WNT5A	ssc-miR-194a_R+2	GO:0045944
ENSSSCG00000004018	AFDN	ssc-miR-194a_R+2	GO:0045944
ENSSSCG00000004789	THBS1	ssc-miR-194a_R+2	GO:0048471
ENSSSCG00000027443	MRAS	ssc-miR-194a_R+2	GO:0070062
ENSSSCG00000028968	RAC1	ssc-miR-194a_R+2	GO:0070062
ENSSSCG00000004789	THBS1	ssc-miR-194a_R+2	GO:0070062
ENSSSCG00000006095	CCNE2	ssc-miR-194a_R+2	GO:0070062
ENSSSCG00000029852	WNT5A	ssc-miR-194a_R+2	GO:0071560
ENSSSCG00000004789	THBS1	ssc-miR-194a_R+2	GO:1902043
ENSSSCG00000004789	THBS1	ssc-miR-194b-5p_1ss10GA	GO:0000187
ENSSSCG00000020672	F2R	ssc-miR-194b-5p_1ss10GA	GO:0000187
ENSSSCG00000004917	MALT1	ssc-miR-194b-5p_1ss10GA	GO:0001650
ENSSSCG00000006095	CCNE2	ssc-miR-194b-5p_1ss10GA	GO:0005634
ENSSSCG00000007058	PLCB4	ssc-miR-194b-5p_1ss10GA	GO:0005634
ENSSSCG00000028968	RAC1	ssc-miR-194b-5p_1ss10GA	GO:0005634
ENSSSCG00000000529	DNM1L	ssc-miR-194b-5p_1ss10GA	GO:0005634
ENSSSCG00000001050	EDN1	ssc-miR-194b-5p_1ss10GA	GO:0005634
ENSSSCG00000004917	MALT1	ssc-miR-194b-5p_1ss10GA	GO:0005730
ENSSSCG00000001050	EDN1	ssc-miR-194b-5p_1ss10GA	GO:0005737
ENSSSCG00000004018	AFDN	ssc-miR-194b-5p_1ss10GA	GO:0005737
ENSSSCG00000028968	RAC1	ssc-miR-194b-5p_1ss10GA	GO:0005737
ENSSSCG00000014878	PAK1	ssc-miR-194b-5p_1ss10GA	GO:0005737
ENSSSCG00000000529	DNM1L	ssc-miR-194b-5p_1ss10GA	GO:0005737
ENSSSCG00000014878	PAK1	ssc-miR-194b-5p_1ss10GA	GO:0005737
ENSSSCG00000000529	DNM1L	ssc-miR-194b-5p_1ss10GA	GO:0005737
ENSSSCG00000004917	MALT1	ssc-miR-194b-5p_1ss10GA	GO:0005737
ENSSSCG00000014878	PAK1	ssc-miR-194b-5p_1ss10GA	GO:0005737
ENSSSCG00000000529	DNM1L	ssc-miR-194b-5p_1ss10GA	GO:0005739
ENSSSCG00000000529	DNM1L	ssc-miR-194b-5p_1ss10GA	GO:0005739
ENSSSCG00000000529	DNM1L	ssc-miR-194b-5p_1ss10GA	GO:0005829
ENSSSCG00000003863	ZFYVE9	ssc-miR-194b-5p_1ss10GA	GO:0005829

ENSSSCG00000004018	AFDN	ssc-miR-194b-5p_1ss10GA	GO:0005829
ENSSSCG00000014878	PAK1	ssc-miR-194b-5p_1ss10GA	GO:0005829
ENSSSCG00000000529	DNM1L	ssc-miR-194b-5p_1ss10GA	GO:0005829
ENSSSCG00000004917	MALT1	ssc-miR-194b-5p_1ss10GA	GO:0005829
ENSSSCG00000014878	PAK1	ssc-miR-194b-5p_1ss10GA	GO:0005829
ENSSSCG00000003863	ZFYVE9	ssc-miR-194b-5p_1ss10GA	GO:0007179
ENSSSCG00000014127	RASGRF2	ssc-miR-194b-5p_1ss10GA	GO:0007264
ENSSSCG00000028968	RAC1	ssc-miR-194b-5p_1ss10GA	GO:0007264
ENSSSCG00000027443	MRAS	ssc-miR-194b-5p_1ss10GA	GO:0007264
ENSSSCG00000001050	EDN1	ssc-miR-194b-5p_1ss10GA	GO:0008284
ENSSSCG00000029852	WNT5A	ssc-miR-194b-5p_1ss10GA	GO:0008284
ENSSSCG00000014878	PAK1	ssc-miR-194b-5p_1ss10GA	GO:0008284
ENSSSCG00000004789	THBS1	ssc-miR-194b-5p_1ss10GA	GO:0008284
ENSSSCG00000014878	PAK1	ssc-miR-194b-5p_1ss10GA	GO:0008284
ENSSSCG00000020672	F2R	ssc-miR-194b-5p_1ss10GA	GO:0008284
ENSSSCG00000014878	PAK1	ssc-miR-194b-5p_1ss10GA	GO:0008284
ENSSSCG00000016832	IL7R	ssc-miR-194b-5p_1ss10GA	GO:0009897
ENSSSCG00000004789	THBS1	ssc-miR-194b-5p_1ss10GA	GO:0009897
ENSSSCG00000007501	BMP7	ssc-miR-194b-5p_1ss10GA	GO:0010942
ENSSSCG00000029852	WNT5A	ssc-miR-194b-5p_1ss10GA	GO:0016477
ENSSSCG00000004018	AFDN	ssc-miR-194b-5p_1ss10GA	GO:0016607
ENSSSCG00000014878	PAK1	ssc-miR-194b-5p_1ss10GA	GO:0016740
ENSSSCG00000014878	PAK1	ssc-miR-194b-5p_1ss10GA	GO:0016740
ENSSSCG00000014878	PAK1	ssc-miR-194b-5p_1ss10GA	GO:0016740
ENSSSCG00000007058	PLCB4	ssc-miR-194b-5p_1ss10GA	GO:0035556
ENSSSCG00000003863	ZFYVE9	ssc-miR-194b-5p_1ss10GA	GO:0042802
ENSSSCG00000007501	BMP7	ssc-miR-194b-5p_1ss10GA	GO:0043065
ENSSSCG00000000529	DNM1L	ssc-miR-194b-5p_1ss10GA	GO:0043065
ENSSSCG00000000529	DNM1L	ssc-miR-194b-5p_1ss10GA	GO:0043065
ENSSSCG00000004917	MALT1	ssc-miR-194b-5p_1ss10GA	GO:0043123
ENSSSCG00000020672	F2R	ssc-miR-194b-5p_1ss10GA	GO:0043123
ENSSSCG00000003863	ZFYVE9	ssc-miR-194b-5p_1ss10GA	GO:0043231
ENSSSCG00000000529	DNM1L	ssc-miR-194b-5p_1ss10GA	GO:0043231
ENSSSCG00000000529	DNM1L	ssc-miR-194b-5p_1ss10GA	GO:0043231
ENSSSCG00000001050	EDN1	ssc-miR-194b-5p_1ss10GA	GO:0045944
ENSSSCG00000007501	BMP7	ssc-miR-194b-5p_1ss10GA	GO:0045944
ENSSSCG00000029852	WNT5A	ssc-miR-194b-5p_1ss10GA	GO:0045944
ENSSSCG00000004018	AFDN	ssc-miR-194b-5p_1ss10GA	GO:0045944
ENSSSCG00000004789	THBS1	ssc-miR-194b-5p_1ss10GA	GO:0048471
ENSSSCG00000027443	MRAS	ssc-miR-194b-5p_1ss10GA	GO:0070062
ENSSSCG00000028968	RAC1	ssc-miR-194b-5p_1ss10GA	GO:0070062
ENSSSCG00000004789	THBS1	ssc-miR-194b-5p_1ss10GA	GO:0070062
ENSSSCG00000029852	WNT5A	ssc-miR-194b-5p_1ss10GA	GO:0071560
ENSSSCG00000006095	CCNE2	ssc-miR-194b-5p_1ss10GA	GO:0097190

ENSSSCG00000004789	THBS1	ssc-miR-194b-5p_1ss10GA	GO:1902043
ENSSSCG00000009051	IL15	ssc-miR-215_R+1	GO:0005654
ENSSSCG00000003194	NR5A1	ssc-miR-215_R+1	GO:0005737
ENSSSCG00000009051	IL15	ssc-miR-215_R+1	GO:0005737
ENSSSCG00000016918	MAP3K1	ssc-miR-215_R+1	GO:0005737
ENSSSCG00000028420	EIF4E	ssc-miR-215_R+1	GO:0005737
ENSSSCG00000016628	WNT2	ssc-miR-215_R+1	GO:0005737
ENSSSCG00000003194	NR5A1	ssc-miR-215_R+1	GO:0005829
ENSSSCG00000004110	TAB2	ssc-miR-215_R+1	GO:0005829
ENSSSCG00000009051	IL15	ssc-miR-215_R+1	GO:0005829
ENSSSCG00000028420	EIF4E	ssc-miR-215_R+1	GO:0005829
ENSSSCG00000016918	MAP3K1	ssc-miR-215_R+1	GO:0006915
ENSSSCG00000029828	RAB5B	ssc-miR-215_R+1	GO:0007264
ENSSSCG00000029852	WNT5A	ssc-miR-215_R+1	GO:0008284
ENSSSCG00000016628	WNT2	ssc-miR-215_R+1	GO:0008284
ENSSSCG00000016832	IL7R	ssc-miR-215_R+1	GO:0009897
ENSSSCG00000029852	WNT5A	ssc-miR-215_R+1	GO:0016477
ENSSSCG00000003194	NR5A1	ssc-miR-215_R+1	GO:0016477
ENSSSCG00000009051	IL15	ssc-miR-215_R+1	GO:0016607
ENSSSCG00000004110	TAB2	ssc-miR-215_R+1	GO:0043123
ENSSSCG00000029828	RAB5B	ssc-miR-215_R+1	GO:0043231
ENSSSCG00000009088	IL2	ssc-miR-215_R+1	GO:0045944
ENSSSCG00000029852	WNT5A	ssc-miR-215_R+1	GO:0045944
ENSSSCG00000004110	TAB2	ssc-miR-215_R+1	GO:0045944
ENSSSCG00000028420	EIF4E	ssc-miR-215_R+1	GO:0048471
ENSSSCG00000029828	RAB5B	ssc-miR-215_R+1	GO:0070062
ENSSSCG00000028420	EIF4E	ssc-miR-215_R+1	GO:0070062
ENSSSCG00000029852	WNT5A	ssc-miR-215_R+1	GO:0071560
ENSSSCG00000016628	WNT2	ssc-miR-215_R+1	GO:0071560
ENSSSCG00000016628	WNT2	ssc-miR-215_R+1	GO:0090263
ENSSSCG00000014339	CTNNA1	ssc-mir-4332-p5_1ss18CA	GO:0003723
ENSSSCG00000000107	CSNK1E	ssc-mir-4332-p5_1ss18CA	GO:0003723
ENSSSCG00000000233	ACVR1B	ssc-mir-4332-p5_1ss18CA	GO:0003723
ENSSSCG00000002804	CSNK2A2	ssc-mir-4332-p5_1ss18CA	GO:0005634
ENSSSCG00000012981	NR5A1	ssc-mir-4332-p5_1ss18CA	GO:0005634
ENSSSCG00000000107	CSNK1E	ssc-mir-4332-p5_1ss18CA	GO:0005634
ENSSSCG00000004022	RPS6KA2	ssc-mir-4332-p5_1ss18CA	GO:0005634
ENSSSCG00000001009	RIPK1	ssc-mir-4332-p5_1ss18CA	GO:0005634
ENSSSCG00000001027	BMP6	ssc-mir-4332-p5_1ss18CA	GO:0005634
ENSSSCG00000001242	GABBR1	ssc-mir-4332-p5_1ss18CA	GO:0005634
ENSSSCG00000001506	NR5A1	ssc-mir-4332-p5_1ss18CA	GO:0005730
ENSSSCG00000001242	GABBR1	ssc-mir-4332-p5_1ss18CA	GO:0005737
ENSSSCG00000004022	RPS6KA2	ssc-mir-4332-p5_1ss18CA	GO:0005737
ENSSSCG00000012981	NR5A1	ssc-mir-4332-p5_1ss18CA	GO:0005737

ENSSSCG00000013269	MAPK8IP1	ssc-mir-4332-p5_1ss18CA	GO:0005737
ENSSSCG00000000107	CSNK1E	ssc-mir-4332-p5_1ss18CA	GO:0005737
ENSSSCG00000005661	NR5A1	ssc-mir-4332-p5_1ss18CA	GO:0005739
ENSSSCG00000001009	RIPK1	ssc-mir-4332-p5_1ss18CA	GO:0005739
ENSSSCG00000000233	ACVR1B	ssc-mir-4332-p5_1ss18CA	GO:0005829
ENSSSCG00000001009	RIPK1	ssc-mir-4332-p5_1ss18CA	GO:0005829
ENSSSCG00000007030	IKBKB	ssc-mir-4332-p5_1ss18CA	GO:0005829
ENSSSCG00000012981	NR5A1	ssc-mir-4332-p5_1ss18CA	GO:0005829
ENSSSCG00000013033	BAD	ssc-mir-4332-p5_1ss18CA	GO:0005829
ENSSSCG00000013269	MAPK8IP1	ssc-mir-4332-p5_1ss18CA	GO:0005829
ENSSSCG00000014339	CTNNA1	ssc-mir-4332-p5_1ss18CA	GO:0005829
ENSSSCG00000007520	GNAS	ssc-mir-4332-p5_1ss18CA	GO:0005829
ENSSSCG00000007520	GNAS	ssc-mir-4332-p5_1ss18CA	GO:0005829
ENSSSCG00000007520	GNAS	ssc-mir-4332-p5_1ss18CA	GO:0005829
ENSSSCG00000007520	GNAS	ssc-mir-4332-p5_1ss18CA	GO:0005829
ENSSSCG00000007520	GNAS	ssc-mir-4332-p5_1ss18CA	GO:0005829
ENSSSCG00000007520	GNAS	ssc-mir-4332-p5_1ss18CA	GO:0005829
ENSSSCG00000007520	GNAS	ssc-mir-4332-p5_1ss18CA	GO:0005829
ENSSSCG00000013033	BAD	ssc-mir-4332-p5_1ss18CA	GO:0006915
ENSSSCG00000001009	RIPK1	ssc-mir-4332-p5_1ss18CA	GO:0006915
ENSSSCG00000001506	NR5A1	ssc-mir-4332-p5_1ss18CA	GO:0007264
ENSSSCG00000017482	CSF3	ssc-mir-4332-p5_1ss18CA	GO:0008284
ENSSSCG00000000293	ITGA5	ssc-mir-4332-p5_1ss18CA	GO:0009897
ENSSSCG00000000293	ITGA5	ssc-mir-4332-p5_1ss18CA	GO:0009897
ENSSSCG00000000293	ITGA5	ssc-mir-4332-p5_1ss18CA	GO:0009897
ENSSSCG00000002804	CSNK2A2	ssc-mir-4332-p5_1ss18CA	GO:0016477
ENSSSCG00000010872	AKT3	ssc-mir-4332-p5_1ss18CA	GO:0016740
ENSSSCG00000000233	ACVR1B	ssc-mir-4332-p5_1ss18CA	GO:0016740
ENSSSCG00000004022	RPS6KA2	ssc-mir-4332-p5_1ss18CA	GO:0016740
ENSSSCG00000004022	RPS6KA2	ssc-mir-4332-p5_1ss18CA	GO:0035556
ENSSSCG00000010872	AKT3	ssc-mir-4332-p5_1ss18CA	GO:0035556
ENSSSCG00000021865	INHBA	ssc-mir-4332-p5_1ss18CA	GO:0042802
ENSSSCG00000005661	NR5A1	ssc-mir-4332-p5_1ss18CA	GO:0043065
ENSSSCG00000001009	RIPK1	ssc-mir-4332-p5_1ss18CA	GO:0043065
ENSSSCG00000013033	BAD	ssc-mir-4332-p5_1ss18CA	GO:0043065
ENSSSCG00000000293	ITGA5	ssc-mir-4332-p5_1ss18CA	GO:0043123
ENSSSCG00000001009	RIPK1	ssc-mir-4332-p5_1ss18CA	GO:0043123
ENSSSCG00000014339	CTNNA1	ssc-mir-4332-p5_1ss18CA	GO:0043231
ENSSSCG00000001027	BMP6	ssc-mir-4332-p5_1ss18CA	GO:0045944
ENSSSCG00000007030	IKBKB	ssc-mir-4332-p5_1ss18CA	GO:0045944
ENSSSCG00000021865	INHBA	ssc-mir-4332-p5_1ss18CA	GO:0045944
ENSSSCG00000012981	NR5A1	ssc-mir-4332-p5_1ss18CA	GO:0045944
ENSSSCG00000004022	RPS6KA2	ssc-mir-4332-p5_1ss18CA	GO:0045944
ENSSSCG00000021865	INHBA	ssc-mir-4332-p5_1ss18CA	GO:0048471

ENSSSCG00000005661	NR5A1	ssc-mir-4332-p5_1ss18CA	GO:0048471
ENSSSCG00000000233	ACVR1B	ssc-mir-4332-p5_1ss18CA	GO:0070062
ENSSSCG00000007520	GNAS	ssc-mir-4332-p5_1ss18CA	GO:0070062
ENSSSCG00000007520	GNAS	ssc-mir-4332-p5_1ss18CA	GO:0070062
ENSSSCG00000007520	GNAS	ssc-mir-4332-p5_1ss18CA	GO:0070062
ENSSSCG00000007520	GNAS	ssc-mir-4332-p5_1ss18CA	GO:0070062
ENSSSCG00000007520	GNAS	ssc-mir-4332-p5_1ss18CA	GO:0070062
ENSSSCG00000007520	GNAS	ssc-mir-4332-p5_1ss18CA	GO:0070062
ENSSSCG00000007520	GNAS	ssc-mir-4332-p5_1ss18CA	GO:0070062
ENSSSCG00000009545	COL4A2	ssc-mir-4332-p5_1ss18CA	GO:0070062
ENSSSCG00000009545	COL4A2	ssc-mir-4332-p5_1ss18CA	GO:0071560
ENSSSCG00000000107	CSNK1E	ssc-mir-4332-p5_1ss18CA	GO:0090263
ENSSSCG00000013033	BAD	ssc-mir-4332-p5_1ss18CA	GO:0097190
ENSSSCG00000014339	CTNNA1	ssc-mir-4332-p5_1ss18CA	GO:2001241
ENSSSCG00000021865	INHBA	ssc-mir-4332-p5_1ss18CA	GO:2001241
